# *ICN_Atlas*: Automated description and quantification of functional MRI activation patterns in the framework of intrinsic connectivity networks

**DOI:** 10.1016/j.neuroimage.2017.09.014

**Published:** 2017-12

**Authors:** Lajos R. Kozák, Louis André van Graan, Umair J. Chaudhary, Ádám György Szabó, Louis Lemieux

**Affiliations:** a MR Research Center, Semmelweis University, 1085, Budapest, Hungary; b Department of Clinical and Experimental Epilepsy, UCL Institute of Neurology, University College London, WC1N 3BG, London, UK; c Epilepsy Society, SL9 0RJ Chalfont St. Peter, Buckinghamshire, UK

**Keywords:** Brain atlas, Functional characterization, Functional magnetic resonance imaging, Resting-state networks, Intrinsic connectivity networks, Data analysis

## Abstract

Generally, the interpretation of functional MRI (fMRI) activation maps continues to rely on assessing their relationship to anatomical structures, mostly in a qualitative and often subjective way. Recently, the existence of persistent and stable brain networks of functional nature has been revealed; in particular these so-called intrinsic connectivity networks (ICNs) appear to link patterns of resting state and task-related state connectivity. These networks provide an opportunity of functionally-derived description and interpretation of fMRI maps, that may be especially important in cases where the maps are predominantly task-unrelated, such as studies of spontaneous brain activity e.g. in the case of seizure-related fMRI maps in epilepsy patients or sleep states. Here we present a new toolbox (*ICN_Atlas*) aimed at facilitating the interpretation of fMRI data in the context of ICN. More specifically, the new methodology was designed to describe fMRI maps in function-oriented, objective and quantitative way using a set of 15 metrics conceived to quantify the degree of ‘engagement’ of ICNs for any given fMRI-derived statistical map of interest. We demonstrate that the proposed framework provides a highly reliable quantification of fMRI activation maps using a publicly available longitudinal (test-retest) resting-state fMRI dataset. The utility of the *ICN_Atlas* is also illustrated on a parametric task-modulation fMRI dataset, and on a dataset of a patient who had repeated seizures during resting-state fMRI, confirmed on simultaneously recorded EEG. The proposed *ICN_Atlas* toolbox is freely available for download at http://icnatlas.com and at http://www.nitrc.org for researchers to use in their fMRI investigations.

## Highlights

•fMRI activation maps are usually described in relation to anatomical structures.
•*ICN_Atlas* describes fMRI data in the framework of intrinsic connectivity networks (ICN).
•*ICN_Atlas* output: ICN ‘engagement’ (spatial and activation strength) metrics.
•*ICN_Atlas* shows high ICN engagement reliability on test-retest resting-state fMRI data.
•Method demonstrated (1) on parametric task-modulation fMRI data set shows differential engagement of ICN with respect to task difficulty, and (2) on epileptic seizure EEG-fMRI data shows compelling evolution of ICN engagement patterns.

## Graphical abstract

**Figure:**
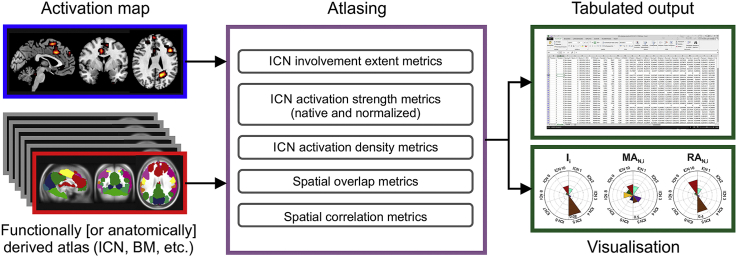

## Abbreviations

AAL: automated anatomical labelling
ANOVA: analysis of variance
BOLD: blood oxygenation-level dependent
DMN: default mode network
EEG: electroencephalography
EFA: exploratory factor analysis
EPI: echo-planar imaging
fMRI: functional magnetic resonance imaging
FWHM: full width at half maximum
GLM: generalized linear model
I_i_: ICN_i_ Spatial Involvement
${\text{I}}_{\text{i}}^{\text{M}}$: Normalised Mean ICN_i_ Activation Density
I_T_: Total ICN Spatial Involvement
${\text{I}}_{\text{T}}^{\text{M}}$: Normalised Global Mean ICN Activation Density
IC: independent component
ICA: independent component analysis
ICC: intra-class correlation coefficient
ICC_B_: between-session ICC
ICC_W_: within-session ICC
ICN: intrinsic connectivity network
IR_i_: ICN_i_ Relative Spatial Involvement
${\text{IR}}_{\text{i}}^{\text{M}}$: Relative Normalised Mean ICN_i_ Activation
J_i_: Jaccard index with ICN_i_
MA: Global Mean ICN Activation
MA_i_: Mean ICN_i_ Activation
MA_N_: Normalised Global Mean ICN Activation
MA_N,i_: Normalised Mean ICN_i_ Activation
MELODIC: Multivariate Exploratory Linear Optimized Decomposition into Independent Components, ICA analysis tool
MNI: Montreal Neurological Institute
MRI: magnetic resonance imaging
NIfTI: Neuroimaging Informatics Technology Initiative
NYU: New York University
NYU-TRT: NYU resting-state fMRI test-retest data
OL_i_: Spatial Overlap with ICN_i_
PCA: principal component analysis
r_i_: Pearson's spatial correlation with ICN_i_
RA_N,i_: Normalised Relative ICN_i_ Activation
rs-fMRI: resting-state fMRI
RSN: resting state network
SPM: statistical parametric map/Statistical Parametric Mapping
SQ_i_: Sørensen-Dice coefficient with ICN_i_
TC-GICA: temporally concatenated group ICA
TRT: test-retest

## 1 Introduction

The analysis and interpretation of functional MRI data activation patterns is usually performed in the framework of brain anatomy. In particular, activation clusters are usually described in terms of their extent and centre of gravity coordinates as defined in standard template spaces, e.g. MNI (Montreal Neurological Institute) or Talairach (Evans et al., 1993, Fox and Lancaster, 2002, Talairach and Tournoux, 1988). A variety of macro- and micro-structural atlasing approaches have been proposed to relate activation clusters to anatomical landmarks, e.g. automated anatomical labelling or parcellations based on gyral and sulcal structure (Damoiseaux et al., 2006, Tzourio-Mazoyer et al., 2002), or on cytoarchitectonic structure, e.g. the Talairach Demon or the SPM Anatomy toolbox (Eickhoff et al., 2005, Lancaster et al., 2000).

Another widely used approach to the description of fMRI activation patterns is based on functional localizers. For example, a target area is identified through a separate localisation measurement after which activations of interest are described with respect to the localizer's functional activations (Saxe et al., 2006). There is some criticism regarding the improper use of functional localizers, especially when used to constrain the analyses per se or due to the risk of circularity (Friston et al., 2006, Kriegeskorte et al., 2009). Furthermore, in the context of pathological activity and in particular in view of the spatio-temporal heterogeneity of epileptic activity-related BOLD patterns this approach may be sub-optimal since it may not provide a comprehensive mapping of all relevant activation foci.

Recent developments showing the correspondence of maps obtained with resting-state and task-based fMRI (Laird et al., 2011, Ray et al., 2013, Smith et al., 2009) may provide a solid background for developing a whole-brain functional networks-based atlasing tool for the interpretation of BOLD patterns derived either from task-based or task-free measurements. Specifically, the pattern of low frequency correlations in the resting brain have been shown to form well identifiable intrinsic connectivity networks (ICNs) or resting state networks (RSN) (Beckmann et al., 2005, Biswal et al., 1995, Laird et al., 2011). ICNs are spatially segregated areas representing underlying functional connectivity (Fox and Raichle, 2007), i.e. intrinsic connectivity, which is important for development, maintenance, and function of the brain (Doria et al., 2010, Pizoli et al., 2011, Raichle, 2010, Raichle and Mintun, 2006, Supekar et al., 2010, Zielinski et al., 2010). As functional units they show synchronized BOLD fluctuations both at rest and while performing specific tasks (Damoiseaux et al., 2006, Laird et al., 2011, Smith et al., 2009). These networks have been observed consistently across imaging sessions (Biswal et al., 2010, Shehzad et al., 2009, Zuo et al., 2010b) and between subjects (Damoiseaux et al., 2006, Shehzad et al., 2009) and can essentially be seen as forming two large anti-correlated systems corresponding to task disengagement and task engagement, respectively; the former is the so-called default mode network (DMN) and the latter is composed of several task-based networks: somatosensory, visual, or attention ICN, etc. (Chai et al., 2012, Golland et al., 2008, Power et al., 2011, Zhang et al., 2011). Data-driven meta-analyses of task-activation data have shown a strong correspondence between the configurations of RSNs and task-based fMRI co-activations both for low and high independent component analysis (ICA) model orders (Laird et al., 2011, Ray et al., 2013, Smith et al., 2009).

In the field of epilepsy, there is an increasing interest of a functional network-based interpretation of the pathological activity. In the particular case of fMRI localisation of epileptic events and discharges (such as observed on simultaneously-recorded EEG) a functionally-derived framework may be more appropriate than an anatomical approach, specifically for the discussion of EEG discharge-related activation and deactivation patterns (Chaudhary et al., 2012), given the relationship between activation patterns and the seizure's clinical signs (semiology) (Chaudhary et al., 2012, Thornton et al., 2010, Tyvaert et al., 2008). Several studies employing independent component analysis to derive spatio-temporal components related to epileptic discharges evidenced networked activation/deactivation patterns partly overlapping and coexisting with ICN-related components (LeVan et al., 2010, Moeller et al., 2011, Rodionov et al., 2007, Thornton et al., 2010). There is also evidence for altered connectivity outside the core epileptic networks, affecting the ICNs possibly as an effect of epilepsy (Centeno and Carmichael, 2014). A study of BOLD changes associated with different electro-clinical phases of epileptic seizures has shown a link between involvement of the DMN and loss of consciousness (Chaudhary et al., 2012). A recently proposed framework emphasizes the importance of the proportion of change produced by epileptic transients relative to steady-state network connectivity in normal controls (Centeno and Carmichael, 2014). This underlines the necessity to interpret epileptic discharge-related activation with respect to the whole connectome.

Here we propose an atlasing tool, called *ICN_Atlas*, for the interpretation of BOLD maps based on the objective quantification of the degree of *engagement* of a set of intrinsic connectivity networks (used here as a set of atlas base maps). Specifically, we aimed to develop a means to describe activations in the framework of ICN by matching data to atlas templates in a similar fashion as anatomy-based atlases do and to calculate various measures of activation extent and level in relation to the chosen atlas maps. We first present the engagement quantification formalism, followed by a validation study and finally an illustration of the new tool's application in the study of epileptic networks.

### 1.1 Principles and implementation of *ICN_Atlas*

#### 1.1.1 The *ICN_Atlas* framework

*ICN_Atlas* is a collection of Matlab (Mathworks Inc., Natick, MA, USA) scripts that serves as an extension to the SPM toolbox (http://www.fil.ion.ucl.ac.uk/spm/) and, as such, works across multiple platforms (Windows PC, Unix, Mac). It is an extensible non-commercial package that is freely available at http://icnatlas.com and at http://www.nitrc.org. The aim was to provide a toolbox with atlasing capabilities analogous to previously published anatomical information-based tools such as the 3D Talairach atlas (Lancaster et al., 2000), or the Automated Anatomical Labelling (Tzourio-Mazoyer et al., 2002). The novelty of the framework lies in the following: (1) it uses functionally-derived atlas base maps based on ICNs; (2) it outputs a series of estimated activation-based metric values to describe the functional activations (input) based on intrinsic functional connectivity (embodied in the atlas base maps).

**Fig. 1:**
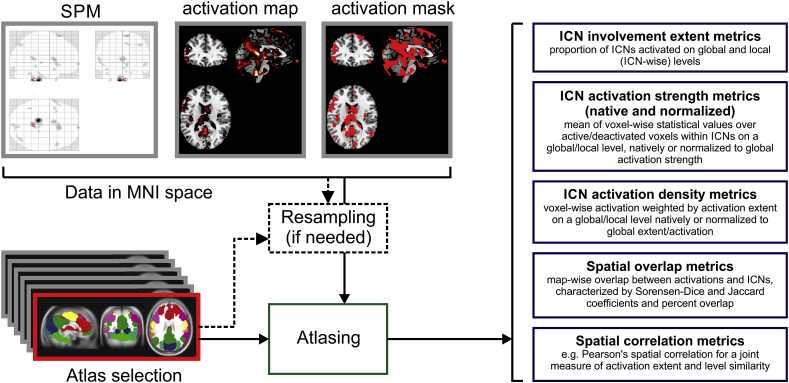
**Schematic of atlasing steps**. The input of the toolbox can either be an SPM in the workspace, a thresholded activation map or an activation mask, the input is then up-sampled and/or iso-voxel transformed if needed to match the selected atlas' resolution, then the output metrics are calculated.

In brief, *ICN_Atlas'* input consists of a volumetric statistical parametric map (SPM) representing an fMRI activation pattern (input map) and its output consists of a series of numeric values representing different measures of the map's degree of involvement for each atlas base map, for an overview see Fig. 1.

As the *ICN_Atlas* toolbox is integrated to the SPM toolbox environment its inputs can be either (1) the currently available SPM in the workspace, (2) exported SPM{T} and SPM{F} maps, or (3) any kind of data in Analyze of NIfTI format. The current version of the *ICN_Atlas* toolbox expects input data to be presented in the Montreal Neurological Institute (MNI) atlas space (Evans et al., 1993).

The atlasing algorithm performs labelling of the input map's active voxels (activation map) according to membership based on voxel-wise correspondence analysis of the activation map and the atlas base maps (see below), and calculates a series of overlap, activation extent, and activation density metrics (described below and in Appendix A) based on the labelling.

#### 1.1.2 Atlas base maps: ICN and anatomical atlases

In *ICN_Atlas*’ current implementation, three sets of ICN base atlases are available based on labelled Gaussianised statistical maps representing ICNs resulting from group-wise resting-state fMRI data (Smith et al., 2009) and BrainMap Project meta-analysis data (Laird et al., 2011, Smith et al., 2009), see below. In addition, an integer label map representing the whole brain Automated Anatomical Labelling (AAL) atlas is also included as an anatomical reference (Tzourio-Mazoyer et al., 2002). N.B., the atlasing framework is extensible and other atlases (either functionally or anatomically-derived, and/or atlases from other species) can easily be integrated.

The three sets of ICN base atlases are as follows:

SMITH10: the 10 adult ICNs based on ICA decomposition (*d* = 20) of resting-state fMRI data (http://fsl.fmrib.ox.ac.uk/analysis/brainmap+rsns/), where *d* is the dimensionality, representing the constraint on the number of independent spatio-temporal components (Smith et al., 2009);

BRAINMAP20: the 18 BrainMap co-activation networks and 2 noise/artefact components based on ICA decomposition (d = 20) of the BrainMap Project large-scale neuroimaging experiment meta-analysis data available at http://brainmap.org/icns/maps.zip (Laird et al., 2005, Laird et al., 2011).

BRAINMAP70: the 65 BrainMap co-activation networks and 5 noise/artefact components based on ICA decomposition (d = 70) of the BrainMap Project large-scale neuroimaging experiment meta-analysis data available at http://brainmap.org/icns/Archive.zip, (Laird et al., 2005, Ray et al., 2013).

For each of these, we have a set of K atlas base maps that represent ICNs (with the exception of 2 and 5 artefactual components for BRAINMAP20 and BRAINMAP70, respectively): $IC_{i}$ each being a statistical map corresponding to an ICA component (IC) which in turn corresponds to one of the K functionally stereotypical $ICN_{i}$ or artefactual component. Given this, an ICN-based atlas can be thought of as the union of ICN-specific statistical base maps, $ICN_{i}$:

(1) $$Atlas:\cup_{i}^{K}\left\{ICN_{i,n}\right\},\;with\;ICN_{i,n}=\{\;\begin{array}{c} \;\langle IC_{i,n}\rangle \;if\;IC_{i,n}>T\\ NaN\;if\;\langle IC_{i,n}\rangle \leq T \end{array}\text{,}$$

where $IC_{i,n}\;$ represents the Z-score of $IC_{i}$ at voxel n, and T is a user-defined threshold that defaults to Z=3 for both the SMITH10, BRAINMAP20 and BRAINMAP70 atlases. However, the prototype $ICN_{i}$ require further treatment for use in *ICN_Atlas* as base maps by assigning each voxel *n* a unique label $l_{n}$ corresponding to the index of the prototype $ICN_{i,n}$ with the highest Z-score. In other words each base atlas A is an array (represented by {}) of voxel labels l as follows:

(2) $$A=\left\{l_{n}\right\},$$

$$with\;l_{n}=\{\begin{array}{c} \arg\max_{j}\left(\langle ICN_{j,n}\rangle \right),\;where\;j\;runs\;from\;1\;to\;K,\;if\;n\;belongs\;to\;any\;of\;the\;K\;\;ICN_{i}\;\\ NaN,\;if\;n\;belongs\;to\;none\;of\;the\;K\;\;ICN_{i} \end{array}$$

where $x_{n}\;$ represents the Z-score of x at voxel n; therefore, $l_{n}$ has a value between 1 and K, or NaN. This scheme ensures that any given voxel belongs to at most one ICN or artefactual component.

Binary versions of the ICN base maps, $\;ICN_{i}^{B}$ can also be obtained as follows:

(3) $$ICN_{i,n}^{B}=\{\;\begin{array}{c} 1\;if\;\langle IC_{i,n}\rangle >T\\ 0\;if\langle \;IC_{i,n}\rangle \leq T \end{array}$$

Each of the resulting atlas base maps are then saved as a matrix of labels and Z-scores, plus information on the defining space (NIfTI affine coordinate definitions) and other descriptive data (including the atlas name and reference of origin.)

The anatomical atlas included with the *ICN_Atlas* tool, CONN132, is based on the *CONN:functional connectivity toolbox's* (https://www.nitrc.org/projects/conn) combined representation of the cortical and subcortical ROIs from the Harvard-Oxford Atlas (Desikan et al., 2006, Frazier et al., 2005, Goldstein et al., 2007, Makris et al., 2006) and the cerebellar ROIs from the AAL atlas (Tzourio-Mazoyer et al., 2002), transformed from 1 × 1x1mm to 2 × 2x2mm resolution using the SPM toolbox to match the functional atlases' base maps spatial characteristics.

#### 1.1.3 The input map labelling scheme

Voxel-wise labelling of the input maps is based on the label of the corresponding base map voxel:

(4) $$L_{n}=A_{n}\cap SPM_{n}$$

where *SPM* represents the input map, which can be thresholded ($SPM_{t}$) or unthresholded.

#### 1.1.4 ICN engagement metrics

In addition to the labelling scheme, in an attempt to capture the essence of ICN involvement embodied in the input map quantitatively as completely as possible, we considered a range of ICN ‘engagement’ metrics. The metrics were inspired firstly by basic descriptive spatial overlap statistics, and secondly by considering the statistical nature of the input maps; for example, the metric ***I***_***i***_ (*ICN*_*i*_ *Spatial Involvement*; see Equation (5) below) represents the ratio of activated ICN_*i*_ voxels to ICN_*i*_ volume and is purely spatial; another, ***MA***_***i***_ (*Mean ICN*_*i*_ *Activation*), is the ratio of the mean of voxel-wise statistical values over the number of activated voxels in ICN_*i*_. The metrics fall into the following categories: spatial extent (overlap), activation strength, activation density and correlation. Furthermore, the proposed metrics are either ICN-specific (vector quantities: one value for each ICN) or global (scalar quantities: calculated over all ICNs). A total of 11 ICN-specific metrics and 4 global metrics are implemented in *ICN_Atlas* and their definitions can be found in Appendix A. In the following, we focus on 4 metrics in order to simplify the presentation. This choice is informed by the results of a Factor Analysis (See section 2.2 ‘*Demonstration’)* aimed at identifying a parsimonious set of metrics that capture *and* summarise ICN engagement for a given dataset.

We used the following variables and symbols in the engagement metrics definitions:

• n: voxel index (*n* = 1, 2, …, *M*, where *M* is the number of voxels in the maps);
• |X|: is the number of non-zero valued voxels in X;
• $X_{n}$: is the statistical value of voxel n in X;
• : represent the voxel-wise product;
• i: represents the ICN index.

The following two metrics are designed to capture the degree of engagement of an ICN in a given input (activation) map in purely spatial terms:

**ICN**_**i**_ **Spatial Involvement (*I***_***i***_**)**: ratio of the number of activated ICN_*i*_ voxels ($\left|SPM_{t}⋂ICN_{i}\right|$) to ICN_*i*_ volume:

(5) $$I_{i}=\frac{\left|SPM_{t}⋂ICN_{i}\right|}{\left|ICN_{i}\right|}$$

In other words, *I*_*i*_ is the proportion of ICN_*i*_ that is activated in the input map.

**Total ICN Spatial Involvement (*I***_***T***_**)**: is a global metric expressing the ratio of the number of activated ICN voxels over the ICN volume over all ICNs:

(6) $$I_{T}=\frac{\sum_{i}\left|SPM_{t}⋂ICN_{i}\right|\sum_{i}}{\sum_{i}\left|ICN_{i}\right|}$$

The following two ICN_*i*_ engagement metrics take each voxel's statistical score (‘activation strength’) into consideration; these are designed to better distinguish between two input maps with similar degrees of spatial involvement of ICN_*i*_ (*I*_*i*_) but different activation strengths, each taking into account the input map's values in the ICNs in different ways:

**Normalised Mean ICN**_**i**_ **Activation (*MA***_***N,i***_**)**: mean of the normalised voxel-wise statistical values relative to the number of activated voxels in ICN_*i*_:

(7) $$MA_{N,i}=\frac{\sum_{n}\frac{{\langle SPM_{t}\rangle }_{n}\times ICN_{i,n}^{B}-\;min\;\langle SPM_{t}\rangle }{max\;\langle SPM_{t}\rangle -min\;\langle SPM_{t}\rangle }\;}{\left|SPM_{t}⋂ICN_{i}\right|}$$

where ${\langle SPM_{t}\rangle }_{n}$ represents the statistical value of input map voxel *n*; min〈*SPM*_*t*_*>* and max〈*SPM*_*t*_*>* are the minimum and maximum, respectively, input map statistical values within or outside the ICNs. The numerator therefore represents the input map's total statistical score within ICN_*i*_ (relative to the map's minimum statistical score), normalised to the range of statistical scores over the map. By dividing this by the number of activated ICN_*i*_ voxels ($\left|SPM_{t}⋂ICN_{i}\right|$) we obtain a measure of engagement ‘intensity’.

**Relative Normalised ICN**_**i**_ **Activation (*RA***_***N,i***_**)** is the ratio of the normalised mean activation in a given ICN over the total normalised ICN activation and has the same numerator as ***MA***_***N,i***_:

(8) $$RA_{N,i}=\frac{\sum_{n}\frac{{\langle SPM_{t}\rangle }_{n}\times ICN_{i,n}^{B}-\;min\;\langle SPM_{t}\rangle }{max\;\langle SPM_{t}\rangle -min\;\langle SPM_{t}\rangle }}{\sum_{j}\sum_{n}\frac{{\langle SPM_{t}\rangle }_{n}\times ICN_{j,n}^{B}-\;min\;\langle SPM_{t}\rangle }{max\;\langle SPM_{t}\rangle -min\;\langle SPM_{t}\rangle }}$$

The denominator being the sum of the numerator over all ICN, therefore representing the input map's total statistical ICN score, ***RA***_***N,i***_ is therefore a metric similar to ***MA***_***N,i***_ but that is relative to the engagement intensity of all ICNs.

The metrics are applicable either to input maps previously subjected to statistical significance thresholding (*SPM*_*t*_, as in the above definitions) or to ‘raw’ (un-thresholded) statistical maps. The former may be more appropriate for involvement metrics where the spatial extent of activation is the determining factor, while the latter can possibly be advantageous for activation metrics depending on the research question, e.g. to compare activation profiles over whole ICNs for different task or behavioural conditions.

#### 1.1.5 *ICN_Atlas* output

The toolbox's primary outputs consist of a table containing the values for all 11 ICN specific and 4 global metrics, and a range of visualization options in the form of bar charts and polar plots, some of which will be illustrated below.

## 2 Material and methods

This section consists of two parts: 1. Validation, on repeat resting-state fMRI scanning data from 25 healthy volunteers. 2. Demonstrations, of a methodology for the identification of a parsimonious set of *ICN_Atlas* engagement metrics in a particular fMRI dataset, and two illustrative applications of *ICN_Atlas* on task fMRI data and fMRI maps of epileptic seizures.

### 2.1 Validation

We validated the *ICN_Atlas* atlasing methodology using the New York University (NYU) resting-state test-retest fMRI dataset (https://www.nitrc.org/projects/nyu_trt), which consists of three rs-fMRI scans acquired in twenty-six participants (mean age 20.5 ± 4.8 years, 11 males) who had no history of psychiatric or neurological illness (in accordance with protocols approved by the institutional review boards of NYU and the NYU School of Medicine). The second and third scans were collected between 5 and 16 months (mean: 11) following the baseline scan, in a single scanning session 45 min apart (for details see Zuo et al., 2010b).

In summary, the validation process consists of: First, we performed group and individual-level ICA analyses of the NYU test-retest (NYU-TRT) data. The results of this analysis are sets of group-level and individual ICs that were subjected to atlasing using SMITH10, BRAINMAP20 and BRAINMAP70 as atlas base maps, to evaluate the proposed methodology's robustness in terms of its ability to identify functionally stereotypical ICNs. Second, we assessed *ICN_Atlas* atlasing repeatability by quantifying ICN engagement at the individual level across the repeat scans in the NYU dataset.

#### 2.1.1 Group- and individual-level IC analyses

Data pre-processing was performed using the spm8 toolbox (http://www.fil.ion.ucl.ac.uk/spm/software/spm8/) with the following steps: (1) realignment and unwarp, (2) normalization to MNI space using the spm8 EPI template as target image, (3) Gaussian spatial smoothing with 6 mm FWHM.

The pre-processed NYU dataset was then analysed by means of independent component analysis (ICA) using MELODIC (http://fsl.fmrib.ox.ac.uk/fsl/fslwiki/MELODIC) with the temporal concatenation group ICA (TC-GICA) approach (Beckmann et al., 2005) followed by dual regression, resulting in1500 (25 subjects * 3 sessions * 20 component) individual-level ICs (Beckmann et al., 2009). Data from the three scanning sessions were included in the same group ICA, and the number of resulting group-level independent components (IC) was limited to 20 (Smith et al., 2009, Zuo et al., 2010b).

##### 2.1.1.1 Group-level ICN engagement quantification

The resulting group-level IC statistical maps were then thresholded at *Z* > 3, and submitted to *ICN_Atlas* atlasing using the SMITH10, BRAIMAP20 and BRAINMAP70 atlases (all thresholded at *Z* > 3). Correspondence to the ICNs was quantified using the metrics ***I***_***i***_, ***MA***_***N,i***_ and ***RA***_***N,i***_, where the index *i* is the name of the relevant atlas base map, for example ***I***_***ICN9***_ represents ICN_i_ Spatial Involvement calculated based on ICN9 of the SMITH10 atlas and ***RA***_***N,BM20-8***_ represents Normalised Relative ICN_i_ Activation calculated based on BRAINMAP20 atlas co-activation network BM20-8, while ***MA***_***N,BM70-2***_ represents Normalised Mean ICN_i_ Activation calculated based on BRAINMAP70 atlas co-activation network BM70-2.

To obtain an overview of the agreement between base atlases we determined whether the highest three engagement values (for each metric) pertain to the same atlas base maps for any given IC (See Fig. 3 and Supplementary Fig. 1 for details). This number was chosen based on the fact that the top 3 values correspond to between 61-99% and 48–95% of the total ***I***_***i***_ for SMITH10 and BRAINMAP20 respectively, and between 21 and 80% of the total ***I***_***i***_ for BRAINMAP70 (see the last rows of Supplementary Tables 2–10 for details).

##### 2.1.1.2 Test-retest repeatability

###### 2.1.1.2.1 IC voxel-wise repeatability at the group level

Within- and between-session repeatability of the ICs were quantified as the mode of the intra-class correlation coefficient (<*ICC*_*W*_*>* and <*ICC*_*B*_>, respectively); *ICC* was calculated using a formula that does not penalize for systematic differences between scanning sessions (Shrout and Fleiss, 1979, Zuo et al., 2010b), for details of the formulae, see Appendix B. The mode of *ICC* was calculated over voxel-wise values greater than zero using an 80-bin histogram spanning the [0–1] interval (Zuo et al., 2010b).

###### 2.1.1.2.2 ICN engagement repeatability at the individual subject level

Each dual-regressed individual IC was thresholded at *Z* > 3, and submitted to *ICN_Atlas* atlasing using SMITH10, BRAINMAP20 and BRAINMAP70 (all thresholded at Z > 3). Within- and between-session ICC were calculated for each metric on three different levels: (1) on the level of individual atlasing steps i.e. for every IC and individual atlas base map combination; (2) at the level of atlas base maps, i.e. collapsed across ICs; and (3) on a global level, i.e. collapsed across ICs and atlas base maps. This allowed us to capture and characterize the inflated variability caused by the different overlap of activations and atlas base maps at the level of individual atlasing steps, while on the other hand we could estimate the stability of metrics at the level of the atlas base maps and globally, by averaging this variability out. The normalization bounds ($max\;SPM_{t}-min\;SPM_{t}$) for the normalised activation metrics ***MA***_***N,i***_ and ***RA***_***N,i***_ were matched across input IC maps within any given session for each subject individually to ensure that the relative activation differences between ICs resulting from the same sessions are taken into account.

### 2.2 Demonstrations

In this section we describe two demonstrations of the application of *ICN_Atlas*: Firstly, we illustrate the problem of selecting a parsimonious subset of the proposed ICN engagement metrics for a given dataset; secondly, we show the results of two applications of *ICN_Atlas*: using a task-based dataset and in the field of epilepsy by quantifying ICN engagement evolution during epileptic seizures.

#### 2.2.1 ICN_Atlas engagement metrics factor analysis

*ICN_Atlas*' output for each input map consists of the value of each metric for each ICN; for example, for the full set of 11 ICN-specific metrics and using the SMITH10 atlas, this represents an output of 110 values per input map, in addition to the 4 global metrics. While a full set of metrics captures a greater amount of the variance than a subset, and therefore may be more useful for a complete analysis, we propose that a reduced subset may be more efficient for many applications and for the illustrative purposes of this report. We therefore sought to identify a subset of three ICN-specific metrics that satisfies the following criteria: 1) captures a sufficient amount of engagement across a given group or type of data; 2) has limited redundancy; 3) represents a summary of the level of engagement. To this effect, we performed a two-stage metrics set reduction procedure using the NYU rs-fMRI data; in each stage we performed a principal component analysis (PCA) and an exploratory factor analysis (EFA). In each variable reduction was performed through a *Varimax* rotation that identifies latent factors that represent linear combinations of existing variables that maximize the shared portion of the variance in the dataset. This was done first on the full set of 11 ICN-specific metrics, ICN_i_ Spatial Involvement (***I***_***i***_), ICN_i_ Relative Spatial Involvement (***IR***_***i***_), Spatial Overlap with ICN_i_ (***OL***_***i***_), Sørensen-Dice coefficient with ICN_i_ (***SQ***_***i***_), Jaccard index with ICN_i_ (***J***_***i***_), Mean ICN_i_ Activation (***MA***_***i***_), Normalised Mean ICN_i_ Activation (***MA***_***N,i***_), Relative Normalised Mean ICN_i_ Activation ($IR_{i}^{M}$), Normalised Relative ICN_i_ Activation (***RA***_***N,i***_), Normalised Mean ICN_i_ Activation Density ($I_{i}^{M}$), and Pearson's spatial correlation with ICN_i_ (***r***_***i***_) (see Appendix A for details on the calculated metrics) and repeated in a second stage on the metrics identified at the first stage based on high unicity and highest loadings on the two factors with the highest explained variance with the aim of identifying three metrics.

#### 2.2.2 Parametric variation of ICN engagement in a task-based fMRI experiment

To demonstrate *ICN_Atlas*' utility on task-based fMRI data, we selected an open access fMRI dataset from the NeuroVault database (http://neurovault.org/collections/659/) corresponding to the experiment described in Vagharchakian et al. (2012), which aimed to investigate how the language processing networks cope with fast visual and auditory sentence presentation rates. Briefly, neural activations for visual and auditory sentence presentation rates representing 20, 40, 60, 80 and 100 percent sentence durations with respect to a baseline of 5.9 syllables/s presentation rate were collected using fMRI and then analysed using GLM ANOVA with specific linear and non-linear contrasts and exclusive/inclusive contrast masking (for details see Vagharchakian et al., 2012). Three distinct response profiles were identified corresponding to (A): linear increase with stimulus duration, denoted as ‘Sensory profile’ characteristic for bilateral sensory cortices; (B): response collapse for the shortest presentation times, described by the authors as the ‘Post-bottleneck profile’, characteristic of activations in the bilateral superior and middle temporal gyri, left inferior frontal and precentral gyri, bilateral occipitotemporal cortex and visual word form area; and (C): maximum activation for intermediate rates, denoted as ‘Buffer profile’, characteristic of activity in the insulae, supplementary motor area bilaterally, anterior cingulate cortex, and left premotor cortex. The authors concluded that these response profiles are consistent with a processing bottleneck that is independent of the sensory limitation.

The data available from NeuroVault, consisted of simple group level compression rate vs. baseline contrast maps for each modality and presentation rate, each represented as Z-maps in MNI space according to the available metadata. Here we aimed to show the utility of *ICN_Atlas* for parametric data by (1) comparing whether atlasing results obtained with anatomical ROI-based atlasing using the CONN132 anatomical atlas for the available maps are consistent with the voxel-wise results published previously (for details see Vagharchakian et al., 2012), and by (2) evaluating whether the proposed ICN-level engagement metrics for the BRAINMAP20 atlas can enhance the interpretation of the study's results.

For the anatomical ROI comparison, we selected the following CONN132 atlas ROIs based on their correspondence with the activation clusters detailed in (Vagharchakian et al., 2012): the right and left insular cortices (ROIs CONN132-3 and CONN132-4), inferior frontal gyrus, pars triangularis left (CONN132-10), inferior frontal gyrus, pars opercularis left (CONN132-12), precentral gyrus, left (CONN132-14), superior temporal gyrus, anterior division right and left (CONN132-17 and CONN132-18), superior temporal gyrus, posterior division left (CONN132-20), lateral occipital cortex, inferior division, right and left (CONN132-45 and CONN132-46), frontal medial cortex (CONN132-49), supplementary motor area (SMA), left (CONN132-51), Heschl's gyrus right and left (CONN132-84 and CONN132-85). Atlasing was performed on unthresholded input maps, reflecting the lack of information in the NeuroVault metadata to support appropriate significance thresholding. Nevertheless, for visualization purposes an input map threshold of *Z* = 3 was also applied, see Fig. 10, below.

#### 2.2.3 ICN engagement evolution during epileptic seizures

To illustrate *ICN_Atlas*’ potential utility in relating BOLD changes to functional networks, we quantified ICN engagement during epileptic seizures in a patient with severe epilepsy (case #4 from Chaudhary et al., 2012).

##### 2.2.3.1 Case report

The patient underwent simultaneous scalp EEG and video recording and functional MRI scanning, during which 7 spontaneous seizures were captured (See Chaudhary et al. (2012) for details of the data acquisition and analysis). The seizures originating in the left temporal lobe were classified as typical, meaning that they are associated with clinical manifestations that are well characterised on clinical video EEG recordings. Ictal semiology was characterised by behavioural arrest, orofacial movements (oral automatisms), manual automatisms and loss of awareness. The seizure developed from stage II of sleep with indication that typical semiology did not fully develop given the constraints of the scanner environment. The patient appeared unaware/unconscious during the whole seizure. The ictal onset phase was characterised with a left temporal theta rhythm on EEG and no signs or symptoms. During the ictal established phase the abnormal activity on EEG became widespread. The patient exhibited orofacial movements (chewing and jaw clenching) and some jerks involving his head and hands. We considered that the patient did not only show such elementary motor signs, but probably aborted manual automatisms. During the late ictal phase left temporal slowing was evident on EEG and there was no semiology.

##### 2.2.3.2 fMRI analysis and ICN engagement quantification

As described in Chaudhary et al. (2012) the seizures captured during video-EEG-fMRI were partitioned into three ‘ictal phases’ based on close review of the EEG and video: ‘Early ictal’ (the start of the seizure), ‘Ictal established’ (characterised by rhythmic activity) and ‘Late ictal’.

The ictal phase-based analysis of the fMRI data is designed to reveal BOLD patterns associated with the specific electro-clinical manifestations characteristic of each phase The BOLD changes associated with each phase were mapped in the form of SPM [F]-maps at a significance threshold of p < 0.001 uncorrected for multiple comparisons with a cluster size threshold of 5 voxels, and co-registered with the patient's anatomical MRI scan and normalised to MNI space (Evans et al., 1993). *ICN_Atlas* was applied using the SMITH10 atlas to the fMRI map obtained for each ictal phase and ICN engagement was quantified for each ictal phase using the metrics ***I***_***i***_, ***RA***_***N,i***_ and ***MA***_***N,i***_ which were identified in the factor analysis described above (see sub-section 3.2.1 ‘*ICN_Atlas* involvement metrics factor analysis’ in Results).

## 3 Results

### 3.1 1.Validation

#### 3.1.1 Group-level independent components

**Fig. 2:**
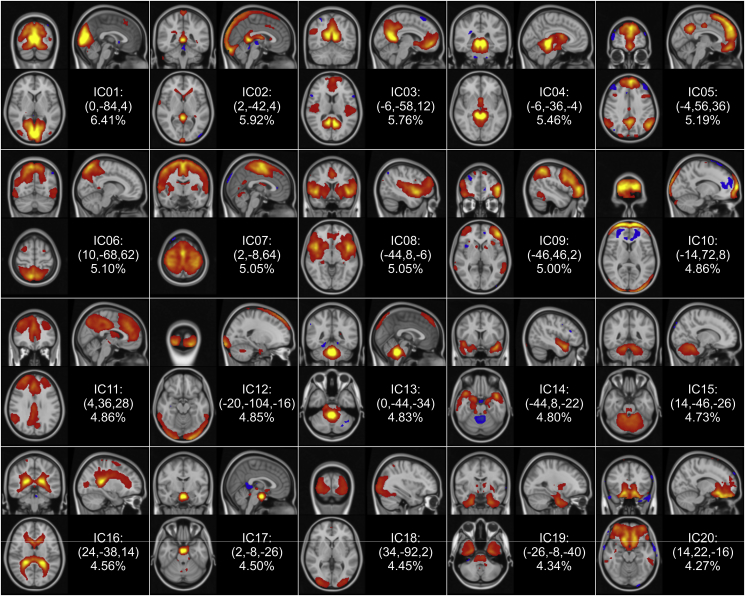
**Group-level components of the NYU-TRT data.** The 20 group-level independent components (ICs) obtained with temporal concatenation group ICA are shown in coronal, sagittal and axial planes going through the peak coordinates (shown in parentheses in MNI standard coordinates) according to radiological convention. The z-statistic maps are ordered according to the percentage of explained variance, and thresholded based on MELODIC's spatial mixture model at Z > 3.

The components obtained with temporal concatenation group ICA (Fig. 2) were consistent with previously published ICNs (Beckmann et al., 2005, Damoiseaux et al., 2006, Laird et al., 2011, Smith et al., 2009, Zuo et al., 2010b) and in particular showed strong similarities with those identified by Zuo et al. (2010b), although their ranking in terms of percentage of variance explained differed.

**Fig. 3:**
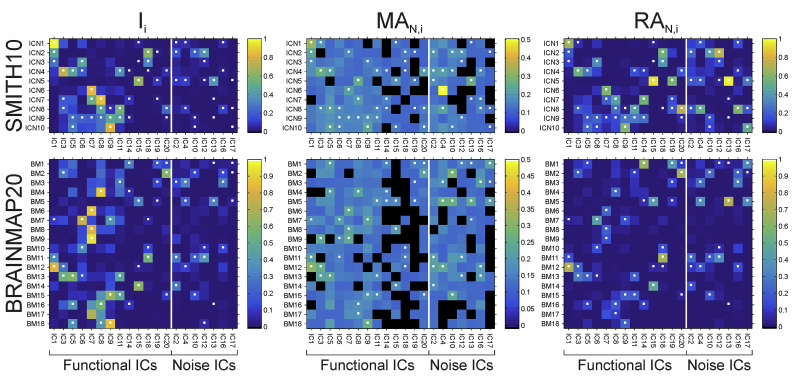
**Correspondence of the I**_**i**_**, MA**_**N,i**_**and RA**_**N,i**_**metrics for the SMITH10 (top row) and the BRAINMAP20 (bottom row) atlases**. Colour coding is according to engagement values for each IC (columns in each panel) and each atlas base map (rows in each panel), the three highest values for each IC (each column) are marked with white dots in each panel. White vertical bars separate functional ICs from noise ICs, black squares on **MA**_**N,i**_ panels show atlas base maps for given ICs where no voxel was active (i.e. I_i_ = 0), therefore **MA**_**N,i**_ is not calculated. The highest three **I**_**i**_ values for any given IC represent 61–99%, 48–95% and 21–80% of the total **I**_**i**_ for the given IC for SMITH10, BRAINMAP20 and BRAINMAP70, respectively (see the last rows of Supplementary Tables 2–10 for details, and Supplementary Fig. 1 for BRAINMAP70).

Thirteen ICs were identified that represent parts or combinations of functionally stereotypical ICNs (Beckmann et al., 2005, Damoiseaux et al., 2006, Laird et al., 2011, Smith et al., 2009) and therefore labelled *functional components*; these were IC1, IC3, IC5, IC6-IC9, IC11, IC14, IC15 and IC18-IC20. Based on their spatio-temporal characteristics, 7 components (IC2, IC4, IC10, IC12, IC13, IC16 and IC17) were labelled as *noise components* (e.g. typically scanner or physiological noise, head movement), which accounted for 34.98% of the variability present in the data. Concerning the functional ICs, IC1, IC6 and IC18 were found to relate to vision, IC6 also covering the superior parietal cortex and the premotor cortex, IC7 corresponded to the primary motor areas along with the association auditory cortices, and IC8 was related to the primary auditory cortices and the medial frontal, cingulate and paracingulate cortices, and the insula, and parts of the executive-control network. We observed that some ICNs were distributed across ICs, e.g. IC3 and IC5 represented the default mode network (DMN), IC9 the fronto-parietal networks corresponding to cognition and language bilaterally, IC11 the executive control and cingulate/paracingulate networks (complementing IC8). In addition, similarly to Zou et al.: cerebellar (IC18), temporal lobe, temporal pole, posterior insula and hippocampus (IC14 and IC19), brainstem (in IC19), and ventromedial prefrontal (IC20) components (Zuo et al., 2010b) were also identified.

#### 3.1.2 Group-level ICN engagement quantification

For all ICs and for each metric at least two of the top three engagement values pertained to the same atlas base maps for SMITH10 and BRAINMAP20 while at least one of the top three engagement values pertained to the same atlas base maps for BRAINMAP70 atlasing (Fig. 3 and Supplementary Fig. 1). Comparison of the matching atlas base maps in the top 3 values across engagement metrics and over all IC showed the following: the average numbers of matching atlas base maps were 2.05, 2.10, and 1.80 for the ***I***_***i***_ vs. ***MA***_***N,i***_; 2.75, 2.60, and 2.10 for the ***I***_***i***_ vs. ***RA***_***N,I*;**_ and 2.10, 2.40, and 1.80 for the ***MA***_***N,i***_ vs. ***RA***_***N,i***_ comparisons for SMITH10, BRAINMAP20 and BRAINMAP70, respectively. Taken together, the number of matches is significantly lower for the ***I***_***i***_ vs. ***RA***_***N,I***_ comparison for BRAINMAP70 compared against the other atlases, and also significantly lower for the ***MA***_***N,i***_ vs. ***RA***_***N,i***_ comparison for BRAINMAP70 vs. BRAIMAP20. Moreover for SMITH10 the (***I***_***i***_ vs. ***MA***_***N,i***_ and ***I***_***i***_ vs. ***RA***_***N,i***_) and the (***I***_***i***_ vs. ***MA***_***N,i***_ and ***MA***_***N,i***_ vs. ***RA***_***N,i***_) comparisons were significantly different (*p* < 0.0001), and for BRAINMAP20 the ***I***_***i***_ vs. ***MA***_***N,i***_ and ***I***_***i***_ vs. ***RA***_***N,i***_ comparison was significantly different (*p* < 0.05).

For the sake of brevity, in the following we summarise the findings by presenting only the highest **ICN**_**i**_ **Spatial Involvement (*I***_***i***_**)** metric value across all ICN for any given input map (group-level IC in this instance); the descriptions of the results for metrics ***MA***_***N,i***_ and ***RA***_***N,i***_ can be found in the Supplementary Materials.

**Fig. 4:**
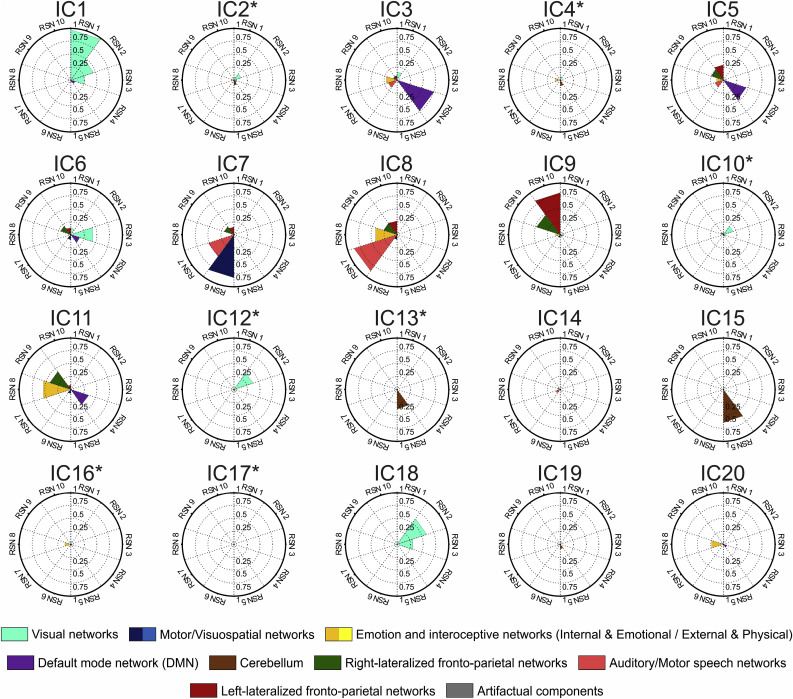
**ICN**_**i**_**Spatial Involvement (I**_**i**_**) of the NYU-TRT group-ICA components for the SMITH10 atlas.** The ICN_i_ involvement metrics are calculated based on the group-level TC-GICA results, ordered according to the percentage of explained variance. This ordering is similar to the one shown in Fig. 2. Noise ICs are marked with an asterisk.

**Fig. 5:**
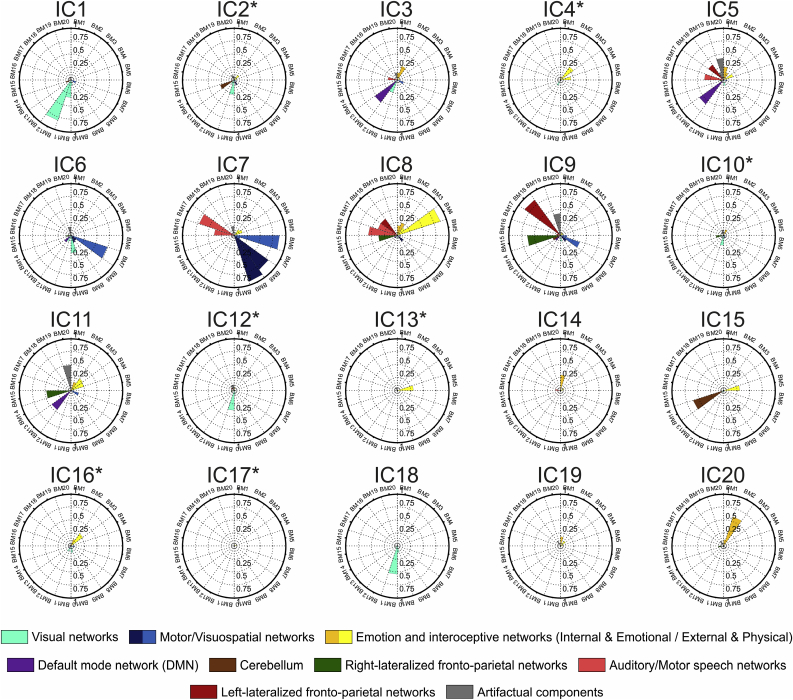
**ICN**_**i**_**Spatial Involvement (I**_**i**_**) of the NYU-TRT group-ICA components for the BRAINMAP20 atlas**. The ICN_i_ involvement metrics are calculated based on the group-level TC-GICA results, ordered according to the percentage of explained variance. This ordering is similar to the one shown in Fig. 2. Noise ICs are marked with an asterisk.

***I***_***i***_ values for SMITH10, BRAINMAP20 and BRAINMAP70 are plotted in Fig. 4, Fig. 5 and Supplementary Fig. 2 respectively (for numerical details see Supplementary Tables 2–4), showing the differing ICN representations in the three atlases (for details see and Table 1, Supplementary Table 1 and Supplementary Figs. 3, 4, and 5). The difference in the total extent of the ICN atlases was reflected in the global spatial engagement metric ***I***_***T***_ (Table 2) with generally lower involvement for BRAINMAP20 and BRAINMAP70 than for SMITH10, since BRAINMAP atlases cover greater part of the brain (and therefore have greater total ICN coverage, which is the denominator of ***I***_***T***_); moreover, as BRAINMAP70 can be considered as a subnetwork representation of BRAINMAP20 it is not surprising that their ***I***_***T***_ results were highly similar. For SMITH10 the temporal lobe and hippocampal components IC14 and IC19 showed low involvement (the highest involvement for IC14 was ***I***_***ICN7***_ = 0.09; for IC19 it was ***I***_***ICN5***_ = 0.09), compared to BRAINMAP20 (***I***_***BM20-1***_ = 0.30 for IC14 and ***I***_***BM20-1***_ = 0.18 for IC19) and BRAINMAP70 (***I***_***BM70-41***_ = 0.44 for IC14 and ***I***_***BM70-39***_ = 0.48 for IC19).

**Table 1:** Functional-anatomical and/or Intrinsic Connectivity Network correspondence of atlas base maps in the BRAINMAP20 and the SMITH10 atlases (for the BRAINMAP70 atlas, see Supplementary Table 1).

ICN #	Atlas base map (ICN) descriptions	
	BRAINMAP20 Atlas	SMITH10 Atlas
1	Limbic and medial-temporal areas	Visual – medial
2	Subgenual ACC and OFC	Visual – occipital pole
3	Bilateral BG and thalamus	Visual – lateral
4	Bilateral anterior insula/frontal opercula and the anterior aspect of the body of the cingulate gyrus	DMN
5	Midbrain	Cerebellum
6	Superior and middle frontal gyri	Sensorimotor
7	Middle frontal gyri and superior parietal lobules	Auditory
8	Ventral precentral gyri, central sulci, postcentral gyri, superior and inferior cerebellum	Executive control
9	Superior parietal lobule	Frontoparietal (perception-somesthesis-pain)
10	Middle and inferior temporal gyri	Frontoparietal (cognition-language)
11	Lateral posterior occipital cortex	
12	Medial posterior occipital cortex	
13	Medial prefrontal and posterior cingulate/precuneus areas, DMN	
14	Cerebellum	
15	Right-lateralized fronto-parietal regions	
16	Transverse temporal gyri	
17	Dorsal precentral gyri, central sulci, postcentral gyri, superior and inferior cerebellum	
18	Left-lateralized fronto-parietal regions	
19	Artefactual component	
20	Artefactual component

**Table 2:** Global ICN Spatial Involvement (I_T_) for each NYU-TRT group ICA IC.

		Global ICN Spatial Involvement: *I*_*T*_		
		SMITH10	BRAINMAP20	BRAINMAP70
Functional ICs	IC01	0.15	0.08	0.08
	IC03	0.15	0.10	0.10
	IC05	0.15	0.15	0.15
	IC06	0.13	0.10	0.09
	IC07	0.21	0.17	0.17
	IC08	0.23	0.16	0.15
	IC09	0.19	0.15	0.14
	IC11	0.19	0.12	0.12
	IC14	0.02	0.04	0.03
	IC15	0.06	0.05	0.04
	IC18	0.07	0.04	0.03
	IC19	0.01	0.03	0.03
	IC20	0.05	0.07	0.07
Noise ICs	IC02	0.04	0.06	0.06
	IC04	0.03	0.04	0.04
	IC10	0.03	0.05	0.05
	IC12	0.03	0.05	0.06
	IC13	0.03	0.03	0.03
	IC16	0.03	0.05	0.08
	IC17	0.00	0.01	0.01

Overall, the ICN engagement results of the group ICA matched well their functional role; for SMITH10, for visual components IC1, IC6, and IC18 the highest involvement values were ***I***_***ICN1***_ = 0.97, ***I***_***ICN3***_ = 0.45 and ***I***_***ICN2***_ = 0.61, respectively; for IC3 and IC5 (DMN), ***I***_***ICN4***_ = 0.75 and ***I***_***ICN4***_ = 0.47, respectively; for the sensory-motor and auditory component IC7, ***I***_***ICN6***_: = 0.84; for the auditory and executive control component IC8, ***I***_***ICN7***_ = 0.87; for the bilateral fronto-parietal component IC9, ***I***_***ICN10***_ = 0.82; for cerebellar component IC15, ***I***_***ICN5***_ = 0.64; for executive control component IC11, ***I***_***ICN8***_ = 0.56; and for prefrontal component IC20, ***I***_***ICN8***_ = 0.24 (see Fig. 4 and Supplementary Table 2 for details).

The engagement results for BRAINMAP20 showed a similar pattern, for the visual components IC1, IC6 and IC18 the highest involvement values were ***I***_***BM20-12***_ = 0.83, ***I***_***BM20-7***_ = 0.75 and ***I***_***BM20-11***_ = 0.55 respectively; for IC3 and IC5 (DMN), ***I***_***BM20-13***_ = 0.54 and ***I***_***BM20-13***_ = 0.58, respectively; for the sensory-motor and auditory component IC7, ***I***_***BM20-9***_ = 0.93 (note, that high involvement were found also for ***I***_***BM20-6***_ = 0.88 and ***I***_***BM20-8***_ = 0.82); for the auditory and executive control component IC8, ***I***_***BM20-4***_ = 0.88; for the bilateral fronto-parietal component IC9, ***I***_***BM20-18***_ = 0.85; for cerebellar component IC15, ***I***_***BM20-14***_ = 0.62; for executive control component IC11, ***I***_***BM20-20***_ = 0.49 (with minimally different ***I***_***BM20-15***_ = 0.48); and for prefrontal component IC20, ***I***_***BM20-2***_ = 0.60 (see Fig. 5 and Supplementary Table 3 for details).

The engagement results for BRAINMAP70 showed a pattern consistent with subnetwork fractionation, when considered against those for BRAINMAP20, in having similarly high involvement values in some of atlas base maps for most ICs (e.g. for visual component IC1 the highest involvement values were ***I***_***BM70-2***_ = 0.98 and ***I***_***BM70-1***_ = 0.97; for visual component IC6 the highest involvement values were ***I***_***BM70-7***_ = 0.85 and ***I***_***BM70-9***_ = 0.80) while for visual component IC18 there was a single highest involvement value of ***I***_***BM70-3***_ = 0.61. For the default mode network, components IC3 and IC5 the highest involvement values were ***I***_***BM70-61***_ = 0.82 and ***I***_***BM70-38***_ = 0.89, respectively; for the sensory-motor and auditory component IC7, ***I***_***BM70-35***_ = 0.98; for the auditory and executive control component IC8, ***I***_***BM70-52***_ = 0.98; for the bilateral fronto-parietal component IC9, ***I***_***BM70-12***_ = 0.96, (with high involvement for ***I***_***BM70-49***_ = 0.89 and ***I***_***BM70-51***_ = 0.86); for cerebellar component IC15, ***I***_***BM70-60***_ = 0.83; for executive control component IC11, ***I***_***BM70-17***_ = 0.74; and for prefrontal component IC20, ***I***_***BM70-20***_ = 0.79 (see Supplementary Fig. 2 and Supplementary Table 4 for details).

The spatial involvement values for the ‘noise’ ICs IC2, IC4, IC10, IC16 and IC17 were all <0.3 for SMITH10, with noise component IC12 and IC13 having the highest values: ***I***_***ICN2***_ = 0.39 and ***I***_***ICN5***_ = 0.38, respectively. Similarly, for BRAINMAP20 the involvement values for noise ICs IC2, IC10, IC16 and IC17 were <0.30, with IC4, IC12, and IC13 showing ***I***_***BM20-3***_ = 0.31, ***I***_***BM20-11***_ = 0.39, and ***I***_***BM20-5***_ = 0.32, respectively. Consistent with the sub-network representation in BRAIMAP70, the ‘noise’ ICs had wider range of maximum ***I***_***i***_, ranging from ***I***_***BM70-56***_ = 0.17 for IC17 to ***I***_***BM70-58***_ = 0.78 for IC16 (for details see Supplementary Fig. 2 and Supplementary Table 4).

#### 3.1.3 Test-retest repeatability

##### 3.1.3.1 IC voxel-wise group level repeatability

Across all ICs the modes of the within- and between-session intra-class correlation coefficients <*ICC*_*W*_> and <*ICC*_*B*_> were in the range of 0.18–0.65. Of the functional ICs, IC9 (bilateral fronto-parietal network), IC3 and IC5 (parts of the DMN), and IC1 (vision) exhibited the highest repeatability, with (<*ICC*_*W*_>, <*ICC*_*B*_>) = (0.63, 0.65), (0.64, 0.61), (0.61, 0.60), and (0.61, 0.59) respectively. Most other functional ICs (IC6, IC7, IC8, IC11, and IC14) had <*ICC*_*W*_> and <*ICC*_*B*_> values in the ranges ([0.44–0.58], [0.43–0.53]) while IC19 (temporal lobe) and IC20 (cerebellar) had lower repeatability ([0.21–0.44], [0.20–0.41]), similar to most of the noise ICs (IC4, IC10, IC12, IC13, IC16 and IC17). Note the high repeatability for noise component IC2 (venous sinuses) with <*ICC*_*W*_> = <*ICC*_*B*_> = 0.65.

##### 3.1.3.2 ICN engagement repeatability at the individual subject level

**Fig. 6:**
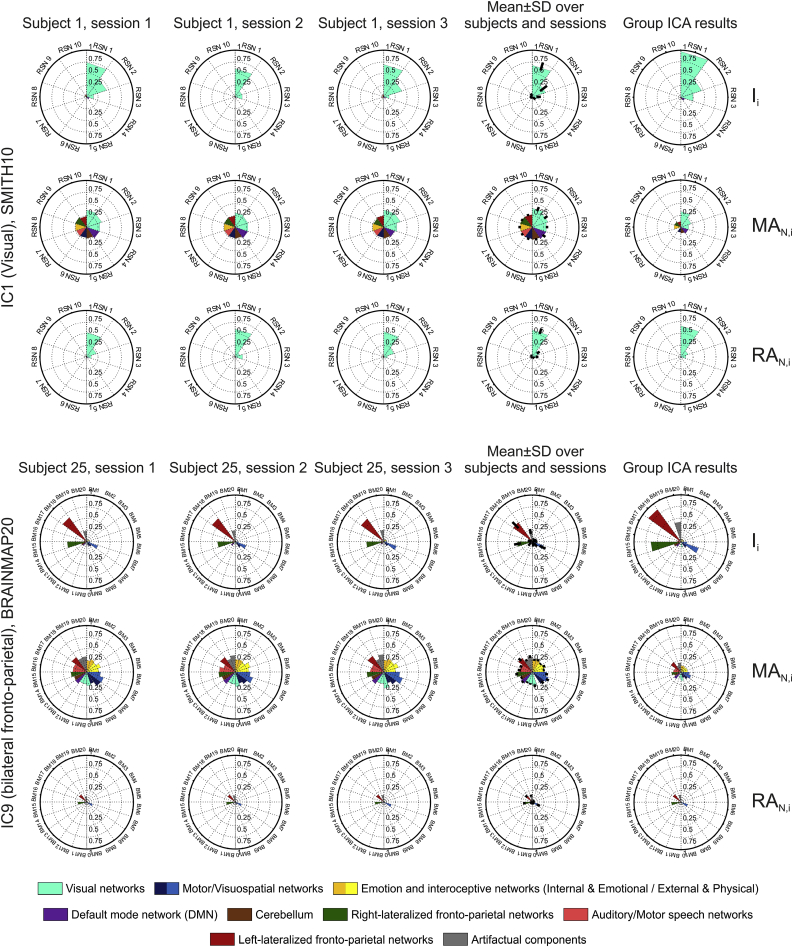
**Representative examples of atlasing on dual-regressed individual data, compared to group results**. Top panel: IC1 (visual IC) for subject #1 atlased using the SMITH10 atlas; Bottom panel: IC9 (bilateral fronto-parietal IC) for subject #25 atlased using the BRAINMAP20 atlas. Engagement metrics: **I**_**i**_, **MA**_**N,i**_ and **RA**_**N,i**_. The left three columns show the result for each of the 3 scanning sessions; the fourth column shows engagement metric mean ± SD over all subjects and across the 3 sessions; the fifth column shows the Group ICA results.

The distribution of engagement metric values for individual dual-regressed single-session ICA maps across base maps were similar to those obtained by atlasing of the group ICA maps; for a visual comparison see Fig. 6.

**Fig. 7:**
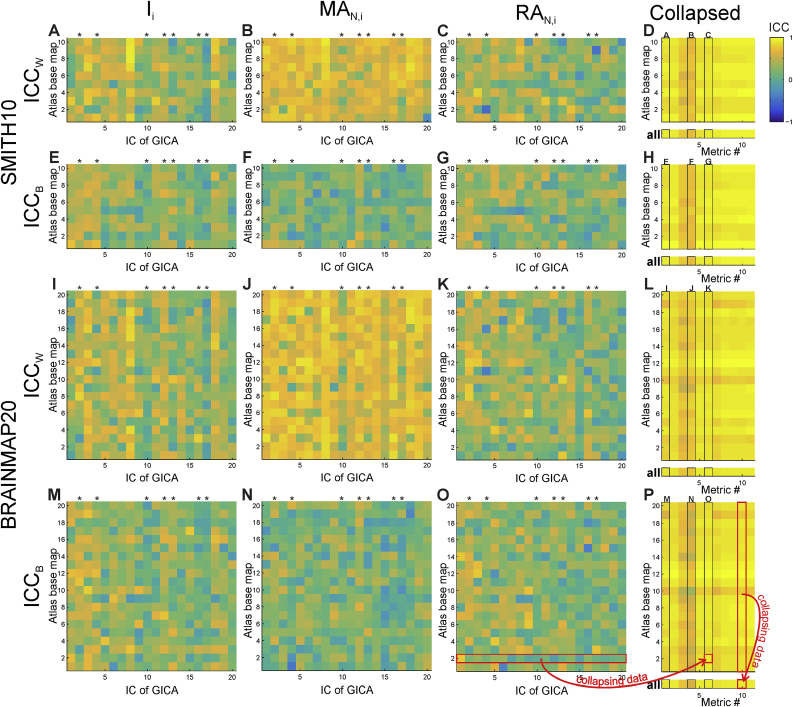
**Test-retest engagement reliability: within- and between-session intra-class correlation coefficient (ICC) scores**. Parts A-D, I–L: within-session; parts E-H, M–P: between-session ICC scores using the SMITH10 and BRAINMAP20 atlases, respectively. ICC scores are shown for every IC and atlas base map combination for the ***I***_***i***_ (A, E, I, M), ***MA***_***N,i***_ (B, F, J, N), and ***RA***_***N,i***_ (C, G, K, O) metrics. Data collapsed either across ICs or across ICs and atlas base maps are shown in panels D, H, L, P in the top and bottom (‘all’) subplots, respectively. The schematic representation of the data collapsing strategy (explained in detail in Methods) is shown in panels O and P: red source and target boxes and red arrows; the panel labels corresponding to the collapsed metrics are marked in the respective subplots (e.g. A, B, C in panel D). Noise ICs are marked by asterisks on all panels. ICC_W_: within-session ICC, ICC_B_: between-session ICC. Metric # represents the output metrics as follows: (1) ***I***_***i***_, (2) ***IR***_***i***_, (3) ***MA***_***i***_, (4) ***MA***_***N,i***_, (5) ***IR***_***i***_^***M***^, (6) ***RA***_***N,i***_, (7) ***I***_***i***_^***M***^, (8) ***OL***_***i***_, (9) ***SQ***, (10) ***J***_***i***_, and (11) ***r***_***i***_. See Supplementary Fig. 8 for BRAINMAP70, and Supplementary Tables 11–34 for numerical values.

**Fig. 8:**
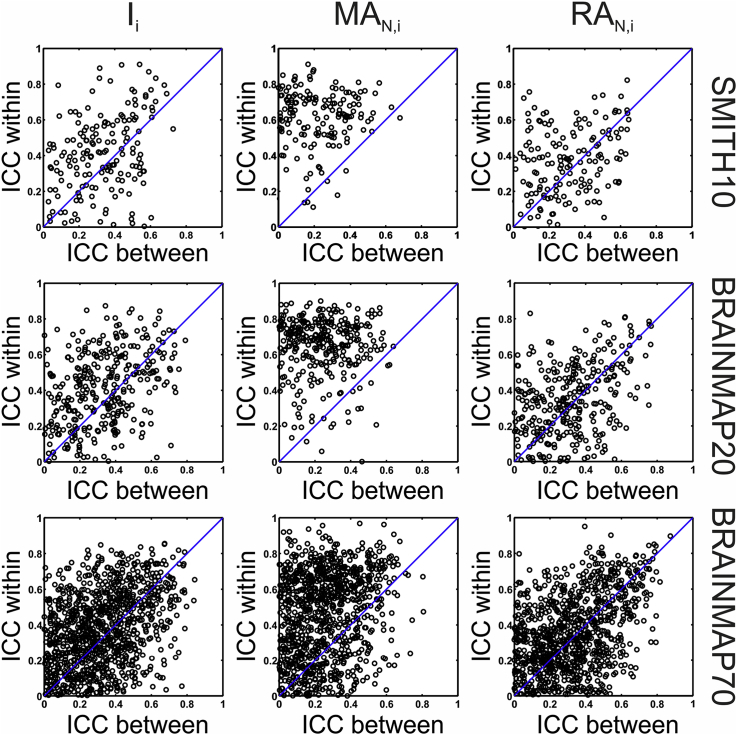
Test-retest engagement reliability: Comparison of within- and between-session ICC scores for the SMITH10, BRAINMAP20, and BRAINMAP70 atlases. Each data points represents an IC atlased with one of the atlas base maps. The majority of within-session ICC scores are higher than the between-session ICC scores. Only positive ICCs are shown.

At the level of atlasing for every IC and individual base map combination, within- and between-session ICN engagement repeatability varied considerably; nevertheless median values indicated fair-to-moderate agreement (see Table 3, Fig. 7, and Supplementary Fig. 8 for details). As expected, within-session ICC tended to be higher than the between-session (Fig. 8). In summary, median test-retest repeatability (<*ICC*_*W*_>; <*ICC*_*B*_>) for the SMITH10 atlas were (0.37; 0.28) for ***I***_***i***_, and (0.63; 0.16) and (0.30; 0.23) for ***MA***_***N,i***_ and ***RA***_***N,i***_ respectively. The results were very similar for the BRAINMAP20 atlas, with test-retest ***I***_***i***_ repeatability of (0.36; 0.28), and (0.66; 0.18) and (0.28; 0.25) for ***MA***_***N,i***_ and ***RA***_***N,i***_ respectively; for the BRAINMAP70 atlas, with test-retest ***I***_***i***_ repeatability of (0.30; 0.25), and (0.39; 0.16) and (0.24; 0.22) for ***MA***_***N,i***_ and ***RA***_***N,i***_ respectively (see Supplementary Tables 11–28 for details). We note a small number of negative ICC values, which were found to reflect minimal or null overlap between the ICs and the atlas base maps, as shown in Supplementary Fig. 9.

**Table 3:** **Test-retest reliability results measured by ICC.** Median and range of within- and between-session ICC scores for I_i_, MA_N,i_, and RA_N,i_ for the SMITH10 and BRAINMAP20 atlases. IC × base map: separate ICC calculations for each IC and atlas base map combination, c.f. Fig. 7 panels A–C and E-F; Base map: ICC calculated for each base map separately, i.e. collapsed across ICs; Global: ICC calculated for data collapsed across ICs and base maps.

		ICC, median and [range]					
		Within-session			Between-session		
		IC × base map	Base map	Global	IC × base map	Base map	Global
SMITH10	***I***_***i***_	0.37 [−0.22–0.91]	0.90 [0.82–0.97]	0.92	0.28 [−0.22*–*0.73]	0.90 [0.81–0.97]	0.91
	***MA***_***N,i***_	0.63 [−0.29–0.91]	0.80 [0.65–0.84]	0.79	0.16 [−0.31–0.68]	0.60 [0.48–0.69]	0.61
	***RA***_***N,i***_	0.30 [−0.48–0.82]	0.90 [0.85–0.96]	0.91	0.23 [−0.28–0.65]	0.92 [0.86–0.96]	0.92
BRAINMAP20	***I***_***i***_	0.36 [−0.18–0.87]	0.89 [0.71–0.95]	0.89	0.28 [−0.25–0.79]	0.87 [0.67–0.96]	0.88
	***MA***_***N,i***_	0.66 [−0.08–0.9]	0.78 [0.55–0.88]	0.78	0.18 [−0.41–0.64]	0.60 [0.32–0.78]	0.62
	***RA***_***N,i***_	0.28 [−0.39–0.83]	0.90 [0.79–0.97]	0.91	0.25 [−0.38–0.77]	0.91 [0.79–0.98]	0.93
BRAINMAP70	***I***_***i***_	0.30 [−0.3–0.89]	0.84 [0.6–0.96]	0.85	0.25 [−0.3–0.84]	0.83 [0.51–0.97]	0.84
	***MA***_***N,i***_	0.39 [−0.39–0.97]	0.46 [0.21–0.74]	0.48	0.16 [−0.45–0.81]	0.46 [0.23–0.67]	0.49
	***RA***_***N,i***_	0.24 [−0.45–0.95]	0.83 [0.5–0.96]	0.87	0.22 [−0.38–0.87]	0.84 [0.51–0.97]	0.89

At the base map level, i.e. collapsed across ICs (hence eliminating most of the IC-related variability), test-retest ICN engagement repeatability ranged between moderate and very strong, with median (<*ICC*_*W*_>; <*ICC*_*B*_>) = (0.90; 0.90) for ***I***_***i***_, while for ***MA***_***N,i***_ and ***RA***_***N,i***_ these were (0.80; 0.60) and (0.90; 0.92), respectively for the SMITH10 atlas. The results were very similar for the BRAINMAP20 atlas, with test-retest atlas base map repeatability for ***I***_***i***_ of (0.89; 0.87), and (0.78; 0.60) and (0.90; 0.91) for ***MA***_***N,i***_ and ***RA***_***N,i***_ respectively; and for the BRAINMAP70 atlas, with test-retest atlas base map ***I***_***i***_ repeatability of (0.84; 0.83), and (0.46; 0.46) and (0.83; 0.84) for ***MA***_***N,i***_ and ***RA***_***N,i***_ respectively (see Table 3, Fig. 7, and Supplementary Materials for details).

Finally, ICN engagement metric reliability calculated over all subjects, atlas base maps, and ICs, showed strong to very strong agreement, with (<*ICC*_*W*_>; <*ICC*_*B*_>) values of: (0.92; 0.91) for ***I***_***i***_, (0.79; 0.61) for ***MA***_***N,i***_ and (0.91; 0.92) for ***RA***_***N,i***_ for SMITH10; for BRAINMAP20, the corresponding values were (0.89; 0.88), (0.78; 0.62) and (0.91; 0.93); and for BRAINMAP70, the corresponding values were (0.85; 0.84), (0.48; 0.49) and (0.87; 0.89) (see Table 3, Fig. 7, and Supplementary Materials for details).

### 3.2 Demonstrations

#### 3.2.1 ICN_Atlas involvement metrics factor analysis

The five metrics identified at the first stage of the factor analysis using the NYU rs-fMRI data were: two spatial involvement metrics: ***I***_***i***_ and ***IR***_***i***_, and three activation strength-weighted metrics: ***MA***_***i***_, ***MA***_***N,i***_, and ***RA***_***N,i***_. The second-stage factor analysis, performed to limit the number of metrics to three, revealed that ***I***_***i***_ and ***RA***_***N,i***_, contributed most to the two latent factors, which explained 68% of the variance, and that ***MA***_***N,i***_ had a high degree of uniqueness.

#### 3.2.2 Parametric variation of ICN engagement in a task-based fMRI experiment

**Fig. 9:**
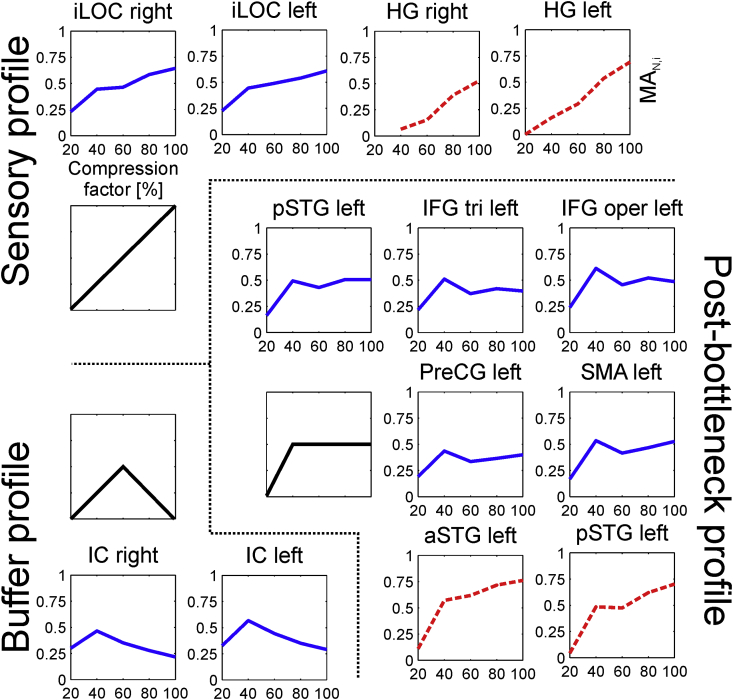
ICN_i_ Spatial Involvement (I_i_) of the auditory and visual parametric modulation fMRI data set calculated using the anatomy-based CONN132 atlas. The three response profiles detailed in Vagharchakian et al. (2012) can be identified in ROIs matching those published previously. The response profiles are represented with solid black lines, the visual stimulus modality is represented with solid blue lines, and the auditory stimulus modality is represented with dashed red lines. 20%, 40%, 60%, 80% and 100% compression factors represent 1333, 645, 429, 323, and 257 words per minute, i.e. 46 ms, 93 ms, 140 ms, 186 ms and 233 ms mean word durations, respectively.

Engagement as estimated by ***MA***_***N,i***_ was found to match the ‘Sensory profile’ (linear increase with stimulus duration) for the visual stimulus modality in the left and right inferior lateral occipital cortex ROIs (CONN132-45 and CONN132-46) and for the auditory stimulus modality in the left and right Heschl's gyri (CONN132-84 and CONN132-85). In addition, the ***MA***_***N,i***_ values for the auditory presentations followed the so-called ‘post-bottleneck profile’ (sudden collapse of activation for the shortest stimulus duration) in the left superior temporal gyrus (CONN132-18 and CONN132-20); for visual stimulation the similar effect was observed for the left posterior superior temporal gyrus (CONN132-20), the left inferior frontal gyrus (CONN132-10 and CONN132-12), left precentral gyrus (CONN132-14), left SMA (CONN132-51), while a pattern of ICN engagement resembling the ‘buffer profile’ (highest activation for intermediate durations) was observed in the insular cortices (CONN132-3 and CONN132-4) for visual stimulation (Fig. 9).

**Fig. 10:**
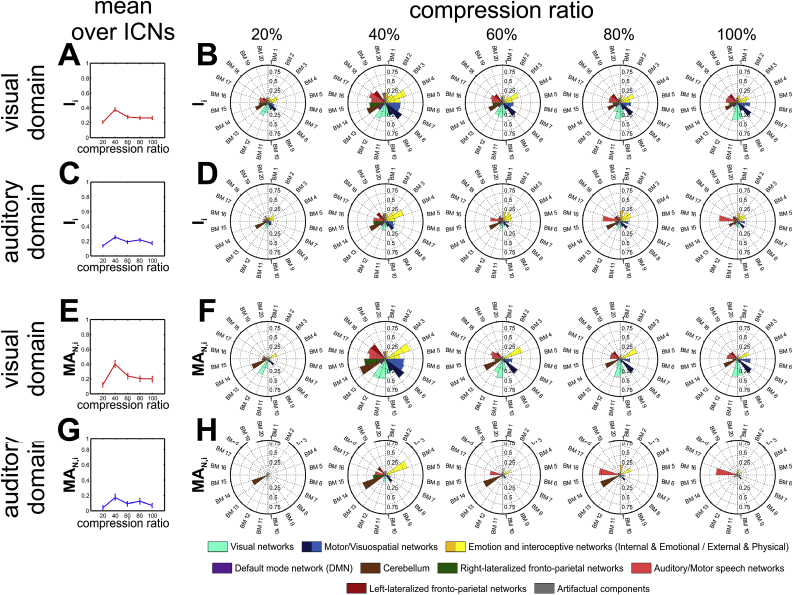
Normalised Mean ICN_i_ Activation (MA_N,i_) and ICN_i_ Spatial Involvement (I_i_) of the auditory and visual parametric modulation fMRI data set calculated using the anatomy-based CONN132 atlas. A, C, E, G: mean engagement values over atlas base maps, B, D, F, H: polar representation of engagement metrics. A–D: Normalised Mean ICNi Activation (MA_N,i_) values obtained without thresholding the input activation maps, E–H: ICN_i_ Spatial Involvement (I_i_) values obtained with Z > 3 thresholding the input activation maps. 20%, 40%, 60%, 80% and 100% compression factors represent 1333, 645, 429, 323, and 257 words per minute, i.e. 46 ms, 93 ms, 140 ms, 186 ms and 233 ms mean word durations, respectively.

ICN engagement as estimated by ***MA***_***N,i***_ and ***I***_***i***_ showed differential involvement of ICNs depending on stimulus modality and stimulus duration (compression ratio). Stimulus modality was clearly visible in the differential engagement of visual and auditory/language ICNs. Regarding stimulus duration, the individual ***MA***_***N,i***_ and ***I***_***i***_ values were found to be stable or increase slightly for easily understood auditory stimuli (60–100% compression ratio), with peak values for the difficult but intelligible (40% compression ratio) and a collapse for the unintelligible (20% compression ratio) stimuli, regardless of stimulus modality (Fig. 10). This behavior resembled the phase profile suggested for integrative regions (Vagharchakian et al., 2012). These parametric changes depending on stimulus duration represented a network-wide behavior, i.e. they were not exclusively driven by a single or a small group of ICNs.

#### 3.2.3 ICN involvement evolution during epileptic seizures

**Fig. 11:**
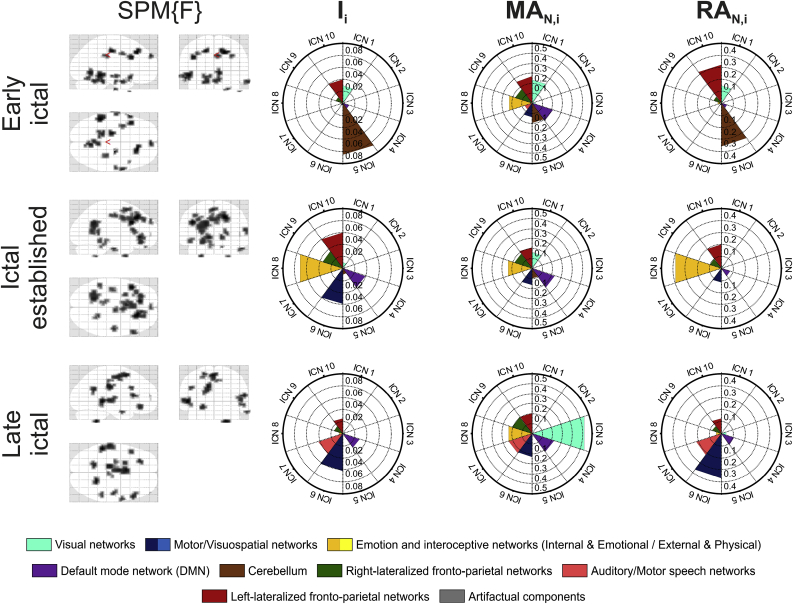
ICN involvement evolution during epileptic seizures as quantified by three metrics across the ictal phases using the SMITH10 atlas. The BOLD changes associated with epileptic activations in the early ictal (top row), ictal established (middle row), and late ictal (bottom row) phases are shown in statistical parametric maps (leftmost column, SPM {F} maps at p < 0.001 significance threshold, uncorrected for multiple comparisons with a cluster size threshold of 5 voxels). Polar plots of I_i_ (second column), MA_N,i_ (third column), and RA_N,i_ (rightmost column) are based on atlasing of the respective SPMs.

As illustrated in Fig. 11, ICN engagement as assessed using the SMITH10 atlas fluctuated across ictal phases. Total spatial involvement (***I***_***T***_) was generally low, with a value of 0.017 in the ictal onset phase, doubling to 0.035 in the ictal established phase and decreasing to 0.020 in the late ictal phase.

With respect to individual ICNs, we note a high degree of involvement in ICN4 (DMN), ICN5 (cerebellum), ICN8 (executive control) and in ICN9 and ICN10 (fronto-parietal) during the Early Ictal phase. Significant involvement intensity changes were seen in ICN6 (sensorimotor network) and ICN8 (executive control) during the Ictal Established phase. The Late Ictal phase was characterised by significantly reduced spatial engagement globally. DMN involvement intensity is maintained throughout the seizures.

We now focus on three ICN, namely the DMN (ICN4), sensorimotor network (ICN6) and executive network (ICN8), in a top down/semiological interpretation perspective on ICN engagement.

The DMN shows a pattern of increasing engagement relative to other ICNs across phases. It ranks 5th in terms of ICN spatial involvement at the Early Ictal phase and shows a pattern of increase and subsequent decrease in the Ictal Established and Late Ictal phases, respectively. Its activation level (***MA***_***N,ICN4***_) is roughly constant throughout the phases, but goes from being negligible in intensity relative to globally-observed activation (***RA***_***N,ICN4***_) in the Early Ictal phase to approximately 4th in importance in the subsequent phases.

The sensorimotor network (ICN6), is the second most spatially involved network (after the cerebellum (ICN5)) at the early ictal phase and its activation level grows consistently across phases as does its intensity relative to the whole-brain activation level, becoming the most prominent in the late ictal phase.

For the executive network (ICN8) the level of spatial involvement is relatively low in the Early Ictal phase while its activation level (***MA***_***N,ICN8***_) is roughly constant throughout the phases similarly to the DMN; however in contrast to the DMN, the executive network becomes very prominent relative to globally-observed activation in the ictal established phase (***RA***_***N,ICN8***_).

## 4 Discussion

The main objective of the proposed *ICN_Atlas* methodology is to provide a quantitative and objective framework to characterize fMRI activation (and deactivation) maps in terms of ‘functional engagement’ in contrast to methods based on anatomically defined coverage and in particular those based purely on visual description of fMRI map anatomical coverage. To this effect it seems appropriate to base the quantification on atlases derived from maps obtained ‘functionally’, namely sets of intrinsic connectivity (or resting state) networks (ICNs) derived based on fMRI data.

We have addressed the issue of validity in terms of repeatability and reproducibility, by applying a commonly used methodology to extract independent components (putative ICNs) from a publically available longitudinally-acquired resting-state fMRI dataset (NYU-TRT dataset). The resulting ICNs were then subjected to the proposed atlasing scheme using three ICN base maps (SMITH10, BRAINMAP20 and BRAINMAP70), thereby providing an assessment of *ICN_Atlas*’ robustness in terms of its ability to identify functionally stereotypical ICNs across scanning sessions. The results of this analysis showed that repeatability as measured by the intra-class correlation coefficient is dependent both on the atlased activation maps and the atlas base map used for atlasing. Repeatability for the atlas base maps showed moderate to very strong agreement depending on the metric considered. The overall repeatability calculated by collapsing data across subjects, IC maps, and atlas base maps, showed strong to very strong within- and between-session agreement. The outcome of the repeatability analysis is on par with previous repeatability estimates obtained on the same data with other approaches (Shehzad et al., 2009, Zuo et al., 2010a, Zuo et al., 2010b).

To demonstrate the potential utility of *ICN_Atlas* we applied it to two datasets: firstly, an independently obtained, open access task-based fMRI dataset (Vagharchakian et al., 2012), selected to show how our tool can capture variations due to parametric modulations; secondly, we also wanted to demonstrate *ICN_Atlas*’ potential utility in clinical research by illustrating its application to fMRI data in one of own areas of expertise, namely fMRI of human epileptic activity.

Conceiving *ICN_Atlas* as a descriptive tool implies data reduction: from a whole-brain functional map to a set of numbers of a size that that facilitate comprehension and communication. We therefore considered the issue of the atlas' output, in particular the quasi-infinite number of conceivable engagement metrics (to be calculated for every ICN). Starting with a wide-ranging set of ICN engagement metrics devised based on general considerations of fMRI maps' spatial and activation intensity, we performed a factor analysis as a rational basis to select a reduced set of metrics; we chose three as a desirable number of metrics to estimate and report on, keeping in mind that this number is multiplied by the number of ICNs in the base atlas, which ranges from 10 to 70 in the three used in this work, as the tool's total output (plus four global metrics). We believe that a limited set of metrics, i.e. 30 for the SMITH10 atlas, per fMRI map is manageable at this very early stage of the tool's application. Future similar analyses on other datasets may reveal a pattern which helps us identify an optimal set of metrics; such a consensus would be beneficial as it would help standardising the methodology.

### 4.1 Choice of atlases

The atlases we chose for this validation study and initial demonstrations represent two very different approaches for describing intrinsic connectivity networks: The SMITH10 atlas is based on resting-state fMRI data, while the BRAINMAP20 and BRAINMAP70 are based on ICA decomposition of task-based fMRI data (Laird et al., 2011, Ray et al., 2013, Smith et al., 2009). It has previously been shown that the SMITH10 and BRAINMAP20 atlases yield highly similar results for ten well-matched ICNs (Smith et al., 2009), but more recently Laird et al. showed that there are 8 additional ICNs that can be reliably derived from task-based data (Laird et al., 2011). The greater number of functional components in BRAINMAP20 results in greater brain coverage, a fact reflected accurately in the global engagement metric (***I***_***T***_) values we obtained (Table 2). Maps obtained with increased ICA dimensionality tend to show the expected subnetwork fractionation with respect to the networks seen at lower dimensionality (Ray et al., 2013, Smith et al., 2009), without significantly affecting global ICN engagement. The cognitive domain based colouring of *ICN_Atlas* output further supports the similarities between the base maps (see Fig. 4, Fig. 5, and Supplementary Figs. 2–4).

It has previously been shown that ICNs obtained with low model order ICA (*d* = 10 or 20) represent large-scale functional networks, while higher model orders lead to subnetwork fractionation (Abou-Elseoud et al., 2010, Ray et al., 2013, Smith et al., 2009). While the SMITH10 and BRAINMAP20 atlases represent well-documented large-scale functional network obtained for model order *d* = 20, what model order would be the best suited for ICN subnetwork-based description of functional activations remains an open question. It has been shown that ICA model order 70 can lead to robustly detectable components (Kiviniemi et al., 2009); furthermore, model orders (*d*) of 60–80 have been shown to: (1) sufficiently separate signal sources; (2) be repeatable; (3) not over-fit the data; and (4) show significant changes in volume and mean Z-score for the evaluation of ICNs (Abou-Elseoud et al., 2010). This was further corroborated by hierarchical clustering analysis on BrainMap metadata matrices, i.e. matrices that were designed to quantify the relationship between ICs and behavioural domains or paradigms, where the quality of hierarchical clustering was found to be highest for ICA model orders d = 20 and d = 70, leading to a more clear-cut correspondence between functional properties and ICNs (Ray et al., 2013). Based on these observations, the BRAINMAP70 atlas (based on ICA of model order 70) seems to provide an appropriate description of ICNs on a subnetwork level.

Our comparisons of the two lower-dimensionality atlas base maps, SMITH10 and BRAINMAP20, have shown contrasting quantitative functional map descriptions (see Supplementary Fig. 3), for example in relation to the temporal lobes, where there is a specific limbic and medial-temporal map (BM20-1) in the BRAINMAP20 atlas which has a minimal overlap with the auditory (ICN7) base map of the SMITH10 atlas. Furthermore, we note that the SMITH10 ICNs do not cover the hippocampi, which may limit this specific base atlas's applicability to data from patients with temporal lobe epilepsy (TLE) for example. It is noteworthy that the anatomical coverage of the BRAINMAP70 atlas is similar to that of BRAINMAP20, as reflected by global engagement metric ***I***_***T***_.

Given the choice of base atlases presented here, all derived from data collected in predominantly healthy adults, one could argue that the utility of *ICN_Atlas* is limited to experimental data obtained on neurologically ‘typical’ adults. Indeed, the optimal atlas depends on the population investigated (Mandal et al., 2012), and no pre-calculated atlas can be considered perfect for all purposes. Still, the Talairach and Tournoux atlas (Talairach and Tournoux, 1988) is based on a single 60 years old female, and the AAL atlas (Tzourio-Mazoyer et al., 2002)is based on the Colin-27 brain template (Holmes et al., 1998), yet the former is still widely used for neurosurgical planning in non-neurotypical patient, and the latter is widely used in fMRI ROI analyses for both neurotypical and –atypical subjects, and even a high proportion of the CONN132 atlas ROIs are based on it. Moreover, there is no widely accepted standard spatial template space for children, and therefore pediatric rs-fMRI analyses can be performed either in the MNI template space (Thomason et al., 2011), or age and study specific templates can be created (Muetzel et al., 2016). Therefore the choice of atlas can be seen as one between generalizability and universality, vs specificity.

Nevertheless, the *ICN_Atlas* framework is designed to accommodate multiple atlases, including any derived from pathological data. For example one could envisage the use of a study-specific *ICN_Atlas* base map creating an ICN template with group ICA from the joint patient-control data (or from data of a specific age-group), and then co-registering is to any spatial template image (either general or study-specific), and then converting it to *ICN_Atlas* base map format.

### 4.2 Validation on longitudinal test-retest data

We chose the NYU-TRT data for our validation study because it is substantial in size, longitudinal, open-access and free-to-use, and well characterised (voxel-wise (Zuo et al., 2010b)). The results of our TC-GICA analysis are similar to Zuo et al., 2010a, Zuo et al., 2010b, with the main difference being the component ordering based on the ranking of the percentage of variance explained. This may be due to the different motion correction algorithms applied (Churchill et al., 2012a, Hoffmann et al., 2015, Power et al., 2015): SPM's algorithm in our case vs. FSL's in Zuo's. We note that although motion correction is a well-known problem in fMRI data analysis, especially for resting-state fMRI, to date no methodological consensus has emerged (Churchill et al., 2012b, Hoffmann et al., 2015, Kalcher et al., 2012, Power et al., 2015).

We also observed slightly different functional partitioning of the obtained ICs compared to those described by Zuo et al., 2010a, Zuo et al., 2010b, in which IC3 and IC5 represent the default mode network (DMN) and IC9, the fronto-parietal networks corresponding to cognition and language bilaterally. These differences may also be related to the different pre-processing pipelines used. Nevertheless, the similarity of our voxel-wise ICC results with those described by Zuo et al., 2010a, Zuo et al., 2010b, especially given the fact that ICs corresponding to intrinsic connectivity networks have higher ICC values than those corresponding to noise (with the exception of IC2) is reassuring. Concerning noise component IC2, its high degree of repeatability is not surprising given that it corresponds to the venous sinuses, an anatomically defined and therefore spatio-temporally stable entity.

Reassured by the above results we went on to assess atlasing repeatability for each metric at three levels: (1) individual atlasing steps for every IC - individual atlas base map combination; (2) atlas base maps; and (3) global, i.e. across ICs and atlas base maps. The results showed that repeatability is dependent both on the atlased activation map and the atlas base map used for atlasing (Fig. 7 and Supplementary Fig. 8). This finding is not unexpected, since activation maps have highly variable spatial distribution, hence there may be very limited or no overlap with some atlas base maps depending on the specific activation pattern which can lead to elevated variability, especially in the border regions of activation clusters thereby influencing the atlasing output due to a small number of voxels with values close to statistical significance (Supplementary Fig. 9).

Since our calculation of ICC at the atlas base map level, i.e. collapsing across ICs, reduces the impact of this IC-derived variability, it can be considered more a reliable assessment of the utility of the atlasing tool itself than the level of individual atlasing steps. At this level, the ICC values showed moderate to very strong agreement, on a par with the voxel-wise atlasing results, and similarly with the strong to very strong agreement observed at the global level. Regarding metric reliability we note the markedly lower value for ***MA***_***N,i***_, reflecting the maps’ greater inter-session variability in terms of overall activation level, an effect which is compensated for in the corresponding relative metric, ***RA***_***N,i***_. This observation suggests that the latter should be favoured in applications.

### 4.3 Demonstration on task-based data

To demonstrate *ICN_Atlas*’ utility on task-based fMRI data we selected an independent, task-based, open-access data set containing parametric (level of task difficulty) modulated data (Vagharchakian et al., 2012). Using this data were able to demonstrate parametric modulation effects in the atlasing output, reflecting task difficulty both for auditory and visual sentence presentation, which are compatible with previously published results and the previously proposed model of a temporal bottleneck in the language comprehension network that is independent of sensory limitation (Vagharchakian et al., 2012). As there was no information in the NeuroVault metadata to support proper significance thresholding, we opted for performing atlasing both on unthresholded input maps, and using an arbitrary (Z > 3) threshold, to emphasize the flexibility of *ICN_Atlas* as a research tool. Indeed, both approaches produced similar results for this data set.

Direct ROI-by-ROI comparison of the results was not possible due (1) to the limited available data, and (2) the different nature of ROIs: in Vagharchakian et al. (2012) they were derived using GLM ANOVA while those used in CONN132 were derived from anatomical landmarks. Indeed the CONN ROIs were much larger than the originally reported clusters, resulting in some of them (e.g. the left inferior occipital gyrus, the left precentral gyrus, and the left SMA) including clusters with different response profiles; this means that *ICN_Atlas* provides a different, more integrative, level of description; this is even more pronounced at the level of ICNs. This is clearly visible in the engagement profiles we obtained: while on the anatomical ROI level with the CONN132 atlas both the sensory, post bottleneck, and buffer response profiles were identifiable, on the ICN level with the BRAINMAP20 atlas the response profile resembled the phase profile suggested for integrative regions (Vagharchakian et al., 2012) both for the ***I***_***i***_ and the ***MA_N__,_***_***i***_ metrics. The latter represented network-wide behavior not exclusively driven by a single or a small group of ICNs, proven by the fact that overall engagements (average ***MA***_***N,i***_ and ***I***_***i***_ values over ICNs) followed the same response characteristic.

Moreover, despite the dominant integrative response profile, the stimulus modality could still be identified from the *ICN_Atlas* output, and there were visible differences in the engagement dynamics of the BRAINMAP20 ICNs, but their detailed analysis is outside the scope of this paper.

Note that, the response profiles were identified visually, as it was not possible to characterize *ICN_Atlas* output on a ROI-by-ROI basis using correlation-based statistics due to the small number of data points available (five for each stimulus modality).

### 4.4 Demonstration on epileptic seizures EEG-fMRI data

We obtained results with *ICN_Atlas* using the dataset of a patient who had repeated seizures during resting-state fMRI scanning, confirmed on simultaneously recorded EEG and video (Chaudhary et al., 2012). The results significant and varying engagement of a range of ICN in different epileptic phases. Specifically, there is a degree of correspondence between the patterns of ICN engagement in this seizure and ictal semiology. Our observation of activation of the DMN during the ictal established phase is consistent with observation of disturbance in normal level of consciousness. In turn, DMN activation is not normally associated with activation of the sensorimotor network (ICN6) and associated manifest motor activity nor with activation of the executive and fronto-parietal networks.

An implicit observation that results from this particular application of *ICN_Atlas* is in relation to the eminence of the Default mode network (DMN) in research. Whilst there has been a level of interest focused on the DMN in epilepsy imaging studies (Gotman et al., 2005, Laufs et al., 2006), this may in part be accounted more by its historical pre-eminence in the field of functional imaging than some intrinsic *a priori* clinical relevance even though fluctuations in awareness and or consciousness are an important consideration in epilepsy (Archer et al., 2003, Berman et al., 2010, Chaudhary et al., 2012, Laufs et al., 2006). We suggest that use of *ICN_Atlas* will help to widen the investigation of the role of other intrinsic connectivity networks in Epileptology. Note however, that the importance of the mesial temporal lobe structures in epilepsy highlights a limitation of the SMITH10 atlas in this field of application.

*ICN_Atlas* not only addresses the issue of interpretation bias (e.g. focus on more or less visually prominent or perceived as interesting) but also introduces objectivity in providing a standardised approach to the characterization of epileptic networks in the clinical context. We refer to the fact that neuroimaging networks are often labelled and referenced generally in a purely visual and qualitative manner, relying on the investigators knowledge of functional localizers (Friston et al., 2006, Saxe et al., 2006), or that of basic functional neuroanatomy as it is evident from the taxonomy of brain activation databases (Laird et al., 2005, Laird et al., 2011, Yarkoni et al., 2011).

Our approach raises the question: To what extent do these patterns of intrinsic connectivity networks activation manifest in seizure semiology? This is a question which should be addressed on an individual and group level. Whilst the illustrative case study provides notional correlation with manifest semiology as it can be understood in terms of network engagement, it raises interesting questions as to the impact of seizures on normal connectivity and cognition, including executive function in relation to normal levels of consciousness. We will further address these issues in future studies.

Furthermore, *ICN_Atlas* allows for more sophisticated analyses than currently performed via quantitative assessment of ICN engagement and thus may add to the debate in relation to the neurobiological nature of seizure networks (Bartolomei et al., 2001, Spencer, 2002, Thornton et al., 2011). For example on a descriptive level, ICN engagement in terms of voxel numbers as well as sum of statistical values, may reflect a predominance of specific ICNs. Such findings can notionally address questions, raised in the literature with respect to the interpretation of BOLD changes found in deeper structures, i.e. whether they reflect normal network activation consequent to ictal activation or indeed widespread underlying abnormalities (Chaudhary et al., 2012). A better understanding of the intrinsic connectivity network composition of clusters in the *ictal* BOLD maps is likely to improve the interpretation of epileptic activity and therefore improve localisation, particularly in comparison to other relevant investigation and descriptions of ICNs (Mantini et al., 2007, van den Heuvel et al., 2009).

### 4.5 Limitations of the proposed approach

The current version of *ICN_Atlas* employs base atlases based on group data, which can be considered as first-degree approximations of each network's representations as independent components, hence they do not reflect inter-individual variability in the networks. Indeed, atlases obtained from *meta*-ICA decomposition of group-level ICA data (Smith et al., 2009) are fundamentally different from maps obtained with ICA decomposition of individual data (Zuo et al., 2010b). This criticism applies to all methods that base their interpretation on these atlases. A theoretical solution to this issue would be a probabilistic base atlas based on individual ICN and/or activation data that may be better suited to represent single-subject activation patterns. We envisage the creation of a probabilistic version of *ICN_Atlas* in which the metrics take into account base atlas voxel weightings.

The three ICN base atlases currently provided with *ICN_Atlas* offer a relatively coherent framework for the description of activations with respect to ICNs. One could envisage that the use of other, custom, base atlases could lead to inconsistencies of description that may hinder comparison across studies. These inconsistencies may nevertheless be reduced by ‘cross-atlasing’ the atlas base maps, e.g. as we presented the comparison of SMITH10 vs BRAINMAP20, and BRAINMAP20 vs BRAINMAP70 in Supplementary Figs. 3 and 4.

We have demonstrated *ICN_Atlas’* utility and flexibility for the description of group-level task-based parametric fMRI data both on unthresholded input maps, and using an arbitrary (Z > 3) threshold; with both approaches having produced similar results for this data set. It is at the discretion of the user to set threshold values, nevertheless there is a default threshold for the atlas base maps set to *Z* = 3, while the simplest recommended approach for the input maps is to use conventional model-based (e.g. Gaussian random field) statistical thresholding.

We have demonstrated *ICN_Atlas'* utility for the description of single-subject epileptic activities derived from EEG-fMRI data. Based on these results we can safely conclude that it can easily and effectively be used as a comparative tool in clinical studies. Nevertheless, it is important to recognise the limitations imposed by standardised and normative tools such as *ICN_Atlas* in single subject analyses. Whilst speed and standardisation is advantageous even in the clinical context, investigators have to be mindful of the fact that ICN-based assessment of individual activation maps (be they EEG-fMRI-derived ictal- or task-related BOLD maps) based on each individual patients’ own intrinsic connectivity networks may be advantageous at least in principle.

Finally, *ICN_Atlas* at its current state can be considered a data summarizer. In the current work we have not considered how the outputs can be analysed in order to discover new neuroscientific facts, beyond the factor analysis to identify a reduced set of metrics. We think that the simplest presentation is necessary at this stage, and that this relative functional simplicity and transparency may help the tool being adopted.

### 4.6 Possible extensions and applications

The scope and spirit of this paper being confined to the presentation, validation and limited demonstration of the utility of our tool, we did not wish to introduce too great a bias in the way it could be applied (although for example the choice of a small number of metrics presented can be justified as being the result of a factor analysis, and to simplify presentation for publication) or how the results should be interpreted. While this can be considered a shortcoming, we believe that the open framework we propose takes into account a degree of uncertainty on the exact nature and extent of the intrinsic connectivity networks. By offering the users the option of choosing a (any) ‘base atlas’ (in the *ICN_Atlas* terminology) of their own preference, we offer the scientific community the possibility of discovering, or agreeing on, the most suitable or optimal atlas for a given specific purpose, or perhaps a large range of applications. This is equally true for the flexible thresholding options implemented for the analyses, which allow for data input derived from different sources besides the SPM toolbox even without statistical information encoded in the file headers. *ICN_Atlas* is a just a tool and, with every other tool, it is the users' responsibility to adhere to proper analysis standards.

The extensible nature of *ICN_Atlas* provides opportunity to include atlas base maps derived from different sources, e.g. probabilistic anatomy (Eickhoff et al., 2005), pediatric ICNs (Muetzel et al., 2016, Thomason et al., 2011), multi-modal anatomical parcellations (Glasser et al., 2016), or even study-specific functional localizers. These extensions could help fine tune the toolbox for the investigators specific needs. On the same token, as the toolbox expects its input to be in the same anatomical space as the data, species specific atlas base maps can also be used for the processing of animal-derived data.

Overcoming the current limitation of being a data summarizer would require the implementation of in-depth analysis approaches. These could include statistical inference for group comparisons, function decoding based on e.g. the BrainMap database using their taxonomical meta-data labelling scheme (Laird et al., 2011) for reverse inference (Yarkoni et al., 2011), etc. We have already demonstrated the utility of factor analysis for identifying the most relevant metrics from the wide range of possible output parameters, but including factor analysis for group-level processing may shed light for differential importance of metrics depending on the research question, or the clinical group investigated.

We developed *ICN_Atlas* with EEG-fMRI in our focus of attention, but the toolset is not limited nor to this acquisition method, neither for the discussion of epileptic activities. Indeed, the approaches to analysis discussed above may provide quantitative assessments of activation data in relation to a range of neuroscientific and clinical questions. Regarding the study of epilepsy-related activations: it is evident that there are significant differences between ictal phases, and as reflected by the metric values. In a departure from the quest for localisation by virtue of cluster classification in terms of statistical significance, *ICN_Atlas* provides a description of intrinsic connectivity network engagement that lends itself to a depiction of activations in terms of functional significance (Centeno and Carmichael, 2014) and could be a potential contributor to the current pre-surgical cluster interpretation in EEG-fMRI studies as well as providing information on semiology in such studies.

## 5 Conclusions

*ICN_Atlas* provides a fast, flexible and objective quantitative comparative approach for characterizing fMRI activation patterns based on functionally-derived atlases of the investigator's choice. It can be applied to activation studies of any nature, providing objective, reproducible and meaningful descriptions of fMRI maps. Based on the presented case demonstration it may open new avenue of research into the cognitive aspects of a range of neurological conditions.

## Appendix A Definition of the ICN recruitment metrics

We used the following variables and symbols in the metrics definitions:

• n: voxel index (*n* = 1, 2, …, *M*, where *M* is the number of voxels in the maps);
• *SPM* is an input map and $SPM_{t}$ its thresholded version;
• $ICN_{i}$ represents the *i*th thresholded prototype ICN map in atlas A, and $ICN_{i}^{B}$ represents the binarized version of the *i*th thresholded prototype ICN map in atlas A
• |X|: is the number of non-zero valued voxels in map X;
• ${\langle X\rangle }_{n}$: is the value of the given voxel n in map X;
• : represent the voxel-wise product
• a horizontal bar over any given variable or metric represents its arithmetic mean over all voxels, e.g. in $\bar{\langle SPM\rangle }$.

Note that in the description of the metrics we use the term activation, although the metrics are also defined for deactivations, and the *ICN_Atlas* toolbox can be applied to deactivation data and used to estimate the metrics for positive and negative input map voxel values.

### A.1 ICN spatial involvement metrics

These metrics represent the proportion of ICN activated on global and local (ICN-wise) levels in the input map, and/or the spatial similarity between the input map thresholded activation pattern and the ICN.

**ICN**_**i**_ **Spatial Involvement (*I***_***i***_**)**: ratio of the number of activated ICN_i_ voxels to ICN_i_ volume:

(A.1) $$I_{i}=\frac{\left|SPM_{t}⋂ICN_{i}\right|}{\left|ICN_{i}\right|}$$

**Total ICN Spatial Involvement (*I***_***T***_**)**: ratio of the total number of activated ICN voxels over the total ICN volume:

(A.2) $$I_{T}=\frac{\sum_{i}\left|SPM_{t}⋂ICN_{i}\right|}{\sum_{i}\left|ICN_{i}\right|}$$

**ICN**_**i**_ **Relative Spatial Involvement (*IR***_***i***_**)**: ratio of the number of activated ICN_i_ voxels over the total number of activated ICN voxels:

(A.3) $$IR_{i}=\frac{\left|SPM_{t}⋂ICN_{i}\right|}{\sum_{i}\left|SPM_{t}⋂ICN_{i}\right|}$$

**Spatial Overlap with ICN**_**i**_ **(*OL***_***i***_**):** spatial overlap between the thresholded activation map and ICN_i_ (Kim and Lee, 2012):

(A.4) $$OL_{i}=\sqrt{\frac{{\left|SPM_{t}⋂ICN_{i}\right|}^{2}}{\left|SPM_{t}\right|\cdot \left|ICN_{i}\right|}}$$

**Sørensen-Dice coefficient with ICN**_**i**_ **(*SQ***_***i***_**):** spatial similarity between the thresholded activation map and ICN_i_:

(A.5) $$SQ_{i}=\frac{2\;\left|SPM_{t}⋂ICN_{i}\right|}{\left|SPM_{t}\right|+\left|ICN_{i}\right|}$$

**Jaccard index with ICN**_**i**_ **(*J***_***i***_**):** spatial similarity index between the thresholded activation map and ICN_i_:

(A.6) $$J_{i}=\frac{\left|SPM_{t}⋂ICN_{i}\right|}{\left|SPM_{t}⋃ICN_{i}\right|}$$

### A.2 ICN activation strength metrics

These metrics represent the significance/strength of ICN activation. Intensity-normalised metrics are defined to estimate the importance of ICN involvement relative to the whole-brain range of activation levels.

**Mean ICN**_**i**_ **Activation (*MA***_***i***_**)**: ratio of the sum of ICN_i_ statistical values to the number of activated ICN_i_ voxels:

(A.7) $$MA_{i}=\frac{\sum_{n}{\langle SPM_{t}\rangle }_{n}\times ICN_{i,n}^{B}\;}{\left|SPM_{t}⋂ICN_{i}\right|}$$

**Global Mean ICN Activation (*MA*)**: mean of voxel-wise statistical values and the number of activated voxels in all ICN:

(A.8) $$MA=\frac{\sum_{i}\sum_{n}{\langle SPM_{t}\rangle }_{n}\times ICN_{i,n}^{B}}{\sum_{i}\left|SPM_{t}⋂ICN_{i}\right|}\text{,}$$

**Normalised Global Mean ICN Activation (*MA***_***N***_**)**: ratio of the mean of normalised voxel-wise statistical values over the number of activated voxels in all ICN:

(A.9) $$MA_{N}=\frac{\sum_{i}\sum_{n}\frac{{\langle SPM_{t}\rangle }_{n}\times ICN_{i,n}^{B}-\;min\;\langle SPM_{t}\rangle }{max\;\langle SPM_{t}\rangle -min\;\langle SPM_{t}\rangle }\;}{\sum_{i}\left|SPM_{t}⋂ICN_{i}\right|}$$

here $min\;SPM_{t}$ is the minimum of the thresholded map, i.e. the threshold value itself, and $max\;SPM_{t}$ is the peak statistical value, so $\;\frac{{\langle SPM_{t}\rangle }_{n}\times ICN_{i,n}^{B}-\;min\;\langle SPM_{t}\rangle }{max\;\langle SPM_{t}\rangle -min\;\langle SPM_{t}\rangle }$ normalises the statistical values between the threshold and the global maximum to the [0,1] interval.

**Normalised Mean ICN**_**i**_ **Activation (*MA***_***N,i***_**)**: ratio of the mean of normalised voxel-wise statistical values and the number of activated voxels in ICN_i_:

(A.10) $$MA_{N,i}=\frac{\sum_{n}\frac{{\langle SPM_{t}\rangle }_{n}\times ICN_{i,n}^{B}-\;min\;\langle SPM_{t}\rangle }{max\;\langle SPM_{t}\rangle -min\langle \;SPM_{t}\rangle }\;}{\left|SPM_{t}⋂ICN_{i}\right|}$$

**Relative Normalised Mean ICN**_**i**_ **Activation (**$IR_{i}^{M}$**)**: ratio of the sum of normalised statistical values for ICN_i_ over the number of activated voxels in all ICN; equivalent to the mean of voxel-wise statistical values over activated voxels in all ICN:

(A.11) $$IR_{i}^{M}=\frac{\sum_{n}\frac{{\langle SPM_{t}\rangle }_{n}\times ICN_{i,n}^{B}-\;min\;\langle SPM_{t}\rangle }{max\;\langle SPM_{t}\rangle -min\;\langle SPM_{t}\rangle }}{\sum_{i}\left|SPM_{t}⋂ICN_{i}\right|}=MA_{N,i}\cdot IR_{i}$$

**Normalised Relative ICN**_**i**_ **Activation (*RA***_***N,i***_**)**: ratio of the summed normalised activation in the given ICN and the total normalised activation in all ICN:

(A.12) $$RA_{N,i}=\frac{\sum_{n}\frac{{\langle SPM_{t}\rangle }_{n}\times ICN_{i,n}^{B}-\;min\;\langle SPM_{t}\rangle }{max\;\langle SPM_{t}\rangle -min\langle \;SPM_{t}\rangle }}{\sum_{i}\sum_{n}\frac{{\langle SPM_{t}\rangle }_{n}\times ICN_{i,n}^{B}-\;min\langle \;SPM_{t}\rangle }{max\;\langle SPM_{t}\rangle -min\;\langle SPM_{t}\rangle }}$$

### A.3 ICN activation density metrics

Similarly to the normalised activation strength metrics these metrics represent the significance/strength of ICN activation, relative to the ICN volume rather than the activated ICN volume.

**Normalised Global Mean ICN Activation Density (**$I_{T}^{M}$**)**: ratio of the sum of normalised statistical values over all ICN to total ICN:

(A.13) $$I_{T}^{M}=\frac{\left(\sum_{i}\sum_{n}\frac{\langle SPM_{t}\rangle \times ICN_{i,n}^{B}-\;min\;\langle SPM_{t}\rangle }{max\;\langle SPM_{t}\rangle -min\langle \;SPM_{t}\rangle }\;\right)}{\sum_{i}\left|ICN_{i}\right|}=\;MA_{N}\cdot I_{t}$$

**Normalised Mean ICN**_**i**_ **Activation Density (**$I_{i}^{M}$**)**: ratio of the sum of normalised statistical values for ICN_i_ to ICN_i_ volume:

(A.14) $$I_{i}^{M}=\frac{\sum_{n}\frac{{\langle SPM_{t}\rangle }_{n}\times ICN_{i,n}^{B}-\;min\;\langle SPM_{t}\rangle }{max\;\langle SPM_{t}\rangle -min\;\langle SPM_{t}\rangle }}{\left|ICN_{i}\right|}=MA_{N,i}\cdot I_{i}$$

### A.4 Other

**Pearson's spatial correlation with ICN**_**i**_ **(*r***_***i***_**):** measures the similarity between the full activation map and ICN_i_ along the voxel dimension; this metric can be used on unthresholded maps and unthresholded atlases, as well.

(A.15) $$r_{i}=\frac{\sum_{n}\left({\langle SPM_{t}\rangle }_{n}-\langle \bar{SPM_{t}}\rangle \right)\;\left({\langle ICN_{i}\rangle }_{n}-\langle \bar{ICN_{i}}\rangle \;\right)}{{\left[\sum_{n}{\left({\langle SPM_{t}\rangle }_{n}-\langle \bar{SPM_{t}}\rangle \right)}^{2}\sum_{n}\left({\langle ICN_{i}\rangle }_{n}-\langle ICN_{i}\rangle \;\right){\;}^{2}\right]}^{1/2}}$$

## Appendix B Estimating repeatability using intra-class correlation

Quantifying a measurement's repeatability, which can be defined “as precision under conditions where independent test/measurement results are obtained with the same method on identical test/measurement items in the same test or measuring facility by the same operator using the same equipment within short intervals of time” (ISO/IEC 3534-2:2006 3.3.5), is essentially an exercise in the more general problem of measuring agreement between measurements (Bland and Altman, 1986, Muller and Buttner, 1994). Considering the well-known potential sources of systematic session-specific (and biologically uninteresting) bias in fMRI, we chose to follow Zuo et al. (2010b) to estimate repeatability. Following Shrout and Fleiss (1979), they propose a formulation of the intra-class correlation coefficient that does not penalize for systematic differences between scanning sessions, within the framework of the analysis of variance (Zuo et al., 2010b).

In summary, in the context of repeat image acquisitions, denoting *Y*_*ij*_ as the *j*-th measurement (voxel value) on the *i*-th subject (for *i* = 1, …, *n* and *j* = 1, …, *d*), modelled as follows:

$$Y_{\mathrm{ij}}=\mu +p_{i}+t_{j}+e_{\mathrm{ij}}\;1\leq i\leq \mathrm{n;}1\leq j\leq d$$

where *μ* is a fixed parameter (some global effect), *p*_*i*_ is the participant effect (variance *σ*^*2*^_*p*_), *t*_*j*_ a scanning session effect (variance *σ*^*2*^_*t*_) and *e*_*ij*_ the measurement error (variance *σ*^*2*^_*e*_). The intra-class correlation (ICC), initially proposed by Fisher (1954) to assess the similarity between corresponding (i.e., repeated) measurements across a group of such measurements (e.g. across specimens in a sample), considering all effects contained in the model, is expressed as:

(B.1) $$ICC_{t}=\frac{\sigma_{p}^{2}}{\sigma_{p}^{2}+\sigma_{t}^{2}+\sigma_{e}^{2}}$$

Ignoring systematic sources of error, Zuo et al., 2010a, Zuo et al., 2010b define a modified ICC, used in this work:

(B.2) $$ICC=\frac{\sigma_{p}^{2}}{\sigma_{p}^{2}+\sigma_{e}^{2}}\text{,}$$

the mean square estimate of which can be written as:

(B.3) $$ICC=\;\frac{{\overset{⌢}{\sigma }}_{p}^{2}}{{\overset{⌢}{\sigma }}_{p}^{2}+{\overset{⌢}{\sigma }}_{e}^{2}}=\frac{MS_{p}\;-\;MS_{e}}{MS_{p}\;+\left(d\;-\;1\right)MS_{e}}\text{,}$$

where:

*MS*_*p*_ = *SS*_*p*_/(*n*−1) (B.4)
*MS*_*e*_ = *SS*_*e*_/((*n*−1)(d-1)) (B.5)

with:

(B.6) $$SS_{p}=d\sum_{i=1}^{n}{\left({\bar{Y}}_{i}-\bar{Y}\right)}^{2}\;$$

(B.7) $$SS_{e}={\sum_{i=1}^{n}\sum_{j=1}^{d}\left(Y_{ij}-\left({\bar{Y}}_{i}+{\bar{Y}}_{j}-{\bar{Y}}_{..}\right)\right)}^{2}\;$$

where:

(B.8) $${\bar{Y}}_{i}=\left(\frac{1}{d}\right)\sum_{j=1}^{d}Y_{ij}\text{,}$$

(B.9) $$\;{\bar{Y}}_{j}=\left(\frac{1}{n}\right)\sum_{i=1}^{n}Y_{ij}\text{,}\;$$

(B.10) $${\bar{Y}}_{..}=\left(\frac{1}{dn}\right)\sum_{j=1}^{d}\sum_{i=1}^{n}Y_{ij}\;$$

## Footnotes

Appendix C Supplementary data related to this article can be found at https://doi.org/10.1016/j.neuroimage.2017.09.014.
