# Supplementary material for: ICN_Atlas: Automated description and quantification of functional MRI activation patterns in the framework of intrinsic connectivity networks
Source: Neuroimage. 2017 Dec;163:319–41. doi: 10.1016/j.neuroimage.2017.09.014 (PMC5725313; doi:10.1016/j.neuroimage.2017.09.014)
Supplement: Supplementary file 1 [file mmc1.pdf]

Supplementary Results

Normalised Mean ICN$_i$ Activation (*$MA_{N,i}$*): for SMITH10 (Supplementary Figure 6, Supplemetary Table 5), amongst the functional ICs the highest $MA_{N,i}$ was observed for IC1 (visual): $MA_{N,ICN1}$ = 0.33, then in the order of decreasing highest $MA_{N,i}$ were IC3 (DMN): $MA_{N,ICN4}$ = 0.26; IC8 (auditory and executive control): $MA_{N,ICN5}$ = 0.25; IC9 (fronto-parietal): $MA_{N,ICN10}$ = 0.24; IC5 (DMN): $MA_{N,ICN10}$ = 0.22; IC7 (sensory-motor and auditory): $MA_{N,ICN6}$ = 0.22; IC20 (ventromedial prefrontal): $MA_{N,ICN4}$ = 0.20; IC15 (cerebellar): $MA_{N,ICN5}$ = 0.19; IC11 (executive control): $MA_{N,ICN9}$ = 0.17; IC6 (visual and motor): $MA_{N,ICN10}$ = 0.17; IC14 (temporal lobe, HC): $MA_{N,ICN7}$ = 0.15; IC18 (visual): $MA_{N,ICN2}$ = 0.15 and IC19 (temporal lobe, HC): $MA_{N,ICN5}$ = 0.15. For the noise ICs the order was the following: IC4: $MA_{N,ICN6}$ = 0.50; IC13: $MA_{N,ICN5}$ = 0.26; IC2: $MA_{N,ICN4}$ = 0.20; IC10: $MA_{N,ICN2}$ = 0.19; IC12: $MA_{N,ICN2}$ = 0.18; IC16: $MA_{N,ICN4}$ = 0.17; IC17: $MA_{N,ICN8}$ = 0.11; note that I_T did not exceed 0.04 for any of the noise ICs.

For BRAINMAP20 (Supplementary Table 6) amongst the functional ICs the highest $MA_{N,i}$ was observed for IC1 (visual): $MA_{N,BM20\text{-}12}$ = 0.31, then in the order of decreasing highest $MA_{N,i}$ were IC9 (fronto-parietal): $MA_{N,BM20\text{-}18}$ = 0.26; IC3 (DMN): $MA_{N,BM20\text{-}12}$ = 0.25; IC5 (DMN): $MA_{N,BM20\text{-}20}$ = 0.24; IC7 (sensory-motor and auditory): $MA_{N,BM20\text{-}9}$ = 0.24; IC8 (auditory and executive control): $MA_{N,BM20\text{-}4}$ = 0.22; IC20 (ventromedial prefrontal): $MA_{N,BM20\text{-}2}$ = 0.22; IC6 (visual and motor): $MA_{N,BM20\text{-}7}$ = 0.20; IC15 (cerebellar): $MA_{N,BM20\text{-}8}$ = 0.20; IC11 (executive control): $MA_{N,BM20\text{-}5}$ = 0.18; IC19 (temporal lobe, HC): $MA_{N,BM20\text{-}19}$ = 0.16; IC14 (temporal lobe, HC): $MA_{N,BM20\text{-}1}$ = 0.16 and IC18 (visual): $MA_{N,BM20\text{-}11}$ = 0.15. For the noise ICs the order was the following: IC4: $MA_{N,BM20\text{-}20}$ = 0.39; IC10: $MA_{N,BM20\text{-}2}$ = 0.28; IC17: $MA_{N,BM20\text{-}19}$ = 0.27; IC13: $MA_{N,BM20\text{-}5}$ = 0.25; IC2: $MA_{N,BM20\text{-}11}$ = 0.22; IC16: $MA_{N,BM20\text{-}3}$ = 0.21 and IC12: $MA_{N,BM20\text{-}11}$ = 0.19; note that I_T did not exceed 0.06 for any of the noise ICs.

For BRAINMAP70 (Supplementary Table 7) amongst the functional ICs the highest $MA_{N,i}$ was observed for IC1 (visual): $MA_{N,BM70\text{-}2}$ = 0.41 then in the order of decreasing highest $MA_{N,i}$ were IC3 (DMN): $MA_{N,BM70\text{-}61}$ = 0.31; IC20 (ventromedial prefrontal): $MA_{N,BM70\text{-}20}$ = 0.28; IC5 (DMN): $MA_{N,BM70\text{-}28}$ = 0.28; IC9 (fronto-parietal): $MA_{N,BM70\text{-}51}$ = 0.26; IC7 (sensory-motor and auditory): $MA_{N,BM70\text{-}35}$ = 0.26; IC8 (auditory and executive control): $MA_{N,BM70\text{-}52}$ = 0.26; IC6 (visual and motor): $MA_{N,BM70\text{-}7}$ = 0.24; IC14 (temporal lobe, HC): $MA_{N,BM70\text{-}10}$ = 0.23; IC15 (cerebellar): $MA_{N,BM70\text{-}60}$ = 0.20; IC11 (executive control): $MA_{N,BM70\text{-}11}$ = 0.17; IC19 (temporal lobe, HC): $MA_{N,BM70\text{-}39}$ = 0.17; and IC18 (visual): $MA_{N,BM70\text{-}3}$ = 0.15. For the noise ICs the order was the following: IC4: $MA_{N,BM70\text{-}69}$ = 0.44; IC10: $MA_{N,BM70\text{-}68}$ = 0.34; IC16: $MA_{N,BM70\text{-}24}$ = 0.27; IC13: $MA_{N,BM70\text{-}65}$ = 0.26; IC17: $MA_{N,BM70\text{-}70}$ = 0.25; IC2: $MA_{N,BM70\text{-}3}$ = 0.23; and IC12: $MA_{N,BM70\text{-}3}$ = 0.19; note that I_T did not exceed 0.08 for any of the noise ICs.

Normalised Relative ICN$_i$ Activation (*$RA_{N,i}$*): for SMITH10 (Supplementary Figure 7, Supplemetary Table 8), amongst the functional ICs the highest $\boldsymbol{RA_{N,i}}$ was observed for IC15 (cerebellar): $\boldsymbol{RA_{N,ICN5}}$ = 0.93, then in the order of decreasing highest $\boldsymbol{RA_{N,i}}$ were IC20 (ventromedial prefrontal): $\boldsymbol{RA_{N,ICN8}}$ = 0.73; IC1 (vision): $\boldsymbol{RA_{N,ICN1}}$ = 0.66; IC9 (fronto-parietal): $\boldsymbol{RA_{N,ICN10}}$ = 0.60; IC7 (sensory-motor and auditory): $\boldsymbol{RA_{N,ICN6}}$ = 0.58; IC19 (temporal lobe, HC): $\boldsymbol{RA_{N,ICN5}}$ = 0.56; IC14 (temporal lobe, HC): $\boldsymbol{RA_{N,ICN7}}$ = 0.55; IC18 (vision): $\boldsymbol{RA_{N,ICN2}}$ = 0.55; IC3 (DMN): $\boldsymbol{RA_{N,ICN4}}$ = 0.54; IC11 (executive control): $\boldsymbol{RA_{N,ICN8}}$ = 0.44; IC8 (auditory and executive control): $\boldsymbol{RA_{N,ICN7}}$ = 0.44; IC6 (visual and motor): $\boldsymbol{RA_{N,ICN3}}$ = 0.34; IC5 (DMN): $\boldsymbol{RA_{N,ICN4}}$ = 0.28. For the noise ICs the order was the following: IC13: $\boldsymbol{RA_{N,ICN5}}$ = 0.96; IC12: $\boldsymbol{RA_{N,ICN2}}$ = 0.75; IC16: $\boldsymbol{RA_{N,ICN8}}$ = 0.59; IC4: $\boldsymbol{RA_{N,ICN8}}$ = 0.51; IC17: $\boldsymbol{RA_{N,ICN10}}$ = 0.50; IC10: $\boldsymbol{RA_{N,ICN2}}$ = 0.42; and IC2: $\boldsymbol{RA_{N,ICN2}}$ = 0.23.

For BRAINMAP20 (Supplementary Table 9), amongst the functional ICs the highest $\boldsymbol{RA_{N,i}}$ was observed for IC1 (vision): $\boldsymbol{RA_{N,BM20\text{-}12}}$ = 0.72; IC18 (vision): $\boldsymbol{RA_{N,BM20\text{-}11}}$ = 0.68; IC20 (ventromedial prefrontal): $\boldsymbol{RA_{N,BM20\text{-}2}}$ = 0.67; IC14 (temporal lobe, HC): $\boldsymbol{RA_{N,BM20\text{-}1}}$ = 0.65; IC15 (cerebellar): $\boldsymbol{RA_{N,BM20\text{-}14}}$ = 0.49; IC6 (visual and motor): $\boldsymbol{RA_{N,BM20\text{-}7}}$ = 0.44; IC19 (temporal lobe, HC): $\boldsymbol{RA_{N,BM20\text{-}1}}$ = 0.40; IC3 (DMN): $\boldsymbol{RA_{N,BM20\text{-}13}}$ = 0.30; IC8 (auditory and executive control): $\boldsymbol{RA_{N,BM20\text{-}4}}$ = 0.25; IC11 (executive control): $\boldsymbol{RA_{N,BM20\text{-}20}}$ = 0.24; IC7 (sensory-motor and auditory): $\boldsymbol{RA_{N,BM20\text{-}6}}$ = 0.23; IC5 (DMN): $\boldsymbol{RA_{N,BM20\text{-}13}}$ = 0.22 and IC9 (fronto-parietal): $\boldsymbol{RA_{N,BM20\text{-}15}}$ = 0.21. For the noise ICs the order was the following: IC13: $\boldsymbol{RA_{N,BM20\text{-}5}}$ = 0.70; IC12: $\boldsymbol{RA_{N,BM20\text{-}11}}$ = 0.35; IC4 : $\boldsymbol{RA_{N,BM20\text{-}5}}$ = 0.35; IC17: $\boldsymbol{RA_{N,BM20\text{-}1}}$ = 0.34; IC16: $\boldsymbol{RA_{N,BM20\text{-}3}}$ = 0.30; IC10 : $\boldsymbol{RA_{N,BM20\text{-}2}}$ = 0.25 and IC2: $\boldsymbol{RA_{N,BM11}}$ = 0.24.

For BRAINMAP70 (Supplementary Table 10) amongst the functional ICs the highest $\boldsymbol{RA_{N,i}}$ was observed for IC18 (visual): $\boldsymbol{RA_{N,BM70\text{-}3}}$ = 0.54 then in the order of decreasing highest $\boldsymbol{RA_{N,i}}$ were IC19 (temporal lobe, HC): $\boldsymbol{RA_{N,BM70\text{-}39}}$ = 0.33; IC15 (cerebellar): $\boldsymbol{RA_{N,BM70\text{-}60}}$ = 0.30; IC1 (visual): $\boldsymbol{RA_{N,BM70\text{-}1}}$ = 0.27; IC3 (DMN): $\boldsymbol{RA_{N,BM70\text{-}61}}$ = 0.26; IC14 (temporal lobe, HC): $\boldsymbol{RA_{N,BM70\text{-}41}}$ = 0.25; IC20 (ventromedial prefrontal): $\boldsymbol{RA_{N,BM70\text{-}20}}$ = 0.25; IC6 (visual and motor): $\boldsymbol{RA_{N,BM70\text{-}7}}$ = 0.19; IC5 (DMN): $\boldsymbol{RA_{N,BM70\text{-}28}}$ = 0.15; IC8 (auditory and executive control): $\boldsymbol{RA_{N,BM70\text{-}52}}$ = 0.13; IC7 (sensory-motor and auditory): $\boldsymbol{RA_{N,BM70\text{-}40}}$ = 0.11; IC11 (executive control): $\boldsymbol{RA_{N,BM70\text{-}69}}$ = 0.11; and IC9 (fronto-parietal): $\boldsymbol{RA_{N,BM70\text{-}53}}$ = 0.09. For the noise ICs the order was the following: IC17: $\boldsymbol{RA_{N,BM70\text{-}56}}$ = 0.51; IC13: $\boldsymbol{RA_{N,BM70\text{-}56}}$ = 0.32; IC12: $\boldsymbol{RA_{N,BM70\text{-}3}}$ = 0.30; IC4: $\boldsymbol{RA_{N,BM70\text{-}56}}$ = 0.25; IC10: $\boldsymbol{RA_{N,BM70\text{-}53}}$ = 0.22; IC2: $\boldsymbol{RA_{N,BM70\text{-}66}}$ = 0.23; and IC16: $\boldsymbol{RA_{N,BM70\text{-}58}}$ = 0.14.
