# Supplementary material for: ICN_Atlas: Automated description and quantification of functional MRI activation patterns in the framework of intrinsic connectivity networks
Source: Neuroimage. 2017 Dec;163:319–41. doi: 10.1016/j.neuroimage.2017.09.014 (PMC5725313; doi:10.1016/j.neuroimage.2017.09.014)

Supplementary Figures

***Supplementary Figure 1. Correspondence of the I_i, $MA_{N,i}$ and $RA_{N,i}$ metrics for the BRAINMAP70 atlas**. Colour coding is according to engagement values for each IC (columns in each panel) and each atlas base map (rows in each panel), the three highest values for each IC (each column) are marked with white dots in each panel. White vertical bars separate functional ICs from noise ICs, black squares on **$MA_{N,i}$** panels show atlas base maps for given ICs where no voxel was active (i.e. I_i=0), therefore **$MA_{N,i}$** is not calculated. The highest three **I_i** values for any given IC represent 21-80% of the total **I_i** for the given IC for BRAINMAP70 (see the last rows of Supplementary Tables 4, 7, and 10 for details).*

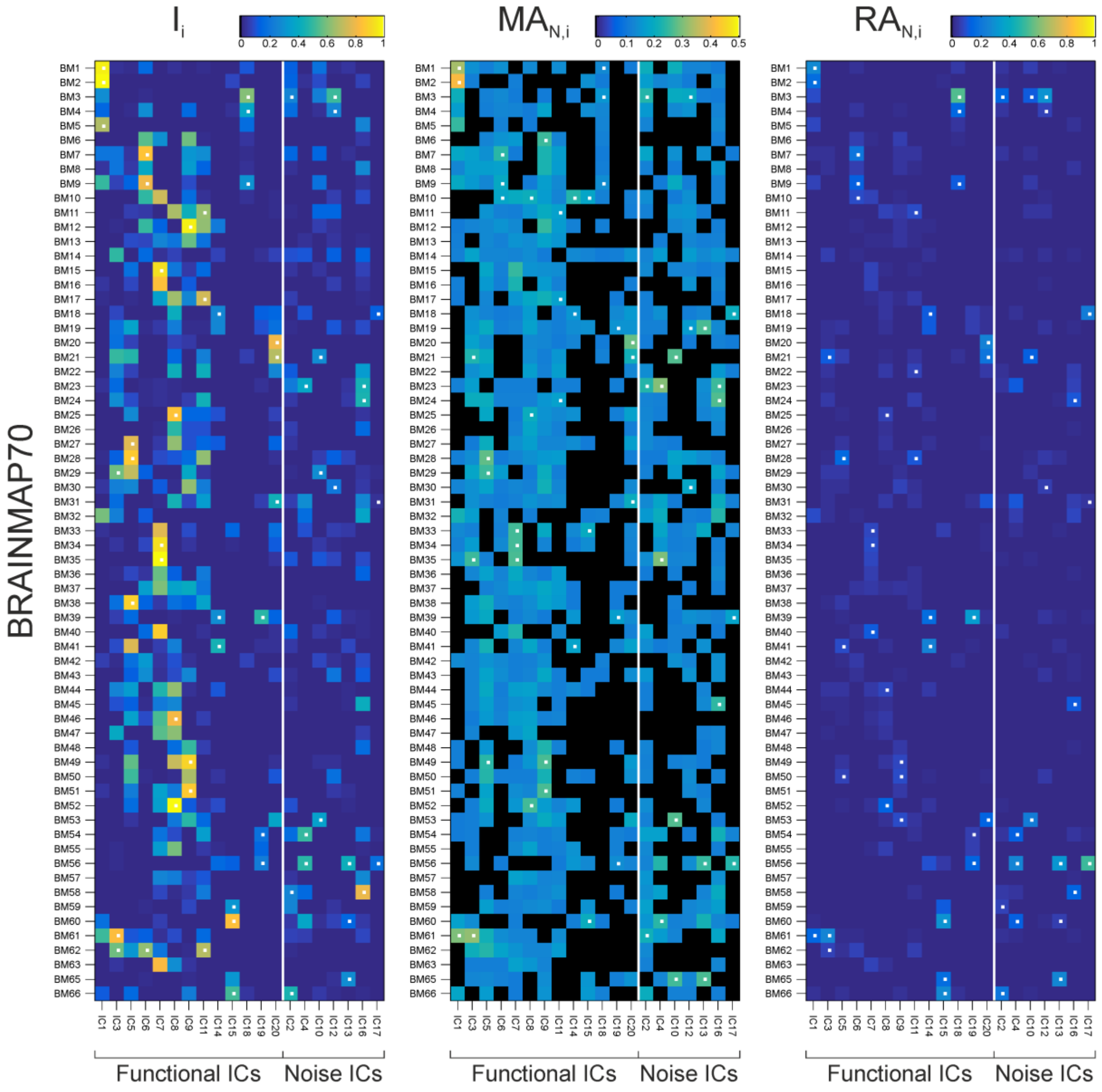

Supplementary Figure 2. ICN$_i$ Spatial Involvement (I_i) of the NYU-TRT group-ICA components for the BRAINMAP70 atlas. *The ICN$_i$ involvement metrics are calculated based on the group-level TC-GICA results, and are ordered according to the percentage of explained variance, similar to the order seen in Figure 2. Noise ICs are marked with an asterisk.*

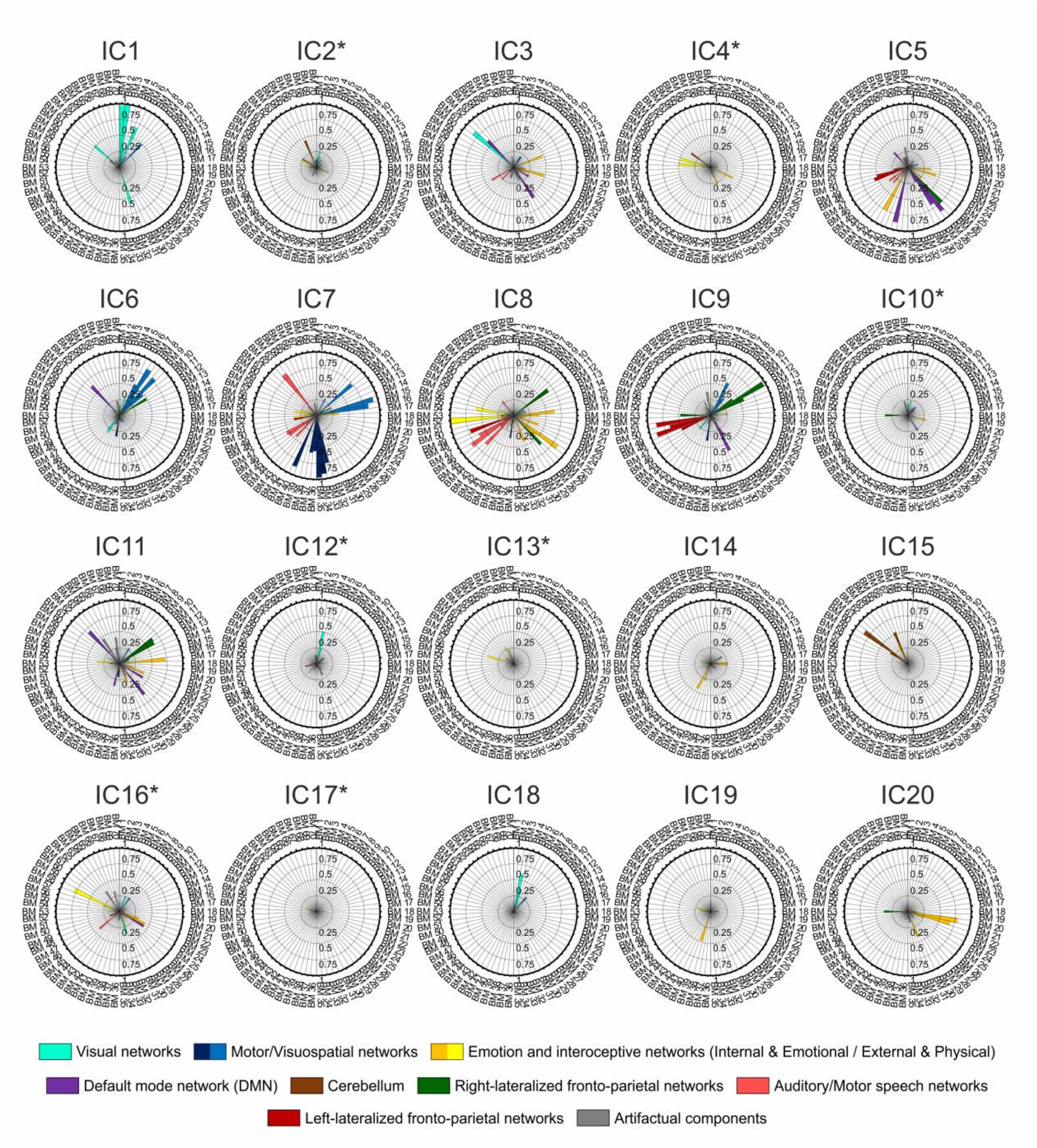

Supplementary figure 3. Comparison of the SMITH10 and the BRAINMAP20 atlas base maps assessed using ICN$_i$ Spatial Involvement (I$_i$). *There is a differential representation of ICNs in the two atlases. Cross-atlasing results are shown, the top panel represents the BRAINMAP20 atlas base maps analysed using the SMITH10 atlas, the bottom panel represents the SMITH10 atlas base maps analysed using the BRAINMAP20 atlas. Circular colour patches drawn left to the atlas base map names represent the cognitive domains best matching the given base map.*

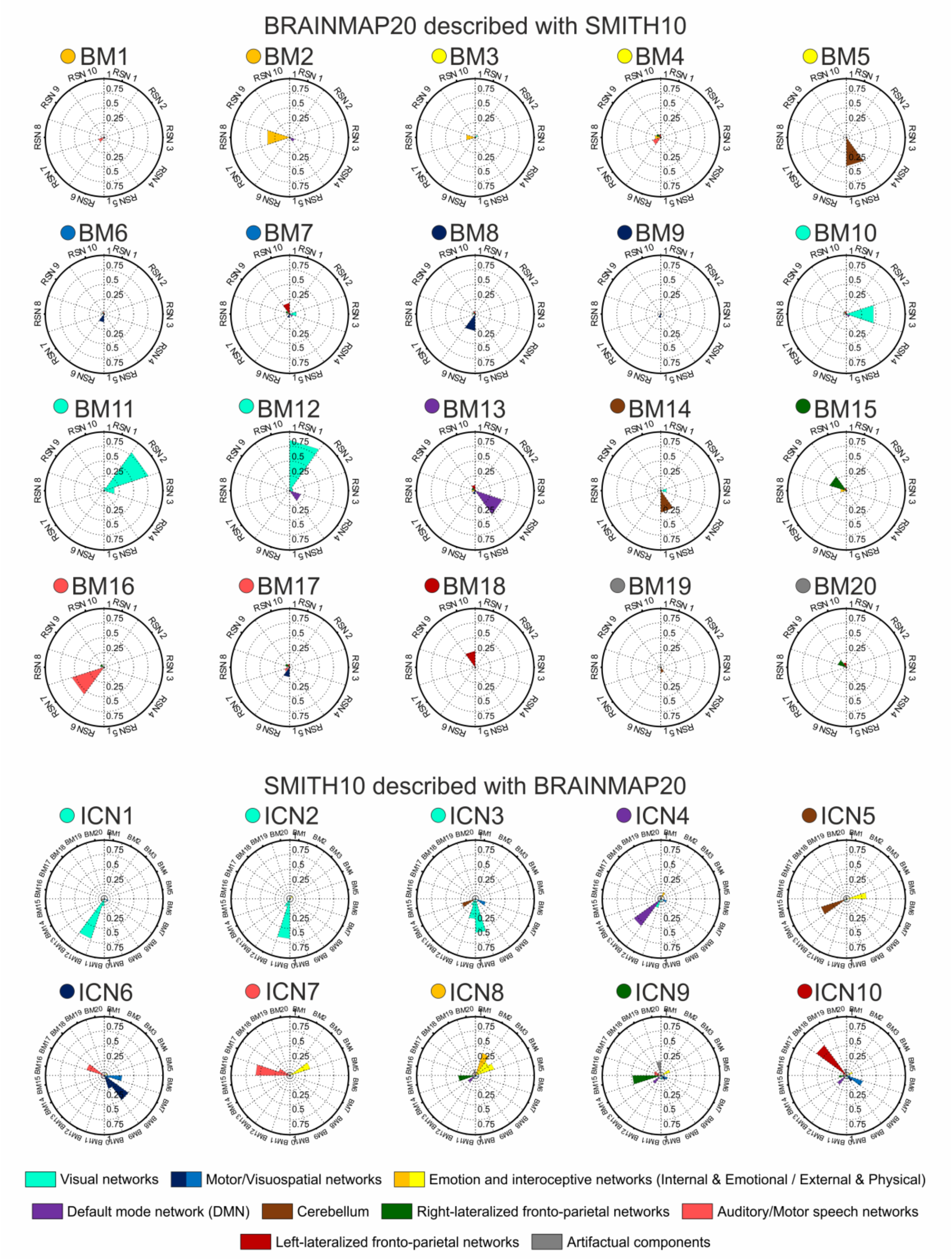

Supplementary Figure 4. Comparison of the BRAINMAP20 and the BRAINMAP70 atlas basemaps assessed using ICN_i Spatial Involvement (I_i). *The results are consistent with sub-network representation of the BRAIMAP20 atlas ICNs (base maps) in the BRAINMAP70 atlas, i.e. the cognitive-domain-based colour coding of the atlasing results match the respective cognitive domains of the BRAINMAP20 atlas base maps. Cross-atlasing results are shown for the BRAINMAP20 atlas base maps analysed using the BRAINMAP70 atlas. Circular colour patches drawn left to the atlas base map names represent the cognitive domains best matching the given base map.*

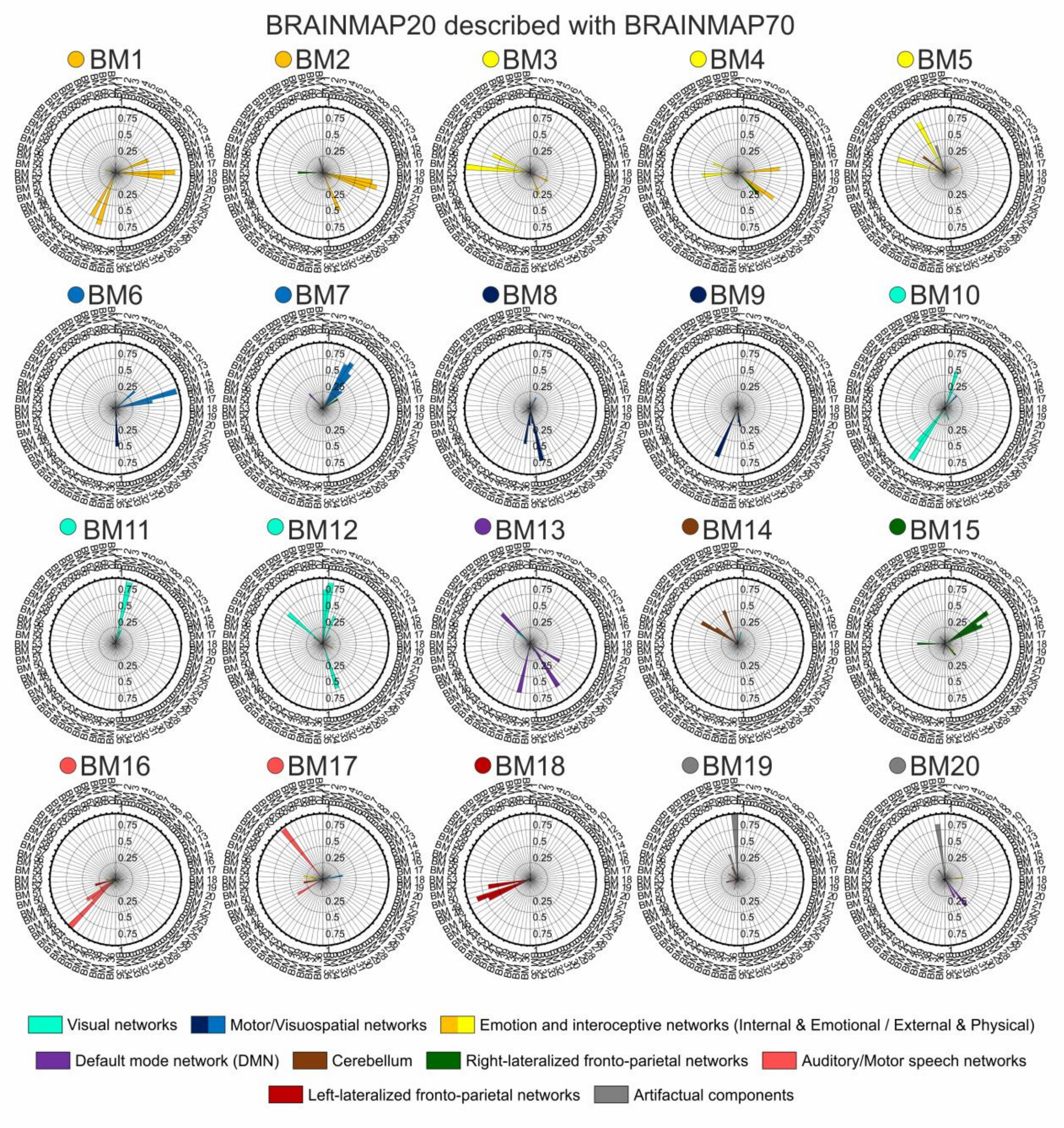

Supplementary Figure 5. Representative examples of the degrees of overlap between ICs and atlas base maps in NYU-TRT group-ICA components with high ICN$_i$ Spatial Involvement (I_i) for the SMITH10 and BRAINMAP20 atlases. *A: pair-wise overlap between IC1 (visual), ICN1, and BM20-12; B: pair-wise overlap between IC3 (DMN), ICN4, and BM20-13; C: pair-wise overlap between IC9 (fronto-parietal), ICN10, and BM20-18.* ***I_i****: ICN$_i$ Spatial Involvement,* ***SQ****: Sørensen-Dice coefficient. Metrics were calculated based on the group-level TC-GICA results (left and middle panels), and by analysing the BRAINMAP20 atlas base maps using the SMITH10 atlas (right panels,* ***I_i*** *values marked by asterisks). Axial, coronal, and sagittal planes match those in Figure 2 for each of the presented ICs. Red, blue, and green patches represent TC-GICA ICs, SMITH10, and BRAINMAP20 atlas base maps, respectively, all thresholded at Z>3; purple, yellow, and cyan patches represent pair-wise overlap.*

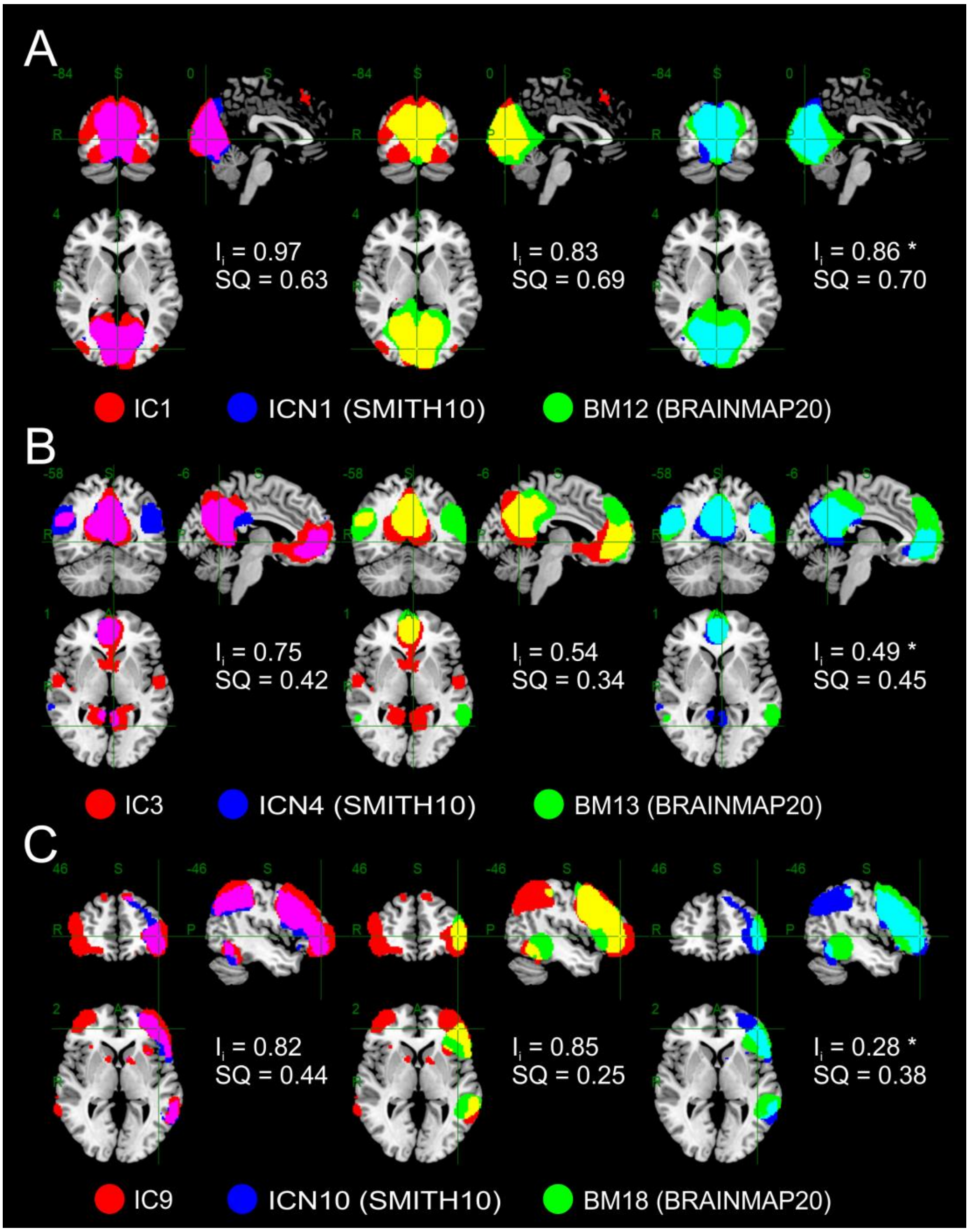

Supplementary Figure 6. Normalised Mean ICN$_i$ Activation ($MA_{N,i}$) for the NYU-TRT group-ICA results for the SMITH10 atlas. *The ICN$_i$ involvement metrics are calculated based on the group-level TC-GICA results, and are ordered according to the percentage of explained variance, similar to the order seen in Figure 2. Noise ICs are marked with an asterisk.*

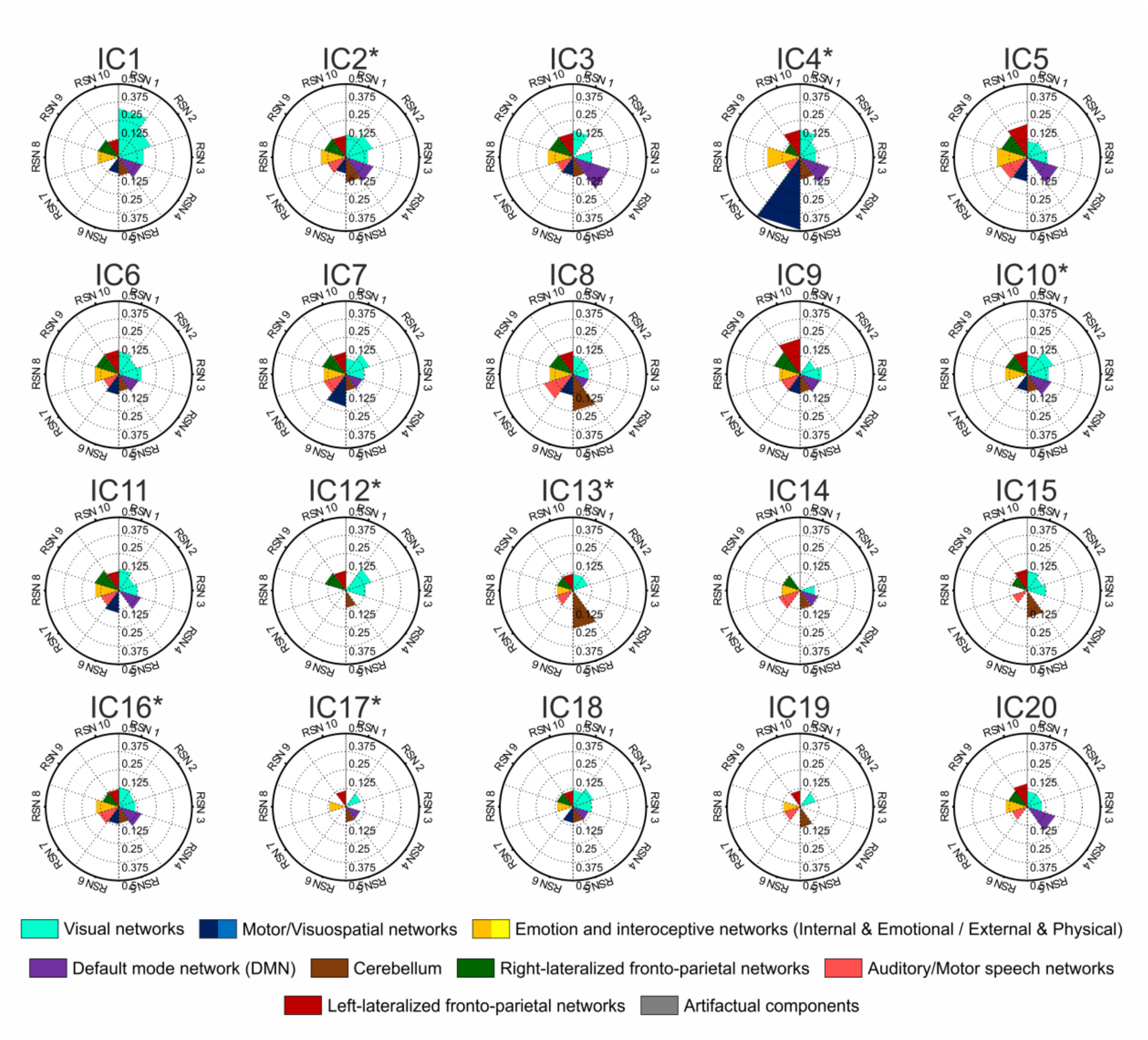

Supplementary Figure 7. Normalised Relative ICN_i Activation ($RA_{N,i}$) for the NYU-TRT group-ICA results for the SMITH10 atlas*. The ICN_i involvement metrics are calculated based on the group-level TC-GICA results, and are ordered according to the percentage of explained variance, similar to the order seen in Figure 2. Noise ICs are marked with an asterisk.*

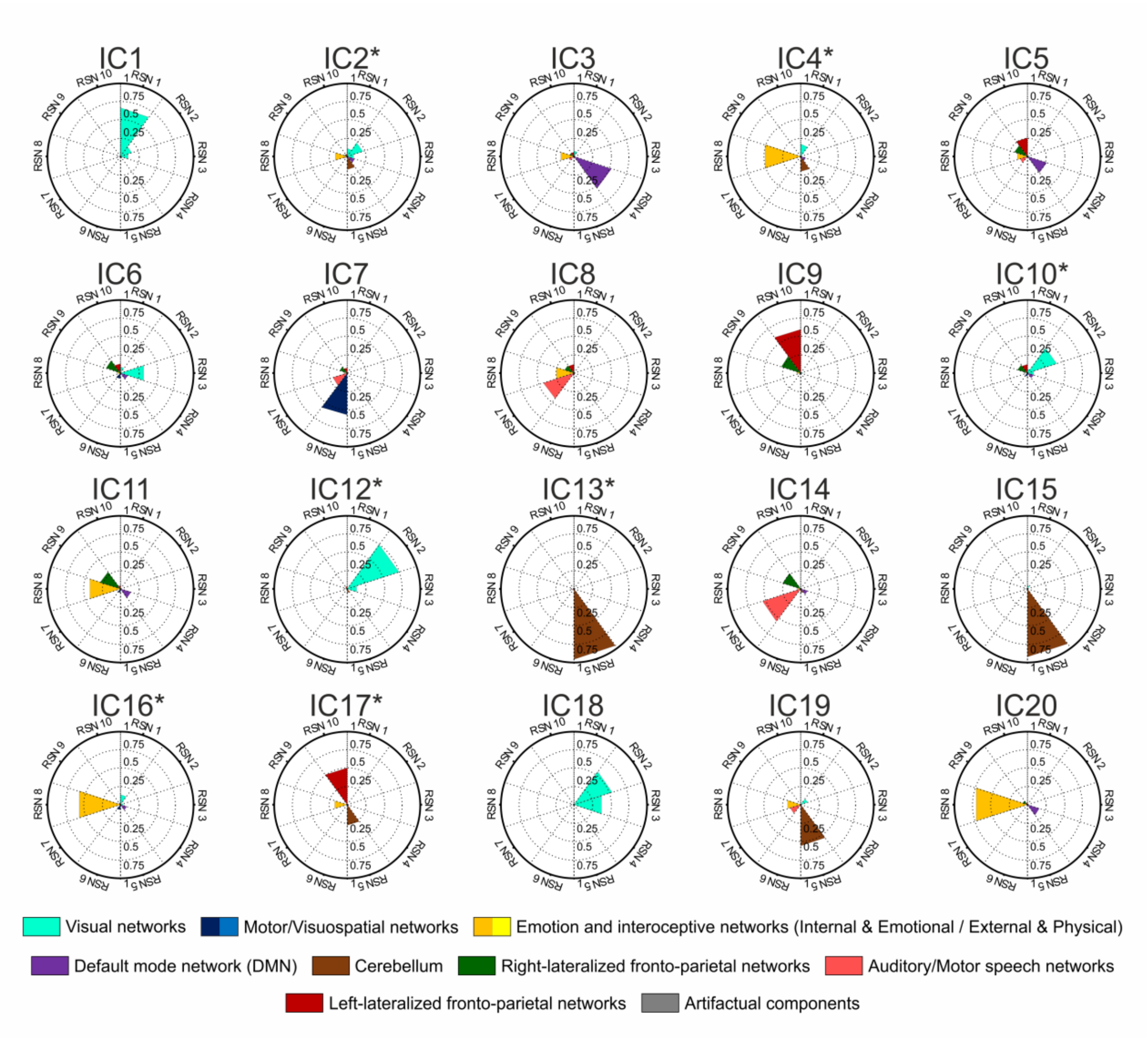

Supplementary Figure 8. Test-retest reliability: within- and between-session intra-class correlation coefficient (ICC) scores. *Parts A-D: within-session; parts E-H: between-session ICC scores using the BRAINMAP70 atlas. ICC scores are shown for every IC and atlas base map combination for the* $\mathbf{I_i}$ *(A, E),* $\mathbf{MA_{N,i}}$ *(B, F), and* $\mathbf{RA_{N,i}}$ *(C, G) metrics. Data collapsed either across ICs or across ICs and atlas base maps are shown in panels D and in the top and bottom ('all') subplots, respectively. The schematic representation of the data collapsing strategy (explained in detail in Methods) is shown in panels G and H: red source and target boxes and red arrows; the panel labels corresponding to the collapsed metrics are marked in the respective subplots (e.g. A, B, C in panel D). Noise ICs are marked by asterisks on all panels.* ICC_W*: within-session ICC,* ICC_B*: between-session ICC. Metric # represents the output metrics as follows: (1)* $\mathbf{I_i}$*, (2)* $\mathbf{IR_i}$*, (3)* $\mathbf{MA_i}$*, (4)* $\mathbf{MA_{N,i}}$*, (5)* $\mathbf{IR_i^M}$*, (6)* $\mathbf{RA_{N,i}}$*, (7)* $\mathbf{I_i^M}$*, (8)* $\mathbf{OL_i}$*, (9)* **SQ***, (10)* $\mathbf{J_i}$*, and (11)* $\mathbf{r_i}$*. See Supplementary Tables 15, 16, 21, 22, 27, 28, 33, and 34 for numerical values.*

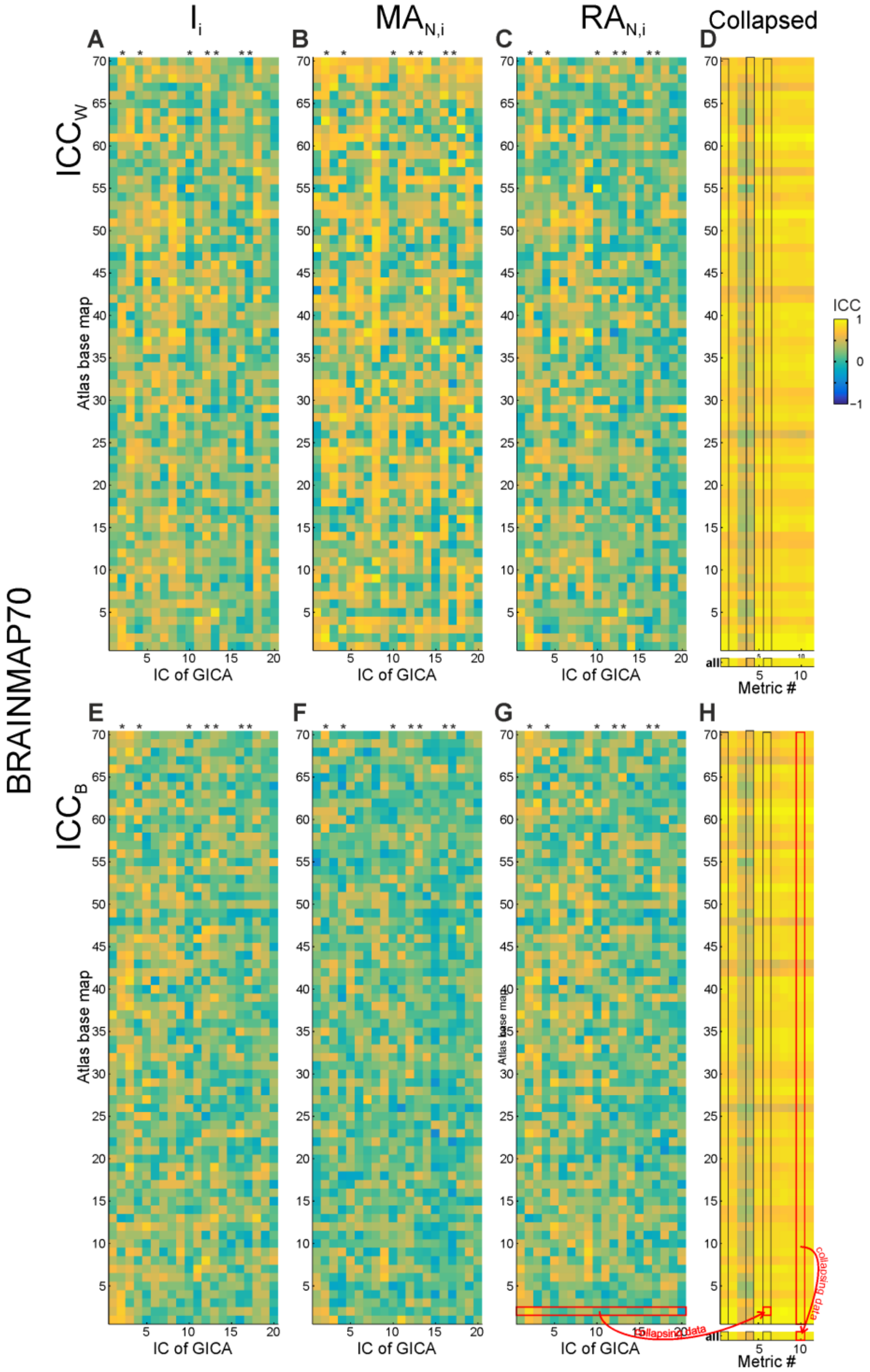

Supplementary Figure 9. Within- and between-session intra-class correlation coefficients for the ICN$_i$ Spatial Involvement (I$_i$) metric scores drawn against mean I$_i$ calculated over sessions of NYU-TRT data. *Negative ICC scores are derived from IC and base atlas map combinations with minimal overlap, as seen from the low **I**$_i$ values. Each data point represents an IC and atlas base map combination, i.e. there are 200 data points for the SMITH10 atlas, 400 data points for the BRAINMAP20 atlas, and 1400 data points for the BRAINMAP70 atlas. Horizontally, the diamonds are located at the mean **I**$_i$ calculated over the dual-regressed single subject ICs from the three sessions, the end points of the horizontal bars represent the means of minimal and maximal **I**$_i$ values averaged over subjects and sessions. Negative ICCs are plotted with red.*

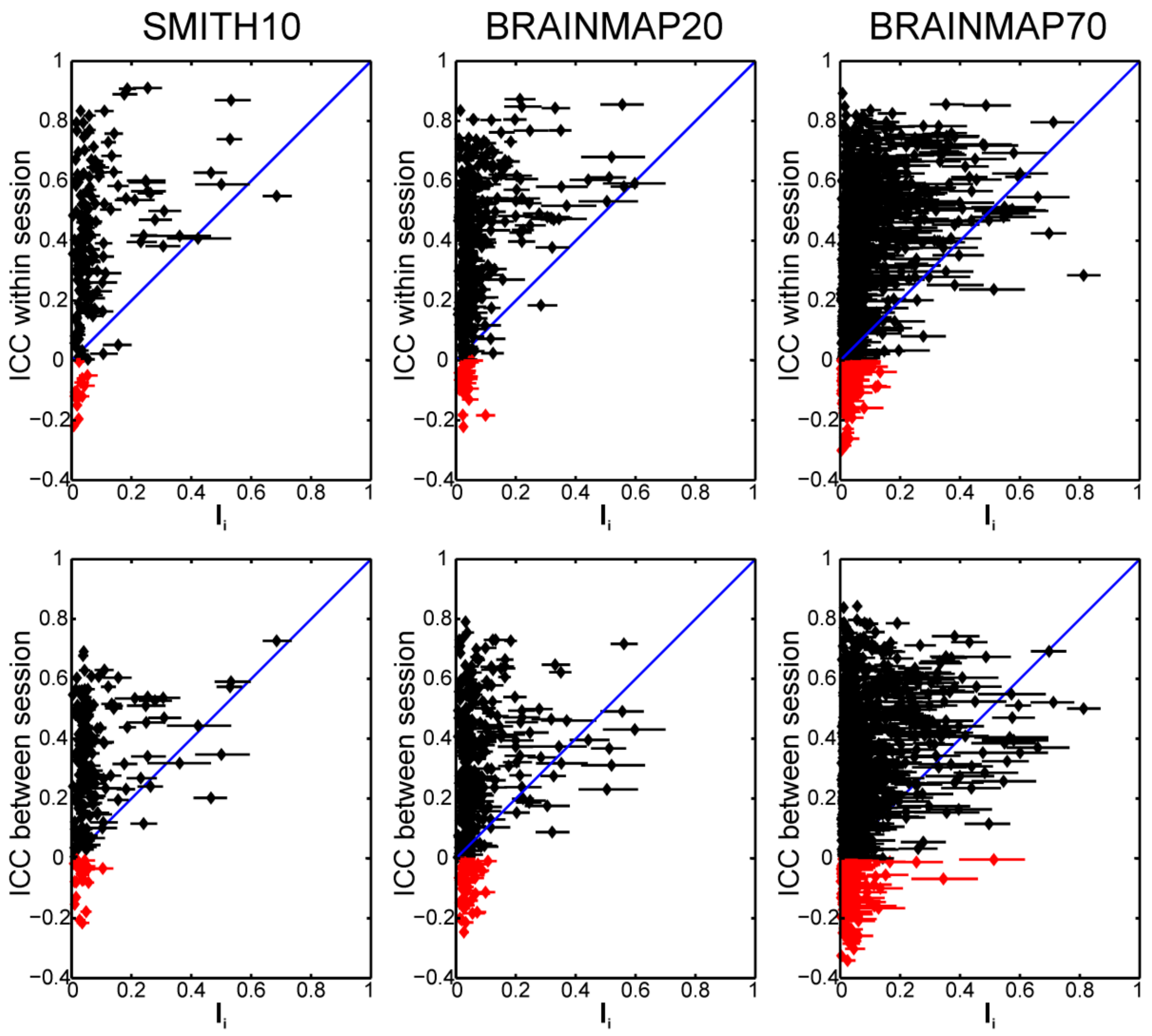

Supplementary Figure 10. Representative examples of the overlap between activation maps and atlas base maps for the parametric modulation task-fMRi experiment. *The changes in the extent of activations follows the 'integrative' phase profile described by Vagharchakian et al. in 2012, i.e. extent of activations is stable or increase slightly for easily understood stimuli (60-100% compression ratio), it peaks for the difficult but intelligible (40% compression ratio) and collapses for the unintelligible (20% compression ratio) stimuli, regardless of stimulus modality (see Figure 10, as well). White outlines represent activation maps thresholded at Z>3, coloured patches represent the BRAINMAP20 base atlas maps coloured according to their respective cognitive domain; minimal hue differences were introduced to be able to separately visualize atlas base maps belonging to the same cognitive domains.*

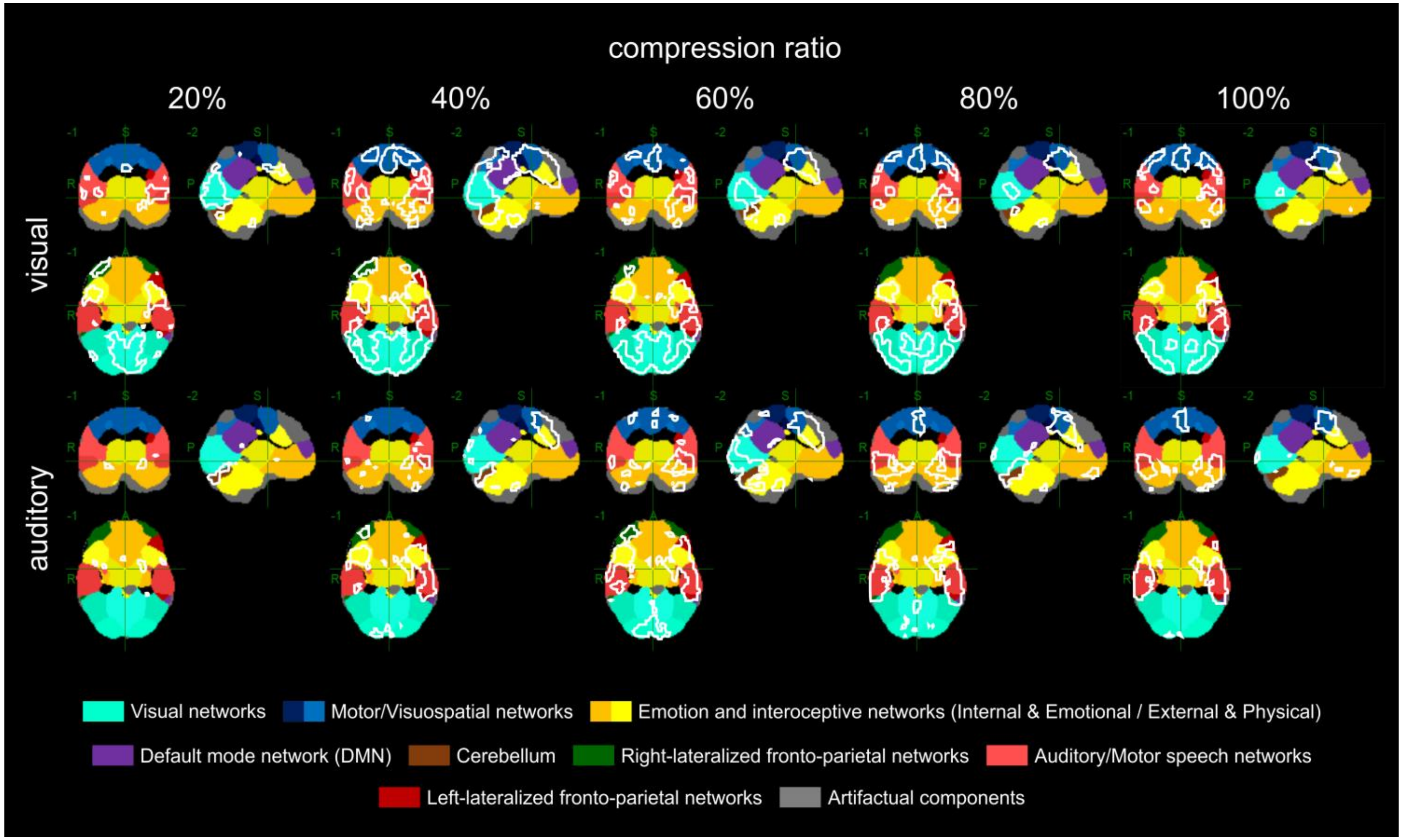

Supplement: Supplementary file 2 [file mmc2.pdf]
