# Supplementary material for: ICN_Atlas: Automated description and quantification of functional MRI activation patterns in the framework of intrinsic connectivity networks
Source: Neuroimage. 2017 Dec;163:319–41. doi: 10.1016/j.neuroimage.2017.09.014 (PMC5725313; doi:10.1016/j.neuroimage.2017.09.014)
Supplement: Supplementary file 3 [file mmc3.pdf]

Supplementary tables

Supplementary Table 1. Functional-anatomical and/or Intrinsic Connectivity Network correspondence of atlas base maps in the BRAINMAP70 atlas.

<table>
<tr><th>ICN #</th><th>Anatomical description</th><th>Intrinsic Connectivity Network cognitive domain</th></tr>
<tr><td>1</td><td>Occipital poles, cuneal cortices, supracalcarine cortices</td><td rowspan="2">Vision (medial occipital)</td></tr>
<tr><td>2</td><td>Lingual gyri, intracalcarine cortices</td></tr>
<tr><td>3</td><td>Occipital poles, occipital fusiform gyri, lateral occipital cortices (inferior division)</td><td>Vision (lateral occipital)</td></tr>
<tr><td>4</td><td>Temporal occipital fusiform cortices, inferior temporal gyri (temporooccipital part), lateral occipital cortices (inferior division)</td><td rowspan="2">Vision (middle and inferior temporal)</td></tr>
<tr><td>5</td><td>Occipital fusiform gyri, intracalcarine cortices</td></tr>
<tr><td>6</td><td>Superior parietal lobules</td><td rowspan="5">Visuospatial</td></tr>
<tr><td>7</td><td>Lateral occipital cortices (superior division), precuneus</td></tr>
<tr><td>8</td><td>Lateral occipital cortices (superior division), superior parietal lobules</td></tr>
<tr><td>9</td><td>Lateral occipital cortices (superior division)</td></tr>
<tr><td>10</td><td>Superior parietal lobules</td></tr>
<tr><td>11</td><td>Frontal poles</td><td rowspan="3">Right lateralized frontoparietal networks (reasoning, attention, inhibition, working memory)</td></tr>
<tr><td>12</td><td>Right angular gyrus, right superior parietal lobule</td></tr>
<tr><td>13</td><td>Right inferior frontal gyrus (pars opercularis, pars triangularis), right middle frontal gyrus, right precentral gyrus</td></tr>
<tr><td>14</td><td>Parahippocampal gyri (posterior division), temporal fusiform cortices (posterior division), hippocampi</td><td>Emotion/Interoceptive (internal/emotional: limbic and mediotemporal)</td></tr>
<tr><td>15</td><td>Supplementary motor cortex</td><td rowspan="2">Visuospatial (premotor, SMA)</td></tr>
<tr><td>16</td><td>Precentral gyri</td></tr>
<tr><td>17</td><td>Paracingulate gyri</td><td rowspan="3">Emotion/Interoceptive (internal/emotional: limbic and mediotemporal)</td></tr>
<tr><td>18</td><td>Amygdalae, pallida</td></tr>
<tr><td>19</td><td>Right middle temporal gyrus (anterior division), right planum polare, right superior temporal gyrus (anterior division)</td></tr>
<tr><td>20</td><td>Subcallosal cortex, frontal orbital cortices, medial frontal cortex</td><td rowspan="3">Emotion/Interoceptive (internal/emotional: subgenual anterior cingulate and orbitofrontal)</td></tr>
<tr><td>21</td><td>Medial frontal cortex, paracingulate gyri, frontal poles</td></tr>
<tr><td>22</td><td>Anterior cingulate gyri, paracingulate gyri</td></tr>
</table>

Supplementary Table 1 continued

**23**	Thalami	Emotion/Interoceptive (internal/emotional: basal ganglia)
**24**	Posterior cingulate gyri	Default mode network
**25**	Left frontal opercular cortex, frontal orbital cortices, insulae	Emotion/Interoceptive (internal/emotional: bilateral anterior insula, frontal opercular cortex)
**26**	Right frontal opercular cortex, right insula, right inferior frontal gyrus (pars triangularis)	
**27**	Right inferior frontal gyrus (pars triangularis)	Right lateralized frontoparietal networks
**28**	Superior frontal gyri, paracingulate gyri, frontal poles	Default mode network
**29**	Left lateral occipital cortex (superior division), left angular gyrus	
**30**	Left superior frontal gyrus, left middle frontal gyrus	
**31**	Accumbens nuclei, caudate nuclei, putamina	Emotion/Interoceptive (internal/emotional: subgenual anterior cingulate and orbitofrontal)
**32**	Lingual gyri, temporal occipital fusiform cortices	Vision – medial occipital
**33**	Left postcentral gyrus, left precentral gyrus, cerebellum (right)	Sensorymotor (hand areas, superior parietal lobule)
**34**	Right postcentral gyrus, right precentral gyrus, right superior parietal lobule	
**35**	Supplementary motor cortex	Sensorymotor (supplementary motor area, premotor area)
**36**	Supplementary motor cortex, anterior cingulate, precentral gyri	
**37**	Supramarginal gyri (anterior and posterior division), postcentral gyri	Sensorymotor (hand areas)
**38**	Left supramarginal gyrus (posterior division), angular gyri	Default mode network
**39**	Parahippocampal gyri (anterior division), temporal fusiform cortices (anterior division), temporal poles	Emotion/Interoceptive (internal/emotional: limbic and mediotemporal)
**40**	Postcentral gyri, precentral gyri	Sensorymotor (foot areas)
**41**	Left middle temporal gyrus (anterior division), left superior temporal gyrus (anterior division), temporal poles, left planum polare	Emotion/Interoceptive (internal/emotional: mediotemporal)
**42**	Lateral occipital cortices (inferior division)	Vision – middle and inferior temporal
**43**	Right inferior temporal gyrus (temporooccipital part), right middle temporal gyrus (temporooccipital part), right inferior temporal gyrus (posterior division), right middle temporal gyrus (posterior division)	
**44**	Superior temporal gyri (posterior division), Heschl's gyri, plana temporale	Auditory

Supplementary Table 1 continued

**45**	Parietal opercular cortices (posterior division), Heschl's gyri, plana temporale	Auditory
**46**	Parietal opercular cortices (posterior division), plana temporale, supramarginal gyri	
**47**	Central opercular cortices, parietal opercular cortices	Speech/language
**48**	Left middle frontal gyrus, left inferior frontal gyrus (pars opercularis and triangularis)	
**49**	Left inferior frontal gyrus (pars triangularis)	
**50**	Left middle temporal gyrus (temporooccipital part and posterior division), left superior temporal gyrus (posterior division), left inferior temporal gyrus (temporooccipital part and posterior division)	
**51**	Left middle frontal gyrus, left inferior frontal gyrus (pars opercularis)	
**52**	Inferior frontal gyri (pars opercularis), frontal opercular cortices, central opercular cortices	Emotion/Interoceptive (external/physical: opercula)
**53**	Frontal poles	Default mode network
**54**	Thalami	Emotion/Interoceptive (external/physical: basal ganglia and thalamus)
**55**	Putamina, insular cortices, pallida	
**56**	Cerebellum, vermis, brainstem	Emotion/Interoceptive (external/physical: midbrain)
**57**	Anterior cingulate gyri	Emotion/Interoceptive (external/physical: anterior cingulate)
**58**	Caudate nuclei	Emotion/Interoceptive (external/physical: basal ganglia)
**59**	Cerebellum	Cerebellum
**60**	Vermis, Cerebellum	
**61**	Supracalcarine cortices, intracalcarine cortices, precuneus	Vision (medial occipital)
**62**	Precuneus	DMN
**63**	Postcentral gyri, precentral gyri	Speech/language (motor components)
**64**	Artefactual component	N/A
**65**	Cerebellum, vermis	Cerebellum
**66**	Cerebellum, vermis	
**67**	Artefactual component	N/A
**68**	Artefactual component	
**69**	Artefactual component	
**70**	Artefactual component	

Supplementary Table 2. ICN$_i$ Spatial Involvement (I$_i$) values for the NYU-TRT group ICA data atlased using the SMITH10 atlas. *Bold typeface indicates the highest 3 values for a given IC, note that due to data presentation using two significant digits minor differences are not shown in the table, but the markings are based on 8 digits precision data. The last row shows the contribution of the 3 highest values for a given IC in percentage of the sum. ICN01 visual (medial), ICN02 visual (occipital pole), ICN03 visual (lateral), ICN04 DMN, ICN05 cerebellum, ICN06 sensori-motor, ICN07 auditory, ICN08 executive control, ICN09 fronto-parietal (perception, somesthesis, pain), ICN10 fronto-parietal (cognition-language).*

	Component ICs													*Noise ICs*					
	IC01	IC03	IC05	IC06	IC07	IC08	IC09	IC11	IC14	IC15	IC18	IC19	IC20	IC02	IC04	IC10	IC12	IC13	IC16	IC17
ICN01	**0.97**	0.16	0.00	0.13	0.00	0.04	0.00	0.04	0.00	**0.04**	0.01	0.00	0.00	**0.07**	**0.07**	0.03	0.00	0.00	**0.06**	0.00
ICN02	**0.47**	0.00	0.01	0.01	0.02	0.01	0.00	0.02	0.00	0.00	**0.61**	**0.03**	0.00	**0.15**	0.01	**0.20**	**0.39**	0.00	0.00	0.00
ICN03	**0.27**	0.01	0.05	**0.45**	0.01	0.02	0.08	0.00	0.00	**0.01**	**0.32**	0.00	0.00	0.05	0.00	0.04	**0.06**	0.00	0.02	0.00
ICN04	0.09	**0.75**	**0.47**	**0.19**	0.01	0.02	0.05	**0.36**	**0.02**	0.00	**0.01**	0.00	**0.08**	0.05	0.03	0.04	0.00	0.00	**0.03**	0.00
ICN05	0.05	0.01	0.00	0.01	0.01	0.00	0.01	0.00	0.01	**0.64**	0.00	**0.09**	0.00	**0.10**	**0.11**	0.01	**0.04**	**0.38**	0.01	**0.00**
ICN06	0.00	0.02	0.02	0.10	**0.84**	0.09	0.05	0.09	0.00	0.00	0.00	0.00	0.00	0.00	0.00	0.02	0.00	0.00	0.02	0.00
ICN07	0.00	**0.18**	0.17	0.00	**0.52**	**0.87**	0.01	0.01	**0.09**	0.00	0.00	**0.02**	0.00	0.02	0.00	0.00	0.00	**0.02**	0.00	0.00
ICN08	0.01	**0.22**	0.13	0.03	0.04	**0.44**	**0.08**	**0.56**	0.00	0.00	0.01	0.02	**0.24**	0.05	**0.10**	0.00	0.00	0.00	**0.12**	**0.00**
ICN09	0.00	0.08	**0.25**	**0.21**	**0.20**	**0.27**	**0.48**	**0.43**	**0.04**	0.00	0.01	0.00	**0.03**	0.01	0.00	**0.04**	0.01	**0.00**	0.00	0.00
ICN10	0.01	0.09	**0.30**	0.14	0.15	0.27	**0.82**	0.09	0.00	0.00	0.00	0.00	0.01	0.01	0.00	**0.04**	0.01	0.00	0.01	**0.00**
Top 3 in % of sum	91%	76%	73%	68%	86%	78%	87%	85%	90%	99%	97%	87%	96%	61%	84%	69%	96%	98%	76%	94%

Supplementary table 3. ICN$_i$ Spatial Involvement (I_i) values for the NYU-TRT group ICA data atlased using the BRAINMAP20 atlas. *Bold typeface indicates the highest 3 values for a given IC, note that due to data presentation using two significant digits minor differences are not shown in the table, but the markings are based on 8 digits precision data. The last row shows the contribution of the 3 highest values for a given IC in percentage of the sum. BM1: limbic and medial-temporal areas, BM2: subgenual ACC and OFC, BM3: bilateral BG and thalamus, BM4: bilateral anterior insula/frontal opercula and the anterior aspect of the body of the cingulate gyrus, BM5: midbrain, BM6: superior and middle frontal gyri, BM7: middle frontal gyri and superior parietal lobules, BM8: ventral precentral gyri, central sulci, postcentral gyri, superior and inferior cerebellum, BM9: superior parietal lobule, BM10: middle and inferior temporal gyri, BM11: lateral posterior occipital cortex, BM12: medial posterior occipital cortex, BM13: medial prefrontal and posterior cingulate/precuneus areas, DMN, BM14: cerebellum, BM15: right-lateralized fronto-parietal regions, BM16: transverse temporal gyri, BM17: dorsal precentral gyri, central sulci, postcentral gyri, superior and inferior cerebellum, BM18: left-lateralized fronto-parietal regions, BM19-20: artefactual.*

	Component ICs													*Noise ICs*						
	IC01	IC03	IC05	IC06	IC07	IC08	IC09	IC11	IC14	IC15	IC18	IC19	IC20	IC02	IC04	IC10	IC12	IC13	IC16	IC17
BM01	0.00	0.14	0.25	0.01	0.02	0.19	0.01	0.00	**0.30**	0.00	0.00	**0.18**	**0.12**	0.02	0.07	0.00	0.06	0.03	0.03	**0.03**
BM02	0.00	**0.28**	0.15	0.00	0.00	0.25	0.06	0.18	0.00	0.00	0.02	0.00	**0.60**	0.04	0.03	**0.12**	0.00	0.00	**0.14**	0.01
BM03	0.00	0.00	0.00	0.00	0.14	0.18	0.04	0.29	0.00	0.00	0.00	**0.12**	0.00	**0.12**	**0.31**	0.00	0.00	0.00	**0.29**	0.00
BM04	0.00	0.00	0.20	0.01	0.17	**0.88**	0.07	0.26	**0.07**	0.00	0.00	0.01	0.02	0.06	0.01	0.00	0.00	0.01	0.00	0.00
BM05	0.00	0.05	0.01	0.03	0.00	0.00	0.01	0.01	0.04	**0.32**	0.00	0.06	0.00	0.01	**0.21**	0.00	0.03	**0.32**	0.00	**0.05**
BM06	0.01	0.00	0.08	0.03	**0.88**	0.07	0.14	0.06	0.00	0.00	0.00	0.00	0.01	0.03	0.00	0.03	0.04	0.00	0.08	0.00
BM07	**0.11**	0.14	0.02	**0.75**	0.13	0.00	0.40	0.16	0.00	0.00	**0.05**	0.00	0.00	0.03	0.00	0.03	0.00	0.00	0.03	0.00
BM08	0.00	0.00	0.01	0.13	**0.82**	0.15	0.15	0.03	0.00	0.03	0.00	0.00	0.06	0.00	0.02	0.01	0.01	0.00	0.01	0.00
BM09	0.00	0.01	0.00	0.14	**0.93**	0.00	0.00	0.02	0.00	0.00	0.00	0.00	0.02	0.11	0.00	0.06	0.01	0.00	0.02	0.00
BM10	0.10	0.02	0.10	**0.34**	0.02	0.07	0.11	0.00	0.00	0.00	**0.13**	0.00	0.00	0.01	0.00	0.04	0.08	0.00	**0.13**	0.00
BM11	**0.34**	0.00	0.02	0.05	0.00	0.00	0.01	0.00	0.00	0.01	**0.55**	0.00	0.00	**0.28**	0.00	**0.19**	**0.38**	0.00	0.01	0.00
BM12	**0.83**	**0.26**	0.01	0.12	0.01	0.04	0.00	0.02	0.00	**0.04**	0.04	0.00	0.00	0.08	**0.11**	0.05	0.00	0.01	0.11	0.00
BM13	0.00	**0.54**	**0.58**	0.15	0.06	0.06	0.12	**0.46**	0.00	0.00	0.00	0.00	0.02	0.03	0.00	0.04	0.01	0.00	0.05	0.00
BM14	0.10	0.00	0.05	0.02	0.01	0.00	0.15	0.00	0.00	**0.62**	0.03	0.00	0.00	**0.29**	0.01	0.01	0.05	0.00	0.01	0.00
BM15	0.00	0.01	0.07	0.09	0.04	0.36	**0.63**	**0.48**	0.02	0.00	0.00	0.00	0.10	0.01	0.00	**0.14**	0.05	0.00	0.00	0.00
BM16	0.00	0.18	**0.37**	0.00	0.38	**0.56**	0.07	0.01	**0.09**	0.00	0.00	0.00	0.00	0.01	0.00	0.00	0.00	0.01	0.02	0.00
BM17	0.00	0.04	0.00	0.01	0.71	0.39	0.05	0.00	0.00	0.00	0.00	0.01	0.00	0.00	0.00	0.00	0.00	0.00	0.02	0.00
BM18	0.04	0.01	0.36	0.00	0.04	**0.39**	**0.85**	0.02	0.00	0.00	0.00	0.00	0.01	0.01	0.00	0.01	0.07	0.01	0.02	0.00
BM19	0.01	0.00	0.16	0.03	0.01	0.00	0.07	0.00	0.01	0.01	0.00	**0.10**	**0.10**	0.09	0.00	0.06	**0.11**	**0.06**	0.01	**0.01**
BM20	0.05	0.14	**0.43**	**0.16**	0.18	0.03	**0.43**	**0.49**	0.02	0.00	0.00	0.01	0.01	0.08	0.03	0.11	**0.12**	**0.05**	0.01	0.00
Top 3 in % of sum	80%	59%	48%	60%	58%	50%	57%	57%	82%	95%	90%	83%	75%	53%	78%	50%	58%	85%	57%	81%

Supplementary table 4. ICN$_i$ Spatial Involvement (I_i) values for the NYU-TRT group ICA data atlased using the BRAINMAP70 atlas. *Bold typeface indicates the highest 3 values for a given IC, note that due to data presentation using two significant digits minor differences are not shown in the table, but the markings are based on 8 digits precision data. The last row shows the contribution of the 3 highest values for a given IC in percentage of the sum. See Supplementary Table 1 for the description of the atlas ICNs (BM01-BM70).*

	Component ICs													*Noise ICs*						
	IC01	IC03	IC05	IC06	IC07	IC08	IC09	IC11	IC14	IC15	IC18	IC19	IC20	IC02	IC04	IC10	IC12	IC13	IC16	IC17
BM01	**0.97**	0.03	0.01	0.15	0.00	0.02	0.00	0.02	0.00	0.00	0.10	0.00	0.00	0.12	0.00	0.18	0.01	0.03	0.00	0.00
BM02	**0.98**	0.00	0.00	0.00	0.01	0.00	0.00	0.00	0.00	0.07	0.00	0.00	0.00	0.13	0.02	0.09	0.00	0.00	0.06	0.00
BM03	0.29	0.00	0.01	0.01	0.00	0.00	0.00	0.00	0.00	0.00	**0.61**	0.00	0.00	**0.25**	0.00	0.23	**0.51**	0.00	0.00	0.00
BM04	0.15	0.00	0.01	0.29	0.01	0.00	0.15	0.00	0.00	0.02	**0.37**	0.00	0.00	0.17	0.00	0.02	**0.25**	0.00	0.00	0.00
BM05	**0.68**	0.00	0.00	0.01	0.00	0.00	0.00	0.00	0.00	0.00	0.16	0.00	0.00	0.00	0.00	0.00	0.00	0.00	0.26	0.00
BM06	0.00	0.00	0.01	0.55	0.30	0.00	0.58	0.02	0.00	0.00	0.04	0.00	0.00	0.00	0.00	0.00	0.00	0.00	0.04	0.00
BM07	0.19	0.20	0.03	**0.85**	0.01	0.00	0.32	0.29	0.00	0.00	0.01	0.00	0.00	0.14	0.00	0.14	0.00	0.02	0.00	0.01
BM08	0.03	0.20	0.05	0.38	0.00	0.00	0.33	0.14	0.00	0.00	0.03	0.00	0.00	0.03	0.00	0.00	0.00	0.00	0.27	0.00
BM09	0.50	0.06	0.13	**0.80**	0.00	0.00	0.08	0.01	0.00	0.00	**0.30**	0.00	0.00	0.00	0.00	0.18	0.00	0.00	0.03	0.00
BM10	0.00	0.00	0.00	0.49	0.73	0.00	0.16	0.03	0.00	0.00	0.02	0.00	0.01	0.01	0.00	0.00	0.06	0.00	0.08	0.00
BM11	0.00	0.00	0.05	0.04	0.01	0.67	0.42	**0.64**	0.00	0.00	0.00	0.00	0.02	0.02	0.00	0.14	0.15	0.01	0.00	0.00
BM12	0.00	0.01	0.00	0.50	0.14	0.00	**0.96**	0.63	0.21	0.00	0.01	0.00	0.00	0.00	0.00	0.05	0.03	0.00	0.01	0.00
BM13	0.00	0.00	0.08	0.08	0.25	0.20	0.57	0.19	0.00	0.00	0.00	0.00	0.00	0.00	0.02	0.00	0.04	0.01	0.02	0.00
BM14	0.00	0.49	0.00	0.15	0.03	0.07	0.00	0.00	0.06	0.01	0.00	0.03	0.07	0.09	0.10	0.00	0.12	0.00	0.13	0.01
BM15	0.00	0.00	0.17	0.00	**0.93**	0.20	0.04	0.16	0.00	0.00	0.00	0.00	0.00	0.04	0.00	0.04	0.00	0.00	0.06	0.00
BM16	0.03	0.00	0.07	0.00	0.83	0.00	0.12	0.01	0.00	0.00	0.00	0.00	0.00	0.00	0.00	0.00	0.06	0.00	0.04	0.00
BM17	0.02	0.00	0.01	0.00	0.35	0.65	0.26	**0.74**	0.00	0.00	0.00	0.00	0.00	0.06	0.00	0.00	0.00	0.00	0.01	0.00
BM18	0.00	0.07	0.00	0.00	0.00	0.41	0.00	0.00	**0.27**	0.00	0.00	0.12	0.14	0.00	0.10	0.00	0.00	0.00	0.00	**0.12**
BM19	0.00	0.18	0.37	0.01	0.04	0.22	0.02	0.01	0.26	0.00	0.00	0.10	0.17	0.02	0.00	0.00	0.11	0.01	0.08	0.00
BM20	0.00	0.17	0.06	0.00	0.00	0.07	0.04	0.00	0.01	0.00	0.00	0.00	**0.79**	0.00	0.00	0.00	0.00	0.00	0.01	0.00
BM21	0.00	0.50	0.47	0.00	0.00	0.16	0.00	0.24	0.00	0.00	0.06	0.00	**0.69**	0.00	0.00	**0.29**	0.00	0.00	0.00	0.00
BM22	0.00	0.24	0.07	0.00	0.00	0.42	0.00	0.42	0.00	0.00	0.00	0.00	0.27	0.11	0.00	0.00	0.00	0.00	0.33	0.00
BM23	0.03	0.16	0.00	0.01	0.02	0.02	0.00	0.01	0.00	0.00	0.00	0.02	0.00	0.22	**0.38**	0.00	0.00	0.04	**0.44**	0.00
BM24	0.00	0.28	0.08	0.02	0.02	0.01	0.06	0.43	0.00	0.00	0.00	0.00	0.00	0.04	0.00	0.00	0.00	0.04	**0.45**	0.00
BM25	0.00	0.00	0.27	0.00	0.00	**0.85**	0.15	0.15	0.10	0.00	0.00	0.00	0.07	0.01	0.01	0.00	0.00	0.01	0.03	0.00
BM26	0.00	0.00	0.00	0.00	0.02	0.48	0.09	0.09	0.00	0.00	0.00	0.00	0.01	0.05	0.00	0.00	0.00	0.00	0.15	0.00
BM27	0.00	0.00	**0.76**	0.00	0.04	0.63	0.09	0.14	0.11	0.01	0.00	0.00	0.13	0.04	0.01	0.00	0.00	0.00	0.00	0.00

Supplementary Table 4 continued

	Component ICs													*Noise ICs*					
	IC01	IC03	IC05	IC06	IC07	IC08	IC09	IC11	IC14	IC15	IC18	IC19	IC20	IC02	IC04	IC10	IC12	IC13	IC16	IC17
BM28	0.02	0.13	**0.87**	0.00	0.08	0.10	0.16	0.63	0.00	0.00	0.00	0.00	0.02	0.00	0.00	0.05	0.13	0.06	0.01	0.00
BM29	0.00	**0.57**	0.71	0.12	0.00	0.00	0.38	0.03	0.00	0.00	0.00	0.00	0.00	0.01	0.00	**0.29**	0.00	0.00	0.04	0.02
BM30	0.03	0.23	0.26	0.00	0.12	0.01	0.63	0.23	0.00	0.00	0.00	0.00	0.00	0.00	0.00	0.00	**0.21**	0.00	0.05	0.00
BM31	0.00	0.17	0.00	0.00	0.02	0.44	0.07	0.33	0.00	0.00	0.00	0.05	**0.43**	0.05	0.29	0.00	0.00	0.00	0.26	**0.03**
BM32	0.59	0.23	0.00	0.11	0.00	0.01	0.00	0.00	0.00	0.00	0.00	0.00	0.00	0.01	0.16	0.00	0.00	0.00	0.37	0.00
BM33	0.02	0.00	0.00	0.01	0.77	0.00	0.09	0.00	0.01	0.15	0.00	0.00	0.17	0.00	0.10	0.00	0.06	0.04	0.00	0.00
BM34	0.00	0.03	0.00	0.08	**0.92**	0.00	0.04	0.00	0.00	0.02	0.00	0.00	0.11	0.00	0.01	0.04	0.02	0.00	0.00	0.00
BM35	0.00	0.01	0.01	0.00	**0.98**	0.00	0.00	0.00	0.00	0.00	0.00	0.00	0.03	0.10	0.01	0.11	0.07	0.00	0.08	0.00
BM36	0.00	0.01	0.07	0.03	0.57	0.12	0.00	0.21	0.00	0.00	0.00	0.00	0.00	0.00	0.00	0.00	0.00	0.00	0.14	0.00
BM37	0.00	0.00	0.00	0.34	0.60	0.36	0.40	0.19	0.00	0.00	0.00	0.00	0.00	0.00	0.00	0.00	0.00	0.00	0.00	0.00
BM38	0.00	0.17	**0.89**	0.13	0.04	0.17	0.13	0.36	0.00	0.00	0.00	0.00	0.00	0.00	0.00	0.02	0.00	0.00	0.00	0.00
BM39	0.00	0.05	0.15	0.00	0.00	0.02	0.00	0.00	**0.28**	0.00	0.00	**0.48**	0.13	0.00	0.00	0.00	0.11	0.03	0.02	0.01
BM40	0.00	0.00	0.00	0.15	0.88	0.00	0.00	0.02	0.00	0.00	0.00	0.00	0.03	0.14	0.00	0.05	0.01	0.00	0.01	0.00
BM41	0.00	0.09	0.76	0.00	0.06	0.23	0.00	0.00	**0.44**	0.00	0.00	0.02	0.03	0.04	0.00	0.01	0.00	0.08	0.00	0.00
BM42	0.06	0.01	0.17	0.32	0.03	0.10	0.02	0.00	0.00	0.00	0.06	0.00	0.00	0.00	0.00	0.07	0.03	0.00	0.08	0.00
BM43	0.00	0.01	0.12	0.29	0.03	0.08	0.26	0.00	0.00	0.00	0.01	0.00	0.09	0.02	0.00	0.00	0.19	0.00	0.11	0.00
BM44	0.00	0.26	0.33	0.00	0.46	0.62	0.02	0.01	0.10	0.01	0.00	0.00	0.00	0.01	0.00	0.00	0.00	0.02	0.00	0.00
BM45	0.00	0.09	0.12	0.01	0.21	0.26	0.00	0.00	0.00	0.00	0.00	0.00	0.00	0.03	0.00	0.00	0.00	0.02	0.41	0.00
BM46	0.00	0.01	0.35	0.08	0.53	**0.80**	0.01	0.05	0.00	0.00	0.00	0.00	0.00	0.00	0.00	0.00	0.00	0.00	0.00	0.00
BM47	0.00	0.39	0.00	0.00	0.55	0.62	0.00	0.00	0.00	0.00	0.00	0.00	0.00	0.00	0.00	0.00	0.00	0.01	0.05	0.00
BM48	0.00	0.00	0.10	0.00	0.00	0.17	0.50	0.06	0.00	0.00	0.00	0.00	0.00	0.01	0.00	0.00	0.00	0.00	0.16	0.00
BM49	0.03	0.00	0.56	0.00	0.00	0.72	**0.89**	0.00	0.00	0.00	0.00	0.00	0.03	0.00	0.00	0.02	0.02	0.00	0.00	0.00
BM50	0.00	0.01	0.51	0.01	0.00	0.02	0.66	0.00	0.03	0.00	0.00	0.00	0.11	0.00	0.00	0.00	0.18	0.00	0.01	0.00
BM51	0.04	0.00	0.23	0.00	0.35	0.21	**0.86**	0.00	0.00	0.00	0.00	0.00	0.00	0.00	0.00	0.00	0.04	0.03	0.00	0.00
BM52	0.00	0.00	0.16	0.00	0.34	**0.98**	0.11	0.03	0.08	0.00	0.00	0.00	0.00	0.09	0.00	0.00	0.00	0.02	0.00	0.00
BM53	0.00	0.00	0.04	0.00	0.00	0.20	0.48	0.15	0.00	0.00	0.00	0.00	0.37	0.01	0.00	**0.35**	0.00	0.00	0.00	0.00
BM54	0.00	0.01	0.00	0.00	0.18	0.09	0.01	0.34	0.00	0.00	0.00	**0.18**	0.00	0.17	**0.50**	0.00	0.01	0.00	0.20	0.01
BM55	0.00	0.02	0.00	0.00	0.33	0.59	0.00	0.05	0.02	0.00	0.00	0.15	0.00	0.00	0.00	0.00	0.00	0.00	0.01	0.00
BM56	0.00	0.01	0.00	0.00	0.00	0.00	0.00	0.01	0.15	0.12	0.00	**0.22**	0.00	0.01	**0.48**	0.00	0.00	**0.42**	0.00	**0.17**
BM57	0.00	0.00	0.00	0.00	0.25	0.23	0.07	0.11	0.00	0.00	0.00	0.00	0.00	0.03	0.00	0.00	0.00	0.00	0.27	0.00

Supplementary Table 4 continued

	Component ICs													*Noise ICs*						
	IC01	IC03	IC05	IC06	IC07	IC08	IC09	IC11	IC14	IC15	IC18	IC19	IC20	IC02	IC04	IC10	IC12	IC13	IC16	IC17
BM58	0.00	0.00	0.01	0.00	0.08	0.01	0.04	0.21	0.00	0.00	0.00	0.00	0.00	**0.26**	0.15	0.00	0.00	0.00	**0.78**	0.00
BM59	0.00	0.00	0.00	0.01	0.01	0.01	0.08	0.00	0.00	**0.31**	0.00	0.00	0.00	0.25	0.00	0.00	0.03	0.01	0.00	0.00
BM60	0.18	0.01	0.00	0.00	0.05	0.00	0.00	0.00	0.00	**0.83**	0.00	0.00	0.00	0.04	0.37	0.02	0.06	0.14	0.04	0.01
BM61	0.51	**0.82**	0.11	0.12	0.02	0.09	0.00	0.15	0.00	0.00	0.00	0.00	0.00	0.07	0.05	0.01	0.00	0.00	0.08	0.00
BM62	0.00	**0.58**	0.31	**0.63**	0.19	0.01	0.01	**0.69**	0.00	0.00	0.00	0.00	0.00	0.01	0.00	0.00	0.00	0.00	0.00	0.00
BM63	0.00	0.04	0.00	0.01	0.81	0.28	0.03	0.00	0.00	0.00	0.00	0.00	0.00	0.00	0.00	0.00	0.00	0.00	0.01	0.00
BM64	0.00	0.09	0.04	0.08	0.02	0.00	0.15	0.47	0.00	0.00	0.00	0.00	0.00	0.03	0.00	0.00	0.01	0.00	0.38	0.00
BM65	0.00	0.01	0.01	0.01	0.00	0.00	0.01	0.00	0.00	0.31	0.00	0.01	0.00	0.00	0.00	0.00	0.02	**0.28**	0.00	0.00
BM66	0.11	0.00	0.16	0.00	0.00	0.00	0.22	0.00	0.00	**0.52**	0.04	0.00	0.00	**0.45**	0.00	0.04	0.02	0.00	0.02	0.00
BM67	0.00	0.01	0.00	0.04	0.01	0.00	0.00	0.00	0.01	0.08	0.00	0.03	0.04	0.06	0.00	0.00	0.06	**0.23**	0.00	0.00
BM68	0.02	0.10	0.16	0.00	0.05	0.07	0.11	0.25	0.00	0.00	0.00	0.00	0.10	0.05	0.00	0.22	0.03	0.00	0.32	0.00
BM69	0.05	0.17	0.31	0.23	0.20	0.02	0.37	0.41	0.04	0.00	0.00	0.01	0.02	0.10	0.05	0.17	0.14	0.05	0.01	0.00
BM70	0.00	0.00	0.15	0.01	0.01	0.00	0.04	0.00	0.01	0.00	0.00	0.12	0.11	0.08	0.00	0.04	0.13	0.06	0.00	0.02
Top 3 in % of sum	48%	29%	24%	32%	21%	21%	24%	22%	45%	68%	70%	57%	46%	26%	48%	32%	33%	56%	26%	80%

Supplementary Table 5. Normalised Mean ICN$_i$ Activation (MA$_{N,i}$) values for the NYU-TRT group ICA data atlased using the SMITH10 atlas. *Bold typeface indicates the highest 3 values for a given IC, note that due to data presentation using two significant digits minor differences are not shown in the table, but the markings are based on 8 digits precision data. The last row shows the contribution of the 3 highest values for a given IC in percentage of the sum. ICN01 visual (medial), ICN02 visual (occipital pole), ICN03 visual (lateral), ICN04 DMN, ICN05 cerebellum, ICN06 sensori-motor, ICN07 auditory, ICN08 executive control, ICN09 fronto-parietal (perception, somesthesis, pain), ICN10 fronto-parietal (cognition-language).*

	Component ICs													*Noise ICs*					
	IC01	IC03	IC05	IC06	IC07	IC08	IC09	IC11	IC14	IC15	IC18	IC19	IC20	IC02	IC04	IC10	IC12	IC13	IC16	IC17
ICN01	**0.33**	**0.18**	0.11	0.16	0.11	0.12		0.15		**0.13**	0.11		0.11	0.16	0.19	0.13		**0.12**	0.13	
ICN02	**0.24**		0.12	0.13	**0.17**	0.12	0.11	0.11		0.11	**0.15**	**0.12**	0.11	**0.19**	0.12	**0.19**	**0.18**	0.11	0.12	**0.11**
ICN03	**0.18**	0.13	0.15	0.16	0.13	0.12	**0.16**	0.13	0.11	0.13	**0.13**		0.11	0.16	0.12	0.16	**0.14**		0.12	
ICN04	0.17	**0.26**	**0.22**	0.14	0.12	0.12	0.15	**0.16**	**0.13**		**0.12**		**0.20**	**0.20**	**0.21**	**0.17**			**0.17**	0.11
ICN05	0.13	0.14		0.12	0.12	**0.25**	0.13		0.13	**0.19**	0.11	**0.15**		**0.18**	0.16	0.12	0.12	**0.26**	0.12	0.11
ICN06	0.11	0.13	0.17	0.14	**0.22**	0.15	0.14	0.16			0.11			0.11	**0.50**	0.12			0.12	
ICN07		0.12	0.19	0.11	0.16	**0.21**	0.14	0.13	**0.15**	0.11		0.11	0.11	0.13	0.11			**0.12**	**0.14**	
ICN08	0.14	**0.17**	**0.21**	**0.17**	0.15	0.16	0.14	**0.16**	**0.13**		0.11	**0.12**	**0.15**	0.18	**0.23**	0.15		0.11	**0.16**	**0.11**
ICN09	0.14	0.17	0.19	**0.16**	**0.17**	**0.17**	**0.19**	**0.17**	0.12	0.11	0.12		0.14	0.15	0.11	0.15	**0.15**	0.11	0.12	
ICN10	0.12	0.17	**0.22**	**0.17**	0.15	0.16	**0.24**	0.13		**0.15**	0.11	0.11	**0.16**	0.15	0.19	**0.16**	0.14	0.11	0.12	**0.11**
Top 3 in % of sum	48%	42%	42%	34%	37%	40%	42%	38%	53%	50%	37%	63%	47%	35%	49%	39%	65%	53%	36%	61%

Supplementary Table 6. Normalised Mean ICN$_i$ Activation (MA$_{N,i}$) values for the NYU-TRT group ICA data atlased using the BRAINMAP20 atlas. *Bold typeface indicates the highest 3 values for a given IC, note that due to data presentation using two significant digits minor differences are not shown in the table, but the markings are based on 8 digits precision data. The last row shows the contribution of the 3 highest values for a given IC in percentage of the sum. BM1: limbic and medial-temporal areas, BM2: subgenual ACC and OFC, BM3: bilateral BG and thalamus, BM4: bilateral anterior insula/frontal opercula and the anterior aspect of the body of the cingulate gyrus, BM5: midbrain, BM6: superior and middle frontal gyri, BM7: middle frontal gyri and superior parietal lobules, BM8: ventral precentral gyri, central sulci, postcentral gyri, superior and inferior cerebellum, BM9: superior parietal lobule, BM10: middle and inferior temporal gyri, BM11: lateral posterior occipital cortex, BM12: medial posterior occipital cortex, BM13: medial prefrontal and posterior cingulate/precuneus areas, DMN, BM14: cerebellum, BM15: right-lateralized fronto-parietal regions, BM16: transverse temporal gyri, BM17: dorsal precentral gyri, central sulci, postcentral gyri, superior and inferior cerebellum, BM18: left-lateralized fronto-parietal regions, BM19-20: artefactual.*

	Component ICs													*Noise ICs*						
	IC01	IC03	IC05	IC06	IC07	IC08	IC09	IC11	IC14	IC15	IC18	IC19	IC20	IC02	IC04	IC10	IC12	IC13	IC16	IC17
BM01	0.12	0.16	**0.21**	0.12	0.12	0.16	0.14	0.11	**0.16**		0.11	**0.15**	**0.15**	0.12	0.17	0.11	0.16	**0.14**	0.13	**0.23**
BM02		0.18	0.18	0.11		0.15	0.14	0.15	0.11		0.12	0.11	**0.22**	0.13	0.17	**0.28**	0.11		0.18	0.12
BM03	0.11	0.12	0.11	0.12	0.12	0.14	0.13	0.13				0.12	0.12	0.16	0.18		0.11	0.12	**0.21**	0.12
BM04			0.21	0.12	0.15	**0.22**	0.14	0.15	**0.15**			0.11	0.15	0.14	0.11	0.11		0.13	0.11	
BM05	0.12	0.18	0.18	0.12	0.14	0.13	0.14	**0.18**	**0.15**	**0.16**		**0.14**	0.12	0.16	**0.26**	**0.26**	0.14	**0.25**	0.13	**0.20**
BM06	0.11	0.11	0.16	0.12	**0.24**	0.15	0.19	0.13					0.12	0.13		0.12	0.14	0.11	0.12	
BM07	0.17	0.17	0.13	**0.20**	0.14	0.11	**0.23**	0.16			0.12			0.16		0.14		0.14	0.12	0.11
BM08	0.13	0.11	0.16	0.13	**0.22**	0.17	0.18	0.13	0.11	**0.20**			0.12		0.17	0.12	0.12	0.14	0.11	
BM09		**0.24**		**0.17**	**0.24**		0.12	0.13					0.12	0.16	0.13	0.11	0.13		0.11	
BM10	0.15	0.15	0.14	0.14	0.12	0.12	0.18	0.11		0.11	**0.13**		0.12	0.13	0.11	0.15	0.14		0.16	
BM11	**0.21**		0.13	0.15	0.11		0.12		0.11	0.12	**0.15**			**0.22**	0.11	0.20	**0.19**	0.11	0.12	
BM12	**0.31**	**0.25**	0.12	0.14	0.11	0.12		0.15		0.15	**0.13**		0.11	**0.21**	**0.24**	0.17	0.11	0.12	**0.19**	
BM13		**0.22**	**0.23**	0.15	0.14	0.13	0.17	0.16	0.12		0.11	0.11	0.12	0.17	0.13	0.17	**0.16**		**0.19**	0.11
BM14	0.16		0.16	0.11	0.12		0.15	0.11		**0.17**	0.12			0.17	0.14	0.12	0.13	0.12	0.11	0.11
BM15	0.11	0.13	0.17	0.13	0.14	0.15	**0.20**	**0.17**	0.12			0.11	0.15	0.12	0.11	**0.23**	0.14	0.11	0.12	
BM16		0.12	0.21	0.11	0.16	**0.18**	0.16	0.12	0.14	0.11			0.12	0.12				0.12	0.18	
BM17	0.17	0.12	0.11	0.11	0.18	**0.18**	0.14			0.15		0.12	0.12	0.12			0.12		0.12	
BM18	0.12	0.11	0.21	0.11	0.12	0.15	**0.26**	0.12					0.13	0.13		0.12	0.14	0.11	0.12	
BM19	0.17	0.14	0.17	0.16	0.16	0.14	0.15	0.15	0.14	0.11	0.12	**0.16**	**0.16**	**0.20**	0.11	0.20	0.14	**0.17**	0.18	**0.27**
BM20	**0.19**	0.16	**0.24**	**0.20**	0.15	0.14	0.20	**0.16**	0.13		0.11	0.11	0.12	0.20	**0.39**	0.17	**0.17**	0.14	0.12	0.11
Top 3 in % of sum	30%	27%	21%	21%	24%	23%	22%	21%	32%	42%	34%	36%	24%	21%	35%	28%	22%	28%	21%	51%

Supplementary Table 7. Normalised Mean ICN$_i$ Activation (MA$_{N,i}$) values for the NYU-TRT group ICA data atlased using the BRAINMAP70 atlas. *Bold typeface indicates the highest 3 values for a given IC, note that due to data presentation using two significant digits minor differences are not shown in the table, but the markings are based on 8 digits precision data. The last row shows the contribution of the 3 highest values for a given IC in percentage of the sum. See Supplementary Table 1 for the description of the atlas ICNs (BM01-BM70).*

	Component ICs													*Noise ICs*						
	IC01	IC03	IC05	IC06	IC07	IC08	IC09	IC11	IC14	IC15	IC18	IC19	IC20	IC02	IC04	IC10	IC12	IC13	IC16	IC17
BM01	**0.33**	0.14	0.11	0.14		0.13		0.14			**0.13**		0.11	0.21		0.21	0.12	0.12	0.11	
BM02	**0.41**	0.11			0.11					0.14	0.11			0.17	0.13	0.14	0.11		0.13	
BM03	0.21		0.12	0.13	0.11		0.11		0.11		**0.15**			**0.23**		0.20	**0.19**	0.11	0.11	
BM04	0.17		0.11	0.13	0.12	0.11	0.18			0.14	0.13			0.16		0.13	0.15		0.12	
BM05	0.25			0.12		0.11					0.12			0.12					0.13	
BM06			0.12	0.15	0.16		**0.25**	0.12			0.12								0.11	
BM07	0.19	0.17	0.14	**0.24**	0.11		0.22	0.17			0.11			0.18		0.14		0.14		0.11
BM08	0.13	0.17	0.17	0.17			0.22	0.16			0.11			0.12		0.11			0.15	
BM09	0.19	0.16	0.13	**0.19**		0.11	0.17	0.13			**0.14**				0.11	0.20	0.11		0.12	
BM10				0.18	0.20	**0.21**	0.16	0.12	**0.23**	**0.18**	0.12		0.12	0.14		0.11	0.14		0.12	
BM11			0.15	0.12	0.11	0.15	0.20	**0.17**					0.11	0.12		0.15	0.15	0.11		
BM12	0.11	0.12	0.13	0.15	0.13		0.25	0.16	0.12		0.11					0.13	0.14		0.11	
BM13	0.11		0.13	0.12	0.15	0.14	0.18	0.15							0.11		0.13	0.11	0.12	
BM14	0.12	0.18	0.13	0.13	0.12	0.14	0.11		0.13	0.12	0.11	0.11	0.15	0.12	0.16		0.16	0.13	0.15	0.12
BM15			0.18	0.12	0.25	0.15	0.14	0.14						0.13		0.12	0.12	0.11	0.12	
BM16	0.11		0.16	0.11	0.22		0.16	0.12					0.11				0.15		0.13	
BM17	0.11		0.13		0.15	0.19	0.21	**0.17**				0.11		0.11					0.11	
BM18		0.14	0.12	0.11	0.11	0.16		0.11	0.15			0.13	0.16	0.11	0.14		0.11	0.13		**0.20**
BM19		0.15	0.20	0.11	0.12	0.17	0.13	0.11	0.14	0.14		**0.15**	0.15	0.12	0.12		0.16	**0.26**	0.13	
BM20		0.17	0.14			0.13	0.13	0.11	0.12		0.11		**0.28**	0.11			0.11		0.12	0.11
BM21		**0.23**	0.20			0.14	0.11	0.15			0.12		**0.21**			**0.27**				
BM22		0.14	0.17			0.17	0.12	0.17					0.13	0.14					0.16	0.11
BM23	0.12	0.17		0.11	0.11	0.11		0.11	0.11			0.11		**0.23**	**0.30**			0.14	**0.25**	
BM24		0.20	0.20	0.12	0.12	0.12	0.15	**0.17**						0.12	0.12			0.13	**0.27**	
BM25			0.19		0.11	**0.20**	0.15	0.13	0.14			0.11	0.17	0.13	0.11			0.12	0.14	
BM26					0.12	0.18	0.14	0.12					0.11	0.13					0.13	
BM27		0.11	0.20		0.14	0.17	0.13	0.13	0.14	0.11			0.17	0.13	0.11	0.11				

Supplementary Table 7 continued

	Component ICs													*Noise ICs*						
	IC01	IC03	IC05	IC06	IC07	IC08	IC09	IC11	IC14	IC15	IC18	IC19	IC20	IC02	IC04	IC10	IC12	IC13	IC16	IC17
BM28	0.11	0.14	**0.28**	0.11	0.12	0.14	0.16	0.16					0.11	0.11	0.11	0.18	0.16	0.14	0.12	
BM29		0.18	**0.25**	0.16		0.11	0.20	0.11						0.12		0.16			0.18	0.11
BM30	0.12	0.14	0.18	0.11	0.13	0.12	0.21	0.13					0.13				**0.18**		0.12	
BM31		0.14	0.12	0.11	0.12	0.15	0.13	0.14				0.12	**0.20**	0.13	0.18	0.11			0.20	0.12
BM32	0.22	0.17		0.12		0.11				0.11	0.12			0.16	0.20		0.11	0.11	0.23	
BM33	0.13	0.12		0.12	**0.26**		0.19		0.13	**0.20**			0.13		0.18	0.11	0.13	0.19		0.12
BM34	0.12	0.16		0.13	**0.26**		0.12		0.11	0.14			0.12		0.15	0.11	0.12		0.11	
BM35	0.15	**0.25**	0.12		**0.26**								0.12	0.14	**0.30**	0.12	0.13		0.12	
BM36		0.13	0.17	0.12	0.19	0.13	0.11	0.16						0.11		0.11			0.13	
BM37			0.12	0.13	0.17	0.16	0.19	0.16	0.11			0.11	0.11				0.11		0.11	
BM38		0.16	0.21	0.13	0.12	0.14	0.18	0.16	0.11			0.11				0.11				
BM39		0.16	0.19		0.11	0.13	0.11		0.15			**0.17**	0.14			0.11	0.14	0.13	0.12	0.18
BM40				0.16	0.23			0.13					0.12	0.17		0.12	0.12		0.11	
BM41		0.13	0.22		0.13	0.18	0.11		**0.18**			0.13	0.17	0.14	0.11	0.12		0.14		
BM42	0.13	0.12	0.15	0.13	0.11	0.12	0.14				0.12					0.14	0.12		0.14	
BM43		0.12	0.18	0.14	0.11	0.12	0.19	0.11		0.11	0.11		0.14	0.15			0.15		0.14	
BM44		0.12	0.19	0.11	0.17	0.17	0.13	0.11	0.13	0.11				0.11				0.12		
BM45		0.12	0.17	0.12	0.14	0.15		0.11						0.11				0.13	**0.24**	
BM46		0.11	0.16	0.12	0.17	0.17	0.12	0.12												
BM47		0.12			0.15	0.18												0.12	0.13	
BM48	0.11		0.15			0.14	0.23	0.12				0.11		0.12				0.11	0.13	
BM49	0.11		**0.24**		0.11	0.15	**0.26**						0.16	0.12		0.12	0.12	0.11		
BM50		0.11	0.23	0.11	0.11	0.11	0.22		0.12	0.11			0.14	0.12			0.14		0.12	
BM51	0.12		0.18		0.14	0.13	**0.26**										0.13	0.11		
BM52		0.11	0.20		0.15	**0.26**	0.16	0.12	0.15			0.11		0.14				0.13		
BM53	0.11	0.11	0.15			0.14	0.20	0.16					0.19	0.12		**0.27**			0.11	
BM54	0.11	0.11			0.12	0.12	0.11	0.13				0.12	0.11	0.17	0.17		0.11		0.21	0.12
BM55		0.11			0.13	0.17	0.11	0.12	0.15			0.12		0.13					0.11	
BM56		0.13		0.11				0.11	0.15	0.13		0.14	0.12	0.12	0.23		0.14	**0.25**		**0.23**
BM57					0.17	0.16	0.12	0.13						0.15					0.14	

Supplementary Table 7 continued

	Component ICs													*Noise ICs*						
	IC01	IC03	IC05	IC06	IC07	IC08	IC09	IC11	IC14	IC15	IC18	IC19	IC20	IC02	IC04	IC10	IC12	IC13	IC16	IC17
BM58			0.11		0.12	0.11	0.13	0.14				0.11		0.14	0.17			0.12	0.20	
BM59			0.17	0.11	0.12	0.13	0.14	0.11		0.15			0.12	0.17			0.13	0.14	0.17	
BM60	0.17	0.14			0.11				0.11	**0.20**	0.11			0.15	0.24	0.12	0.12	0.22	0.13	0.11
BM61	**0.30**	**0.31**	0.21	0.14	0.12	0.12		0.14						**0.21**	0.16	0.12			0.18	
BM62		0.21	0.23	0.18	0.15	0.13	0.13	0.16						0.13						
BM63		0.11	0.11	0.11	0.17	0.15	0.12							0.12					0.13	
BM64		0.14	0.13	0.13	0.13		0.15	0.16						0.14			0.16		0.14	
BM65		0.13	0.13	0.12	0.11		0.14			0.15		0.11	0.15		0.13	0.26	0.13	**0.26**		
BM66	0.18		0.16		0.11		0.15			0.16	0.12			0.20		0.12	0.12		0.11	
BM67	0.23	0.13		0.17	0.14			0.15	0.13	0.14		0.13	0.15	0.18	0.11		0.16	0.18	0.15	
BM68	0.12	0.13	0.17	0.11	0.15	0.12	0.16	0.17					0.12	0.12		**0.34**	0.14		0.16	0.11
BM69	0.20	0.17	0.22	**0.21**	0.16	0.12	0.19	0.17	**0.16**		0.12	0.11	0.12	0.20	**0.44**	0.17	**0.18**	0.14	0.12	0.11
BM70	0.14	0.13	0.16	0.14	0.14	0.11	0.14	0.14	0.14	0.11		**0.16**	0.15	0.21		0.17	0.14	0.17	0.11	**0.25**
Top 3 in % of sum	19%	11%	9%	10%	9%	9%	9%	7%	17%	22%	18%	19%	14%	9%	24%	17%	10%	17%	10%	32%

Supplementary table 8. Normalised Relative ICN$_i$ Activation (RA$_{N,i}$) values for the NYU-TRT group ICA data atlased using the SMITH10 atlas. *Bold typeface indicates the highest 3 values for a given IC, note that due to data presentation using two significant digits minor differences are not shown in the table, but the markings are based on 8 digits precision data. The last row shows the contribution of the 3 highest values for a given IC in percentage of the sum. ICN01 visual (medial), ICN02 visual (occipital pole), ICN03 visual (lateral), ICN04 DMN, ICN05 cerebellum, ICN06 sensori-motor, ICN07 auditory, ICN08 executive control, ICN09 fronto-parietal (perception, somesthesis, pain), ICN10 fronto-parietal (cognition-language).*

	Component ICs													*Noise ICs*						
	IC01	IC03	IC05	IC06	IC07	IC08	IC09	IC11	IC14	IC15	IC18	IC19	IC20	IC02	IC04	IC10	IC12	IC13	IC16	IC17
ICN01	**0.66**	**0.08**	0.00	0.08	0.00	0.01	0.00	0.01	0.00	**0.04**	0.01	0.00	0.00	0.11	**0.16**	0.06	0.00	0.00	**0.14**	0.00
ICN02	**0.17**	0.00	0.00	0.00	0.01	0.00	0.00	0.00	0.00	0.00	**0.55**	0.10	0.00	**0.23**	0.02	**0.42**	**0.75**	0.00	0.01	0.01
ICN03	**0.11**	0.00	0.02	**0.34**	0.00	0.00	0.03	0.00	0.00	**0.01**	**0.40**	0.00	0.00	0.09	0.00	0.09	**0.14**	0.00	0.04	0.00
ICN04	0.03	**0.54**	**0.28**	0.11	0.00	0.00	0.01	**0.16**	**0.10**	0.00	0.01	0.00	**0.17**	0.12	0.07	0.09	0.00	0.00	**0.10**	0.03
ICN05	0.01	0.00	0.00	0.00	0.00	0.00	0.00	0.00	0.05	**0.93**	0.00	**0.56**	0.00	**0.18**	**0.21**	0.02	**0.06**	**0.96**	0.02	**0.29**
ICN06	0.00	0.01	0.01	0.08	**0.58**	0.04	0.02	0.05	0.00	0.00	0.00	0.00	0.00	0.00	0.00	0.04	0.00	0.00	0.07	0.00
ICN07	0.00	0.07	0.11	0.00	**0.21**	**0.44**	0.00	0.00	**0.55**	0.00	0.00	**0.15**	0.00	0.04	0.01	0.00	0.00	**0.02**	0.01	0.00
ICN08	0.00	**0.19**	0.13	0.04	0.02	**0.25**	**0.05**	**0.44**	0.03	0.00	**0.01**	**0.18**	**0.73**	**0.16**	**0.51**	0.01	0.00	0.00	**0.59**	**0.18**
ICN09	0.00	0.05	**0.19**	**0.21**	**0.10**	**0.13**	**0.28**	**0.29**	**0.26**	0.00	0.01	0.00	**0.06**	0.03	0.01	**0.14**	0.02	**0.01**	0.00	0.00
ICN10	0.00	0.06	**0.25**	**0.13**	0.07	0.12	**0.60**	0.04	0.00	0.01	0.00	0.01	0.03	0.03	0.01	**0.12**	0.03	0.00	0.02	**0.50**
Top 3 in % of sum	95%	80%	72%	68%	89%	82%	93%	88%	91%	99%	96%	89%	97%	58%	88%	69%	95%	99%	82%	96%

Supplementary Table 9. Normalised Relative ICN$_i$ Activation (RA$_{N,i}$) values for the NYU-TRT group ICA data atlased using BRAINMAP20. *Bold typeface indicates the highest 3 values for a given IC, note that due to data presentation using two significant digits minor differences are not shown in the table, but the markings are based on 8 digits precision data. The last row shows the contribution of the 3 highest values for a given IC in percentage of the sum. BM1: limbic and medial-temporal areas, BM2: subgenual ACC and OFC, BM3: bilateral BG and thalamus, BM4: bilateral anterior insula/frontal opercula and the anterior aspect of the body of the cingulate gyrus, BM5: midbrain, BM6: superior and middle frontal gyri, BM7: middle frontal gyri and superior parietal lobules, BM8: ventral precentral gyri, central sulci, postcentral gyri, superior and inferior cerebellum, BM9: superior parietal lobule, BM10: middle and inferior temporal gyri, BM11: lateral posterior occipital cortex, BM12: medial posterior occipital cortex, BM13: medial prefrontal and posterior cingulate/precuneus areas, DMN, BM14: cerebellum, BM15: right-lateralized fronto-parietal regions, BM16: transverse temporal gyri, BM17: dorsal precentral gyri, central sulci, postcentral gyri, superior and inferior cerebellum, BM18: left-lateralized fronto-parietal regions*, BM19-20: artefactual.

	Component ICs													*Noise ICs*						
	IC01	IC03	IC05	IC06	IC07	IC08	IC09	IC11	IC14	IC15	IC18	IC19	IC20	IC02	IC04	IC10	IC12	IC13	IC16	IC17
BM01	0.00	0.08	0.12	0.00	0.01	0.08	0.00	0.00	**0.65**	0.00	0.00	**0.40**	**0.10**	0.02	0.09	0.00	0.09	0.04	0.04	**0.34**
BM02	0.00	**0.19**	0.06	0.00	0.00	0.10	0.02	0.10	0.00	0.00	0.03	0.00	**0.67**	0.03	0.04	**0.25**	0.00	0.00	**0.21**	0.04
BM03	0.00	0.00	0.00	0.00	0.02	0.04	0.01	0.08	0.00	0.00	0.00	**0.12**	0.00	0.08	**0.25**	0.00	0.00	0.00	**0.30**	0.01
BM04	0.00	0.00	0.05	0.00	0.03	**0.25**	0.01	0.07	0.07	0.00	0.00	0.00	0.01	0.02	0.00	0.00	0.00	0.00	0.00	0.00
BM05	0.00	0.03	0.00	0.01	0.00	0.00	0.00	0.00	**0.08**	**0.42**	0.00	0.10	0.00	0.00	**0.35**	0.00	0.03	**0.70**	0.00	**0.34**
BM06	0.00	0.00	0.01	0.01	**0.23**	0.02	0.03	0.01	0.00	0.00	0.00	0.00	0.00	0.01	0.00	0.02	0.02	0.00	0.04	0.00
BM07	**0.04**	0.06	0.00	**0.44**	0.03	0.00	0.15	0.06	0.00	0.00	0.06	0.00	0.00	0.02	0.00	0.02	0.00	0.00	0.02	0.01
BM08	0.00	0.00	0.00	0.04	**0.20**	0.03	0.03	0.01	0.00	0.03	0.00	0.00	0.02	0.00	0.01	0.01	0.01	0.00	0.00	0.00
BM09	0.00	0.00	0.00	0.03	**0.16**	0.00	0.00	0.00	0.00	0.00	0.00	0.00	0.00	0.03	0.00	0.02	0.01	0.00	0.00	0.00
BM10	0.03	0.01	0.02	**0.14**	0.00	0.02	0.04	0.00	0.00	0.00	**0.16**	0.00	0.00	0.00	0.00	0.03	0.07	0.00	0.12	0.00
BM11	**0.14**	0.00	0.00	0.02	0.00	0.00	0.00	0.00	0.00	0.01	**0.68**	0.00	0.00	**0.24**	0.00	**0.17**	**0.35**	0.00	0.01	0.00
BM12	**0.72**	**0.20**	0.00	0.06	0.00	0.01	0.00	0.01	0.00	**0.05**	**0.06**	0.00	0.00	0.09	**0.17**	0.05	0.00	0.01	**0.14**	0.00
BM13	0.00	**0.30**	**0.22**	0.07	0.01	0.02	0.04	**0.19**	0.00	0.00	0.00	0.00	0.01	0.02	0.00	0.03	0.01	0.00	0.05	0.00
BM14	0.02	0.00	0.01	0.00	0.00	0.00	0.03	0.00	0.00	**0.49**	0.02	0.00	0.00	**0.15**	0.01	0.00	0.03	0.00	0.00	0.01
BM15	0.00	0.00	0.02	0.03	0.01	0.09	**0.20**	**0.20**	0.02	0.00	0.00	0.00	0.05	0.00	0.00	**0.16**	0.04	0.00	0.00	0.00
BM16	0.00	0.06	**0.12**	0.00	0.09	**0.18**	0.02	0.00	**0.12**	0.00	0.00	0.00	0.00	0.01	0.00	0.00	0.00	0.01	0.02	0.00
BM17	0.00	0.01	0.00	0.00	0.15	**0.10**	0.01	0.00	0.00	0.00	0.00	0.01	0.00	0.00	0.00	0.00	0.00	0.00	0.01	0.00
BM18	0.01	0.00	0.07	0.00	0.00	0.06	**0.21**	0.00	0.00	0.00	0.00	0.00	0.00	0.00	0.00	0.00	0.03	0.00	0.01	0.00
BM19	0.01	0.00	0.09	0.03	0.01	0.00	0.04	0.00	0.04	0.01	0.00	**0.36**	**0.13**	**0.18**	0.00	0.13	**0.19**	**0.16**	0.01	**0.23**
BM20	0.03	0.06	**0.18**	**0.11**	0.04	0.01	**0.16**	**0.24**	0.03	0.00	0.00	0.01	0.00	0.08	0.07	0.10	**0.13**	**0.06**	0.01	0.01
Top 3 in % of sum	90%	69%	52%	69%	59%	53%	57%	63%	84%	95%	89%	88%	89%	57%	77%	58%	67%	92%	65%	91%

Supplementary Table 10. Normalised Relative ICN$_i$ Activation (RA$_{N,i}$) values for the NYU-TRT group ICA data atlased using BRAINMAP70. *Bold typeface indicates the highest 3 values for a given IC, note that due to data presentation using two significant digits minor differences are not shown in the table, but the markings are based on 8 digits precision data. The last row shows the contribution of the 3 highest values for a given IC in percentage of the sum. See Supplementary Table 1 for the description of the atlas ICNs (BM01-BM70).*

	Component ICs													*Noise ICs*						
	IC01	IC03	IC05	IC06	IC07	IC08	IC09	IC11	IC14	IC15	IC18	IC19	IC20	IC02	IC04	IC10	IC12	IC13	IC16	IC17
BM01	**0.27**	0.00	0.00	0.02	0.00	0.00	0.00	0.00	0.00	0.00	0.05	0.00	0.00	0.04	0.00	0.06	0.00	0.01	0.00	0.00
BM02	**0.16**	0.00	0.00	0.00	0.00	0.00	0.00	0.00	0.00	0.01	0.00	0.00	0.00	0.02	0.00	0.01	0.00	0.00	0.00	0.00
BM03	0.08	0.00	0.00	0.00	0.00	0.00	0.00	0.00	0.00	0.00	**0.54**	0.00	0.00	**0.14**	0.00	0.12	**0.30**	0.00	0.00	0.00
BM04	0.02	0.00	0.00	0.03	0.00	0.00	0.01	0.00	0.00	0.01	**0.14**	0.00	0.00	0.03	0.00	0.00	0.06	0.00	0.00	0.00
BM05	0.07	0.00	0.00	0.00	0.00	0.00	0.00	0.00	0.00	0.00	0.04	0.00	0.00	0.00	0.00	0.00	0.00	0.00	0.02	0.00
BM06	0.00	0.00	0.00	0.05	0.02	0.00	0.05	0.00	0.00	0.00	0.01	0.00	0.00	0.00	0.00	0.00	0.00	0.00	0.00	0.00
BM07	0.03	0.03	0.00	**0.19**	0.00	0.00	0.04	0.04	0.00	0.00	0.00	0.00	0.00	0.03	0.00	0.03	0.00	0.01	0.00	0.01
BM08	0.00	0.02	0.00	0.05	0.00	0.00	0.03	0.01	0.00	0.00	0.01	0.00	0.00	0.00	0.00	0.00	0.00	0.00	0.04	0.00
BM09	0.06	0.01	0.01	**0.13**	0.00	0.00	0.01	0.00	0.00	0.00	**0.12**	0.00	0.00	0.00	0.00	0.04	0.00	0.00	0.00	0.00
BM10	0.00	0.00	0.00	0.08	0.07	0.00	0.01	0.00	0.00	0.00	0.01	0.00	0.00	0.00	0.00	0.00	0.01	0.00	0.01	0.00
BM11	0.00	0.00	0.00	0.01	0.00	0.06	0.05	**0.09**	0.00	0.00	0.00	0.00	0.00	0.00	0.00	0.03	0.04	0.00	0.00	0.00
BM12	0.00	0.00	0.00	0.02	0.00	0.00	0.03	0.02	0.02	0.00	0.00	0.00	0.00	0.00	0.00	0.00	0.00	0.00	0.00	0.00
BM13	0.00	0.00	0.00	0.01	0.01	0.01	0.04	0.02	0.00	0.00	0.00	0.00	0.00	0.00	0.00	0.00	0.01	0.00	0.00	0.00
BM14	0.00	0.06	0.00	0.01	0.00	0.00	0.00	0.00	0.02	0.00	0.00	0.01	0.01	0.01	0.02	0.00	0.03	0.00	0.02	0.01
BM15	0.00	0.00	0.01	0.00	0.07	0.01	0.00	0.01	0.00	0.00	0.00	0.00	0.00	0.00	0.00	0.00	0.00	0.00	0.00	0.00
BM16	0.00	0.00	0.00	0.00	0.07	0.00	0.01	0.00	0.00	0.00	0.00	0.00	0.00	0.00	0.00	0.00	0.01	0.00	0.00	0.00
BM17	0.00	0.00	0.00	0.00	0.01	0.03	0.01	0.04	0.00	0.00	0.00	0.00	0.00	0.00	0.00	0.00	0.00	0.00	0.00	0.00
BM18	0.00	0.01	0.00	0.00	0.00	0.04	0.00	0.00	**0.12**	0.00	0.00	0.05	0.02	0.00	0.02	0.00	0.00	0.00	0.00	**0.23**
BM19	0.00	0.02	0.04	0.00	0.00	0.02	0.00	0.00	0.11	0.00	0.00	0.05	0.03	0.00	0.00	0.00	0.03	0.01	0.01	0.00
BM20	0.00	0.02	0.00	0.00	0.00	0.01	0.00	0.00	0.00	0.00	0.00	0.00	**0.25**	0.00	0.00	0.00	0.00	0.00	0.00	0.00
BM21	0.00	**0.11**	0.06	0.00	0.00	0.01	0.00	0.03	0.00	0.00	0.03	0.00	**0.19**	0.00	0.00	**0.13**	0.00	0.00	0.00	0.00
BM22	0.00	0.03	0.01	0.00	0.00	0.04	0.00	0.06	0.00	0.00	0.00	0.00	0.04	0.02	0.00	0.00	0.00	0.00	0.06	0.00
BM23	0.00	0.01	0.00	0.00	0.00	0.00	0.00	0.00	0.00	0.00	0.00	0.00	0.00	0.04	0.11	0.00	0.00	0.01	0.06	0.00
BM24	0.00	0.04	0.01	0.00	0.00	0.00	0.00	0.05	0.00	0.00	0.00	0.00	0.00	0.01	0.00	0.00	0.00	0.01	**0.11**	0.00
BM25	0.00	0.00	0.02	0.00	0.00	**0.06**	0.01	0.01	0.03	0.00	0.00	0.00	0.01	0.00	0.00	0.00	0.00	0.00	0.00	0.00
BM26	0.00	0.00	0.00	0.00	0.00	0.02	0.00	0.00	0.00	0.00	0.00	0.00	0.00	0.00	0.00	0.00	0.00	0.00	0.01	0.00
BM27	0.00	0.00	0.04	0.00	0.00	0.03	0.00	0.01	0.02	0.00	0.00	0.00	0.01	0.00	0.00	0.00	0.00	0.00	0.00	0.00
BM28	0.00	0.02	**0.15**	0.00	0.01	0.01	0.02	**0.10**	0.00	0.00	0.00	0.00	0.00	0.00	0.00	0.02	0.05	0.03	0.00	0.00

Supplementary Table 10 continued

	Component ICs													*Noise ICs*						
	IC01	IC03	IC05	IC06	IC07	IC08	IC09	IC11	IC14	IC15	IC18	IC19	IC20	IC02	IC04	IC10	IC12	IC13	IC16	IC17
BM29	0.00	0.04	0.04	0.01	0.00	0.00	0.02	0.00	0.00	0.00	0.00	0.00	0.00	0.00	0.00	0.03	0.00	0.00	0.00	0.01
BM30	0.00	0.03	0.02	0.00	0.01	0.00	0.07	0.02	0.00	0.00	0.00	0.00	0.00	0.00	0.00	0.00	0.06	0.00	0.01	0.00
BM31	0.00	0.02	0.00	0.00	0.00	0.04	0.01	0.04	0.00	0.00	0.00	0.02	0.11	0.01	0.10	0.00	0.00	0.00	0.06	0.04
BM32	0.08	0.03	0.00	0.01	0.00	0.00	0.00	0.00	0.00	0.00	0.00	0.00	0.00	0.00	0.05	0.00	0.00	0.00	0.07	0.00
BM33	0.00	0.00	0.00	0.00	**0.08**	0.00	0.01	0.00	0.00	0.05	0.00	0.00	0.02	0.00	0.03	0.00	0.01	0.02	0.00	0.00
BM34	0.00	0.00	0.00	0.01	**0.09**	0.00	0.00	0.00	0.00	0.00	0.00	0.00	0.01	0.00	0.00	0.01	0.00	0.00	0.00	0.00
BM35	0.00	0.00	0.00	0.00	0.07	0.00	0.00	0.00	0.00	0.00	0.00	0.00	0.00	0.01	0.00	0.01	0.01	0.00	0.01	0.00
BM36	0.00	0.00	0.01	0.00	0.05	0.01	0.00	0.03	0.00	0.00	0.00	0.00	0.00	0.00	0.00	0.00	0.00	0.00	0.02	0.00
BM37	0.00	0.00	0.00	0.04	0.04	0.03	0.04	0.02	0.00	0.00	0.00	0.00	0.00	0.00	0.00	0.00	0.00	0.00	0.00	0.00
BM38	0.00	0.01	0.05	0.01	0.00	0.01	0.01	0.02	0.00	0.00	0.00	0.00	0.00	0.00	0.00	0.00	0.00	0.00	0.00	0.00
BM39	0.00	0.01	0.02	0.00	0.00	0.00	0.00	0.00	**0.16**	0.00	0.00	**0.33**	0.03	0.00	0.00	0.00	0.03	0.01	0.00	0.01
BM40	0.00	0.00	0.00	0.03	**0.11**	0.00	0.00	0.00	0.00	0.00	0.00	0.00	0.00	0.04	0.00	0.01	0.00	0.00	0.00	0.00
BM41	0.00	0.01	**0.08**	0.00	0.00	0.03	0.00	0.00	**0.25**	0.00	0.00	0.01	0.01	0.01	0.00	0.00	0.00	0.03	0.00	0.00
BM42	0.01	0.00	0.01	0.04	0.00	0.01	0.00	0.00	0.00	0.00	0.02	0.00	0.00	0.00	0.00	0.02	0.01	0.00	0.01	0.00
BM43	0.00	0.00	0.01	0.02	0.00	0.00	0.02	0.00	0.00	0.00	0.00	0.00	0.01	0.00	0.00	0.00	0.03	0.00	0.01	0.00
BM44	0.00	0.03	0.04	0.00	0.04	**0.07**	0.00	0.00	0.05	0.00	0.00	0.00	0.00	0.00	0.00	0.00	0.00	0.01	0.00	0.00
BM45	0.00	0.01	0.01	0.00	0.02	0.03	0.00	0.00	0.00	0.00	0.00	0.00	0.00	0.01	0.00	0.00	0.00	0.01	**0.12**	0.00
BM46	0.00	0.00	0.02	0.01	0.02	0.05	0.00	0.00	0.00	0.00	0.00	0.00	0.00	0.00	0.00	0.00	0.00	0.00	0.00	0.00
BM47	0.00	0.02	0.00	0.00	0.02	0.03	0.00	0.00	0.00	0.00	0.00	0.00	0.00	0.00	0.00	0.00	0.00	0.00	0.00	0.00
BM48	0.00	0.00	0.01	0.00	0.00	0.01	0.04	0.00	0.00	0.00	0.00	0.00	0.00	0.00	0.00	0.00	0.00	0.00	0.01	0.00
BM49	0.00	0.00	0.04	0.00	0.00	0.04	0.07	0.00	0.00	0.00	0.00	0.00	0.00	0.00	0.00	0.00	0.00	0.00	0.00	0.00
BM50	0.00	0.00	0.06	0.00	0.00	0.00	**0.08**	0.00	0.01	0.00	0.00	0.00	0.02	0.00	0.00	0.00	0.04	0.00	0.00	0.00
BM51	0.00	0.00	0.01	0.00	0.01	0.01	0.04	0.00	0.00	0.00	0.00	0.00	0.00	0.00	0.00	0.00	0.00	0.00	0.00	0.00
BM52	0.00	0.00	0.01	0.00	0.02	**0.13**	0.01	0.00	0.04	0.00	0.00	0.00	0.00	0.01	0.00	0.00	0.00	0.00	0.00	0.00
BM53	0.00	0.00	0.00	0.00	0.00	0.03	**0.09**	0.03	0.00	0.00	0.00	0.00	**0.13**	0.00	0.00	**0.22**	0.00	0.00	0.00	0.00
BM54	0.00	0.00	0.00	0.00	0.01	0.01	0.00	0.03	0.00	0.00	0.00	0.06	0.00	0.03	**0.13**	0.00	0.00	0.00	0.03	0.01
BM55	0.00	0.00	0.00	0.00	0.02	0.05	0.00	0.00	0.01	0.00	0.00	0.05	0.00	0.00	0.00	0.00	0.00	0.00	0.00	0.00
BM56	0.00	0.00	0.00	0.00	0.00	0.00	0.00	0.00	0.09	0.05	0.00	**0.13**	0.00	0.00	**0.25**	0.00	0.00	**0.32**	0.00	**0.51**
BM57	0.00	0.00	0.00	0.00	0.01	0.01	0.00	0.00	0.00	0.00	0.00	0.00	0.00	0.00	0.00	0.00	0.00	0.00	0.01	0.00

Supplementary Table 10 continued

	Component ICs													*Noise ICs*						
	IC01	IC03	IC05	IC06	IC07	IC08	IC09	IC11	IC14	IC15	IC18	IC19	IC20	IC02	IC04	IC10	IC12	IC13	IC16	IC17
BM58	0.00	0.00	0.00	0.00	0.00	0.00	0.00	0.02	0.00	0.00	0.00	0.00	0.00	0.04	0.04	0.00	0.00	0.00	**0.14**	0.00
BM59	0.00	0.00	0.00	0.00	0.00	0.00	0.01	0.00	0.00	0.11	0.00	0.00	0.00	0.06	0.00	0.00	0.01	0.00	0.00	0.00
BM60	0.02	0.00	0.00	0.00	0.00	0.00	0.00	0.00	0.00	**0.30**	0.00	0.00	0.00	0.01	**0.13**	0.00	0.01	0.06	0.00	0.01
BM61	**0.14**	**0.26**	0.01	0.02	0.00	0.01	0.00	0.02	0.00	0.00	0.00	0.00	0.00	0.02	0.02	0.00	0.00	0.00	0.02	0.00
BM62	0.00	**0.06**	0.02	0.06	0.01	0.00	0.00	0.05	0.00	0.00	0.00	0.00	0.00	0.00	0.00	0.00	0.00	0.00	0.00	0.00
BM63	0.00	0.00	0.00	0.00	0.06	0.02	0.00	0.00	0.00	0.00	0.00	0.00	0.00	0.00	0.00	0.00	0.00	0.00	0.00	0.00
BM64	0.00	0.01	0.00	0.01	0.00	0.00	0.01	0.05	0.00	0.00	0.00	0.00	0.00	0.00	0.00	0.00	0.00	0.00	0.04	0.00
BM65	0.00	0.00	0.00	0.00	0.00	0.00	0.00	0.00	0.00	**0.16**	0.00	0.01	0.00	0.00	0.00	0.00	0.01	**0.27**	0.00	0.00
BM66	0.02	0.00	0.02	0.00	0.00	0.00	0.03	0.00	0.00	**0.28**	0.02	0.00	0.00	**0.19**	0.00	0.01	0.01	0.00	0.00	0.00
BM67	0.00	0.00	0.00	0.01	0.00	0.00	0.00	0.00	0.00	0.02	0.00	0.01	0.01	0.01	0.00	0.00	0.01	0.08	0.00	0.00
BM68	0.00	0.01	0.02	0.00	0.00	0.01	0.01	0.04	0.00	0.00	0.00	0.00	0.02	0.01	0.00	**0.12**	0.01	0.00	0.06	0.00
BM69	0.02	0.05	**0.07**	**0.09**	0.03	0.00	**0.08**	**0.11**	0.04	0.00	0.00	0.01	0.00	0.06	0.09	0.08	**0.09**	0.03	0.00	0.01
BM70	0.00	0.00	0.05	0.00	0.00	0.00	0.01	0.00	0.02	0.00	0.00	**0.26**	0.07	**0.09**	0.00	0.04	**0.12**	**0.09**	0.00	**0.16**
Top 3 in % of sum	57%	43%	31%	41%	27%	27%	25%	30%	53%	74%	81%	72%	57%	42%	51%	47%	50%	68%	36%	89%

Supplementary Table 11. Within session ICC values for ICN$_i$ Spatial Involvement (I_i) values for the NYU-TRT group ICA data atlased using the SMITH10 atlas. *ICN01 visual (medial), ICN02 visual (occipital pole), ICN03 visual (lateral), ICN04 DMN, ICN05 cerebellum, ICN06 sensori-motor, ICN07 auditory, ICN08 executive control, ICN09 fronto-parietal (perception, somesthesis, pain), ICN10 fronto-parietal (cognition-language). For visual representation see Figure 7 panel A.*

	Component ICs													*Noise ICs*						
	IC01	IC03	IC05	IC06	IC07	IC08	IC09	IC11	IC14	IC15	IC18	IC19	IC20	IC02	IC04	IC10	IC12	IC13	IC16	IC17
ICN01	0.55	0.61	0.39	0.67	0.39	0.82	0.08	0.47	0.34	0.00	0.48	0.30	0.32	0.34	0.75	0.37	0.48	0.27	0.17	-0.13
ICN02	0.38	0.06	0.50	0.49	0.31	0.52	-0.15	0.46	0.43	0.35	0.42	0.32	0.33	0.65	0.00	0.02	0.54	-0.08	-0.07	-0.12
ICN03	0.76	0.77	0.21	0.57	0.33	0.57	0.26	0.79	0.46	0.23	0.60	0.19	0.34	0.25	0.31	0.20	0.37	0.58	0.21	-0.11
ICN04	0.41	0.74	0.56	0.68	0.22	0.77	0.47	0.40	0.15	0.46	0.62	0.43	0.44	0.29	0.70	0.51	0.26	0.04	0.37	0.03
ICN05	0.01	0.30	0.40	-0.10	0.36	0.42	0.13	0.15	0.48	0.41	0.83	0.22	0.11	0.35	0.53	0.23	0.47	0.05	0.36	0.42
ICN06	0.05	0.56	0.62	0.61	0.59	0.65	0.41	0.17	0.34	0.07	0.21	0.42	-0.11	0.60	0.36	0.17	0.48	0.07	0.33	0.03
ICN07	0.10	0.83	0.63	0.55	0.60	0.87	0.03	0.62	0.39	0.42	0.67	0.44	0.23	0.30	0.34	0.18	0.70	0.44	0.40	0.08
ICN08	0.08	0.73	0.46	0.48	0.34	0.91	0.24	0.50	0.33	0.09	0.65	0.48	0.29	0.33	0.67	0.19	0.35	0.30	0.26	-0.12
ICN09	0.26	0.58	0.63	0.58	0.50	0.91	0.42	0.47	0.16	0.55	0.79	0.14	0.17	0.60	0.41	0.30	0.41	0.02	0.31	-0.22
ICN10	0.31	0.71	0.54	0.52	0.35	0.89	0.63	0.30	0.36	0.12	0.76	0.52	-0.06	0.43	0.31	0.18	0.38	-0.20	0.16	-0.05

Supplementary Table 12. Between-session ICC values for ICN$_i$ Spatial Involvement (I_i) values for the NYU-TRT group ICA data atlased using the SMITH10 atlas. *ICN01 visual (medial), ICN02 visual (occipital pole), ICN03 visual (lateral), ICN04 DMN, ICN05 cerebellum, ICN06 sensori-motor, ICN07 auditory, ICN08 executive control, ICN09 fronto-parietal (perception, somesthesis, pain), ICN10 fronto-parietal (cognition-language). For visual representation see Figure 7 panel E.*

	Component ICs													*Noise ICs*					
	IC01	IC03	IC05	IC06	IC07	IC08	IC09	IC11	IC14	IC15	IC18	IC19	IC20	IC02	IC04	IC10	IC12	IC13	IC16	IC17
ICN01	0.73	0.61	0.00	0.25	0.15	0.38	0.12	0.36	-0.07	0.57	0.31	0.26	0.08	0.37	0.69	0.18	0.46	0.51	0.27	0.22
ICN02	0.54	0.04	-0.08	0.17	-0.05	0.29	-0.01	0.06	0.03	0.25	0.32	0.36	0.33	0.61	0.32	0.12	0.53	-0.04	-0.03	0.01
ICN03	0.51	0.60	0.04	0.34	0.54	0.39	0.44	0.08	0.04	0.15	0.51	0.48	0.15	0.56	0.56	0.44	0.41	0.34	0.07	-0.15
ICN04	0.24	0.57	0.24	0.52	0.27	0.50	-0.08	0.27	0.18	0.20	0.32	0.25	0.06	0.48	0.47	0.22	0.35	0.19	0.14	0.32
ICN05	0.39	0.33	-0.08	0.06	-0.04	-0.07	-0.13	0.21	0.24	0.44	0.31	0.29	-0.21	0.40	0.45	0.24	0.27	0.60	-0.03	0.17
ICN06	0.49	0.30	0.09	0.52	0.35	0.18	0.23	0.20	0.04	0.50	0.38	0.34	-0.01	0.48	0.55	0.08	0.24	0.18	0.04	0.04
ICN07	0.35	0.63	0.44	0.47	0.45	0.59	0.16	0.44	0.27	0.31	0.56	0.44	0.22	0.54	0.50	-0.05	0.63	0.50	0.10	0.10
ICN08	0.35	0.57	0.28	0.14	0.52	0.54	-0.18	0.47	0.19	0.05	0.15	0.39	0.23	0.35	0.61	0.20	0.37	0.19	0.10	-0.22
ICN09	0.42	0.36	0.50	0.20	0.28	0.44	0.12	0.53	-0.03	0.28	0.68	0.03	0.40	0.41	0.54	0.10	0.50	0.50	-0.01	-0.02
ICN10	0.56	0.51	0.23	0.39	0.39	0.32	0.20	0.43	0.07	0.28	0.27	0.21	-0.05	0.35	0.38	-0.03	0.22	0.33	-0.03	0.08

Supplementary Table 13. Within-session ICC values for ICN_i Spatial Involvement (I_i) values for the NYU-TRT group ICA data atlased using the BRAINMAP20 atlas. *BM1: limbic and medial-temporal areas, BM2: subgenual ACC and OFC, BM3: bilateral BG and thalamus, BM4: bilateral anterior insula/frontal opercula and the anterior aspect of the body of the cingulate gyrus, BM5: midbrain, BM6: superior and middle frontal gyri, BM7: middle frontal gyri and superior parietal lobules, BM8: ventral precentral gyri, central sulci, postcentral gyri, superior and inferior cerebellum, BM9: superior parietal lobule, BM10: middle and inferior temporal gyri, BM11: lateral posterior occipital cortex, BM12: medial posterior occipital cortex, BM13: medial prefrontal and posterior cingulate/precuneus areas, DMN, BM14: cerebellum, BM15: right-lateralized fronto-parietal regions, BM16: transverse temporal gyri, BM17: dorsal precentral gyri, central sulci, postcentral gyri, superior and inferior cerebellum, BM18: left-lateralized fronto-parietal regions, BM19-20: artefactual. For visual representation see Figure 7 panel I.*

	Component ICs													*Noise ICs*					
	IC01	IC03	IC05	IC06	IC07	IC08	IC09	IC11	IC14	IC15	IC18	IC19	IC20	IC02	IC04	IC10	IC12	IC13	IC16	IC17
BM01	-0.07	0.58	0.65	0.42	0.45	0.69	0.14	0.36	0.43	0.08	0.50	0.54	0.40	0.33	0.28	-0.02	0.60	0.61	0.36	-0.10
BM02	0.09	0.67	0.24	0.43	0.02	0.76	0.11	0.50	0.26	0.05	0.48	0.31	0.48	0.37	0.27	0.18	0.47	-0.11	0.29	-0.13
BM03	0.21	0.62	0.54	0.17	0.65	0.61	0.34	0.27	0.55	0.33	0.50	0.46	-0.04	-0.18	0.62	-0.02	0.42	0.08	0.81	0.32
BM04	0.17	0.73	0.65	0.63	0.39	0.86	0.11	0.62	0.47	-0.06	0.67	0.39	0.25	0.22	0.43	-0.05	0.15	0.15	0.31	-0.06
BM05	0.30	0.60	0.28	-0.06	0.42	0.08	0.14	0.20	0.59	0.48	0.31	0.43	-0.07	0.30	0.49	0.13	0.71	0.02	0.05	0.19
BM06	0.46	0.71	0.19	0.37	0.68	0.62	0.72	0.65	0.63	0.12	0.29	0.46	0.18	0.29	0.42	0.00	0.53	0.31	0.51	0.31
BM07	0.60	0.69	0.34	0.60	0.40	0.50	0.45	0.47	0.38	0.31	0.59	0.36	0.16	0.39	0.13	0.03	0.39	0.25	0.21	-0.09
BM08	0.20	0.50	0.63	0.53	0.53	0.66	0.47	0.25	0.58	0.18	0.28	0.29	0.35	0.83	0.45	0.28	0.62	0.09	0.18	0.33
BM09	0.22	0.03	0.23	0.52	0.59	0.59	0.16	-0.08	0.45	0.08	0.39	0.19	0.26	0.69	0.15	0.16	0.63	0.32	0.58	0.19
BM10	0.53	0.68	0.42	0.53	0.21	0.73	0.43	0.71	0.63	0.30	0.65	0.00	0.17	0.30	0.35	0.31	0.50	0.74	0.43	-0.10
BM11	0.40	0.15	0.36	0.60	0.48	0.29	-0.11	0.57	0.15	0.39	0.47	0.16	0.06	0.73	-0.10	0.07	0.54	0.00	0.03	-0.04
BM12	0.58	0.65	0.46	0.61	0.36	0.80	0.10	0.28	0.34	-0.01	0.55	0.33	0.21	0.61	0.74	0.29	0.45	0.09	0.21	0.03
BM13	0.03	0.77	0.47	0.64	0.28	0.72	0.28	0.18	0.18	0.40	0.48	0.52	0.21	0.49	0.16	0.42	0.32	0.12	0.23	-0.06
BM14	0.50	0.49	0.36	0.26	0.49	0.51	0.29	0.19	0.11	0.52	0.81	0.03	0.27	0.46	0.42	0.35	0.50	0.48	0.28	0.27
BM15	0.24	0.33	0.58	0.47	0.51	0.87	0.38	0.48	0.14	0.25	0.74	0.18	0.12	0.54	0.50	0.32	0.26	0.14	0.33	0.05
BM16	-0.01	0.80	0.66	0.21	0.61	0.84	0.39	0.05	0.31	0.38	0.51	0.28	0.09	0.56	-0.09	0.11	0.68	0.51	0.39	-0.04
BM17	-0.18	0.68	0.47	0.01	0.58	0.77	0.00	0.17	0.19	0.52	0.54	0.35	0.05	0.52	0.47	-0.09	0.56	-0.06	0.38	0.09
BM18	0.51	0.71	0.57	0.57	0.30	0.85	0.61	0.19	0.14	-0.03	0.59	0.48	-0.02	0.42	0.35	-0.04	0.51	-0.03	0.57	-0.03
BM19	-0.04	0.42	0.64	0.42	0.01	0.47	0.17	0.59	0.33	0.19	0.68	0.42	0.34	0.71	0.36	0.15	0.61	0.23	0.10	-0.22
BM20	0.21	0.73	0.53	0.70	0.53	0.70	0.44	0.49	0.20	0.16	0.60	0.34	0.28	0.44	0.04	0.27	0.59	0.17	0.23	0.17

Supplementary Table 14. Between-session ICC values for ICN$_i$ Spatial Involvement (I_i) values for the NYU-TRT group ICA data atlased using the BRAINMAP20 atlas. *BM1: limbic and medial-temporal areas, BM2: subgenual ACC and OFC, BM3: bilateral BG and thalamus, BM4: bilateral anterior insula/frontal opercula and the anterior aspect of the body of the cingulate gyrus, BM5: midbrain, BM6: superior and middle frontal gyri, BM7: middle frontal gyri and superior parietal lobules, BM8: ventral precentral gyri, central sulci, postcentral gyri, superior and inferior cerebellum, BM9: superior parietal lobule, BM10: middle and inferior temporal gyri, BM11: lateral posterior occipital cortex, BM12: medial posterior occipital cortex, BM13: medial prefrontal and posterior cingulate/precuneus areas, DMN, BM14: cerebellum, BM15: right-lateralized fronto-parietal regions, BM16: transverse temporal gyri, BM17: dorsal precentral gyri, central sulci, postcentral gyri, superior and inferior cerebellum, BM18: left-lateralized fronto-parietal regions, BM19-20: artefactual. For visual representation see Figure 7 panel M.*

	Component ICs													*Noise ICs*					
	IC01	IC03	IC05	IC06	IC07	IC08	IC09	IC11	IC14	IC15	IC18	IC19	IC20	IC02	IC04	IC10	IC12	IC13	IC16	IC17
BM01	0.56	0.66	0.48	-0.25	0.20	0.41	0.16	0.29	0.28	0.20	0.61	0.48	0.22	0.35	0.41	0.08	0.21	0.64	0.04	0.38
BM02	0.71	0.66	0.17	0.20	0.06	0.41	-0.14	0.26	0.41	-0.11	0.04	0.36	0.18	0.22	0.39	0.30	0.18	0.16	0.17	-0.03
BM03	0.34	0.59	0.02	0.49	0.50	0.73	0.39	0.41	0.41	0.33	0.60	0.58	0.07	-0.11	0.63	0.21	0.62	0.39	0.54	0.49
BM04	0.39	0.21	0.73	0.36	0.39	0.49	-0.07	0.32	0.21	0.01	0.25	0.27	0.33	0.40	0.34	0.28	0.40	0.25	-0.03	0.14
BM05	0.47	0.44	-0.15	0.19	0.23	0.13	-0.20	0.03	0.29	0.37	-0.06	0.39	0.16	0.36	0.59	0.17	0.33	0.63	-0.14	0.01
BM06	0.30	0.46	0.22	0.38	0.31	0.23	0.67	0.14	0.19	-0.07	0.56	0.54	0.08	0.28	-0.03	0.03	0.24	0.39	0.38	0.13
BM07	0.57	0.58	0.13	0.40	0.44	0.23	0.19	0.63	0.19	0.14	0.42	0.38	0.15	0.59	0.28	-0.06	0.74	0.20	0.04	0.23
BM08	0.14	0.56	0.40	0.52	0.23	0.36	0.24	0.39	0.20	0.39	0.44	0.14	0.23	0.48	0.69	0.28	0.35	0.00	0.11	0.06
BM09	0.38	0.33	-0.15	0.70	0.43	-0.21	-0.10	0.03	0.34	0.12	-0.17	0.07	0.36	0.55	0.00	-0.04	0.23	0.12	0.13	0.18
BM10	0.58	0.79	0.34	0.39	0.60	0.43	0.67	0.00	0.02	0.27	0.44	0.36	0.16	0.37	0.41	0.46	0.38	0.07	0.10	-0.07
BM11	0.49	0.08	0.08	0.29	0.20	0.36	-0.03	-0.07	0.08	0.35	0.37	0.40	0.20	0.73	0.36	0.13	0.47	-0.01	0.00	0.03
BM12	0.72	0.61	-0.10	0.33	0.21	0.35	0.13	0.07	-0.10	0.51	0.28	0.42	0.07	0.67	0.70	0.14	0.46	0.43	0.38	0.19
BM13	0.24	0.62	0.28	0.52	0.28	0.40	-0.04	0.34	0.23	0.24	0.36	0.19	0.55	0.51	0.55	0.09	0.43	0.20	0.18	0.15
BM14	0.53	0.04	0.14	0.46	0.03	0.22	0.43	-0.08	0.28	0.46	0.40	0.22	-0.16	0.64	0.41	0.15	0.52	0.41	0.07	0.19
BM15	0.50	0.44	0.45	0.13	0.26	0.34	0.09	0.46	-0.02	0.11	0.56	-0.21	0.16	0.51	0.55	-0.01	0.06	0.55	-0.18	0.02
BM16	0.12	0.64	0.31	0.32	0.45	0.65	0.45	0.05	0.24	0.27	0.71	0.59	0.20	0.61	0.45	-0.13	0.64	0.61	0.23	0.07
BM17	0.01	0.19	0.26	-0.07	0.32	0.42	0.01	0.41	0.25	0.75	0.59	0.29	-0.02	0.36	0.41	0.29	0.58	0.24	0.03	-0.08
BM18	0.63	0.16	0.15	0.40	0.10	0.24	0.37	-0.05	0.04	0.13	0.14	0.05	0.08	0.06	0.08	-0.07	0.23	0.37	-0.12	-0.08
BM19	0.53	0.73	0.23	0.18	0.29	0.22	0.30	-0.05	0.38	0.25	0.28	0.48	0.20	0.57	0.42	0.17	0.25	0.39	0.03	-0.02
BM20	0.55	0.52	0.19	0.40	0.30	0.45	0.20	0.50	0.17	0.17	0.28	0.11	0.16	0.27	0.20	-0.18	0.35	0.39	0.04	0.05

Supplementary Table 15. Within-session ICC values for ICN$_i$ Spatial Involvement (I_i) values for the NYU-TRT group ICA data atlased using the BRAINMAP70 atlas. *See Supplementary Table 1 for the description of the atlas ICNs (BM01-BM70). For visual representation see Supplementary Figure 7 panel A.*

	Component ICs													*Noise ICs*					
	IC01	IC03	IC05	IC06	IC07	IC08	IC09	IC11	IC14	IC15	IC18	IC19	IC20	IC02	IC04	IC10	IC12	IC13	IC16	IC17
BM01	0.42	0.74	0.28	0.43	0.18	0.37	0.23	0.36	0.61	0.00	0.49	0.08	0.34	0.66	0.00	-0.09	0.43	-0.07	-0.16	0.06
BM02	0.28	0.62	0.48	0.20	0.66	0.31	0.05	0.42	0.46	0.22	0.30	0.37	0.55	0.73	0.48	0.15	0.36	-0.08	0.22	0.43
BM03	0.40	0.11	0.30	0.42	0.41	0.27	0.03	0.56	0.31	0.32	0.41	0.07	0.18	0.78	-0.13	0.14	0.56	-0.04	-0.05	-0.07
BM04	0.53	0.26	0.11	0.60	0.44	0.55	0.26	0.79	0.09	0.26	0.32	0.22	0.01	0.66	0.05	0.21	0.57	0.44	-0.13	-0.05
BM05	0.72	0.46	0.46	0.59	0.26	0.33	0.12	0.16	0.16	0.33	0.35	-0.01	0.05	0.45	0.21	0.06	0.28	0.89	0.31	-0.05
BM06	0.29	0.10	0.30	0.43	0.51	0.42	0.66	0.59	0.11	0.05	0.35	0.20	-0.01	0.33	0.33	0.58	0.39	0.32	0.32	0.12
BM07	0.65	0.57	0.24	0.51	0.04	0.18	0.27	0.58	0.41	0.30	0.52	0.38	0.01	0.51	0.09	0.14	0.21	0.17	0.19	0.22
BM08	0.19	0.70	0.65	0.48	0.04	0.58	0.57	0.14	0.30	0.27	0.70	0.16	0.44	0.04	-0.03	0.34	0.54	-0.13	0.30	-0.26
BM09	0.74	0.67	0.30	0.65	0.00	0.48	0.51	-0.02	-0.13	0.17	0.58	0.11	0.55	-0.14	0.40	-0.02	0.34	0.23	0.02	-0.19
BM10	0.35	0.26	0.70	0.47	0.46	0.54	0.66	0.45	0.19	0.22	0.56	0.64	0.16	0.49	0.48	0.13	0.31	0.30	0.37	0.05
BM11	0.09	0.64	0.51	0.32	0.26	0.78	0.54	0.35	0.00	0.02	0.64	0.10	-0.01	0.24	0.26	0.20	0.12	0.25	0.37	0.08
BM12	0.20	0.32	0.25	0.53	0.32	0.42	0.50	0.51	0.28	0.34	0.66	0.13	0.52	0.74	0.24	0.25	0.50	0.25	0.47	-0.04
BM13	0.12	0.05	0.34	0.38	0.63	0.52	0.47	0.42	0.23	0.18	0.50	0.25	0.10	0.20	0.53	0.01	0.19	0.32	0.49	-0.14
BM14	0.19	0.66	0.01	0.19	0.44	0.25	0.29	0.09	0.46	0.14	0.34	0.26	0.02	0.16	0.24	0.09	0.74	0.06	0.36	0.17
BM15	0.29	0.18	0.38	0.34	0.69	0.62	0.14	0.50	0.68	0.12	0.33	0.42	0.16	0.29	0.14	-0.11	0.29	0.31	0.48	-0.06
BM16	0.58	0.75	0.52	0.44	0.72	0.28	0.34	0.30	0.09	0.52	0.55	0.49	0.37	0.25	0.06	0.52	0.60	0.08	0.19	-0.12
BM17	0.27	0.41	0.46	0.33	0.51	0.75	0.13	0.47	0.17	-0.07	0.26	0.26	-0.13	0.77	0.09	0.07	0.04	-0.10	-0.02	0.31
BM18	-0.10	0.31	0.27	0.14	0.06	0.67	-0.08	0.18	0.58	0.16	0.52	0.46	0.27	0.49	0.31	0.10	0.08	0.27	0.33	0.11
BM19	0.13	0.60	0.55	0.29	0.28	0.57	0.40	0.29	0.44	0.22	0.11	0.49	0.17	0.03	0.34	-0.05	0.72	0.54	0.47	-0.30
BM20	0.26	0.47	0.37	-0.08	0.14	0.42	-0.11	-0.02	0.08	0.09	0.31	0.06	0.24	0.53	0.07	0.48	0.37	-0.05	0.34	-0.16
BM21	0.09	0.40	0.32	0.55	-0.18	0.63	0.19	0.51	0.26	0.33	0.41	0.35	0.38	0.33	0.14	0.11	0.40	-0.09	0.38	0.35
BM22	-0.07	0.53	0.50	0.43	-0.04	0.72	0.24	0.43	0.33	0.05	0.43	0.15	0.47	0.39	0.36	0.27	0.22	0.58	0.03	-0.19
BM23	0.22	0.80	0.42	0.18	0.60	0.47	0.49	0.16	0.37	0.16	0.37	0.63	0.02	0.21	0.59	0.28	0.52	0.42	0.56	0.35
BM24	-0.08	0.65	0.52	0.39	0.36	0.31	0.10	0.08	0.50	0.10	0.17	0.27	0.04	0.30	0.60	0.23	0.32	0.59	0.69	-0.13
BM25	0.37	0.59	0.39	-0.10	0.18	0.72	0.29	0.28	0.48	-0.14	0.35	0.33	0.28	0.00	0.65	0.14	0.07	0.24	0.27	-0.11
BM26	0.03	0.42	0.05	0.14	0.02	0.58	0.55	0.49	0.15	0.36	0.54	0.21	0.02	0.21	0.13	0.23	0.02	0.05	0.58	-0.17
BM27	-0.10	0.37	0.45	0.14	-0.08	0.69	0.37	0.24	0.31	0.16	0.47	0.28	0.45	0.43	0.54	-0.02	0.04	0.36	0.20	-0.08
BM28	-0.08	0.74	0.47	0.14	0.33	0.76	0.11	0.40	0.20	0.54	0.30	-0.17	-0.09	0.41	-0.07	0.20	0.45	0.02	0.14	0.17
BM29	0.05	0.71	0.61	0.70	0.08	0.64	0.68	0.67	0.06	-0.05	0.44	0.75	0.62	-0.06	0.15	0.48	-0.08	-0.27	0.23	-0.16

Supplementary Table 15 continued

	Component ICs													*Noise ICs*					
	IC01	IC03	IC05	IC06	IC07	IC08	IC09	IC11	IC14	IC15	IC18	IC19	IC20	IC02	IC04	IC10	IC12	IC13	IC16	IC17
BM30	0.23	0.55	0.35	0.56	0.68	0.39	0.55	0.40	0.45	-0.10	0.12	0.28	0.07	0.23	0.06	-0.19	0.44	0.04	0.04	0.32
BM31	0.48	0.81	0.54	0.10	0.39	0.63	0.17	0.56	0.20	0.00	0.49	0.41	0.37	0.48	0.27	0.44	0.37	-0.06	0.57	0.00
BM32	0.40	0.61	0.21	0.69	0.43	0.57	0.04	0.35	0.40	-0.06	0.44	0.13	0.07	0.23	0.51	0.17	0.38	-0.01	0.49	0.02
BM33	0.03	0.56	0.36	0.68	0.48	0.66	0.37	0.12	0.41	-0.04	0.41	0.47	0.46	0.55	0.35	0.42	0.75	0.25	0.14	0.40
BM34	0.15	0.31	0.51	0.50	0.55	0.28	0.50	0.09	0.53	0.55	0.11	0.31	0.22	0.29	-0.05	-0.05	0.31	0.40	-0.01	0.16
BM35	0.40	0.43	-0.01	-0.06	0.62	0.06	-0.10	0.23	0.42	-0.01	0.08	-0.05	0.19	0.51	0.38	0.01	0.48	0.22	0.74	0.16
BM36	0.37	0.31	0.64	0.44	0.50	0.53	0.24	0.27	0.49	0.05	-0.01	0.31	-0.05	0.61	0.20	0.01	0.54	-0.05	0.47	-0.06
BM37	0.38	0.20	0.82	0.25	0.52	0.72	0.66	0.42	0.37	0.27	0.38	0.01	0.16	0.34	0.10	0.01	0.37	-0.24	0.23	0.13
BM38	0.30	0.74	0.57	0.68	0.27	0.83	0.84	0.28	0.12	0.56	0.75	0.21	-0.26	0.58	-0.18	-0.08	0.05	-0.16	0.24	-0.04
BM39	0.00	0.45	0.42	0.27	0.34	0.58	0.01	0.50	0.38	0.05	0.48	0.41	0.50	0.74	0.00	0.11	0.58	0.53	0.09	0.11
BM40	0.07	-0.16	0.23	0.54	0.51	0.64	0.33	-0.03	0.49	0.04	0.26	0.24	0.41	0.70	0.15	0.12	0.60	0.34	0.59	0.20
BM41	-0.06	0.65	0.70	0.26	0.60	0.53	0.31	0.42	0.40	0.13	0.34	0.29	0.24	0.67	0.07	0.11	0.56	0.41	-0.07	0.32
BM42	0.55	0.50	0.49	0.65	0.20	0.66	0.29	0.78	0.75	0.11	0.74	0.02	0.05	0.08	0.24	0.21	0.36	0.60	0.36	-0.10
BM43	0.15	0.21	0.62	0.17	0.34	0.55	0.54	0.41	0.68	0.28	0.43	0.67	0.00	0.40	0.27	0.72	0.71	0.02	0.55	-0.07
BM44	0.06	0.67	0.80	-0.16	0.56	0.86	0.29	0.30	0.25	0.14	0.13	0.03	0.09	0.54	-0.13	0.08	0.33	0.53	0.36	0.02
BM45	0.02	0.58	0.48	0.54	0.57	0.44	0.23	0.47	0.21	0.38	0.43	0.33	0.09	0.35	0.08	0.35	0.66	0.48	0.52	-0.25
BM46	0.14	0.59	0.74	0.61	0.39	0.60	0.47	0.54	0.21	0.79	0.60	0.29	0.10	0.13	0.22	-0.16	0.26	0.72	-0.06	-0.18
BM47	-0.11	0.72	0.11	0.09	0.73	0.59	0.02	-0.04	0.57	0.47	0.08	0.46	0.02	-0.04	-0.11	-0.14	0.59	0.03	0.26	0.30
BM48	0.20	0.71	0.69	0.20	0.21	0.64	0.78	0.16	-0.03	-0.09	0.03	0.27	0.08	0.10	0.24	-0.04	0.19	-0.13	0.10	0.39
BM49	0.32	0.31	0.76	0.48	0.05	0.75	0.49	0.72	0.18	-0.06	0.60	0.25	0.10	0.54	0.31	0.04	0.22	0.28	0.63	-0.01
BM50	0.26	0.52	0.46	0.41	0.23	0.79	0.57	0.25	0.51	0.41	0.17	0.49	-0.02	0.30	0.55	0.25	0.67	-0.01	0.01	-0.16
BM51	0.66	0.58	0.59	0.78	0.51	0.78	0.85	0.38	0.22	-0.13	0.54	0.33	-0.07	0.47	0.25	-0.05	0.26	0.24	-0.07	0.38
BM52	0.09	0.64	0.61	0.45	0.61	0.80	0.38	0.61	0.41	-0.11	0.85	0.67	0.58	0.44	0.45	0.17	0.32	0.04	0.32	0.13
BM53	0.36	0.50	0.63	0.73	0.22	0.77	0.44	0.55	0.33	0.16	0.74	0.36	0.34	0.41	0.28	0.20	0.44	-0.23	-0.01	-0.06
BM54	0.24	0.23	0.51	0.14	0.59	0.49	0.04	0.13	0.63	0.20	0.68	0.37	-0.03	0.02	0.51	-0.16	0.10	0.04	0.64	0.13
BM55	0.11	0.33	0.34	0.01	0.57	0.73	-0.28	0.27	0.44	0.20	0.20	0.41	0.05	0.32	0.19	0.69	0.05	0.11	0.42	-0.05
BM56	0.10	0.39	0.32	-0.09	0.18	0.43	-0.08	0.12	0.38	0.48	0.19	0.55	0.58	0.61	0.54	-0.15	0.45	0.37	-0.06	-0.09
BM57	0.10	0.26	0.32	0.29	0.03	0.74	0.17	0.53	0.49	-0.07	0.82	0.55	0.34	0.19	0.01	0.02	0.06	0.02	0.20	0.17
BM58	0.26	0.06	0.03	0.10	0.28	0.46	0.60	0.16	0.31	0.39	0.43	0.28	0.25	0.09	0.34	-0.16	0.44	0.06	0.67	0.35

Supplementary Table 15 continued

	Component ICs													*Noise ICs*					
	IC01	IC03	IC05	IC06	IC07	IC08	IC09	IC11	IC14	IC15	IC18	IC19	IC20	IC02	IC04	IC10	IC12	IC13	IC16	IC17
BM59	-0.03	0.30	0.38	0.20	0.54	0.15	0.11	-0.01	0.12	0.40	0.65	-0.03	0.40	0.57	0.10	0.69	0.50	0.32	0.30	0.12
BM60	0.07	0.39	0.03	0.31	0.44	0.61	0.09	0.19	0.50	0.50	0.61	0.24	-0.02	0.39	0.63	0.36	0.49	0.10	0.18	0.10
BM61	0.70	0.61	0.68	0.65	-0.04	0.80	0.27	0.62	0.26	0.27	0.35	0.27	-0.13	0.63	0.74	0.07	0.31	0.02	0.17	0.10
BM62	0.19	0.76	0.59	0.30	0.55	0.38	0.59	0.25	0.61	0.42	0.16	0.14	-0.08	0.45	0.00	0.06	0.51	0.12	0.40	0.12
BM63	-0.01	0.66	0.66	0.08	0.53	0.75	0.05	-0.02	0.09	0.39	0.74	0.41	-0.09	0.39	0.06	-0.25	0.51	0.00	0.31	0.12
BM64	0.01	0.69	0.63	0.34	-0.04	0.58	0.48	0.54	0.51	0.56	0.46	0.21	0.18	0.33	0.24	0.13	0.37	-0.17	0.55	0.05
BM65	0.21	0.24	0.01	-0.03	0.45	0.04	0.03	-0.06	0.03	0.58	0.21	0.10	-0.08	0.25	0.11	-0.02	0.49	-0.03	0.22	0.27
BM66	0.56	0.00	0.40	0.21	0.31	0.55	0.25	0.23	0.15	0.53	0.74	-0.02	0.16	0.55	0.30	-0.10	0.49	0.06	0.33	0.42
BM67	-0.08	0.59	0.10	0.10	-0.09	-0.16	0.13	0.02	0.04	0.15	0.10	0.18	0.24	0.50	0.36	0.12	0.75	0.42	0.25	0.36
BM68	0.11	0.74	0.68	0.33	0.32	0.82	0.59	0.55	0.00	0.23	0.44	0.34	0.12	0.44	0.22	0.47	0.38	0.44	0.49	0.05
BM69	0.37	0.74	0.42	0.74	0.48	0.62	0.53	0.41	0.26	0.16	0.71	0.31	0.46	0.34	0.10	0.32	0.59	0.20	0.29	0.01
BM70	-0.10	0.28	0.63	0.42	0.12	0.43	0.23	0.65	0.33	0.12	0.60	0.29	0.27	0.76	0.46	0.15	0.60	0.28	0.11	-0.19

Supplementary Table 16. Between-session ICC values for ICN$_i$ Spatial Involvement (I$_i$) values for the NYU-TRT group ICA data atlased using the BRAINMAP70 atlas. *See Supplementary Table 1 for the description of the atlas ICNs (BM01-BM70). For visual representation see Supplementary Figure 7 panel E.*

	Component ICs													*Noise ICs*						
	IC01	IC03	IC05	IC06	IC07	IC08	IC09	IC11	IC14	IC15	IC18	IC19	IC20	IC02	IC04	IC10	IC12	IC13	IC16	IC17
BM01	0.69	0.57	-0.13	0.03	0.09	0.39	-0.12	0.28	0.13	0.04	0.33	0.40	-0.02	0.72	0.24	-0.06	0.08	0.33	-0.24	0.16
BM02	0.50	0.48	0.25	0.01	0.01	0.18	0.20	-0.01	0.16	0.39	0.28	0.44	-0.14	0.78	0.36	0.27	0.25	0.41	0.43	0.47
BM03	0.56	0.04	0.07	0.42	0.21	0.35	-0.07	-0.07	0.03	0.27	0.31	0.32	0.02	0.72	0.43	0.15	0.46	-0.06	-0.07	0.00
BM04	0.50	0.21	-0.09	0.42	0.43	0.09	0.31	-0.14	0.21	0.18	0.45	0.46	-0.08	0.72	0.35	0.28	0.30	0.37	-0.06	-0.07
BM05	0.56	0.64	0.15	0.48	0.22	0.24	0.24	0.00	0.27	0.30	0.47	0.31	0.01	0.37	0.10	0.12	0.42	-0.03	0.32	0.06
BM06	0.27	0.46	0.54	0.51	0.59	0.42	0.43	0.64	0.06	0.41	0.30	0.49	0.25	0.25	0.17	0.15	0.08	0.27	0.20	-0.02
BM07	0.65	0.59	0.34	0.47	0.37	0.35	0.25	0.79	0.50	0.01	0.51	0.07	0.01	0.52	0.40	-0.09	0.52	0.42	-0.10	0.37
BM08	0.40	0.38	0.53	0.16	0.26	-0.01	0.50	0.16	0.08	0.17	0.28	0.44	0.60	0.26	-0.03	-0.10	0.84	0.26	0.47	0.17
BM09	0.64	0.64	0.04	0.41	-0.18	0.18	0.71	0.03	-0.08	-0.01	0.54	0.28	0.47	-0.05	0.58	0.17	0.41	0.41	0.29	0.17
BM10	0.50	0.43	-0.08	0.41	0.16	0.14	0.51	0.49	0.12	-0.22	0.20	-0.11	0.03	0.33	0.16	0.12	0.23	0.29	0.19	-0.28
BM11	0.20	0.34	0.43	0.14	0.29	0.30	0.48	0.44	-0.14	0.24	0.49	-0.21	0.15	0.40	0.34	0.17	-0.15	0.53	-0.10	-0.17
BM12	0.24	0.43	0.75	0.51	0.44	0.23	0.40	0.60	0.17	0.03	0.37	0.47	-0.01	0.66	0.52	0.24	0.55	0.39	0.04	0.12
BM13	0.56	-0.11	0.35	0.12	0.26	0.31	0.61	0.29	0.24	0.47	0.80	0.06	0.14	-0.19	0.42	0.23	0.46	0.73	0.20	0.44
BM14	0.04	0.48	0.07	0.49	0.39	0.13	0.06	-0.05	0.55	0.22	0.40	0.27	0.11	0.53	0.12	-0.15	0.22	0.32	0.16	0.44
BM15	0.11	0.31	0.43	0.04	0.39	0.14	0.33	0.10	0.08	0.20	0.07	0.48	-0.23	0.28	-0.12	0.16	0.24	0.55	0.37	0.26
BM16	0.32	0.30	0.30	0.21	0.29	0.29	0.16	0.36	-0.05	0.49	0.41	0.18	0.36	0.14	0.03	0.03	0.17	0.06	0.11	-0.09
BM17	0.25	0.10	0.04	0.40	0.56	0.57	0.57	0.23	0.44	0.13	0.32	0.17	-0.25	0.47	0.26	0.36	0.05	0.55	0.04	-0.19
BM18	0.41	0.54	0.39	-0.11	0.06	0.62	0.00	0.67	0.35	0.30	0.63	0.58	0.19	0.27	0.17	0.36	0.17	0.44	0.18	0.36
BM19	0.09	0.78	0.24	-0.11	0.44	0.46	0.58	0.37	0.28	0.05	0.33	0.66	0.28	-0.05	0.23	0.07	0.13	0.75	-0.02	0.06
BM20	0.51	0.26	0.04	-0.09	-0.05	0.17	0.20	0.24	0.42	-0.04	0.36	0.16	0.00	0.34	-0.05	-0.09	0.14	0.06	0.24	-0.05
BM21	0.15	0.62	0.52	0.45	-0.05	0.42	-0.03	0.22	0.42	-0.14	-0.10	0.13	-0.07	0.22	0.33	0.49	0.06	0.00	-0.07	0.04
BM22	-0.12	0.47	0.41	0.15	-0.07	0.25	-0.12	0.54	0.30	-0.01	-0.16	0.59	0.35	0.30	0.05	-0.02	0.27	0.27	0.19	-0.03
BM23	0.19	0.41	0.03	0.31	0.32	0.34	0.08	-0.02	0.40	0.60	0.33	0.06	0.14	0.21	0.62	-0.06	0.60	0.42	0.71	0.43
BM24	0.13	0.49	0.08	0.20	0.35	0.39	0.19	0.05	0.30	-0.02	0.49	0.25	0.11	0.49	0.08	-0.02	0.15	0.29	0.41	0.18
BM25	0.22	0.24	0.69	0.14	-0.03	0.35	0.03	0.38	0.14	0.04	0.23	0.25	0.37	0.02	0.62	0.15	-0.28	0.10	-0.13	0.23
BM26	0.25	0.70	-0.06	-0.09	0.32	0.41	0.48	-0.17	-0.07	0.06	0.09	0.46	-0.03	0.51	-0.05	0.45	0.35	-0.08	0.14	-0.03
BM27	0.10	0.26	0.74	0.11	0.12	0.41	0.50	0.11	-0.06	0.32	0.23	0.31	0.26	0.23	0.19	-0.15	-0.01	0.20	-0.03	-0.02

Supplementary Table 16 continued

	Component ICs													*Noise ICs*						
	IC01	IC03	IC05	IC06	IC07	IC08	IC09	IC11	IC14	IC15	IC18	IC19	IC20	IC02	IC04	IC10	IC12	IC13	IC16	IC17
BM28	0.56	0.64	0.12	0.02	0.12	0.32	0.24	0.56	0.36	0.08	0.44	-0.24	0.33	0.16	0.31	0.33	0.00	0.44	-0.26	-0.07
BM29	0.41	0.44	0.72	0.58	0.31	0.41	0.34	0.54	-0.04	0.35	0.25	-0.04	0.39	-0.07	0.08	0.26	0.15	-0.05	0.21	0.10
BM30	0.42	0.43	0.27	-0.11	0.03	0.63	0.39	0.22	-0.04	-0.02	0.07	0.13	0.22	0.25	-0.02	-0.18	0.25	0.15	0.04	-0.11
BM31	0.40	0.79	0.03	0.06	0.19	0.60	0.00	0.36	0.28	0.21	0.40	0.40	0.40	0.32	0.43	-0.07	0.21	-0.07	0.28	0.16
BM32	0.66	0.71	0.20	0.61	0.54	0.20	0.34	0.02	0.19	0.50	0.16	0.32	-0.07	0.31	0.36	0.02	0.47	-0.01	0.55	0.04
BM33	0.00	0.64	0.15	0.37	0.26	0.40	0.50	-0.19	0.29	0.61	0.31	0.25	0.31	0.64	0.42	0.66	0.42	0.19	-0.04	0.09
BM34	0.08	0.34	0.14	0.38	0.37	-0.12	0.33	0.29	0.12	0.14	0.14	0.21	0.21	0.34	0.58	0.00	0.33	0.23	-0.03	0.00
BM35	0.47	0.45	0.10	0.54	0.35	-0.02	-0.26	0.02	0.25	-0.13	0.29	0.51	0.18	0.57	-0.02	0.01	0.25	0.40	0.63	0.34
BM36	0.36	0.15	0.39	0.53	0.34	0.38	0.02	0.46	0.03	0.07	0.12	0.22	-0.12	0.45	-0.13	0.08	0.03	0.10	0.18	0.10
BM37	0.79	0.25	0.75	0.29	0.42	0.14	0.60	0.70	0.57	0.32	0.25	-0.14	-0.03	0.28	0.51	-0.26	0.26	0.23	0.09	0.03
BM38	0.20	0.76	0.31	0.29	0.51	0.60	0.54	0.41	0.27	0.29	-0.03	0.06	0.06	0.60	0.35	-0.19	0.50	0.28	0.17	0.17
BM39	0.16	0.59	0.30	-0.02	0.11	0.34	0.03	0.23	0.32	0.13	0.54	0.25	0.11	0.68	0.11	0.04	0.42	0.70	0.02	0.44
BM40	0.40	0.25	-0.20	0.71	0.39	-0.27	-0.02	0.03	0.28	0.27	-0.17	0.10	0.32	0.58	0.01	0.21	0.39	0.22	0.10	0.12
BM41	0.14	0.84	0.65	-0.30	0.35	0.19	0.47	0.33	0.38	0.06	0.30	0.06	0.13	0.70	0.19	-0.16	-0.14	0.50	0.01	0.59
BM42	0.53	0.73	0.47	0.46	0.67	0.55	0.48	-0.03	-0.12	0.12	0.51	0.13	0.16	0.51	0.29	0.34	0.41	0.14	0.06	-0.10
BM43	0.01	0.60	0.10	0.29	0.23	0.31	0.59	0.30	0.25	0.28	0.17	0.47	0.08	0.47	-0.12	0.58	0.28	0.03	0.03	0.28
BM44	-0.11	0.69	0.61	0.32	0.50	0.67	0.50	0.27	0.33	0.28	0.55	0.44	0.18	0.65	0.30	-0.11	0.60	0.54	-0.11	-0.25
BM45	0.17	0.47	0.33	0.41	0.58	0.42	0.02	-0.17	0.21	0.26	0.60	0.36	-0.24	0.41	0.28	0.23	0.42	0.32	0.46	0.20
BM46	0.49	0.44	0.62	0.60	0.22	0.57	0.36	0.74	0.02	0.25	0.09	0.28	0.04	0.18	0.74	0.05	0.65	0.37	0.24	-0.08
BM47	0.14	0.36	0.37	0.13	0.58	0.42	-0.09	-0.24	0.45	0.45	0.26	0.32	0.06	0.37	0.13	0.10	0.77	0.68	0.26	0.03
BM48	-0.13	0.09	0.44	0.02	0.05	0.44	0.44	0.35	-0.04	-0.13	0.41	-0.13	0.02	-0.13	-0.12	-0.12	0.00	0.31	0.29	-0.07
BM49	0.64	0.44	0.60	0.47	-0.06	0.15	0.32	0.01	0.07	-0.17	-0.03	0.08	-0.19	0.01	0.13	0.02	0.12	0.11	-0.20	-0.17
BM50	0.48	0.18	0.36	0.06	0.42	0.43	0.62	0.18	0.13	0.38	0.50	0.50	0.16	0.28	0.39	0.41	0.23	0.45	0.27	0.50
BM51	0.29	0.18	0.64	0.24	0.58	0.49	0.67	-0.13	-0.08	-0.10	0.06	0.21	0.12	0.05	0.24	0.00	0.45	0.12	-0.03	0.06
BM52	0.38	0.68	0.70	0.06	0.50	0.52	-0.01	0.66	0.43	0.14	0.32	0.30	0.19	0.48	0.29	0.13	0.37	0.10	0.13	0.36
BM53	0.63	0.22	0.38	0.41	0.04	0.32	0.03	-0.01	0.10	0.15	0.30	0.25	-0.01	0.33	0.46	0.21	-0.07	0.13	-0.18	0.23
BM54	0.23	0.55	0.20	0.38	0.37	0.71	0.41	0.44	0.33	0.44	0.24	0.35	-0.03	0.11	0.48	-0.11	0.51	0.25	0.56	0.51
BM55	0.39	0.27	-0.06	-0.03	0.45	0.55	-0.06	0.25	0.52	0.07	0.35	0.47	0.61	0.09	0.06	0.41	0.77	0.19	-0.11	-0.06

Supplementary Table 16 continued

	Component ICs													*Noise ICs*						
	IC01	IC03	IC05	IC06	IC07	IC08	IC09	IC11	IC14	IC15	IC18	IC19	IC20	IC02	IC04	IC10	IC12	IC13	IC16	IC17
BM56	0.08	0.32	0.03	-0.03	0.32	0.14	0.08	-0.03	0.00	0.30	0.15	0.44	-0.11	0.64	0.56	0.19	0.25	0.67	0.25	0.32
BM57	0.66	0.20	-0.15	0.18	0.31	0.58	0.17	0.29	0.44	0.36	0.38	0.23	0.15	0.21	0.19	-0.15	0.00	0.32	0.17	0.44
BM58	0.18	0.11	-0.23	0.17	0.26	0.38	0.66	0.31	0.38	-0.06	0.55	0.40	0.06	0.15	0.63	-0.05	0.51	0.45	0.52	0.52
BM59	0.13	0.06	0.23	0.38	0.02	0.23	0.13	-0.14	0.26	0.27	-0.02	0.23	0.13	0.63	-0.12	0.20	0.35	0.36	-0.04	0.24
BM60	0.06	0.12	-0.03	0.15	0.13	0.15	-0.05	0.21	0.33	0.55	0.61	0.08	0.14	0.35	0.69	0.17	0.08	0.65	0.00	0.19
BM61	0.52	0.51	0.48	0.31	-0.01	0.43	0.22	0.30	-0.14	-0.14	0.36	0.47	0.05	0.73	0.46	0.53	0.32	0.32	0.18	0.23
BM62	0.42	0.67	0.57	0.46	0.37	0.21	0.69	0.25	0.38	0.31	0.24	0.05	-0.05	0.18	-0.03	0.04	0.14	0.28	0.07	0.25
BM63	-0.09	0.20	0.11	0.07	0.27	0.52	-0.14	0.22	0.17	0.67	0.51	0.04	0.05	0.23	0.32	0.18	0.44	0.24	-0.06	-0.01
BM64	0.25	0.63	0.58	0.19	0.49	0.11	0.48	0.41	0.08	-0.15	0.18	0.01	-0.34	0.19	-0.33	-0.17	0.01	0.19	0.20	0.04
BM65	0.26	0.28	-0.15	0.43	0.04	-0.22	-0.06	0.42	-0.01	0.35	-0.02	0.21	0.27	0.17	0.18	0.09	0.24	0.48	0.01	0.07
BM66	0.73	-0.05	0.09	0.08	0.27	0.43	0.41	-0.02	0.35	0.59	0.50	0.07	0.08	0.61	0.04	0.22	0.61	0.25	0.32	0.13
BM67	0.15	0.40	0.02	0.13	0.08	0.35	-0.13	0.09	0.37	0.24	0.19	0.04	0.18	0.36	0.57	-0.04	0.44	0.30	-0.15	0.27
BM68	0.63	0.78	0.57	-0.17	0.07	0.24	0.55	0.43	0.08	0.08	0.21	0.10	-0.02	0.37	0.24	0.31	0.27	0.06	0.32	-0.09
BM69	0.40	0.51	0.21	0.44	0.32	0.48	0.38	0.41	0.14	0.12	0.50	0.24	0.24	0.47	0.33	-0.14	0.45	0.35	0.03	0.09
BM70	0.45	0.71	0.22	0.12	0.27	0.26	0.32	-0.03	0.37	0.12	0.22	0.56	0.17	0.60	0.43	0.30	0.21	0.39	0.02	-0.06

Supplementary Table 17. Within-session ICC values for Normalised Mean ICN$_i$ Activation (MA$_{N,i}$) values for the NYU-TRT group ICA data atlased using the SMITH10 atlas. *ICN01 visual (medial), ICN02 visual (occipital pole), ICN03 visual (lateral), ICN04 DMN, ICN05 cerebellum, ICN06 sensori-motor, ICN07 auditory, ICN08 executive control, ICN09 fronto-parietal (perception, somesthesis, pain), ICN10 fronto-parietal (cognition-language). For visual representation see Figure 7 panel B.*

	Component ICs													*Noise ICs*					
	IC01	IC03	IC05	IC06	IC07	IC08	IC09	IC11	IC14	IC15	IC18	IC19	IC20	IC02	IC04	IC10	IC12	IC13	IC16	IC17
ICN01	0.75	0.81	0.54	0.79	0.59	0.71	0.45	0.61	0.51	0.44	0.59	0.46	-0.29	0.81	0.75	0.60	0.31	0.54	0.59	0.34
ICN02	0.52	0.14	0.59	0.64	0.64	0.71	0.64	0.56	0.61	0.56	0.58	0.53	0.31	0.76	0.80	0.38	0.67	0.68	0.71	0.30
ICN03	0.62	0.69	0.75	0.71	0.50	0.66	0.45	0.55	0.72	0.54	0.73	0.60	0.51	0.53	0.72	0.14	0.50	0.62	0.52	0.11
ICN04	0.71	0.61	0.56	0.77	0.27	0.71	0.55	0.32	0.66	0.54	0.57	0.73	0.52	0.39	0.62	0.51	0.63	0.70	0.71	0.46
ICN05	0.71	0.73	0.78	0.30	0.78	0.73	0.71	0.63	0.55	0.62	0.69	0.47	0.26	0.63	0.60	0.33	0.68	0.50	0.69	0.78
ICN06	0.63	0.70	0.76	0.64	0.45	0.84	0.69	0.40	0.69	0.58	0.81	0.74	0.59	0.69	0.49	0.69	0.21	0.55	0.83	0.71
ICN07	0.78	0.69	0.63	0.78	0.43	0.81	0.34	0.70	0.72	0.59	0.88	0.73	0.62	0.62	0.84	0.40	0.61	0.28	0.73	0.67
ICN08	0.74	0.63	0.61	0.76	0.78	0.87	0.52	0.52	0.59	0.50	0.81	0.69	0.53	0.71	0.62	0.65	0.63	0.54	0.66	0.44
ICN09	0.85	0.60	0.53	0.74	0.70	0.82	0.50	0.46	0.73	0.51	0.74	0.80	0.65	0.66	0.77	0.18	0.73	0.64	0.65	0.68
ICN10	0.68	0.73	0.70	0.79	0.72	0.91	0.53	0.55	0.75	0.72	0.83	0.76	0.56	0.67	0.82	0.61	0.64	0.72	0.64	0.48

Supplementary Table 18. Between-session ICC values for Normalised Mean ICN$_i$ Activation (MA$_{N,i}$) values for the NYU-TRT group ICA data atlased using the SMITH10 atlas. *ICN01 visual (medial), ICN02 visual (occipital pole), ICN03 visual (lateral), ICN04 DMN, ICN05 cerebellum, ICN06 sensori-motor, ICN07 auditory, ICN08 executive control, ICN09 fronto-parietal (perception, somesthesis, pain), ICN10 fronto-parietal (cognition-language). For visual representation see Figure 7 panel F.*

	Component ICs													*Noise ICs*						
	IC01	IC03	IC05	IC06	IC07	IC08	IC09	IC11	IC14	IC15	IC18	IC19	IC20	IC02	IC04	IC10	IC12	IC13	IC16	IC17
ICN01	0.54	0.45	0.48	0.02	-0.12	0.21	0.34	0.68	0.22	0.00	-0.01	0.20	0.24	0.56	0.39	0.01	0.38	0.01	0.50	0.22
ICN02	0.25	0.14	-0.11	0.06	-0.02	0.16	0.01	0.01	0.38	0.16	0.25	0.06	-0.02	0.48	-0.23	0.10	0.63	0.26	0.07	0.18
ICN03	0.01	0.42	0.42	0.05	-0.20	0.34	0.31	0.02	0.14	-0.31	0.06	0.37	0.28	0.52	-0.02	0.16	0.30	0.08	0.14	0.19
ICN04	0.28	0.27	0.40	-0.06	0.16	0.00	0.22	0.04	0.07	-0.03	-0.06	0.30	0.30	0.18	0.38	0.29	0.36	-0.02	0.26	0.14
ICN05	0.36	-0.07	0.36	0.24	0.00	-0.09	0.05	-0.12	0.14	0.34	0.45	0.38	0.27	0.34	0.06	0.23	0.40	0.38	0.35	0.06
ICN06	-0.09	0.10	0.46	-0.13	0.18	0.17	0.30	0.03	0.00	0.05	-0.04	0.02	0.02	0.31	0.09	0.15	0.17	-0.07	0.09	0.04
ICN07	-0.01	0.20	0.34	0.30	-0.28	0.32	-0.23	0.05	0.45	-0.17	0.20	0.12	0.41	0.37	0.09	0.02	0.52	-0.05	0.06	0.25
ICN08	0.18	0.37	0.46	0.04	0.32	0.19	0.13	0.21	0.12	-0.03	-0.04	0.09	0.37	0.17	0.40	0.30	0.46	0.02	0.07	0.04
ICN09	0.02	0.04	0.31	0.01	-0.18	-0.02	0.31	0.28	0.08	-0.08	0.09	0.12	0.42	0.04	0.01	0.33	0.43	0.20	0.09	-0.02
ICN10	0.07	0.21	0.07	0.10	0.17	0.17	0.22	0.14	0.38	0.19	0.18	0.16	0.30	0.29	0.09	0.22	0.53	-0.15	0.08	-0.28

Supplementary Table 19. Within-session ICC values for Normalised Mean ICN$_i$ Activation (MA$_{N,i}$) values for the NYU-TRT group ICA data atlased using the BRAINMAP20 atlas. *BM1: limbic and medial-temporal areas, BM2: subgenual ACC and OFC, BM3: bilateral BG and thalamus, BM4: bilateral anterior insula/frontal opercula and the anterior aspect of the body of the cingulate gyrus, BM5: midbrain, BM6: superior and middle frontal gyri, BM7: middle frontal gyri and superior parietal lobules, BM8: ventral precentral gyri, central sulci, postcentral gyri, superior and inferior cerebellum, BM9: superior parietal lobule, BM10: middle and inferior temporal gyri, BM11: lateral posterior occipital cortex, BM12: medial posterior occipital cortex, BM13: medial prefrontal and posterior cingulate/precuneus areas, DMN, BM14: cerebellum, BM15: right-lateralized fronto-parietal regions, BM16: transverse temporal gyri, BM17: dorsal precentral gyri, central sulci, postcentral gyri, superior and inferior cerebellum, BM18: left-lateralized fronto-parietal regions, BM19-20: artefactual. For visual representation see Figure 7 panel J.*

	Component ICs													*Noise ICs*					
	IC01	IC03	IC05	IC06	IC07	IC08	IC09	IC11	IC14	IC15	IC18	IC19	IC20	IC02	IC04	IC10	IC12	IC13	IC16	IC17
BM01	0.65	0.70	0.77	0.83	0.71	0.87	0.62	0.57	0.71	0.37	0.61	0.78	0.44	0.80	0.59	0.53	0.74	0.60	0.85	0.30
BM02	0.56	0.76	0.65	0.76	0.31	0.86	0.48	0.65	0.65	0.46	0.76	0.73	0.41	0.78	0.68	0.00	0.54	0.66	0.71	0.40
BM03	0.89	0.79	0.25	0.41	0.72	0.85	0.78	0.56	0.69	0.41	0.28	0.75	0.64	0.47	0.68	0.25	0.80	0.20	0.88	0.55
BM04	0.71	0.61	0.71	0.76	0.72	0.82	0.60	0.54	0.68	0.30	0.84	0.75	0.69	0.65	0.86	0.14	0.68	0.51	0.63	0.65
BM05	0.80	0.70	0.83	0.46	0.74	0.82	0.63	0.49	0.71	0.56	0.65	0.63	0.57	0.66	0.73	0.46	0.70	0.63	0.68	0.57
BM06	0.80	0.82	0.55	0.30	0.19	0.72	0.67	0.63	0.72	0.49	0.77	0.71	0.27	0.82	0.23	0.45	0.50	0.43	0.77	0.74
BM07	0.75	0.62	0.76	0.76	0.61	0.74	0.54	0.62	0.58	0.52	0.82	0.77	0.63	0.71	0.70	0.38	0.48	0.78	0.47	0.52
BM08	0.78	0.67	0.86	0.80	0.35	0.73	0.75	0.70	0.79	0.62	0.77	0.69	0.70	0.90	0.65	0.60	0.65	0.66	0.82	0.40
BM09	0.39	0.68	0.42	0.65	0.22	0.74	-0.08	0.71	0.67	0.55	0.73	0.32	0.04	0.68	-0.01	0.59	0.21	0.65	0.82	0.53
BM10	0.75	0.65	0.81	0.79	0.52	0.75	0.59	0.68	0.66	0.42	0.72	0.67	0.46	0.72	0.72	0.24	0.62	0.62	0.67	0.30
BM11	0.57	0.13	0.56	0.85	0.79	0.64	0.66	0.70	0.66	0.54	0.68	0.62	0.50	0.76	0.81	0.06	0.58	0.76	0.76	0.11
BM12	0.76	0.72	0.89	0.89	0.71	0.76	0.74	0.68	0.65	0.45	0.77	0.59	0.54	0.73	0.62	0.39	0.84	0.72	0.75	0.45
BM13	0.66	0.74	0.54	0.73	0.56	0.77	0.79	0.49	0.77	0.53	0.70	0.80	0.63	0.55	0.57	0.64	0.85	0.72	0.71	0.43
BM14	0.60	0.21	0.72	0.66	0.60	0.82	0.74	0.71	0.76	0.57	0.74	0.37	0.57	0.69	0.46	0.59	0.56	0.60	0.85	0.39
BM15	0.74	0.71	0.56	0.76	0.68	0.82	0.50	0.60	0.72	0.36	0.78	0.78	0.56	0.62	0.79	0.21	0.65	0.78	0.63	0.54
BM16	0.79	0.79	0.53	0.73	0.41	0.76	0.83	0.69	0.71	0.48	0.34	0.82	0.64	0.69	0.69	0.65	0.78	0.48	0.67	0.73
BM17	0.25	0.62	0.69	0.68	0.58	0.85	0.62	0.73	0.75	0.59	0.88	0.82	0.79	0.76	0.19	0.61	0.63	0.64	0.75	0.62
BM18	0.69	0.68	0.87	0.11	0.55	0.88	0.58	0.54	0.66	0.39	0.90	0.75	0.62	0.87	0.80	0.31	0.83	0.75	0.62	0.62
BM19	0.69	0.77	0.72	0.72	0.69	0.77	0.71	0.74	0.80	0.37	0.81	0.80	0.60	0.86	0.68	0.19	0.76	0.58	0.71	0.39
BM20	0.64	0.68	0.65	0.85	0.59	0.84	0.62	0.65	0.74	0.53	0.78	0.80	0.77	0.77	0.54	0.25	0.83	0.75	0.66	0.73

Supplementary Table 20. Between-session ICC values for Normalised Mean ICN$_i$ Activation (MA$_{N,i}$) values for the NYU-TRT group ICA data atlased using the BRAINMAP20 atlas. *BM1: limbic and medial-temporal areas, BM2: subgenual ACC and OFC, BM3: bilateral BG and thalamus, BM4: bilateral anterior insula/frontal opercula and the anterior aspect of the body of the cingulate gyrus, BM5: midbrain, BM6: superior and middle frontal gyri, BM7: middle frontal gyri and superior parietal lobules, BM8: ventral precentral gyri, central sulci, postcentral gyri, superior and inferior cerebellum, BM9: superior parietal lobule, BM10: middle and inferior temporal gyri, BM11: lateral posterior occipital cortex, BM12: medial posterior occipital cortex, BM13: medial prefrontal and posterior cingulate/precuneus areas, DMN, BM14: cerebellum, BM15: right-lateralized fronto-parietal regions, BM16: transverse temporal gyri, BM17: dorsal precentral gyri, central sulci, postcentral gyri, superior and inferior cerebellum, BM18: left-lateralized fronto-parietal regions, BM19-20: artefactual. For visual representation see Figure 7 panel N.*

	Component ICs													*Noise ICs*						
	IC01	IC03	IC05	IC06	IC07	IC08	IC09	IC11	IC14	IC15	IC18	IC19	IC20	IC02	IC04	IC10	IC12	IC13	IC16	IC17
BM01	-0.03	0.23	0.46	0.14	0.05	0.15	0.27	-0.02	0.27	-0.14	0.00	0.27	0.51	0.02	-0.41	-0.06	0.24	0.15	-0.06	0.44
BM02	-0.01	0.43	0.36	0.31	-0.12	0.23	0.42	0.09	0.37	-0.16	-0.06	0.33	0.45	0.16	-0.22	0.46	0.42	0.08	0.23	0.00
BM03	0.07	0.35	-0.17	-0.09	0.10	0.25	0.27	0.00	0.23	-0.06	0.38	0.07	0.25	-0.06	0.10	0.18	0.44	-0.07	0.39	0.23
BM04	0.06	0.02	0.51	0.00	0.44	0.24	-0.11	0.24	0.28	0.11	0.04	0.18	0.24	0.59	-0.03	-0.12	0.44	0.13	0.03	0.05
BM05	0.24	0.29	0.29	0.36	0.12	0.02	0.09	0.18	0.20	0.28	0.16	0.21	0.39	0.29	0.27	0.10	0.15	0.57	0.19	0.37
BM06	0.08	-0.06	0.43	0.46	0.29	0.13	0.40	0.28	0.09	-0.12	0.29	-0.03	0.11	0.23	0.18	0.23	0.28	0.18	0.16	-0.03
BM07	0.25	0.36	0.43	0.34	0.15	0.12	0.62	0.30	0.13	-0.25	0.11	0.36	0.51	0.20	0.08	0.34	0.29	0.26	0.02	-0.05
BM08	0.20	0.15	0.44	0.25	0.25	0.11	0.44	0.24	0.01	-0.02	0.02	0.42	0.38	0.23	0.06	0.14	0.64	0.22	-0.15	-0.02
BM09	0.35	0.14	0.08	0.36	0.39	0.00	-0.14	0.26	0.11	-0.14	-0.08	0.08	-0.09	0.43	0.02	-0.07	0.30	0.35	0.00	0.00
BM10	0.09	0.54	0.52	0.13	0.06	0.15	0.44	0.19	0.23	-0.24	0.06	0.25	0.17	0.38	0.10	0.25	0.09	-0.06	0.06	-0.26
BM11	0.30	0.07	-0.14	0.33	0.14	0.20	-0.05	-0.11	0.46	0.35	0.21	0.55	0.27	0.41	-0.05	0.24	0.51	0.24	0.01	-0.06
BM12	0.49	0.58	0.17	-0.03	0.13	0.19	0.25	0.53	0.31	0.05	0.03	0.35	0.20	0.37	0.32	0.12	0.32	0.24	0.43	0.22
BM13	-0.12	0.43	0.39	0.06	0.26	0.49	0.43	0.11	0.29	-0.02	0.15	0.29	0.31	0.40	0.10	-0.06	0.30	-0.18	0.20	0.02
BM14	0.49	0.03	0.36	0.35	-0.01	-0.18	0.34	-0.10	0.22	0.39	0.40	0.28	0.19	0.57	0.19	-0.06	0.31	-0.18	0.52	-0.03
BM15	0.01	0.24	0.45	-0.05	0.26	0.01	0.36	0.26	0.07	-0.26	0.22	0.14	0.21	-0.24	0.13	0.54	0.48	0.27	-0.08	-0.16
BM16	0.10	0.14	0.26	0.38	-0.06	0.50	0.50	0.13	0.40	-0.18	0.14	0.12	0.33	0.22	0.05	0.38	0.29	0.03	0.29	-0.13
BM17	-0.22	-0.01	0.14	0.22	0.07	0.22	0.21	-0.16	0.07	0.17	0.04	0.13	0.01	0.14	0.12	0.26	0.30	0.01	0.04	0.03
BM18	0.18	0.02	0.31	0.15	0.01	0.01	0.47	0.17	-0.11	-0.17	0.00	0.11	0.28	-0.02	0.06	0.00	0.56	-0.21	-0.06	-0.11
BM19	0.06	0.08	0.11	0.04	-0.12	0.01	0.05	0.07	0.20	0.10	-0.32	0.37	0.32	0.56	-0.16	0.08	0.39	0.18	0.11	0.41
BM20	0.10	0.18	0.38	0.28	0.32	0.35	0.46	0.23	0.10	-0.05	-0.15	0.22	0.38	0.38	0.61	0.11	0.54	0.44	0.09	0.09

Supplementary Table 21. Within-session ICC values for Normalised Mean ICN$_i$ Activation (MA$_{N,i}$) values for the NYU-TRT group ICA data atlased using the BRAINMAP70 atlas. *See Supplementary Table 1 for the description of atlas ICNs (BM01-BM70). For visual representation see Supplementary Figure 7 panel B.*

	Component ICs													*Noise ICs*					
	IC01	IC03	IC05	IC06	IC07	IC08	IC09	IC11	IC14	IC15	IC18	IC19	IC20	IC02	IC04	IC10	IC12	IC13	IC16	IC17
BM01	0.61	0.69	0.47	0.74	0.54	0.44	-0.10	0.67	0.43	0.07	0.77	0.12	0.43	0.68	-0.09	0.14	0.24	0.66	0.60	-0.03
BM02	0.72	0.07	0.12	0.14	0.25	0.09	0.52	-0.06	0.45	0.22	-0.03	0.21	-0.07	0.91	0.26	0.04	0.07	0.48	0.31	0.05
BM03	0.55	0.05	0.32	0.80	0.76	0.56	0.48	0.71	0.65	0.57	0.65	0.33	0.40	0.79	0.81	0.16	0.55	0.75	0.70	0.62
BM04	0.61	0.51	0.64	0.74	0.70	0.69	0.57	0.22	0.68	0.46	0.73	0.59	0.34	0.62	0.15	0.41	0.59	0.39	0.62	-0.13
BM05	0.83	0.41	-0.08	0.14	0.07	0.10	0.08	-0.16	0.46	0.03	0.59	0.21	0.00	0.13	0.18	-0.08	0.36	-0.07	0.56	-0.10
BM06	0.36	-0.13	0.49	0.54	0.39	0.03	0.68	0.52	0.04	-0.05	0.65	0.33	-0.24	0.28	0.18	0.50	0.25	0.36	0.70	-0.06
BM07	0.57	0.63	0.54	0.75	0.40	0.21	0.75	0.63	0.28	0.14	0.73	0.64	0.30	0.72	0.53	0.22	0.24	0.38	0.49	0.60
BM08	0.13	0.62	0.74	0.67	0.13	0.09	0.43	0.56	0.62	-0.05	0.67	0.12	0.52	0.11	0.18	0.11	-0.11	-0.02	0.42	0.01
BM09	0.70	0.36	0.36	0.78	0.66	0.93	0.23	0.10	0.19	-0.20	0.71	0.68	0.02	-0.11	0.17	0.02	0.11	0.10	0.45	0.10
BM10	0.54	0.62	0.33	0.69	0.12	0.09	0.66	0.65	0.54	0.02	0.79	0.43	0.69	0.47	0.26	0.18	0.49	0.24	0.71	0.40
BM11	0.18	0.24	0.19	0.16	0.01	0.83	0.48	0.58	0.70	-0.17	0.28	0.44	0.40	0.56	0.77	0.32	0.54	0.16	0.67	0.68
BM12	0.37	0.47	0.22	0.29	0.44	0.31	0.31	0.68	0.51	-0.08	0.33	0.30	-0.15	0.37	0.11	0.65	0.60	0.13	0.36	0.07
BM13	0.31	0.10	0.27	0.56	0.41	0.17	0.58	0.60	0.63	0.44	0.12	0.12	0.41	0.18	0.24	-0.08	0.34	0.08	0.01	-0.24
BM14	0.00	0.72	-0.09	0.68	0.42	0.76	0.24	0.44	0.60	0.45	0.53	0.66	0.24	0.68	0.50	0.47	0.61	0.90	0.74	-0.04
BM15	-0.04	-0.16	0.51	0.29	0.25	0.68	0.14	0.48	0.08	-0.21	-0.11	0.64	0.17	0.43	-0.03	-0.02	0.36	0.06	0.77	0.07
BM16	0.25	0.05	0.38	0.09	0.64	0.48	0.37	0.14	0.17	-0.04	0.75	0.87	0.06	0.34	0.34	-0.12	0.33	-0.03	0.71	-0.17
BM17	0.09	-0.09	-0.23	0.11	0.45	0.77	0.44	0.56	0.39	-0.16	0.18	0.24	-0.15	0.13	0.15	0.34	0.32	0.59	-0.01	0.43
BM18	-0.14	0.73	0.08	-0.13	0.51	0.70	0.09	0.05	0.70	-0.08	0.54	0.81	0.27	0.78	0.63	0.48	0.53	0.21	0.20	0.30
BM19	0.01	0.54	0.63	0.57	0.53	0.78	0.77	0.13	0.75	0.38	0.68	0.56	0.31	0.64	-0.01	0.12	0.63	0.29	0.75	-0.17
BM20	0.06	0.73	0.61	0.15	0.52	0.72	0.55	0.47	0.55	-0.02	0.08	0.26	0.41	0.17	0.27	0.64	0.52	0.68	0.63	0.34
BM21	-0.15	0.79	0.61	0.44	-0.09	0.74	0.60	0.49	0.67	0.50	0.68	0.24	0.31	0.26	0.57	0.00	0.68	0.71	0.66	0.34
BM22	-0.02	0.61	0.38	0.15	0.36	0.66	0.23	0.31	0.38	0.13	0.64	-0.10	0.59	0.65	0.08	-0.16	0.40	0.43	0.56	0.23
BM23	0.13	0.77	0.45	0.10	0.23	0.76	0.12	0.63	0.55	0.19	0.07	0.12	0.39	0.72	0.64	0.76	0.37	0.70	0.80	-0.07
BM24	0.30	0.83	0.74	0.81	0.75	0.64	0.36	0.45	0.17	0.54	0.30	0.70	-0.18	0.76	0.55	0.13	0.10	0.35	0.82	0.08
BM25	-0.04	0.03	0.50	0.42	0.14	0.73	0.55	0.69	0.60	0.06	0.72	0.32	0.40	0.63	0.74	0.14	0.00	-0.24	0.64	0.58
BM26	-0.18	0.53	-0.06	0.10	-0.04	0.61	0.70	0.67	0.06	0.05	0.87	-0.06	0.14	0.01	0.61	-0.16	-0.15	-0.05	0.61	0.03
BM27	-0.20	0.31	0.58	0.00	0.24	0.71	0.62	0.56	0.55	0.34	-0.05	0.59	0.51	0.53	0.54	0.26	0.27	0.02	-0.03	0.35

Supplementary Table 21 continued

	Component ICs													*Noise ICs*						
	IC01	IC03	IC05	IC06	IC07	IC08	IC09	IC11	IC14	IC15	IC18	IC19	IC20	IC02	IC04	IC10	IC12	IC13	IC16	IC17
BM28	0.61	0.86	0.58	0.36	0.49	0.72	0.67	0.65	0.72	-0.02	0.59	0.65	0.65	0.48	0.82	0.32	0.68	0.60	0.56	-0.01
BM29	0.22	0.65	0.60	0.69	-0.03	0.32	0.55	0.16	0.14	-0.02	0.13	0.92	-0.02	0.12	0.42	0.59	-0.04	-0.05	0.54	0.17
BM30	0.56	0.60	0.59	-0.03	0.63	0.19	0.69	0.55	0.09	0.02	0.62	0.58	0.06	0.55	0.27	0.00	0.73	-0.01	0.65	0.74
BM31	0.65	0.69	0.60	-0.04	0.39	0.80	0.02	0.55	0.29	0.22	0.47	0.66	0.56	0.78	0.67	0.29	0.47	0.39	0.79	0.29
BM32	0.72	0.76	-0.09	0.79	0.18	0.76	0.26	0.32	0.58	0.42	0.21	0.57	0.21	0.75	0.59	0.83	0.59	0.12	0.62	-0.38
BM33	0.26	0.11	-0.13	0.34	0.42	0.19	0.72	0.03	0.29	0.47	0.60	0.31	0.62	0.43	0.43	-0.02	0.64	0.62	0.28	0.18
BM34	0.16	0.19	0.21	0.72	0.28	-0.08	0.28	0.17	0.60	0.01	0.72	0.10	0.56	0.13	0.52	0.26	0.37	-0.03	0.57	0.20
BM35	0.25	0.40	0.31	0.52	0.14	0.69	-0.13	0.69	0.08	-0.01	-0.13	0.27	0.25	0.58	0.28	0.60	0.23	0.43	0.66	0.58
BM36	-0.06	0.64	0.49	0.17	0.27	0.77	-0.23	0.51	0.54	0.26	0.84	0.36	-0.25	0.40	0.10	-0.02	0.21	0.05	0.79	0.29
BM37	0.54	0.44	0.54	0.77	0.47	0.70	0.54	0.38	0.19	0.92	0.33	0.48	0.52	0.61	0.30	0.22	0.22	0.18	0.04	0.18
BM38	0.18	0.57	0.41	0.60	0.88	0.74	0.51	0.42	0.67	0.37	0.32	0.05	0.30	0.05	0.14	-0.36	-0.16	-0.02	-0.08	-0.26
BM39	0.36	0.70	0.63	0.05	0.32	0.85	0.71	0.56	0.67	0.41	-0.02	0.68	0.61	0.60	0.69	0.43	0.66	0.61	0.55	0.27
BM40	0.01	0.37	0.17	0.67	0.23	0.74	0.13	0.71	0.59	0.17	0.44	0.05	0.41	0.60	0.00	0.55	0.38	0.72	0.84	0.50
BM41	0.49	0.73	0.71	0.60	0.58	0.65	-0.03	0.27	0.60	0.26	0.64	0.78	0.55	0.55	0.39	0.23	0.52	0.37	0.62	-0.17
BM42	0.80	0.62	0.79	0.72	0.50	0.80	0.12	0.00	0.26	0.37	0.66	0.33	0.22	0.65	0.18	0.00	0.39	0.42	0.42	-0.08
BM43	-0.39	-0.03	0.71	0.63	0.08	0.85	0.46	0.09	0.94	0.25	0.78	0.08	0.34	0.51	0.18	0.22	0.46	0.01	0.68	0.28
BM44	0.71	0.73	0.75	0.36	0.37	0.78	0.76	0.31	0.66	0.33	-0.02	0.02	0.39	0.65	-0.03	0.44	0.27	0.60	0.65	0.26
BM45	0.25	0.59	0.58	0.63	0.53	0.71	-0.15	0.24	0.17	0.51	0.74	0.28	0.07	0.59	0.00	0.51	0.02	0.11	0.50	0.57
BM46	0.09	0.00	0.58	0.55	0.29	0.72	-0.03	0.21	0.61	0.07	0.18	-0.03	-0.02	0.41	0.05	-0.13	0.14	0.73	0.42	-0.27
BM47	-0.18	0.62	-0.08	0.14	0.69	0.63	0.17	-0.11	0.47	0.66	0.47	0.41	0.10	0.28	0.02	0.16	0.41	0.16	0.23	-0.06
BM48	0.96	0.19	0.79	0.49	0.04	0.83	0.57	0.28	0.17	0.03	0.57	0.69	0.31	0.38	0.32	0.08	-0.26	-0.04	0.54	-0.17
BM49	0.37	-0.04	0.81	0.25	-0.16	0.85	0.65	0.03	0.58	-0.14	0.14	0.58	0.48	0.33	0.25	0.28	0.42	0.10	0.25	-0.01
BM50	0.02	0.59	0.38	0.59	0.70	0.82	0.66	0.22	0.65	0.56	0.14	0.63	0.26	0.74	-0.07	-0.09	0.70	0.11	0.29	-0.11
BM51	0.12	0.45	0.64	0.34	0.66	0.25	0.66	-0.07	0.26	0.23	0.32	0.39	-0.21	0.08	-0.04	0.25	0.32	0.69	-0.10	0.31
BM52	0.17	0.63	0.70	0.62	0.72	0.81	0.62	0.54	0.64	0.20	0.34	0.72	0.16	0.65	0.70	0.26	0.49	0.55	0.65	0.36
BM53	0.68	0.71	0.43	0.28	0.53	0.78	0.53	0.49	0.59	0.37	0.84	0.72	0.42	0.66	0.69	0.03	0.52	0.55	0.64	0.23
BM54	0.84	-0.01	0.39	0.42	0.18	0.76	0.04	0.40	0.42	-0.06	0.10	0.56	0.01	0.33	0.69	0.17	0.40	0.39	0.74	0.26
BM55	-0.09	0.73	-0.02	0.30	0.87	0.79	-0.04	0.74	0.54	0.56	0.78	0.66	-0.13	0.00	-0.10	-0.05	0.06	0.20	0.16	-0.06

Supplementary Table 21 continued

	Component ICs													*Noise ICs*						
	IC01	IC03	IC05	IC06	IC07	IC08	IC09	IC11	IC14	IC15	IC18	IC19	IC20	IC02	IC04	IC10	IC12	IC13	IC16	IC17
BM56	0.41	0.71	-0.04	0.11	0.51	0.79	-0.05	0.66	0.80	0.71	0.56	0.67	0.69	0.60	0.68	0.24	0.68	0.66	0.41	0.48
BM57	-0.08	0.09	0.04	0.21	0.33	0.80	0.38	0.60	0.05	0.01	0.02	0.69	0.21	0.49	0.20	0.19	-0.13	0.44	0.85	0.04
BM58	0.09	-0.15	0.16	0.21	0.24	0.61	0.46	0.67	0.10	0.17	0.89	0.04	0.08	0.65	0.69	-0.12	0.37	0.11	0.85	0.58
BM59	0.12	0.10	0.51	0.52	0.56	0.96	0.55	0.31	0.57	0.52	0.64	0.79	0.19	0.74	-0.12	0.31	0.61	0.56	0.34	-0.15
BM60	0.81	0.75	0.12	-0.02	0.73	0.30	-0.26	-0.12	0.28	0.62	0.54	0.24	0.01	0.62	0.71	0.17	0.53	0.46	0.22	0.72
BM61	0.74	0.71	0.72	0.82	0.55	0.68	0.12	0.43	0.27	-0.17	-0.12	0.66	-0.23	0.59	0.75	0.71	0.23	0.18	0.57	0.31
BM62	0.55	0.67	0.50	0.63	0.64	0.89	0.36	0.38	-0.05	0.17	0.97	0.29	-0.05	0.44	-0.02	0.12	0.16	0.73	0.17	0.15
BM63	-0.07	0.57	0.13	0.26	0.54	0.76	0.18	-0.16	0.13	0.50	0.19	0.02	0.11	0.92	0.16	0.25	0.11	0.02	0.08	0.67
BM64	0.27	0.37	0.50	0.70	0.15	0.67	0.78	0.66	0.28	0.27	0.80	-0.10	0.20	0.59	-0.33	0.09	0.47	-0.04	0.69	-0.14
BM65	-0.05	0.09	0.04	0.50	0.04	0.58	0.09	0.58	0.13	0.55	0.41	0.68	0.38	0.38	0.11	0.30	0.27	0.68	0.09	-0.01
BM66	0.70	-0.09	0.59	0.17	0.24	0.78	0.57	0.32	0.54	0.47	0.76	0.62	0.37	0.72	0.43	0.38	0.58	0.24	0.86	0.38
BM67	0.17	0.88	0.17	0.47	0.55	0.07	-0.11	0.56	0.40	0.31	0.44	0.45	0.44	0.69	0.18	-0.03	0.44	0.54	0.54	0.40
BM68	0.54	0.75	0.67	0.75	0.63	0.76	0.65	0.47	0.27	0.13	0.72	0.65	0.53	0.67	0.00	-0.12	0.63	0.69	0.67	0.41
BM69	0.67	0.64	0.81	0.84	0.55	0.80	0.64	0.48	0.79	0.51	0.70	0.77	0.72	0.80	0.57	0.25	0.84	0.70	0.59	0.62
BM70	0.71	0.75	0.63	0.65	0.56	0.64	0.67	0.77	0.76	0.15	0.59	0.81	0.54	0.87	0.68	0.27	0.71	0.57	0.68	0.41

Supplementary Table 22. Between-session ICC values for Normalised Mean ICN$_i$ Activation (MA$_{N,i}$) values for the NYU-TRT group ICA data atlased using the BRAINMAP70 atlas. *See Supplementary Table 1 for the description of atlas ICNs (BM01-BM70). For visual representation see Supplementary Figure 7 panel F.*

	Component ICs													*Noise ICs*					
	IC01	IC03	IC05	IC06	IC07	IC08	IC09	IC11	IC14	IC15	IC18	IC19	IC20	IC02	IC04	IC10	IC12	IC13	IC16	IC17
BM01	0.43	0.71	0.01	0.17	0.35	0.24	0.20	0.80	0.44	-0.13	0.29	-0.17	0.26	0.52	-0.10	0.19	0.37	0.22	0.05	-0.18
BM02	0.58	0.08	0.54	0.31	0.18	0.03	0.08	0.11	-0.02	0.17	0.15	0.35	-0.02	0.64	0.30	0.01	-0.06	0.14	0.40	0.26
BM03	0.32	-0.16	0.13	0.27	0.19	0.24	-0.04	-0.03	0.51	0.08	0.19	0.42	-0.02	0.41	-0.23	0.19	0.55	0.30	0.10	0.02
BM04	0.31	0.13	0.05	0.34	-0.07	-0.10	0.28	-0.16	0.08	-0.32	0.24	0.25	0.15	0.56	-0.07	0.11	0.33	-0.13	0.26	-0.30
BM05	0.58	-0.22	0.05	-0.07	0.11	0.31	0.17	-0.14	0.12	0.11	0.18	-0.16	0.16	0.33	-0.19	-0.29	0.29	-0.06	0.18	0.01
BM06	0.51	0.00	0.46	0.24	0.23	0.40	0.72	0.07	0.42	0.22	0.14	0.43	-0.06	0.14	-0.20	0.26	0.05	-0.04	0.12	0.12
BM07	0.25	0.45	0.44	0.56	0.03	0.35	0.62	0.44	0.20	-0.10	0.20	0.34	0.05	0.23	0.01	0.38	0.34	0.44	0.15	0.13
BM08	0.29	0.40	0.58	-0.01	0.16	0.43	0.74	0.30	0.03	-0.24	0.13	0.04	0.35	-0.03	0.07	-0.09	0.40	0.00	0.16	-0.10
BM09	0.27	0.44	-0.08	0.30	-0.19	-0.13	0.66	0.25	-0.04	0.07	0.03	0.55	0.10	0.28	0.61	-0.07	0.01	-0.01	0.05	-0.23
BM10	-0.18	0.00	0.10	0.10	0.19	0.20	0.20	0.37	0.21	0.16	0.27	0.28	0.13	0.24	0.06	0.27	0.45	0.13	0.06	-0.27
BM11	0.00	0.21	0.26	-0.20	0.12	0.18	0.34	0.29	-0.17	-0.12	-0.09	0.11	0.24	0.01	-0.07	0.18	0.42	0.06	0.06	-0.24
BM12	0.43	0.25	0.29	0.50	0.34	0.37	0.39	0.36	0.00	-0.18	0.43	0.36	0.15	0.52	0.42	0.37	0.26	-0.16	-0.12	0.22
BM13	0.09	-0.03	0.51	0.33	0.10	0.23	0.38	0.52	0.15	0.27	-0.01	0.23	-0.01	-0.13	0.10	-0.10	0.35	0.05	0.01	-0.07
BM14	-0.07	0.35	0.05	0.44	0.05	0.09	0.12	0.04	0.51	-0.20	0.21	0.31	0.45	0.36	-0.17	-0.15	0.33	0.26	0.03	0.14
BM15	-0.14	0.21	0.48	0.10	0.45	0.17	0.24	0.15	-0.06	0.15	-0.05	-0.18	-0.07	0.38	0.14	0.27	0.12	0.42	-0.05	0.07
BM16	-0.08	-0.13	0.48	0.12	0.10	0.05	0.25	0.01	-0.14	-0.11	0.16	0.29	0.16	0.08	0.13	0.10	0.46	0.02	0.25	-0.35
BM17	-0.05	0.12	-0.13	0.38	0.49	0.34	0.48	0.51	0.28	0.21	0.53	0.31	0.47	0.39	-0.09	0.05	-0.04	0.13	0.03	-0.10
BM18	-0.12	0.31	-0.24	-0.24	0.06	0.31	-0.23	0.34	0.42	0.13	0.04	0.15	0.22	-0.06	-0.27	0.17	0.28	0.59	0.09	0.48
BM19	-0.09	0.15	0.33	0.17	0.07	0.43	0.30	0.18	0.31	-0.25	0.21	0.18	0.34	0.19	0.38	0.17	-0.16	0.14	-0.14	0.03
BM20	-0.04	0.33	0.35	0.15	0.03	0.15	0.47	0.09	0.36	-0.04	-0.15	0.19	0.45	0.06	0.04	0.05	0.34	0.08	-0.01	0.01
BM21	0.24	0.51	0.42	0.12	0.01	0.12	0.03	0.05	0.31	0.02	-0.12	0.15	0.31	0.27	-0.10	0.33	0.37	0.12	0.05	0.07
BM22	-0.21	0.19	0.44	0.10	0.23	0.55	0.12	0.22	0.43	-0.03	-0.14	-0.05	-0.01	0.19	0.14	0.25	-0.02	-0.04	0.17	-0.43
BM23	0.17	0.66	-0.14	-0.01	0.17	0.20	0.31	0.17	0.26	-0.45	0.54	0.03	0.28	0.46	0.30	-0.13	0.30	0.04	0.50	0.22
BM24	0.47	0.46	0.24	0.04	0.15	0.07	0.23	0.02	-0.01	0.55	-0.12	0.40	0.22	0.27	0.01	-0.03	0.14	-0.13	0.44	0.09
BM25	0.15	0.21	0.56	-0.01	0.32	0.11	0.10	0.33	0.29	-0.16	-0.03	0.24	0.11	-0.16	0.23	0.08	0.17	0.36	0.08	0.36
BM26	-0.03	0.11	0.20	-0.13	0.05	0.24	0.23	-0.41	0.14	-0.18	0.09	-0.31	-0.09	0.15	0.37	-0.20	0.04	0.18	0.07	-0.11

Supplementary Table 22 continued

	Component ICs													*Noise ICs*					
	IC01	IC03	IC05	IC06	IC07	IC08	IC09	IC11	IC14	IC15	IC18	IC19	IC20	IC02	IC04	IC10	IC12	IC13	IC16	IC17
BM27	0.42	0.23	0.38	-0.10	0.73	0.06	0.33	0.30	0.21	0.29	0.10	0.15	0.34	0.18	0.06	-0.22	0.00	0.18	0.16	0.10
BM28	0.20	0.47	0.42	-0.09	0.30	0.43	0.60	0.31	0.30	-0.01	-0.06	0.29	0.09	-0.16	-0.09	0.04	0.50	0.40	0.00	-0.21
BM29	0.25	0.45	0.52	0.40	0.24	0.12	0.56	0.22	0.15	-0.40	0.35	0.02	0.14	0.34	0.21	0.05	-0.01	-0.06	0.06	-0.12
BM30	0.00	0.48	0.34	-0.09	0.17	-0.02	0.43	0.24	0.17	-0.15	-0.09	0.24	0.42	0.10	-0.14	0.14	0.64	0.00	0.12	0.01
BM31	-0.16	0.10	0.14	-0.02	0.16	0.20	0.28	0.19	0.21	0.10	0.17	0.28	0.29	0.18	0.18	0.35	0.34	0.08	0.37	0.25
BM32	0.41	0.57	-0.17	0.29	0.04	0.34	0.46	-0.07	0.61	0.05	0.14	0.44	-0.05	0.51	0.20	0.11	0.46	0.25	0.43	0.17
BM33	0.27	0.08	-0.01	0.44	0.26	0.49	0.52	0.00	0.26	0.22	0.20	0.11	0.42	0.30	0.05	0.55	0.62	0.29	0.08	0.06
BM34	0.44	0.33	0.04	0.50	0.25	-0.03	0.50	0.30	0.17	-0.23	0.27	0.09	0.05	-0.05	-0.11	-0.05	0.58	-0.12	0.32	0.01
BM35	0.39	0.34	0.18	0.27	0.45	-0.15	-0.03	0.22	0.01	-0.21	-0.01	0.22	0.10	0.64	-0.10	0.24	0.40	0.22	0.05	-0.12
BM36	0.04	-0.03	0.23	0.24	0.14	0.20	-0.08	0.30	0.23	0.19	-0.11	0.05	0.14	0.31	0.08	0.37	0.13	-0.07	0.13	-0.04
BM37	0.00	0.52	0.38	0.25	-0.01	0.11	0.43	0.48	0.03	0.06	-0.05	0.33	0.13	0.05	0.08	0.37	0.07	0.09	0.21	0.22
BM38	0.24	0.35	0.31	-0.06	0.05	0.57	0.57	0.54	0.33	-0.11	-0.11	0.20	0.13	0.06	0.41	0.16	-0.03	0.20	0.06	0.05
BM39	-0.01	0.25	0.36	-0.01	0.32	0.16	0.46	0.00	0.29	0.09	-0.21	0.34	0.54	0.33	-0.33	0.08	0.34	0.28	0.23	-0.08
BM40	0.04	0.08	0.65	0.50	0.32	0.06	0.16	0.22	0.02	0.13	0.18	0.35	0.25	0.49	-0.17	-0.23	0.39	0.23	0.00	-0.11
BM41	0.23	0.37	0.52	-0.06	0.22	0.31	-0.10	0.02	0.40	0.04	0.40	0.31	0.31	0.48	0.09	0.13	0.27	0.16	0.41	0.18
BM42	0.31	0.54	0.63	0.33	0.14	0.29	0.01	0.08	-0.05	-0.18	0.02	0.37	0.34	0.09	0.14	0.04	0.18	0.16	-0.10	0.14
BM43	0.11	0.62	0.45	-0.05	-0.07	0.07	0.58	0.25	0.16	-0.15	0.18	-0.02	0.05	0.49	0.25	0.20	0.42	0.22	0.04	0.24
BM44	0.22	0.24	0.49	0.36	-0.03	0.54	0.50	0.63	0.27	-0.03	-0.13	0.35	0.02	0.18	0.15	0.16	0.15	0.13	0.23	-0.17
BM45	0.31	0.10	0.27	0.04	0.04	0.59	0.04	0.12	-0.05	-0.11	0.00	0.38	0.13	-0.08	0.03	0.21	0.30	-0.01	0.30	-0.13
BM46	0.29	0.09	0.30	0.30	0.27	0.38	0.57	0.58	0.35	-0.20	0.24	0.07	0.20	0.36	0.28	0.03	0.02	0.51	0.07	-0.17
BM47	0.05	0.18	-0.04	-0.10	0.34	0.48	0.18	-0.02	-0.02	-0.07	0.81	0.07	0.20	-0.15	0.35	0.11	0.25	0.00	0.19	0.02
BM48	0.59	0.31	0.50	0.55	0.23	0.24	0.47	0.14	-0.09	-0.21	-0.12	0.09	0.25	-0.16	0.17	0.23	0.08	0.39	0.15	0.14
BM49	0.21	0.03	0.50	-0.08	0.02	-0.05	0.50	0.03	-0.17	-0.24	-0.02	0.21	-0.17	0.33	0.22	0.02	0.23	-0.05	-0.25	0.02
BM50	0.09	0.06	0.27	0.27	0.21	0.51	0.50	0.02	0.33	-0.08	0.56	-0.01	0.35	0.28	0.55	0.05	0.43	0.29	0.15	0.23
BM51	-0.10	0.10	0.46	0.44	0.26	0.50	0.56	-0.17	-0.26	0.39	0.25	-0.11	-0.21	0.43	0.47	0.01	0.17	0.14	-0.01	0.02
BM52	0.04	0.24	0.57	0.16	0.36	0.36	0.00	0.57	0.46	-0.05	0.41	0.22	0.17	0.51	-0.16	0.00	0.17	-0.06	-0.03	-0.01

Supplementary Table 22 continued

	Component ICs													*Noise ICs*						
	IC01	IC03	IC05	IC06	IC07	IC08	IC09	IC11	IC14	IC15	IC18	IC19	IC20	IC02	IC04	IC10	IC12	IC13	IC16	IC17
BM53	0.25	0.12	0.19	0.08	0.12	0.09	0.45	0.17	0.11	-0.12	-0.05	0.27	0.41	0.16	-0.12	0.60	0.25	0.13	-0.11	0.09
BM54	-0.03	-0.14	0.33	-0.10	0.08	0.40	0.07	-0.03	0.21	0.12	-0.18	-0.14	0.10	0.05	0.13	0.17	0.58	0.13	0.53	0.03
BM55	-0.41	-0.24	-0.08	-0.19	0.06	0.23	0.07	0.06	0.28	-0.11	0.07	0.30	-0.15	0.19	0.32	0.04	0.16	0.45	0.21	0.01
BM56	-0.21	0.21	0.15	0.29	0.11	0.10	-0.26	-0.04	0.18	0.12	-0.01	0.17	0.41	0.05	0.22	-0.10	0.25	0.64	-0.12	0.44
BM57	0.24	0.24	0.02	0.21	0.07	0.29	-0.04	0.24	0.21	0.15	0.17	0.12	0.17	0.15	0.04	0.36	0.08	0.38	0.31	-0.07
BM58	0.47	0.21	0.12	0.12	0.12	0.37	0.38	0.18	0.49	0.13	0.24	0.24	0.34	0.03	0.24	0.48	0.63	0.15	0.32	0.03
BM59	0.00	-0.05	0.51	0.16	0.15	0.24	0.41	0.01	0.44	0.03	0.18	0.08	0.11	0.66	0.02	0.21	0.34	0.48	0.07	0.37
BM60	0.31	0.50	-0.23	-0.01	0.23	0.07	-0.09	0.34	0.10	0.32	0.18	-0.04	-0.05	0.51	0.54	-0.14	0.26	0.27	0.09	0.12
BM61	0.54	0.56	0.35	-0.04	0.18	0.02	-0.03	0.14	0.12	0.02	0.17	0.45	0.10	0.55	-0.01	-0.20	0.21	0.00	0.24	0.05
BM62	0.18	0.42	0.30	0.36	0.46	-0.10	-0.12	0.05	0.05	0.19	0.45	0.15	-0.20	0.21	0.28	0.05	0.08	0.36	0.10	0.05
BM63	-0.13	-0.06	-0.13	-0.05	0.05	0.22	0.36	0.06	0.16	0.17	0.06	0.11	0.14	0.22	0.19	0.06	0.10	-0.10	-0.04	0.07
BM64	-0.26	0.27	0.43	-0.08	0.13	-0.23	0.62	0.26	0.09	0.01	-0.08	0.34	-0.07	0.42	-0.39	0.25	-0.25	0.18	0.16	-0.08
BM65	-0.17	0.06	0.14	0.48	0.18	-0.14	0.31	0.01	0.23	0.35	0.11	0.09	0.13	-0.28	-0.16	0.16	-0.08	0.47	0.32	0.04
BM66	0.26	-0.01	0.11	0.17	0.49	-0.17	0.18	0.03	0.20	0.63	0.59	0.32	0.18	0.59	-0.29	0.00	0.18	0.42	0.56	-0.13
BM67	0.44	0.28	-0.03	0.31	0.10	0.14	0.29	0.24	0.37	0.03	0.20	0.28	0.12	0.25	0.27	0.21	0.43	0.15	0.12	-0.19
BM68	-0.11	0.64	0.43	-0.05	-0.36	-0.05	0.45	0.20	-0.14	-0.12	-0.11	0.10	0.15	-0.19	0.38	0.50	0.14	0.10	0.02	-0.10
BM69	0.28	0.21	0.56	0.31	0.24	0.52	0.45	0.25	-0.03	-0.08	0.03	0.25	0.42	0.40	0.66	0.03	0.55	0.38	0.05	0.02
BM70	0.05	0.05	0.09	0.09	-0.12	-0.14	0.34	0.24	0.22	0.00	-0.21	0.39	0.36	0.68	0.00	0.03	0.28	0.16	0.18	0.39

Supplementary Table 23. Within-session ICC values for Normalised Relative ICN$_i$ Activation (RA$_{N,i}$) values for the NYU-TRT group ICA data atlased using the SMITH10 atlas. *ICN01 visual (medial), ICN02 visual (occipital pole), ICN03 visual (lateral), ICN04 DMN, ICN05 cerebellum, ICN06 sensori-motor, ICN07 auditory, ICN08 executive control, ICN09 fronto-parietal (perception, somesthesis, pain), ICN10 fronto-parietal (cognition-language). For a visual representation see Figure 7 panel C.*

	Component ICs													*Noise ICs*					
	IC01	IC03	IC05	IC06	IC07	IC08	IC09	IC11	IC14	IC15	IC18	IC19	IC20	IC02	IC04	IC10	IC12	IC13	IC16	IC17
ICN01	0.32	0.64	0.08	0.64	0.67	0.73	0.24	0.53	0.54	-0.07	0.24	0.20	0.14	0.53	0.57	0.16	0.05	0.38	0.28	-0.02
ICN02	0.25	-0.01	0.15	0.39	0.18	0.46	-0.09	0.39	0.72	0.45	0.22	0.63	0.54	0.65	-0.48	0.39	0.03	0.17	0.04	0.34
ICN03	0.60	0.64	0.07	0.41	0.26	0.27	0.25	0.76	0.32	-0.02	0.46	0.10	-0.03	0.61	0.31	0.43	0.21	0.74	-0.07	-0.11
ICN04	0.51	0.82	0.61	0.41	0.14	0.55	0.59	0.14	0.07	0.44	-0.06	0.19	0.39	0.37	0.33	0.59	-0.01	0.11	0.26	0.09
ICN05	0.23	0.26	0.12	0.02	0.15	0.19	0.04	-0.01	0.47	0.14	0.17	-0.07	0.12	0.50	0.37	-0.03	0.23	0.39	0.19	0.21
ICN06	0.13	0.31	0.59	0.39	0.45	0.38	0.37	-0.02	0.18	-0.27	-0.04	0.27	0.05	0.60	0.36	0.16	0.60	0.00	0.15	0.24
ICN07	0.35	0.58	0.55	0.50	0.65	0.64	-0.02	0.40	0.42	0.09	0.41	0.25	0.20	0.36	0.37	0.50	0.49	0.25	0.13	0.46
ICN08	0.20	0.06	0.64	0.10	0.24	0.50	0.25	-0.05	0.19	0.17	0.58	0.44	0.14	0.33	0.40	0.52	0.09	0.39	0.51	0.07
ICN09	0.21	0.56	0.42	0.37	0.37	0.73	0.42	0.41	0.38	0.31	0.54	-0.10	0.23	0.53	0.17	0.35	0.20	0.05	0.38	-0.46
ICN10	0.43	0.58	0.65	0.45	0.30	0.64	-0.17	0.29	0.38	0.30	0.39	0.22	-0.25	0.47	0.23	0.55	0.01	-0.26	0.11	0.23

Supplementary Table 24. Between-session ICC values for Normalised Relative ICN$_i$ Activation (RA$_{N,i}$) values for the NYU-TRT group ICA data atlased using the SMITH10 atlas. *ICN01 visual (medial), ICN02 visual (occipital pole), ICN03 visual (lateral), ICN04 DMN, ICN05 cerebellum, ICN06 sensori-motor, ICN07 auditory, ICN08 executive control, ICN09 fronto-parietal (perception, somesthesis, pain), ICN10 fronto-parietal (cognition-language). For a visual representation see Figure 7 panel G.*

	Component ICs													*Noise ICs*						
	IC01	IC03	IC05	IC06	IC07	IC08	IC09	IC11	IC14	IC15	IC18	IC19	IC20	IC02	IC04	IC10	IC12	IC13	IC16	IC17
ICN01	0.49	0.64	-0.10	0.18	0.12	0.45	0.31	0.50	0.08	0.03	0.26	0.47	-0.10	0.33	0.20	0.19	0.10	0.60	-0.06	0.18
ICN02	0.59	-0.14	-0.09	0.16	0.08	0.26	0.11	0.06	0.06	-0.01	0.15	0.17	0.24	0.64	0.05	0.32	0.20	-0.06	-0.21	0.32
ICN03	0.37	0.46	0.04	0.04	0.43	0.38	0.59	0.09	0.01	0.10	0.07	0.35	0.19	0.52	0.40	0.10	0.15	0.50	-0.05	-0.14
ICN04	0.34	0.64	0.40	0.42	0.45	0.60	0.21	0.48	0.22	0.29	-0.05	0.36	0.26	0.52	0.30	0.09	-0.20	-0.03	0.16	0.15
ICN05	0.53	0.18	-0.05	-0.03	-0.18	-0.28	-0.04	0.18	0.09	0.10	0.24	0.20	0.08	0.45	0.08	0.08	0.24	0.49	0.13	-0.03
ICN06	0.31	0.27	0.15	0.58	0.47	0.11	0.46	0.34	0.17	0.43	0.43	0.24	-0.17	0.65	0.22	0.21	0.14	0.45	0.25	0.09
ICN07	0.27	0.35	0.64	0.54	0.57	0.08	0.23	0.48	-0.03	-0.02	0.49	0.57	0.41	0.37	0.43	0.28	0.44	0.35	0.29	0.48
ICN08	0.24	0.14	0.57	0.15	0.40	0.59	0.06	-0.12	0.10	0.17	0.18	0.14	0.42	0.26	0.19	0.53	-0.07	0.15	0.19	0.22
ICN09	0.19	0.10	0.60	0.31	0.26	0.60	0.37	0.63	0.30	0.12	0.54	0.02	-0.10	0.58	0.28	0.19	-0.06	0.51	0.31	0.21
ICN10	0.39	0.42	0.59	0.24	0.32	0.35	0.19	0.47	0.01	0.13	0.28	0.43	-0.18	0.22	0.38	0.38	0.25	0.12	-0.04	0.09

Supplementary Table 25. Within-session ICC values for Normalised Relative ICN$_i$ Activation (RA$_{N,i}$) values for the NYU-TRT group ICA data atlased using the BRAINMAP20 atlas. *BM1: limbic and medial-temporal areas, BM2: subgenual ACC and OFC, BM3: bilateral BG and thalamus, BM4: bilateral anterior insula/frontal opercula and the anterior aspect of the body of the cingulate gyrus, BM5: midbrain, BM6: superior and middle frontal gyri, BM7: middle frontal gyri and superior parietal lobules, BM8: ventral precentral gyri, central sulci, postcentral gyri, superior and inferior cerebellum, BM9: superior parietal lobule, BM10: middle and inferior temporal gyri, BM11: lateral posterior occipital cortex, BM12: medial posterior occipital cortex, BM13: medial prefrontal and posterior cingulate/precuneus areas, DMN, BM14: cerebellum, BM15: right-lateralized fronto-parietal regions, BM16: transverse temporal gyri, BM17: dorsal precentral gyri, central sulci, postcentral gyri, superior and inferior cerebellum, BM18: left-lateralized fronto-parietal regions, BM19-20: artefactual. For a visual representation see Figure 7 panel K.*

	Component ICs													*Noise ICs*						
	IC01	IC03	IC05	IC06	IC07	IC08	IC09	IC11	IC14	IC15	IC18	IC19	IC20	IC02	IC04	IC10	IC12	IC13	IC16	IC17
BM01	0.01	0.00	0.56	0.31	0.37	0.25	0.52	0.10	0.18	-0.02	-0.15	0.28	-0.22	0.31	0.16	0.15	0.03	0.76	0.57	0.52
BM02	0.32	0.31	0.00	0.20	-0.21	0.23	0.09	0.18	0.26	0.10	0.22	0.02	0.12	0.10	-0.08	0.23	0.15	-0.08	0.56	0.03
BM03	0.28	0.45	0.40	0.09	0.56	0.35	0.65	0.30	0.55	0.15	0.46	0.32	-0.13	0.31	0.16	0.34	0.49	0.30	0.58	0.42
BM04	0.19	0.34	0.76	0.50	0.21	0.59	0.20	0.32	0.31	-0.08	0.39	0.23	0.41	0.19	0.23	0.32	-0.11	0.49	0.31	0.33
BM05	0.24	0.57	0.23	-0.04	0.25	0.22	0.09	0.14	0.55	0.02	0.01	0.00	-0.09	0.50	0.55	0.01	0.12	0.46	0.30	-0.13
BM06	0.60	0.57	0.26	0.42	0.53	0.39	0.79	0.36	0.62	0.02	-0.03	0.27	0.03	0.45	0.29	0.10	0.35	0.37	0.74	0.19
BM07	0.57	0.46	0.15	0.31	0.50	0.23	0.42	0.46	0.47	0.01	0.32	0.17	0.12	0.55	0.03	0.08	0.13	0.37	0.27	0.12
BM08	0.27	0.19	0.67	0.32	0.23	0.48	0.61	0.43	0.31	0.03	0.05	-0.10	0.36	0.81	0.26	0.17	0.41	0.24	0.12	0.34
BM09	0.51	-0.09	0.31	0.39	0.53	0.38	0.04	0.14	0.60	0.10	0.04	0.45	0.02	0.78	0.15	0.25	0.60	0.35	0.55	0.19
BM10	0.45	0.51	0.48	0.42	0.15	0.45	0.59	0.60	0.54	0.18	0.36	-0.13	-0.01	0.41	0.51	0.32	-0.02	0.83	0.34	-0.14
BM11	0.15	0.05	0.04	0.29	0.35	0.18	-0.13	0.39	0.47	0.24	0.34	0.10	0.25	0.69	-0.39	0.24	0.22	0.25	-0.27	0.21
BM12	0.24	0.77	0.21	0.47	0.67	0.63	0.31	0.34	0.45	-0.02	0.34	0.08	0.22	0.81	0.55	0.22	0.08	0.07	0.38	0.40
BM13	0.21	0.70	0.51	0.40	0.49	0.32	0.47	0.29	0.14	0.54	-0.04	0.30	0.35	0.66	0.15	0.46	-0.05	0.21	0.06	-0.07
BM14	0.66	0.31	0.27	0.24	0.35	0.30	0.39	0.18	0.14	0.00	0.22	-0.22	0.16	0.71	0.54	0.21	0.10	0.17	0.46	0.38
BM15	0.24	0.22	0.37	0.15	0.41	0.54	0.44	0.40	0.07	0.03	0.50	-0.06	0.20	0.47	0.35	-0.08	0.51	0.07	0.28	0.32
BM16	0.22	0.52	0.63	0.20	0.71	0.38	0.48	-0.06	-0.09	0.02	0.09	0.41	-0.04	0.53	-0.31	0.17	0.38	0.29	0.22	0.39
BM17	0.21	0.54	0.28	-0.26	0.34	0.73	0.02	-0.06	0.40	0.09	0.15	0.23	0.20	0.42	0.41	-0.10	0.47	-0.21	0.50	0.19
BM18	0.63	0.54	0.61	0.54	0.24	0.64	0.21	-0.04	0.15	-0.08	0.33	0.16	-0.16	0.39	0.39	-0.11	0.30	-0.23	0.53	0.21
BM19	-0.04	0.12	0.51	-0.06	-0.01	0.17	0.27	0.40	0.52	0.02	0.39	0.36	0.18	0.65	0.54	0.56	0.17	0.36	0.43	0.02
BM20	0.15	0.77	0.62	0.33	0.67	0.36	0.22	0.32	0.43	0.14	0.36	0.31	0.20	0.58	0.17	0.29	0.68	0.18	0.52	0.29

Supplementary Table 26. Between-session ICC values for Normalised Relative ICN_i Activation (RA_{Ni}) values for the NYU-TRT group ICA data atlased using the BRAINMAP20 atlas. *BM1: limbic and medial-temporal areas, BM2: subgenual ACC and OFC, BM3: bilateral BG and thalamus, BM4: bilateral anterior insula/frontal opercula and the anterior aspect of the body of the cingulate gyrus, BM5: midbrain, BM6: superior and middle frontal gyri, BM7: middle frontal gyri and superior parietal lobules, BM8: ventral precentral gyri, central sulci, postcentral gyri, superior and inferior cerebellum, BM9: superior parietal lobule, BM10: middle and inferior temporal gyri, BM11: lateral posterior occipital cortex, BM12: medial posterior occipital cortex, BM13: medial prefrontal and posterior cingulate/precuneus areas, DMN, BM14: cerebellum, BM15: right-lateralized fronto-parietal regions, BM16: transverse temporal gyri, BM17: dorsal precentral gyri, central sulci, postcentral gyri, superior and inferior cerebellum, BM18: left-lateralized fronto-parietal regions, BM19-20: artefactual. For a visual representation see Figure 7 panel O.*

	Component ICs													*Noise ICs*						
	IC01	IC03	IC05	IC06	IC07	IC08	IC09	IC11	IC14	IC15	IC18	IC19	IC20	IC02	IC04	IC10	IC12	IC13	IC16	IC17
BM01	0.56	0.26	0.65	-0.37	0.21	0.30	0.44	0.18	0.20	0.03	0.15	0.49	0.28	0.34	0.29	0.20	0.20	0.65	0.44	0.22
BM02	0.75	0.29	0.30	0.32	-0.10	0.14	-0.03	0.15	0.27	-0.10	0.01	0.09	0.15	0.18	0.13	-0.01	-0.14	0.08	0.22	0.08
BM03	0.26	0.36	0.02	0.51	0.49	0.68	0.60	0.37	0.33	0.13	0.33	0.28	0.03	-0.04	0.24	0.12	0.50	0.31	-0.11	0.75
BM04	0.16	0.05	0.77	0.33	0.59	0.45	-0.02	0.46	0.40	0.02	0.04	-0.01	0.46	0.63	0.10	0.45	0.28	0.33	-0.02	0.13
BM05	0.34	0.32	-0.01	0.21	0.23	-0.03	0.02	0.16	0.42	0.10	-0.01	0.17	0.32	0.23	0.58	0.04	0.40	0.52	-0.11	0.20
BM06	0.20	0.35	0.55	0.40	0.60	0.36	0.75	0.14	0.11	0.26	0.46	0.29	-0.10	0.30	0.20	0.11	0.11	0.65	0.40	0.07
BM07	0.65	0.25	0.28	0.52	0.44	0.28	0.69	0.53	0.28	-0.05	0.20	0.18	0.12	0.61	0.15	0.09	0.25	0.16	0.00	0.37
BM08	0.00	0.56	0.53	0.51	0.44	0.56	0.56	0.58	0.13	0.16	0.49	-0.18	0.14	0.62	0.43	0.06	0.27	-0.05	0.02	0.27
BM09	0.38	0.25	-0.06	0.57	0.36	-0.22	-0.14	0.35	0.40	0.19	0.14	0.06	0.39	0.50	0.15	0.05	0.02	0.16	0.26	-0.07
BM10	0.47	0.62	0.43	0.42	0.48	0.47	0.74	0.08	0.02	0.01	-0.07	0.23	0.04	0.38	0.29	0.29	0.09	0.09	0.10	0.02
BM11	0.46	-0.05	0.02	0.06	0.27	0.45	0.12	0.08	0.18	0.03	0.37	0.19	0.11	0.56	0.35	0.36	0.20	-0.14	0.06	0.34
BM12	0.54	0.76	-0.22	0.32	0.21	0.47	0.34	0.38	0.03	-0.07	0.07	0.47	-0.01	0.62	0.14	0.31	0.23	0.41	0.31	0.10
BM13	0.22	0.70	0.47	0.19	0.34	0.30	0.31	0.37	0.38	0.34	0.33	0.53	0.28	0.54	0.34	0.19	0.12	-0.15	0.34	0.31
BM14	0.76	-0.02	0.36	0.27	-0.04	0.10	0.41	-0.19	0.25	0.11	0.18	0.22	0.04	0.66	0.30	-0.01	0.22	0.32	0.23	0.06
BM15	0.36	0.19	0.56	0.13	0.21	0.55	0.31	0.54	-0.06	0.45	0.63	-0.14	0.05	0.41	0.24	0.24	0.19	0.51	-0.38	0.25
BM16	0.27	0.33	0.34	0.36	0.48	0.06	0.56	0.30	0.05	0.28	0.35	0.36	0.28	0.55	0.22	0.00	0.39	0.26	0.53	0.04
BM17	-0.06	-0.11	0.29	0.08	0.17	0.66	0.30	0.20	0.09	0.33	0.37	0.57	-0.09	0.29	0.36	0.27	0.53	0.31	0.24	0.13
BM18	0.50	0.02	0.63	0.34	0.04	0.10	0.29	0.03	0.06	0.10	0.25	0.11	0.19	0.03	0.12	-0.05	0.23	0.16	-0.26	-0.11
BM19	0.48	0.47	0.15	0.11	0.21	0.02	0.45	-0.08	0.55	0.16	0.34	0.34	-0.05	0.65	0.36	0.44	0.24	0.30	0.08	0.41
BM20	0.50	0.53	0.37	0.48	0.19	0.27	0.08	0.32	0.37	0.00	0.02	0.07	0.10	0.35	0.13	0.17	0.45	0.35	0.52	0.24

Supplementary Table 27. Within-session ICC values for Normalised Relative ICN$_i$ Activation (RA$_{N,i}$) values for the NYU-TRT group ICA data atlased using the BRAINMAP70 atlas. *See Supplementary Table 1 for the description of atlas ICNs (BM01-BM70). For visual representation see Supplementary Figure 7 panel C.*

	Component ICs													*Noise ICs*						
	IC01	IC03	IC05	IC06	IC07	IC08	IC09	IC11	IC14	IC15	IC18	IC19	IC20	IC02	IC04	IC10	IC12	IC13	IC16	IC17
BM01	0.05	0.62	0.13	0.45	0.30	0.52	0.65	0.29	0.83	-0.07	0.24	-0.05	0.31	0.78	-0.16	-0.03	0.40	-0.01	0.01	0.07
BM02	0.23	0.68	0.43	0.37	0.67	0.27	0.03	0.63	0.49	-0.02	0.32	0.17	0.26	0.90	0.41	0.13	0.18	-0.05	0.24	0.59
BM03	0.26	0.06	-0.07	0.31	0.24	0.18	0.05	0.38	0.60	0.39	0.17	0.03	0.36	0.70	-0.38	0.24	0.24	0.23	-0.26	0.19
BM04	0.53	0.11	0.02	0.33	0.42	0.40	0.36	0.75	0.05	-0.05	0.39	0.11	0.03	0.68	0.04	0.16	0.50	0.32	-0.21	0.00
BM05	0.77	0.25	0.22	0.30	0.20	0.21	0.15	0.08	0.27	0.44	0.42	0.12	-0.01	0.29	0.19	0.07	0.09	0.86	0.36	-0.06
BM06	0.29	-0.06	0.46	0.41	0.39	0.14	0.50	0.54	0.15	0.03	0.47	0.02	0.01	0.40	0.22	0.33	0.20	0.48	0.30	0.25
BM07	0.66	0.42	0.15	0.33	-0.14	0.02	0.65	0.45	0.51	0.19	0.20	0.15	-0.07	0.56	0.03	-0.03	0.24	0.08	0.27	0.29
BM08	0.33	0.41	0.70	0.47	-0.17	0.59	0.63	0.27	0.46	0.14	0.59	-0.12	0.49	-0.03	-0.03	0.35	0.29	-0.10	0.22	-0.17
BM09	0.75	0.67	0.10	0.56	0.07	0.17	0.64	0.01	-0.14	0.31	0.14	-0.06	0.42	-0.14	0.25	0.07	0.29	0.35	0.13	-0.22
BM10	0.36	-0.03	0.54	0.34	0.73	0.27	0.67	0.25	0.13	0.43	0.53	0.56	-0.13	0.29	0.17	0.24	0.21	0.15	0.55	-0.08
BM11	0.34	0.55	0.57	0.37	0.31	0.64	0.61	0.27	0.00	0.08	0.33	0.02	0.02	0.23	0.37	0.19	0.53	0.24	0.13	0.08
BM12	0.16	0.23	0.38	0.71	0.36	0.33	0.37	0.73	0.24	-0.25	0.43	0.02	0.36	0.77	0.17	-0.12	0.53	0.26	0.29	0.12
BM13	0.08	0.02	0.17	0.29	0.50	0.35	0.46	0.60	0.28	0.17	-0.10	0.01	0.11	0.24	0.26	0.02	0.20	0.28	0.34	-0.07
BM14	0.18	0.54	0.34	0.16	0.24	0.11	0.33	0.20	0.46	0.11	0.52	0.32	-0.03	0.25	0.24	0.18	0.22	0.14	0.36	0.31
BM15	0.35	-0.10	0.34	0.42	0.49	0.62	0.12	0.20	0.63	-0.01	0.10	0.33	-0.05	0.52	0.15	0.11	0.13	0.33	0.42	-0.17
BM16	0.71	0.68	0.55	0.37	0.55	0.03	0.49	0.13	0.07	0.43	0.51	0.21	0.31	0.36	-0.02	0.29	0.38	0.22	0.17	-0.11
BM17	0.22	0.32	0.44	0.22	0.53	0.64	0.18	0.57	0.13	-0.05	0.07	0.38	-0.15	0.80	0.26	0.17	0.03	-0.08	-0.05	0.63
BM18	-0.01	-0.10	0.25	0.01	0.14	0.21	-0.11	0.00	0.16	-0.02	0.06	0.08	-0.14	0.50	0.23	0.38	0.24	0.39	0.16	0.46
BM19	0.19	0.23	0.53	-0.02	0.31	0.23	0.44	-0.01	0.48	0.19	0.13	-0.10	-0.28	0.06	0.23	-0.10	0.26	0.12	0.37	-0.22
BM20	0.44	0.18	0.35	-0.14	0.08	0.60	0.02	0.03	-0.08	0.22	0.17	-0.03	0.28	0.64	0.07	0.49	0.32	0.24	0.24	-0.25
BM21	0.31	0.66	0.24	0.41	-0.16	-0.01	0.08	0.24	0.35	0.51	-0.04	-0.03	0.05	0.24	-0.08	0.38	-0.17	0.04	0.25	0.37
BM22	0.03	0.09	0.42	0.61	-0.06	0.35	0.32	0.28	0.23	0.26	0.26	0.02	0.29	0.16	0.41	0.43	0.05	0.62	0.38	-0.12
BM23	-0.03	0.60	0.25	0.15	0.44	0.56	0.57	0.34	0.26	0.29	-0.04	0.26	0.54	0.71	0.68	0.38	0.23	0.32	0.34	0.17
BM24	-0.01	0.63	0.38	0.36	0.49	0.42	0.34	0.24	0.48	0.26	-0.13	0.13	-0.06	0.37	0.58	0.10	0.19	0.53	0.53	-0.15
BM25	0.36	0.25	0.29	-0.18	0.10	0.42	0.51	0.22	0.35	-0.16	0.09	0.29	0.38	-0.12	0.65	0.28	0.01	0.48	0.33	0.20

Supplementary Table 27 continued

	Component ICs													*Noise ICs*					
	IC01	IC03	IC05	IC06	IC07	IC08	IC09	IC11	IC14	IC15	IC18	IC19	IC20	IC02	IC04	IC10	IC12	IC13	IC16	IC17
BM26	-0.03	0.26	0.03	0.07	-0.03	0.26	0.83	0.32	0.06	0.53	0.32	0.18	-0.01	0.13	0.36	0.52	-0.07	0.19	0.47	-0.21
BM27	-0.08	0.51	0.63	0.07	-0.15	0.44	0.50	0.02	0.28	0.21	0.51	0.12	0.58	0.48	0.43	0.10	-0.19	0.18	0.14	0.05
BM28	-0.19	0.61	0.15	0.06	0.32	0.53	0.13	0.36	0.20	0.40	0.03	-0.19	0.03	0.32	-0.04	0.17	0.38	-0.05	0.23	0.31
BM29	0.04	0.70	0.65	0.59	0.21	0.61	0.69	0.71	0.04	-0.02	0.51	0.67	0.80	0.00	0.10	0.59	-0.02	-0.30	0.44	0.16
BM30	0.26	0.53	0.19	0.40	0.71	0.23	0.54	-0.07	0.28	-0.23	0.11	0.11	-0.06	0.34	0.00	-0.08	0.56	0.00	0.39	-0.01
BM31	0.67	0.46	0.63	0.14	0.18	0.02	0.25	0.35	0.16	-0.06	0.31	0.20	0.19	0.59	0.03	0.68	0.16	0.21	0.61	0.27
BM32	0.54	0.58	-0.03	0.69	0.47	0.46	0.12	0.19	0.23	-0.12	0.33	0.13	-0.13	0.32	0.58	0.14	0.20	-0.03	0.43	0.13
BM33	0.08	0.24	0.23	0.54	0.01	0.65	0.70	0.02	0.19	0.01	-0.18	0.06	0.24	0.54	0.44	0.40	0.55	0.46	0.13	0.58
BM34	0.34	0.32	0.34	0.34	0.38	0.02	0.49	0.23	0.64	0.39	-0.32	0.30	0.07	0.53	-0.14	-0.20	0.19	0.37	0.05	0.24
BM35	0.56	0.39	0.08	-0.04	0.52	0.08	-0.13	0.17	0.42	-0.07	0.05	-0.06	0.21	0.59	0.37	0.02	0.39	0.43	0.47	0.14
BM36	0.53	0.13	0.80	0.18	0.44	0.37	0.10	0.20	0.24	0.18	0.02	0.31	-0.10	0.52	0.01	-0.10	0.22	-0.06	0.49	-0.06
BM37	0.30	0.22	0.84	-0.06	0.50	0.27	0.71	0.62	0.21	0.00	-0.06	-0.12	0.33	0.43	0.03	0.18	0.22	-0.04	0.01	0.05
BM38	0.60	0.71	0.70	0.66	0.49	0.79	0.90	0.47	0.24	0.29	0.30	-0.02	-0.16	0.65	-0.19	-0.17	-0.45	-0.08	0.32	-0.20
BM39	0.02	0.34	0.44	0.16	0.38	0.54	0.18	0.33	0.67	0.18	-0.04	0.51	0.46	0.60	-0.03	0.19	0.02	0.69	0.09	0.47
BM40	0.15	-0.24	0.32	0.45	0.47	0.45	0.25	0.16	0.63	0.32	0.00	0.51	0.22	0.75	0.01	0.15	0.47	0.51	0.70	0.13
BM41	0.06	0.45	0.48	0.38	0.56	0.58	0.64	0.31	0.17	0.16	0.04	0.35	-0.06	0.75	0.07	0.13	0.11	0.36	-0.08	0.34
BM42	0.50	0.42	0.61	0.74	0.20	0.49	0.24	0.66	0.55	-0.10	0.59	0.13	-0.15	0.08	0.35	0.02	-0.04	0.70	0.05	-0.05
BM43	-0.08	0.13	0.80	0.56	0.17	0.45	0.62	0.39	0.75	0.14	0.10	0.28	0.12	0.34	0.05	0.74	0.55	-0.12	0.50	-0.05
BM44	0.32	0.31	0.70	-0.24	0.63	0.43	0.31	0.10	-0.06	-0.08	-0.05	0.27	-0.07	0.43	-0.27	0.01	-0.06	0.21	0.26	0.37
BM45	0.16	0.35	0.43	0.40	0.70	0.36	0.33	0.39	0.21	0.29	0.24	0.16	-0.10	0.38	-0.17	0.60	0.48	0.39	0.18	-0.08
BM46	0.03	0.44	0.69	0.50	0.62	0.26	0.51	0.62	0.22	0.34	0.36	0.24	0.13	0.08	0.12	-0.18	0.18	0.78	-0.33	-0.15
BM47	-0.04	0.58	-0.01	0.00	0.59	0.65	0.06	0.08	0.59	0.20	0.07	0.26	0.05	-0.07	-0.06	-0.17	0.36	0.28	0.38	0.80
BM48	0.23	0.31	0.75	0.15	0.15	0.59	0.85	-0.06	-0.02	-0.09	-0.04	-0.06	0.15	0.02	0.34	-0.03	-0.13	-0.12	0.13	0.78
BM49	0.32	0.17	0.53	0.20	-0.04	0.44	0.13	0.57	0.14	0.03	0.22	0.08	0.16	0.52	0.33	0.00	-0.18	0.14	0.24	0.12
BM50	0.32	0.36	0.60	0.39	0.17	0.70	0.51	0.57	0.48	0.36	0.14	0.22	-0.17	0.41	0.50	0.11	0.35	-0.01	0.02	-0.18
BM51	0.67	0.57	0.77	0.71	0.60	0.68	0.72	0.40	0.25	-0.05	0.31	0.05	-0.09	0.48	0.09	-0.03	0.20	0.05	0.07	0.45

Supplementary Table 27 continued

	Component ICs													Noise ICs						
	IC01	IC03	IC05	IC06	IC07	IC08	IC09	IC11	IC14	IC15	IC18	IC19	IC20	IC02	IC04	IC10	IC12	IC13	IC16	IC17
BM52	0.13	0.45	0.84	0.36	0.54	0.79	0.60	0.46	0.64	-0.22	0.69	0.67	0.44	0.43	0.27	0.50	0.21	-0.09	0.21	0.30
BM53	0.35	0.37	0.49	0.55	0.01	0.56	0.67	0.06	0.31	0.06	0.65	0.29	0.31	0.16	0.19	-0.03	0.15	-0.12	0.03	0.16
BM54	0.33	0.31	0.51	0.10	0.43	0.42	0.42	0.17	0.62	0.07	0.67	0.40	-0.23	0.29	0.23	0.02	0.01	0.36	0.33	-0.01
BM55	0.19	0.19	0.20	0.06	0.71	0.53	-0.28	-0.05	0.40	-0.08	0.09	0.52	0.04	0.52	0.21	0.95	0.04	0.16	0.40	0.12
BM56	0.13	0.34	0.18	-0.03	0.24	0.48	-0.14	-0.08	0.19	0.41	0.26	0.31	0.21	0.74	0.74	-0.04	0.05	0.50	-0.02	0.05
BM57	0.05	0.15	0.11	0.21	0.18	0.69	0.14	0.45	0.62	-0.02	0.32	0.51	0.34	0.21	-0.03	0.06	0.01	0.10	-0.01	0.13
BM58	0.20	0.11	-0.06	0.08	0.30	0.51	0.79	0.30	0.31	0.18	0.37	0.20	0.30	0.29	0.12	-0.18	0.52	0.04	0.44	0.20
BM59	-0.04	0.15	0.35	-0.02	0.33	0.15	0.30	0.07	0.14	0.05	-0.03	-0.13	0.33	0.72	0.02	0.46	-0.03	0.04	0.15	0.12
BM60	0.22	0.32	-0.03	0.33	0.28	0.64	0.01	0.12	0.41	0.31	0.61	0.13	0.03	0.62	0.31	0.23	0.19	0.27	0.63	0.30
BM61	0.41	0.74	0.73	0.53	-0.14	0.61	0.31	0.46	0.35	0.13	-0.01	0.28	-0.11	0.62	0.48	0.12	0.04	0.05	0.30	0.22
BM62	0.17	0.81	0.71	0.12	0.70	0.51	0.57	0.44	0.53	0.47	0.12	-0.13	-0.07	0.62	0.00	0.08	0.24	0.22	0.26	0.29
BM63	0.35	0.50	0.34	-0.04	0.20	0.75	0.04	-0.15	0.14	0.02	0.12	0.30	-0.03	0.21	0.01	-0.26	0.35	-0.06	0.42	0.18
BM64	-0.01	0.65	0.55	0.23	0.04	0.58	0.74	0.27	0.47	0.49	0.04	0.51	0.22	0.19	0.15	0.10	0.11	-0.25	0.58	0.33
BM65	0.22	0.28	-0.01	-0.02	0.10	0.26	0.06	-0.05	-0.01	-0.07	0.20	-0.09	-0.03	0.37	0.21	0.04	0.01	0.62	0.19	0.30
BM66	0.58	0.03	0.43	0.21	0.29	0.24	0.26	0.08	0.33	0.14	0.29	-0.24	-0.08	0.66	0.25	-0.06	0.42	0.03	0.70	0.19
BM67	-0.07	0.41	0.07	0.03	0.05	-0.14	0.23	0.11	-0.01	0.15	0.03	0.06	0.22	0.62	0.50	-0.02	0.53	0.47	0.25	0.50
BM68	0.11	0.64	0.78	-0.06	0.31	0.59	0.75	0.47	-0.02	0.61	0.46	0.35	-0.02	0.42	0.15	0.34	0.24	0.15	0.49	0.24
BM69	0.40	0.66	0.63	0.26	0.62	0.26	0.54	0.16	0.45	0.19	0.58	-0.02	0.43	0.56	0.22	0.33	0.66	0.33	0.51	0.09
BM70	-0.07	0.07	0.47	0.13	-0.09	0.06	0.25	0.52	0.49	-0.22	0.29	0.10	-0.02	0.68	0.73	0.46	0.26	0.33	0.36	0.17

Supplementary Table 28. Between-session ICC values for Normalised Relative ICN$_i$ Activation (RA$_{Ni}$) values for the NYU-TRT group ICA data atlased using the BRAINMAP70 atlas. *See Supplementary Table 1 for the description of atlas ICNs (BM01-BM70). For visual representation see Supplementary Figure 7 panel C.*

	Component ICs													*Noise ICs*						
	IC01	IC03	IC05	IC06	IC07	IC08	IC09	IC11	IC14	IC15	IC18	IC19	IC20	IC02	IC04	IC10	IC12	IC13	IC16	IC17
BM01	0.25	0.57	-0.03	0.10	0.00	0.29	-0.05	0.65	0.42	0.28	0.38	0.12	0.21	0.68	0.13	0.20	0.06	0.38	0.11	-0.04
BM02	0.49	0.57	0.08	0.01	0.19	0.38	0.38	0.01	0.08	0.04	0.20	0.55	-0.26	0.87	0.28	0.12	0.29	0.30	0.23	0.38
BM03	0.55	-0.06	0.01	0.30	0.32	0.42	0.15	0.03	0.14	0.00	0.30	0.17	-0.08	0.54	0.38	0.29	0.16	-0.02	-0.14	0.38
BM04	0.66	-0.06	-0.08	0.34	0.41	-0.15	0.08	-0.23	0.10	0.17	0.30	0.24	-0.05	0.54	0.27	0.14	-0.03	0.33	0.15	-0.01
BM05	0.62	0.29	0.07	0.28	0.24	0.27	0.21	-0.02	0.25	0.29	0.60	0.56	0.01	0.23	-0.06	-0.04	0.18	-0.01	0.32	0.17
BM06	0.42	0.11	0.65	0.45	0.45	0.33	0.68	0.60	0.21	0.37	0.41	0.47	0.30	0.27	0.03	0.26	-0.09	0.20	0.20	0.02
BM07	0.72	0.32	0.43	0.42	0.41	0.32	0.59	0.61	0.52	-0.08	0.16	-0.03	-0.07	0.33	0.17	-0.03	0.23	0.47	-0.02	0.36
BM08	0.37	0.25	0.64	0.44	0.12	0.08	0.70	0.22	0.15	0.00	0.32	-0.05	0.57	0.17	-0.09	0.02	0.46	0.24	0.60	0.11
BM09	0.58	0.53	-0.01	0.26	-0.16	0.34	0.82	0.29	-0.10	0.02	0.31	0.17	0.51	-0.03	0.47	0.35	0.16	0.39	0.20	0.08
BM10	0.52	0.28	0.18	0.31	0.67	0.21	0.71	0.46	0.12	-0.13	-0.12	0.00	-0.14	0.32	0.21	0.20	-0.13	0.47	0.36	-0.12
BM11	0.05	0.15	0.62	0.35	0.26	0.46	0.61	0.37	-0.21	0.33	0.58	-0.02	0.17	0.33	-0.04	0.12	0.40	0.52	-0.20	-0.18
BM12	0.21	0.37	0.77	0.54	0.37	0.34	0.50	0.42	0.17	0.16	0.48	0.51	-0.13	0.45	0.34	0.19	0.24	0.51	0.34	0.15
BM13	0.41	-0.11	0.26	0.15	0.28	0.47	0.39	0.41	0.22	0.60	0.10	0.04	-0.04	-0.23	0.34	-0.06	0.21	0.57	0.37	0.33
BM14	-0.08	0.28	0.06	0.68	0.38	0.19	0.31	0.35	0.32	-0.07	0.32	0.41	0.08	0.61	0.11	0.16	0.39	0.22	0.31	0.46
BM15	0.01	0.20	0.59	0.25	0.38	0.34	0.33	0.13	0.04	-0.05	-0.01	0.18	-0.08	0.33	0.29	0.28	0.40	0.60	0.21	0.21
BM16	0.28	0.44	0.59	0.28	-0.01	0.16	0.30	0.19	-0.05	0.45	0.02	0.11	0.35	0.14	-0.03	-0.04	0.01	0.17	0.12	0.03
BM17	0.17	0.28	0.32	0.29	0.51	0.40	0.56	0.32	0.32	0.17	0.23	0.16	-0.28	0.47	0.36	0.50	-0.09	0.45	0.22	-0.15
BM18	0.44	0.49	0.25	-0.17	0.02	0.30	0.00	0.41	0.65	0.18	-0.06	0.05	0.16	0.15	0.12	0.36	0.14	0.28	0.29	0.35
BM19	0.22	0.63	0.49	-0.19	0.60	0.34	0.43	0.39	0.24	-0.35	0.14	0.47	0.29	-0.01	0.06	0.15	0.11	0.48	-0.02	-0.08
BM20	0.48	0.17	0.20	-0.02	-0.13	-0.08	0.40	0.43	0.14	-0.17	0.25	0.25	0.18	0.50	-0.12	-0.19	0.20	-0.03	0.34	0.03
BM21	0.02	0.67	0.44	0.47	-0.02	0.28	0.00	0.12	0.42	0.19	-0.38	-0.05	-0.18	0.24	0.07	0.00	-0.02	-0.12	-0.09	0.18
BM22	-0.05	0.22	0.38	0.27	0.02	0.17	0.02	0.49	0.26	0.03	-0.22	0.47	0.26	0.27	0.04	-0.03	0.22	0.17	0.15	-0.11
BM23	0.18	0.60	-0.20	0.43	0.29	0.58	0.06	-0.05	0.15	0.23	-0.10	-0.17	0.41	0.76	0.39	-0.05	0.46	0.12	0.18	0.62
BM24	0.21	0.51	0.33	0.11	0.61	0.45	0.42	0.24	0.36	0.06	0.25	0.39	0.23	0.49	0.04	-0.03	0.10	0.22	-0.05	0.20

Supplementary Table 28 continued

	Component ICs													*Noise ICs*						
	IC01	IC03	IC05	IC06	IC07	IC08	IC09	IC11	IC14	IC15	IC18	IC19	IC20	IC02	IC04	IC10	IC12	IC13	IC16	IC17
BM25	0.10	-0.05	0.50	0.11	-0.11	0.35	0.22	0.38	0.16	0.01	0.33	0.12	0.22	-0.07	0.42	0.15	-0.28	0.30	-0.11	0.00
BM26	0.12	0.69	-0.03	-0.13	0.24	0.45	0.49	-0.15	-0.04	0.02	-0.03	0.25	0.02	0.46	-0.04	0.51	0.22	-0.11	0.06	-0.11
BM27	-0.01	0.31	0.47	-0.02	0.29	0.54	0.68	0.10	0.47	0.19	0.00	0.05	-0.06	0.42	0.12	-0.14	-0.03	0.06	-0.10	-0.01
BM28	0.52	0.61	0.21	0.04	0.12	-0.03	0.17	0.44	0.39	0.40	0.50	-0.22	0.12	0.07	0.14	-0.01	0.46	0.41	0.18	0.19
BM29	0.39	0.45	0.77	0.41	0.35	0.58	0.64	0.74	0.00	0.27	0.21	0.26	0.49	-0.08	0.05	0.22	0.07	0.04	0.02	-0.05
BM30	0.29	0.46	0.39	-0.18	0.09	0.47	0.55	0.22	-0.02	0.07	-0.03	0.37	0.23	0.35	0.00	-0.17	0.61	0.09	0.17	-0.07
BM31	0.52	0.65	0.03	-0.01	0.11	0.25	0.22	0.26	0.20	0.29	0.40	-0.01	0.33	0.22	0.11	0.29	-0.08	0.15	0.23	0.52
BM32	0.74	0.74	0.15	0.56	0.55	0.29	0.52	0.02	0.22	0.24	0.32	0.46	-0.10	0.54	-0.10	0.00	0.16	0.03	0.37	0.00
BM33	-0.07	0.50	0.19	0.35	0.54	0.53	0.80	-0.20	0.19	0.07	0.34	0.06	0.33	0.70	0.29	0.44	0.51	0.13	-0.03	0.44
BM34	0.21	0.22	0.11	0.42	0.27	-0.17	0.63	0.50	0.12	0.40	0.35	0.23	-0.07	0.31	0.39	0.17	0.33	0.19	-0.02	-0.18
BM35	0.47	0.26	0.32	0.18	0.64	0.19	-0.16	-0.02	0.04	-0.12	0.14	0.40	0.10	0.36	0.11	-0.06	0.26	0.65	0.48	0.19
BM36	0.20	0.28	0.41	0.48	0.30	0.38	0.03	0.37	0.11	0.06	0.11	-0.03	-0.05	0.62	-0.12	0.27	0.02	0.16	0.42	0.15
BM37	0.79	0.25	0.79	0.33	0.62	0.33	0.69	0.72	0.38	0.15	0.20	-0.02	-0.07	0.36	0.34	-0.02	0.12	0.45	0.10	0.08
BM38	0.27	0.75	0.56	0.39	0.46	0.50	0.54	0.47	0.40	0.13	-0.09	-0.07	0.02	0.51	0.09	-0.17	-0.06	0.28	0.00	0.37
BM39	0.24	0.48	0.44	-0.03	0.27	0.31	0.24	0.13	0.36	-0.03	0.41	0.54	0.27	0.68	0.12	-0.12	0.40	0.69	0.30	0.63
BM40	0.23	0.25	-0.12	0.56	0.28	-0.30	0.01	0.35	0.24	0.43	0.09	0.01	0.35	0.55	-0.04	0.28	0.38	0.33	0.19	-0.02
BM41	0.09	0.79	0.64	-0.33	0.20	0.52	0.39	0.28	0.01	0.26	0.30	-0.33	0.08	0.76	0.01	0.37	-0.02	0.42	0.57	0.48
BM42	0.47	0.69	0.58	0.45	0.45	0.56	0.59	-0.05	-0.10	-0.18	0.28	0.12	0.02	0.36	0.22	0.23	0.35	0.17	-0.03	-0.02
BM43	0.04	0.31	0.49	0.62	0.20	0.42	0.66	0.34	0.38	0.00	0.14	0.13	-0.15	0.52	-0.07	0.59	0.08	-0.01	0.19	0.41
BM44	0.05	0.48	0.68	0.26	0.60	0.43	0.58	0.37	0.15	0.20	0.15	0.36	0.25	0.61	0.28	0.03	0.40	0.12	0.14	-0.11
BM45	0.30	0.05	0.37	0.36	0.47	0.11	0.06	-0.18	0.08	0.35	0.53	0.37	-0.15	0.22	0.23	0.38	-0.02	0.25	0.28	0.17
BM46	0.45	0.52	0.58	0.67	0.43	0.14	0.35	0.67	0.11	0.32	0.02	0.12	0.00	0.10	0.41	0.13	0.31	0.43	0.30	-0.10
BM47	0.26	-0.03	0.27	0.17	0.62	0.55	-0.04	-0.16	0.49	0.47	0.17	0.28	0.18	0.40	0.18	0.22	0.41	0.61	0.47	0.03
BM48	-0.22	0.05	0.73	0.21	0.05	0.46	0.78	0.32	0.00	-0.14	0.25	-0.28	-0.01	-0.13	-0.08	-0.11	-0.01	0.10	0.27	-0.09
BM49	0.56	0.19	0.68	0.49	-0.17	0.14	0.57	0.03	0.12	0.01	0.01	0.24	-0.20	-0.02	0.06	-0.04	0.17	0.01	-0.29	-0.21
BM50	0.39	0.18	0.46	0.04	0.21	0.56	0.74	0.36	0.24	0.25	0.28	0.53	-0.13	0.43	0.32	0.51	0.05	0.45	0.44	0.50

Supplementary Table 28 continued

	Component ICs													*Noise ICs*						
	IC01	IC03	IC05	IC06	IC07	IC08	IC09	IC11	IC14	IC15	IC18	IC19	IC20	IC02	IC04	IC10	IC12	IC13	IC16	IC17
BM51	0.23	0.05	0.77	0.24	0.68	0.44	0.58	-0.12	-0.06	-0.07	0.21	0.43	0.22	0.08	0.13	-0.02	0.19	-0.01	-0.11	0.01
BM52	0.20	0.67	0.75	0.22	0.55	0.58	0.14	0.64	0.38	0.05	0.48	0.65	0.10	0.52	0.16	0.03	0.11	0.16	0.10	0.77
BM53	0.52	0.06	0.23	0.47	0.07	0.37	0.50	-0.01	0.35	0.49	0.19	0.36	-0.16	0.35	0.46	0.32	-0.06	0.11	-0.13	0.48
BM54	0.14	0.27	0.09	0.42	0.30	0.73	0.44	0.34	0.26	-0.08	0.17	0.21	-0.07	0.21	0.20	-0.16	0.29	0.22	0.17	0.63
BM55	0.35	0.05	-0.11	0.14	0.55	0.46	-0.04	0.00	0.59	0.28	0.28	0.27	0.43	0.23	-0.06	0.39	0.33	0.20	0.03	0.01
BM56	-0.03	0.13	0.06	0.01	0.45	0.13	0.14	0.01	0.26	-0.03	0.20	0.25	-0.02	0.47	0.49	0.04	0.22	0.53	-0.08	0.37
BM57	0.53	0.30	0.00	-0.05	0.53	0.64	0.18	0.50	0.58	0.52	0.19	0.12	0.20	0.35	-0.03	-0.12	0.07	0.35	0.47	0.61
BM58	0.25	-0.05	-0.14	0.16	0.36	0.06	0.68	0.47	0.40	0.04	0.23	0.08	0.01	-0.02	0.35	-0.12	0.53	0.34	0.20	0.52
BM59	0.19	0.09	0.41	0.21	-0.04	0.09	0.13	-0.21	0.13	0.16	-0.13	0.35	0.54	0.56	-0.09	0.05	-0.01	0.35	-0.03	-0.03
BM60	0.36	0.35	-0.05	0.17	0.16	0.03	-0.09	0.12	0.33	-0.01	0.54	-0.10	0.13	0.50	0.40	0.21	0.08	0.47	-0.08	0.00
BM61	0.55	0.62	0.64	0.47	0.16	0.45	0.29	0.28	-0.07	-0.03	0.27	0.37	0.10	0.65	0.15	0.65	0.07	0.24	0.09	0.24
BM62	0.53	0.79	0.49	0.06	0.47	0.15	0.76	0.66	0.21	0.25	-0.06	0.20	-0.01	0.28	-0.05	0.19	0.09	0.19	0.18	-0.06
BM63	-0.07	-0.02	0.20	0.24	0.09	0.71	0.00	0.19	0.02	0.20	0.14	0.11	0.08	0.20	0.14	0.15	0.39	0.16	0.00	0.20
BM64	0.25	0.60	0.67	0.36	0.42	0.07	0.52	0.29	0.18	-0.25	0.21	-0.04	-0.30	0.16	-0.29	-0.11	0.22	0.05	0.28	0.00
BM65	0.17	0.20	-0.11	0.42	0.01	-0.04	0.04	0.31	-0.22	0.17	-0.12	-0.03	0.13	0.07	0.16	0.07	0.10	0.71	0.17	0.07
BM66	0.73	-0.07	0.26	-0.15	0.30	0.34	0.41	0.06	0.38	0.45	0.29	0.01	-0.03	0.47	-0.09	0.18	0.45	0.07	0.40	0.15
BM67	0.29	0.05	-0.01	0.19	0.31	0.40	0.02	0.04	0.40	0.31	0.48	0.22	0.14	0.51	0.62	-0.12	0.46	0.07	-0.18	-0.04
BM68	0.67	0.66	0.72	-0.17	0.06	0.28	0.74	0.56	-0.08	-0.04	0.02	0.30	-0.12	0.48	0.18	0.06	0.39	-0.04	0.46	-0.01
BM69	0.36	0.48	0.41	0.48	0.12	0.37	0.48	0.20	0.53	-0.07	0.31	0.09	0.37	0.55	0.56	0.15	0.43	0.40	0.35	0.15
BM70	0.40	0.49	0.09	0.09	-0.01	0.02	0.48	-0.06	0.51	0.03	0.38	0.42	-0.25	0.72	0.33	0.34	0.14	0.31	0.02	0.44

Supplementary Table 29. Within-session ICC values for the NYU-TRT group ICA data atlased using the SMITH10 atlas. *I_i: ICN$_i$ Spatial Involvement; IR_i: ICN$_i$ Relative Spatial Involvement; MA_i: Mean ICN$_i$ Activation ; $MA_{N,i}$: Normalised Mean ICN$_i$ Activation ; IR_i^M: Relative Normalised Mean ICN$_i$ Activation; $RA_{N,i}$: Normalised Relative ICN$_i$ Activation ; I_i^M: Normalised Mean ICN$_i$ Activation Density; OL_i: Spatial Overlap with ICNi; SQ: Sørensen-Dice coefficient with ICN$_i$; J_i: Jaccard index with ICN$_i$, and r_i: Pearson's spatial correlation, overall: data collapsed across all atlas base maps. See Table 1 for the description of atlas ICNs (ICN01-ICN10). For visual representation see Figure 7 panel D.*

	I_i	IR_i	MA_i	$MA_{N,i}$	IR_i^M	$RA_{N,i}$	I_i^M	OL_i	SQ	J_i	r_i
ICN01	0.97	0.95	0.76	0.77	0.97	0.96	0.97	0.98	0.98	0.97	0.99
ICN02	0.86	0.86	0.77	0.80	0.87	0.87	0.83	0.90	0.90	0.89	0.88
ICN03	0.82	0.85	0.57	0.65	0.85	0.85	0.80	0.88	0.89	0.89	0.89
ICN04	0.94	0.91	0.79	0.80	0.95	0.93	0.94	0.96	0.97	0.97	0.98
ICN05	0.86	0.89	0.71	0.76	0.89	0.90	0.84	0.91	0.90	0.88	0.91
ICN06	0.93	0.91	0.62	0.69	0.94	0.93	0.91	0.95	0.95	0.96	0.96
ICN07	0.95	0.88	0.80	0.83	0.91	0.90	0.95	0.95	0.95	0.95	0.96
ICN08	0.87	0.87	0.75	0.79	0.88	0.88	0.86	0.90	0.90	0.91	0.92
ICN09	0.88	0.85	0.77	0.81	0.87	0.85	0.86	0.92	0.92	0.92	0.93
ICN10	0.93	0.90	0.82	0.84	0.93	0.91	0.93	0.95	0.94	0.95	0.96
**overall**	0.92	0.90	0.76	0.79	0.92	0.91	0.91	0.94	0.94	0.94	0.95

Supplementary Table 30. Between-session ICC values for the NYU-TRT group ICA data atlased using the SMITH10 atlas. *I_i: ICN_i Spatial Involvement; IR_i: ICN_i Relative Spatial Involvement; MA_i: Mean ICN_i Activation ; $MA_{N,i}$: Normalised Mean ICN_i Activation ; IR_i^M: Relative Normalised Mean ICN_i Activation; $RA_{N,i}$: Normalised Relative ICN_i Activation ; I_i^M: Normalised Mean ICN_i Activation Density; OL_i: Spatial Overlap with ICNi; SQ: Sørensen-Dice coefficient with ICN_i; J_i: Jaccard index with ICN_i, and r_i: Pearson's spatial correlation, overall: data collapsed across all atlas base maps. See Table 1 for the description of atlas ICNs (ICN01-ICN10). For visual representation see Figure 7 panel H.*

	I_i	IR_i	MA_i	$MA_{N,i}$	IR_i^M	$RA_{N,i}$	I_i^M	OL_i	SQ	J_i	r_i
ICN01	0.97	0.96	0.81	0.69	0.97	0.96	0.97	0.99	0.98	0.99	0.99
ICN02	0.87	0.89	0.75	0.63	0.88	0.89	0.86	0.91	0.91	0.90	0.90
ICN03	0.81	0.86	0.67	0.48	0.85	0.86	0.79	0.89	0.90	0.90	0.89
ICN04	0.93	0.93	0.77	0.64	0.94	0.94	0.94	0.96	0.97	0.97	0.98
ICN05	0.90	0.91	0.76	0.63	0.90	0.93	0.87	0.93	0.93	0.91	0.94
ICN06	0.92	0.93	0.67	0.49	0.95	0.94	0.90	0.95	0.95	0.96	0.96
ICN07	0.93	0.89	0.78	0.60	0.91	0.91	0.92	0.95	0.95	0.95	0.96
ICN08	0.84	0.89	0.75	0.54	0.88	0.89	0.84	0.90	0.91	0.91	0.92
ICN09	0.86	0.88	0.77	0.56	0.89	0.88	0.87	0.92	0.92	0.92	0.93
ICN10	0.90	0.92	0.75	0.60	0.93	0.93	0.90	0.95	0.95	0.95	0.96
**overall**	0.91	0.91	0.76	0.61	0.92	0.92	0.91	0.94	0.95	0.95	0.96

Supplementary Table 31. Within-session ICC values for the NYU-TRT group ICA data atlased using the BRAINMAP20 atlas. *I_i: ICN$_i$ Spatial Involvement; IR_i: ICN$_i$ Relative Spatial Involvement; MA_i: Mean ICN$_i$ Activation ; $MA_{N,i}$: Normalised Mean ICN$_i$ Activation ; IR_i^M: Relative Normalised Mean ICN$_i$ Activation; $RA_{N,i}$: Normalised Relative ICN$_i$ Activation ; I_i^M: Normalised Mean ICN$_i$ Activation Density; OL_i: Spatial Overlap with ICNi; SQ: Sørensen-Dice coefficient with ICN$_i$; J_i: Jaccard index with ICN$_i$, and r_i: Pearson's spatial correlation, overall: data collapsed across all atlas base maps. See Table 1 for the description of atlas ICNs (BM01-BM20). For visual representation see Figure 7 panel L.*

	I_i	IR_i	MA_i	$MA_{N,i}$	IR_i^M	$RA_{N,i}$	I_i^M	OL_i	SQ	J_i	r_i
BM01	0.80	0.89	0.71	0.78	0.90	0.90	0.81	0.88	0.88	0.88	0.90
BM02	0.83	0.87	0.67	0.74	0.91	0.90	0.81	0.89	0.89	0.90	0.90
BM03	0.77	0.85	0.60	0.69	0.86	0.85	0.77	0.83	0.83	0.83	0.79
BM04	0.93	0.89	0.77	0.81	0.91	0.91	0.93	0.93	0.92	0.93	0.95
BM05	0.81	0.88	0.75	0.80	0.88	0.89	0.79	0.88	0.87	0.87	0.90
BM06	0.93	0.89	0.66	0.71	0.92	0.91	0.91	0.95	0.94	0.95	0.96
BM07	0.89	0.89	0.79	0.82	0.91	0.90	0.89	0.92	0.92	0.93	0.93
BM08	0.91	0.89	0.74	0.80	0.92	0.91	0.89	0.93	0.93	0.93	0.93
BM09	0.91	0.87	0.53	0.55	0.90	0.90	0.88	0.93	0.92	0.92	0.94
BM10	0.71	0.80	0.57	0.68	0.78	0.79	0.69	0.78	0.79	0.79	0.75
BM11	0.86	0.89	0.76	0.79	0.91	0.90	0.84	0.91	0.91	0.90	0.90
BM12	0.95	0.96	0.86	0.88	0.98	0.97	0.96	0.98	0.97	0.97	0.99
BM13	0.92	0.93	0.81	0.84	0.95	0.94	0.92	0.95	0.95	0.96	0.97
BM14	0.88	0.87	0.61	0.70	0.88	0.87	0.86	0.91	0.90	0.89	0.91
BM15	0.86	0.85	0.72	0.77	0.87	0.86	0.84	0.89	0.89	0.90	0.90
BM16	0.90	0.85	0.75	0.79	0.87	0.86	0.89	0.90	0.89	0.89	0.89
BM17	0.89	0.81	0.59	0.69	0.85	0.83	0.87	0.89	0.87	0.88	0.86
BM18	0.93	0.89	0.78	0.81	0.94	0.92	0.93	0.94	0.93	0.93	0.95
BM19	0.66	0.84	0.72	0.78	0.85	0.85	0.68	0.77	0.76	0.76	0.81
BM20	0.87	0.85	0.80	0.83	0.88	0.86	0.86	0.90	0.90	0.91	0.90
overall	0.89	0.90	0.73	0.78	0.92	0.91	0.89	0.92	0.92	0.92	0.93

Supplementary Table 32. Between-session ICC values for the NYU-TRT group ICA data atlased using the BRAINMAP20 atlas. *I_i: ICN$_i$ Spatial Involvement; IR_i: ICN$_i$ Relative Spatial Involvement; MA_i: Mean ICN$_i$ Activation ; $MA_{N,i}$: Normalised Mean ICN$_i$ Activation ; IR_i^M: Relative Normalised Mean ICN$_i$ Activation; $RA_{N,i}$: Normalised Relative ICN$_i$ Activation ; I_i^M: Normalised Mean ICN$_i$ Activation Density; OL_i: Spatial Overlap with ICNi; SQ: Sørensen-Dice coefficient with ICN$_i$; J_i: Jaccard index with ICN$_i$, and r_i: Pearson's spatial correlation, overall: data collapsed across all atlas base maps. See Table 1 for the description of atlas ICNs (BM01-BM20). For visual representation see Figure 7 panel P.*

	I_i	IR_i	MA_i	$MA_{N,i}$	IR_i^M	$RA_{N,i}$	I_i^M	OL_i	SQ	J_i	r_i
BM01	0.79	0.90	0.72	0.55	0.90	0.91	0.81	0.90	0.90	0.90	0.93
BM02	0.79	0.90	0.72	0.62	0.89	0.91	0.79	0.88	0.88	0.88	0.90
BM03	0.82	0.84	0.65	0.51	0.83	0.84	0.81	0.86	0.85	0.84	0.84
BM04	0.92	0.91	0.75	0.62	0.92	0.92	0.91	0.94	0.94	0.94	0.96
BM05	0.84	0.91	0.77	0.65	0.90	0.92	0.82	0.92	0.91	0.91	0.93
BM06	0.89	0.88	0.69	0.57	0.91	0.90	0.87	0.93	0.93	0.93	0.94
BM07	0.89	0.92	0.80	0.68	0.93	0.93	0.90	0.94	0.94	0.94	0.95
BM08	0.90	0.91	0.72	0.60	0.92	0.93	0.88	0.94	0.94	0.94	0.94
BM09	0.91	0.85	0.58	0.53	0.90	0.88	0.89	0.93	0.91	0.91	0.94
BM10	0.67	0.79	0.47	0.32	0.77	0.79	0.64	0.77	0.79	0.79	0.75
BM11	0.86	0.92	0.81	0.72	0.91	0.92	0.85	0.92	0.92	0.91	0.91
BM12	0.96	0.97	0.87	0.78	0.97	0.98	0.96	0.98	0.98	0.99	0.99
BM13	0.92	0.94	0.79	0.67	0.95	0.95	0.93	0.95	0.96	0.96	0.96
BM14	0.87	0.90	0.55	0.46	0.90	0.91	0.86	0.92	0.91	0.90	0.92
BM15	0.84	0.89	0.73	0.60	0.90	0.90	0.85	0.90	0.90	0.91	0.91
BM16	0.87	0.86	0.76	0.62	0.88	0.88	0.88	0.90	0.89	0.89	0.91
BM17	0.85	0.86	0.67	0.48	0.87	0.87	0.83	0.88	0.88	0.89	0.87
BM18	0.88	0.89	0.66	0.58	0.92	0.91	0.89	0.92	0.92	0.92	0.93
BM19	0.62	0.87	0.69	0.53	0.87	0.88	0.66	0.77	0.76	0.76	0.83
BM20	0.84	0.87	0.77	0.66	0.87	0.87	0.86	0.89	0.90	0.90	0.89
overall	0.88	0.92	0.73	0.62	0.92	0.93	0.88	0.92	0.93	0.93	0.94

Supplementary Table 33. Within-session ICC values for the NYU-TRT group ICA data atlased using the BRAINMAP70 atlas. *I_i: ICN_i Spatial Involvement; IR_i: ICN_i Relative Spatial Involvement; MA_i: Mean ICN_i Activation ; $MA_{N,i}$: Normalised Mean ICN_i Activation ; IR_i^M: Relative Normalised Mean ICN_i Activation; $RA_{N,i}$: Normalised Relative ICN_i Activation ; I_i^M: Normalised Mean ICN_i Activation Density; OL_i: Spatial Overlap with ICNi; SQ: Sørensen-Dice coefficient with ICN_i; J_i: Jaccard index with ICN_i, and r_i: Pearson's spatial correlation, overall: data collapsed across all atlas base maps. See Supplementary Table 1 for the description of atlas ICNs (BM01-BM70). For visual representation see Supplementary Figure 7 panel D.*

	I_i	IR_i	MA_i	$MA_{N,i}$	IR_i^M	$RA_{N,i}$	I_i^M	OL_i	SQ	J_i	r_i
BM01	0.94	0.93	0.55	0.56	0.95	0.94	0.95	0.96	0.94	0.94	0.97
BM02	0.96	0.93	0.42	0.44	0.96	0.95	0.96	0.96	0.94	0.93	0.97
BM03	0.85	0.86	0.69	0.74	0.87	0.86	0.83	0.89	0.88	0.87	0.87
BM04	0.69	0.76	0.47	0.53	0.74	0.75	0.67	0.74	0.76	0.75	0.71
BM05	0.83	0.89	0.33	0.33	0.92	0.90	0.86	0.89	0.89	0.89	0.88
BM06	0.82	0.79	0.36	0.37	0.84	0.82	0.84	0.84	0.82	0.82	0.86
BM07	0.88	0.84	0.67	0.70	0.88	0.86	0.89	0.89	0.86	0.86	0.91
BM08	0.71	0.70	0.48	0.47	0.74	0.72	0.70	0.75	0.73	0.73	0.76
BM09	0.87	0.86	0.39	0.41	0.87	0.86	0.87	0.89	0.87	0.87	0.90
BM10	0.85	0.82	0.55	0.61	0.84	0.83	0.83	0.88	0.87	0.87	0.88
BM11	0.82	0.76	0.45	0.48	0.80	0.78	0.82	0.82	0.81	0.81	0.83
BM12	0.85	0.78	0.49	0.48	0.81	0.80	0.83	0.85	0.81	0.80	0.84
BM13	0.72	0.63	0.38	0.38	0.67	0.64	0.70	0.72	0.68	0.68	0.68
BM14	0.66	0.69	0.46	0.49	0.70	0.69	0.67	0.70	0.71	0.71	0.70
BM15	0.91	0.77	0.30	0.32	0.85	0.83	0.91	0.89	0.84	0.84	0.92
BM16	0.91	0.84	0.33	0.34	0.89	0.87	0.89	0.92	0.89	0.89	0.93
BM17	0.87	0.80	0.39	0.39	0.83	0.81	0.86	0.87	0.84	0.84	0.87
BM18	0.76	0.73	0.38	0.42	0.74	0.75	0.75	0.75	0.73	0.73	0.75
BM19	0.72	0.73	0.37	0.40	0.74	0.73	0.72	0.75	0.75	0.75	0.73
BM20	0.83	0.84	0.45	0.50	0.90	0.88	0.84	0.87	0.85	0.86	0.89
BM21	0.86	0.85	0.56	0.60	0.88	0.86	0.82	0.89	0.89	0.88	0.88
BM22	0.76	0.73	0.36	0.39	0.74	0.73	0.75	0.77	0.77	0.76	0.77
BM23	0.84	0.89	0.59	0.60	0.90	0.91	0.86	0.89	0.88	0.88	0.91
BM24	0.80	0.75	0.55	0.57	0.81	0.80	0.83	0.82	0.80	0.80	0.82

Supplementary Table 33 continued

	I_i	IR_i	MA_i	$MA_{N,i}$	IR_i^M	$RA_{N,i}$	I_i^M	OL_i	SQ	J_i	r_i
BM25	0.79	0.70	0.34	0.36	0.72	0.71	0.81	0.77	0.73	0.73	0.79
BM26	0.60	0.48	0.27	0.26	0.51	0.50	0.60	0.56	0.50	0.50	0.59
BM27	0.80	0.75	0.46	0.48	0.78	0.76	0.80	0.80	0.78	0.78	0.80
BM28	0.88	0.84	0.59	0.64	0.87	0.86	0.88	0.89	0.87	0.88	0.90
BM29	0.84	0.79	0.49	0.50	0.85	0.82	0.87	0.84	0.82	0.82	0.87
BM30	0.79	0.71	0.43	0.44	0.75	0.71	0.81	0.79	0.76	0.76	0.78
BM31	0.73	0.69	0.54	0.57	0.71	0.70	0.73	0.72	0.70	0.70	0.70
BM32	0.79	0.84	0.45	0.45	0.86	0.84	0.80	0.85	0.85	0.85	0.84
BM33	0.86	0.79	0.41	0.44	0.84	0.83	0.85	0.86	0.83	0.83	0.89
BM34	0.91	0.84	0.35	0.37	0.89	0.88	0.88	0.92	0.89	0.90	0.93
BM35	0.91	0.81	0.45	0.45	0.87	0.86	0.89	0.91	0.87	0.87	0.93
BM36	0.82	0.77	0.39	0.39	0.81	0.79	0.82	0.84	0.82	0.83	0.87
BM37	0.82	0.72	0.53	0.54	0.75	0.72	0.81	0.81	0.77	0.77	0.80
BM38	0.86	0.82	0.42	0.43	0.85	0.83	0.86	0.88	0.85	0.85	0.88
BM39	0.78	0.89	0.55	0.57	0.89	0.90	0.79	0.86	0.88	0.87	0.87
BM40	0.89	0.85	0.43	0.46	0.88	0.87	0.85	0.91	0.89	0.90	0.91
BM41	0.86	0.87	0.57	0.59	0.89	0.88	0.87	0.89	0.88	0.88	0.90
BM42	0.66	0.67	0.42	0.46	0.68	0.68	0.65	0.69	0.71	0.71	0.66
BM43	0.64	0.62	0.36	0.36	0.63	0.62	0.63	0.63	0.62	0.62	0.61
BM44	0.87	0.77	0.46	0.48	0.79	0.78	0.87	0.85	0.82	0.82	0.84
BM45	0.81	0.80	0.40	0.41	0.85	0.85	0.80	0.84	0.83	0.83	0.83
BM46	0.87	0.80	0.37	0.37	0.82	0.81	0.86	0.86	0.83	0.83	0.85
BM47	0.88	0.81	0.36	0.36	0.83	0.82	0.88	0.87	0.83	0.83	0.88
BM48	0.73	0.71	0.49	0.50	0.78	0.75	0.76	0.75	0.73	0.74	0.77
BM49	0.89	0.82	0.44	0.47	0.88	0.85	0.91	0.89	0.85	0.85	0.89
BM50	0.85	0.82	0.38	0.40	0.85	0.83	0.85	0.87	0.85	0.85	0.88
BM51	0.89	0.77	0.49	0.50	0.86	0.83	0.90	0.88	0.81	0.81	0.90

Supplementary Table 33 continued

	I_i	IR_i	MA_i	$MA_{N,i}$	IR_i^M	$RA_{N,i}$	I_i^M	OL_i	SQ	J_i	r_i
BM52	0.95	0.92	0.62	0.67	0.94	0.94	0.96	0.95	0.94	0.94	0.97
BM53	0.81	0.82	0.65	0.69	0.84	0.84	0.78	0.84	0.83	0.84	0.83
BM54	0.66	0.78	0.46	0.49	0.79	0.78	0.65	0.74	0.77	0.77	0.69
BM55	0.81	0.80	0.20	0.21	0.81	0.81	0.82	0.82	0.81	0.81	0.79
BM56	0.81	0.89	0.62	0.66	0.88	0.88	0.82	0.90	0.90	0.90	0.88
BM57	0.63	0.62	0.36	0.36	0.62	0.62	0.62	0.65	0.64	0.63	0.66
BM58	0.85	0.83	0.30	0.33	0.84	0.84	0.86	0.87	0.85	0.85	0.87
BM59	0.77	0.76	0.41	0.45	0.77	0.76	0.76	0.79	0.78	0.77	0.77
BM60	0.90	0.87	0.40	0.42	0.87	0.87	0.88	0.92	0.88	0.87	0.92
BM61	0.95	0.94	0.63	0.64	0.97	0.96	0.95	0.96	0.96	0.96	0.97
BM62	0.86	0.86	0.61	0.61	0.89	0.87	0.86	0.89	0.88	0.88	0.89
BM63	0.84	0.69	0.31	0.33	0.73	0.69	0.82	0.81	0.75	0.75	0.77
BM64	0.76	0.78	0.29	0.33	0.79	0.79	0.74	0.81	0.80	0.80	0.79
BM65	0.78	0.84	0.41	0.46	0.86	0.87	0.79	0.82	0.80	0.79	0.85
BM66	0.86	0.84	0.51	0.54	0.87	0.86	0.85	0.89	0.89	0.88	0.89
BM67	0.53	0.66	0.45	0.45	0.65	0.66	0.55	0.60	0.64	0.63	0.64
BM68	0.76	0.77	0.61	0.65	0.82	0.81	0.74	0.80	0.79	0.79	0.81
BM69	0.81	0.79	0.79	0.82	0.82	0.81	0.80	0.85	0.84	0.84	0.84
BM70	0.65	0.79	0.70	0.76	0.80	0.79	0.67	0.74	0.74	0.74	0.78
overall	0.85	0.85	0.47	0.48	0.88	0.87	0.86	0.87	0.87	0.87	0.88

Supplementary Table 34. Between-session ICC values for the NYU-TRT group ICA data atlased using the BRAINMAP70 atlas. *I_i: ICN$_i$ Spatial Involvement; IR_i: ICN$_i$ Relative Spatial Involvement; MA_i: Mean ICN$_i$ Activation ; $MA_{N,i}$: Normalised Mean ICN$_i$ Activation ; IR_i^M: Relative Normalised Mean ICN$_i$ Activation; $RA_{N,i}$: Normalised Relative ICN$_i$ Activation ; I_i^M: Normalised Mean ICN$_i$ Activation Density; OL_i: Spatial Overlap with ICNi; SQ: Sørensen-Dice coefficient with ICN$_i$; J_i: Jaccard index with ICN$_i$, and r_i: Pearson's spatial correlation, overall: data collapsed across all atlas base maps. See Supplementary Table 1 for the description of atlas ICNs (BM01-BM70). For visual representation see Supplementary Figure 7 panel H.*

	I_i	IR_i	MA_i	$MA_{N,i}$	IR_i^M	$RA_{N,i}$	I_i^M	OL_i	SQ	J_i	r_i
BM01	0.95	0.95	0.56	0.57	0.96	0.96	0.96	0.97	0.96	0.96	0.98
BM02	0.97	0.95	0.54	0.51	0.96	0.97	0.97	0.97	0.96	0.96	0.97
BM03	0.86	0.89	0.70	0.63	0.88	0.90	0.85	0.90	0.90	0.90	0.90
BM04	0.71	0.77	0.35	0.30	0.75	0.77	0.70	0.77	0.78	0.77	0.76
BM05	0.83	0.89	0.29	0.27	0.90	0.90	0.85	0.89	0.90	0.90	0.87
BM06	0.82	0.84	0.44	0.44	0.86	0.85	0.84	0.86	0.85	0.85	0.86
BM07	0.90	0.87	0.69	0.63	0.90	0.89	0.92	0.91	0.90	0.90	0.93
BM08	0.72	0.76	0.55	0.54	0.77	0.77	0.71	0.78	0.79	0.79	0.79
BM09	0.87	0.88	0.45	0.42	0.88	0.87	0.86	0.90	0.90	0.90	0.89
BM10	0.83	0.83	0.47	0.41	0.85	0.85	0.82	0.87	0.87	0.87	0.89
BM11	0.82	0.81	0.44	0.39	0.83	0.83	0.83	0.85	0.85	0.85	0.86
BM12	0.83	0.79	0.57	0.56	0.80	0.80	0.82	0.84	0.81	0.81	0.83
BM13	0.71	0.69	0.37	0.37	0.71	0.70	0.72	0.73	0.72	0.72	0.70
BM14	0.66	0.69	0.32	0.29	0.69	0.69	0.67	0.71	0.71	0.71	0.71
BM15	0.87	0.79	0.44	0.42	0.85	0.83	0.87	0.88	0.84	0.84	0.90
BM16	0.86	0.83	0.38	0.34	0.85	0.85	0.83	0.88	0.87	0.87	0.89
BM17	0.85	0.75	0.54	0.53	0.78	0.76	0.85	0.85	0.79	0.78	0.85
BM18	0.78	0.77	0.47	0.40	0.78	0.79	0.77	0.82	0.80	0.80	0.81
BM19	0.67	0.76	0.44	0.41	0.74	0.76	0.67	0.74	0.76	0.76	0.73
BM20	0.83	0.83	0.50	0.47	0.86	0.86	0.84	0.87	0.86	0.85	0.88
BM21	0.82	0.84	0.55	0.53	0.84	0.84	0.81	0.86	0.85	0.85	0.85
BM22	0.74	0.77	0.36	0.33	0.76	0.76	0.72	0.79	0.79	0.78	0.80
BM23	0.84	0.86	0.60	0.56	0.88	0.89	0.86	0.89	0.87	0.87	0.91
BM24	0.81	0.76	0.55	0.50	0.78	0.80	0.81	0.83	0.80	0.79	0.83

Supplementary Table 34 continued

	I_i	IR_i	MA_i	$MA_{N,i}$	IR_i^M	$RA_{N,i}$	I_i^M	OL_i	SQ	J_i	r_i
BM25	0.82	0.72	0.41	0.37	0.76	0.75	0.81	0.82	0.78	0.78	0.84
BM26	0.55	0.48	0.28	0.27	0.52	0.51	0.56	0.56	0.52	0.52	0.62
BM27	0.80	0.74	0.52	0.50	0.76	0.75	0.81	0.80	0.77	0.78	0.80
BM28	0.88	0.88	0.56	0.51	0.90	0.90	0.89	0.91	0.91	0.91	0.92
BM29	0.84	0.82	0.52	0.50	0.85	0.84	0.85	0.86	0.85	0.85	0.87
BM30	0.70	0.68	0.48	0.47	0.72	0.70	0.75	0.72	0.70	0.69	0.71
BM31	0.73	0.76	0.41	0.40	0.77	0.77	0.73	0.76	0.75	0.75	0.74
BM32	0.85	0.85	0.47	0.46	0.86	0.86	0.84	0.88	0.87	0.87	0.89
BM33	0.88	0.85	0.58	0.53	0.88	0.88	0.86	0.91	0.89	0.90	0.93
BM34	0.92	0.85	0.40	0.38	0.90	0.89	0.89	0.92	0.90	0.90	0.94
BM35	0.92	0.83	0.41	0.41	0.88	0.88	0.90	0.92	0.88	0.89	0.94
BM36	0.81	0.78	0.49	0.47	0.80	0.80	0.80	0.84	0.83	0.83	0.87
BM37	0.81	0.78	0.42	0.41	0.79	0.78	0.80	0.84	0.82	0.82	0.82
BM38	0.83	0.83	0.48	0.49	0.86	0.85	0.84	0.85	0.84	0.84	0.86
BM39	0.78	0.88	0.53	0.47	0.88	0.89	0.78	0.87	0.89	0.89	0.88
BM40	0.89	0.84	0.51	0.48	0.88	0.87	0.86	0.91	0.89	0.89	0.92
BM41	0.87	0.88	0.59	0.53	0.89	0.89	0.88	0.91	0.90	0.90	0.92
BM42	0.63	0.66	0.43	0.37	0.66	0.66	0.62	0.68	0.69	0.69	0.65
BM43	0.51	0.60	0.44	0.40	0.59	0.60	0.48	0.59	0.62	0.62	0.55
BM44	0.87	0.83	0.53	0.47	0.85	0.84	0.87	0.88	0.86	0.86	0.88
BM45	0.80	0.84	0.49	0.44	0.85	0.87	0.79	0.87	0.87	0.87	0.85
BM46	0.84	0.79	0.50	0.49	0.82	0.81	0.84	0.85	0.82	0.82	0.85
BM47	0.85	0.80	0.38	0.37	0.83	0.81	0.85	0.86	0.83	0.83	0.87
BM48	0.61	0.51	0.53	0.52	0.61	0.57	0.69	0.61	0.56	0.55	0.65
BM49	0.85	0.85	0.37	0.35	0.89	0.88	0.85	0.88	0.87	0.88	0.89
BM50	0.83	0.85	0.58	0.56	0.86	0.86	0.83	0.87	0.87	0.87	0.89
BM51	0.84	0.75	0.53	0.52	0.83	0.81	0.87	0.84	0.79	0.79	0.87

Supplementary Table 34 continued

	I_i	IR_i	MA_i	$MA_{N,i}$	IR_i^M	$RA_{N,i}$	I_i^M	OL_i	SQ	J_i	r_i
BM52	0.94	0.92	0.60	0.53	0.94	0.94	0.94	0.95	0.93	0.94	0.97
BM53	0.74	0.87	0.61	0.55	0.87	0.88	0.76	0.82	0.84	0.84	0.80
BM54	0.74	0.81	0.45	0.41	0.80	0.81	0.71	0.79	0.80	0.80	0.75
BM55	0.81	0.81	0.24	0.23	0.81	0.81	0.80	0.83	0.82	0.82	0.80
BM56	0.83	0.90	0.64	0.58	0.89	0.91	0.83	0.91	0.92	0.91	0.89
BM57	0.64	0.65	0.44	0.43	0.66	0.66	0.63	0.68	0.67	0.67	0.69
BM58	0.86	0.82	0.49	0.46	0.82	0.83	0.84	0.89	0.85	0.84	0.88
BM59	0.75	0.82	0.38	0.40	0.81	0.81	0.75	0.81	0.82	0.82	0.80
BM60	0.92	0.86	0.45	0.42	0.88	0.88	0.90	0.93	0.89	0.88	0.95
BM61	0.94	0.95	0.72	0.67	0.96	0.97	0.95	0.97	0.96	0.96	0.97
BM62	0.88	0.87	0.57	0.55	0.88	0.88	0.88	0.90	0.89	0.89	0.90
BM63	0.80	0.73	0.35	0.33	0.76	0.75	0.79	0.81	0.78	0.78	0.79
BM64	0.69	0.76	0.31	0.30	0.76	0.76	0.67	0.76	0.78	0.78	0.77
BM65	0.80	0.87	0.43	0.44	0.88	0.90	0.78	0.86	0.86	0.86	0.87
BM66	0.87	0.90	0.56	0.52	0.90	0.90	0.88	0.91	0.90	0.90	0.90
BM67	0.54	0.62	0.38	0.37	0.61	0.62	0.55	0.62	0.64	0.64	0.62
BM68	0.68	0.76	0.63	0.56	0.78	0.79	0.69	0.76	0.77	0.77	0.77
BM69	0.79	0.82	0.77	0.67	0.83	0.84	0.81	0.85	0.85	0.85	0.85
BM70	0.62	0.82	0.68	0.57	0.83	0.84	0.65	0.74	0.75	0.75	0.80
overall	0.84	0.87	0.51	0.49	0.88	0.89	0.85	0.88	0.88	0.88	0.89
